# Molecular Editing of 5‑Alkynyl-1,2,3-triazines via a Silver-Mediated Skeletal Remodeling Approach: Solvent-Controlled Switchable Synthesis of Functionalized Pyrroles and Furans

**DOI:** 10.1021/acs.joc.5c01536

**Published:** 2025-10-22

**Authors:** Hsiang-Wen Chen, Wan-Hsuan Liu, Chia-Hao Chang, Jiun-Jie Shie

**Affiliations:** Institute of Chemistry, 38017 Academia Sinica, Taipei 11529, Taiwan

## Abstract

We present a skeletal remodeling approach using 5-alkynyl-1,2,3-triazines (**3**) for the switchable construction of functionalized pyrroles (**4**) and furans (**5**). This flexible and adaptable method for heterocycle editing involves the nucleophilic ring opening of 1,2,3-triazines, followed by subsequent cyclization. The process can be finely tuned by adjusting the silver-mediated conditions and solvent systems. This protocol provides a new avenue for the efficient synthesis of five-membered aromatic heterocycles.

## Graphical abstract

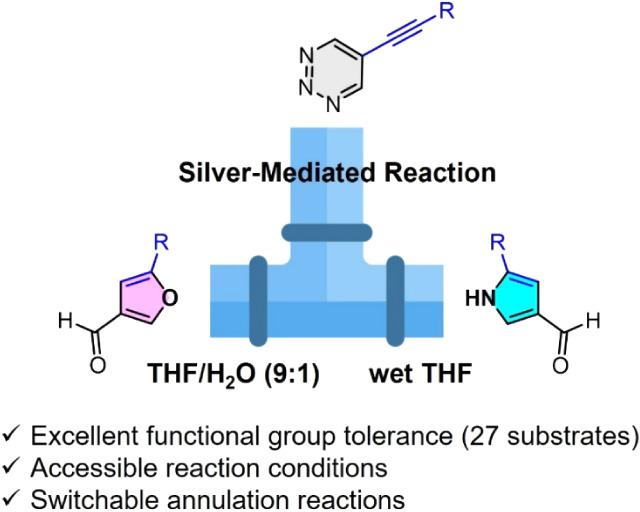

## Introduction

Five-membered aromatic heterocycles are key structural motifs of various natural products, pharmaceuticals, and optical materials.^1a,1b^ Among them, functionalized pyrrole and furan molecules represent a class of essential synthetic intermediates for accessing other valuable compounds with applications in medicine and advanced materials.^1c–1f^ The synthesis of these structural building blocks has received significant attention, and various elegant methods have been developed to prepare functionalized pyrroles and furans.^2^ In recent years, chemists have been continuously interested in the regioselective synthesis of polysubstituted furans and pyrroles. Most methods for constructing these heterocyclic skeletons from acyclic dicarbonyl precursors rely on condensation–cyclization reactions, including the traditional Paal–Knorr,^3^ Clauson–Kaas, Feist–Bénary, and Hantzsch reactions^4^ for the synthesis of fully substituted furan and pyrrole derivatives. These well-known classical methods have a limited substrate scope owing to their functional group tolerance because they typically employ a strong acid under relatively harsh conditions. To overcome these limitations, several synthetic strategies have been developed for converting alkenes and alkynes into functionalized furans and pyrroles, thereby creating complex molecular skeletons. These strategies include cyclization processes,^5^ as well as ring-closing,^6^ ring-expansion,^7^ rearrangement,^8^ heteroannulation,^9^ and multicomponent^10^ reactions. Although these effective approaches have led to significant advances in the field, the development of new protocols for the synthesis of heterocycles remains a compelling and ongoing area of research in organic synthesis. As significant synthon components of high-value compounds, 3-formylpyrrole and furan derivatives serve as precursors for various functional materials, bioactive compounds, and natural products. The formylation of pyrrole and furan derivatives typically involves common Vilsmeier–Haack-type reactions, which often face issues related to regioselective formylation.^11^ Several methods have been devised to synthesize 3-formylpyrrole and furan derivatives via a sequential multicomponent reaction using *in situ*-generated aldimines and 1,4-ketoaldehydes.^12^ However, achieving the regioselective synthesis of substituted pyrrole- and furan-3-carbaldehydes via these synthetic methods remains a challenging task. Moreover, practical approaches based on readily available starting materials, cost-effective catalysts, and straightforward procedures have garnered significant interest in the promotion of sustainable development and optimal synthesis processes. In particular, methods for the flexible synthesis of pyrrole and furan derivatives from the same starting materials are attractive and remain largely unexplored. Herein, we present an alternative skeletal remodeling approach for the switchable synthesis of 5-substituted pyrrole- and furan-3-carbaldehydes (**4** and **5**) from 5-alkynyl-1,2,3-triazines **3** by adjusting the solvent systems.

Molecular editing has emerged as a modern synthetic strategy for the modification of heterocycles. This approach involves the insertion, deletion, or exchange of individual atoms, enabling ring contraction, expansion, and structural diversification. In this study, we explored the possibility of disrupting a simple heterocycle under accessible conditions, resulting in ring opening to produce a solvent-controlled intermediate that can flexibly transform into various types of heterocyclic rings. 1,2,3-Triazines have proven to be valuable heterocycles in annulation reactions owing to their versatile reactivity, which can be broadly categorized into two main paradigms: (1) pericyclic annulative processes and (2) regioselective ring opening via nucleophilic addition. Boger and co-workers demonstrated the cycloaddition reactions of 1,2,3-triazines with various amine-related dienophiles and provided new insights into the synthesis of *N*-containing heterocycles.^13^ In contrast, only a few syntheses have been reported within the second paradigm. Our previous work showed that 1,2,3-triazine could serve as a stable alternative to unmanageable malondialdehyde in the synthesis of β-aminoacrylonitriles using secondary amines.^14^ The reaction proceeded via the addition of amines to 1,2,3-triazine followed by *in situ* loss of nitrogen and imine oxidation to produce β-aminoacrylonitrile. In this approach, the ring opening of **3** with an appropriate nucleophile initially results in a reactive intermediate that is primed for subsequent cyclization. Notably, a diverse array of intermediates can be generated by employing different nucleophilic reagents (such as H_2_O, primary amines, and secondary amines), providing a versatile platform for the flexible synthesis of various heterocycles. On the other hand, 5-alkynyl-1,2,3-triazines **3** are more stable than 1,3-enynes and can be readily prepared on a large scale from commercially available terminal alkynes and 5-bromo-1,2,3-triazine **1** via the Sonogashira coupling reaction (Table S1). Recently, we demonstrated the AgNO_3_-mediated annulation reaction of 5-alkynyl-1,2,3-triazines with primary amines to construct a variety of 1,5-substituted pyrrole-3-carbaldehydes.^15^ In this target reaction, we hypothesize that the nucleophilic attack of a secondary amine on **3** will lead to the ring opening of the 1,2,3-triazine molecules, resulting in the formation of the reactive intermediate **B**. This may be followed by silver-mediated cyclization and annulation. Finally, hydrolysis and elimination yielded substituted pyrrole-3-carbaldehyde **4**. In contrast, when water is used as a nucleophile in the solvent, the reaction is expected to produce the hydrated intermediate **C**, which is subsequently cyclized to form the furan-3-carbaldehyde **5** from **3** (Scheme 1).

**Scheme 1:**
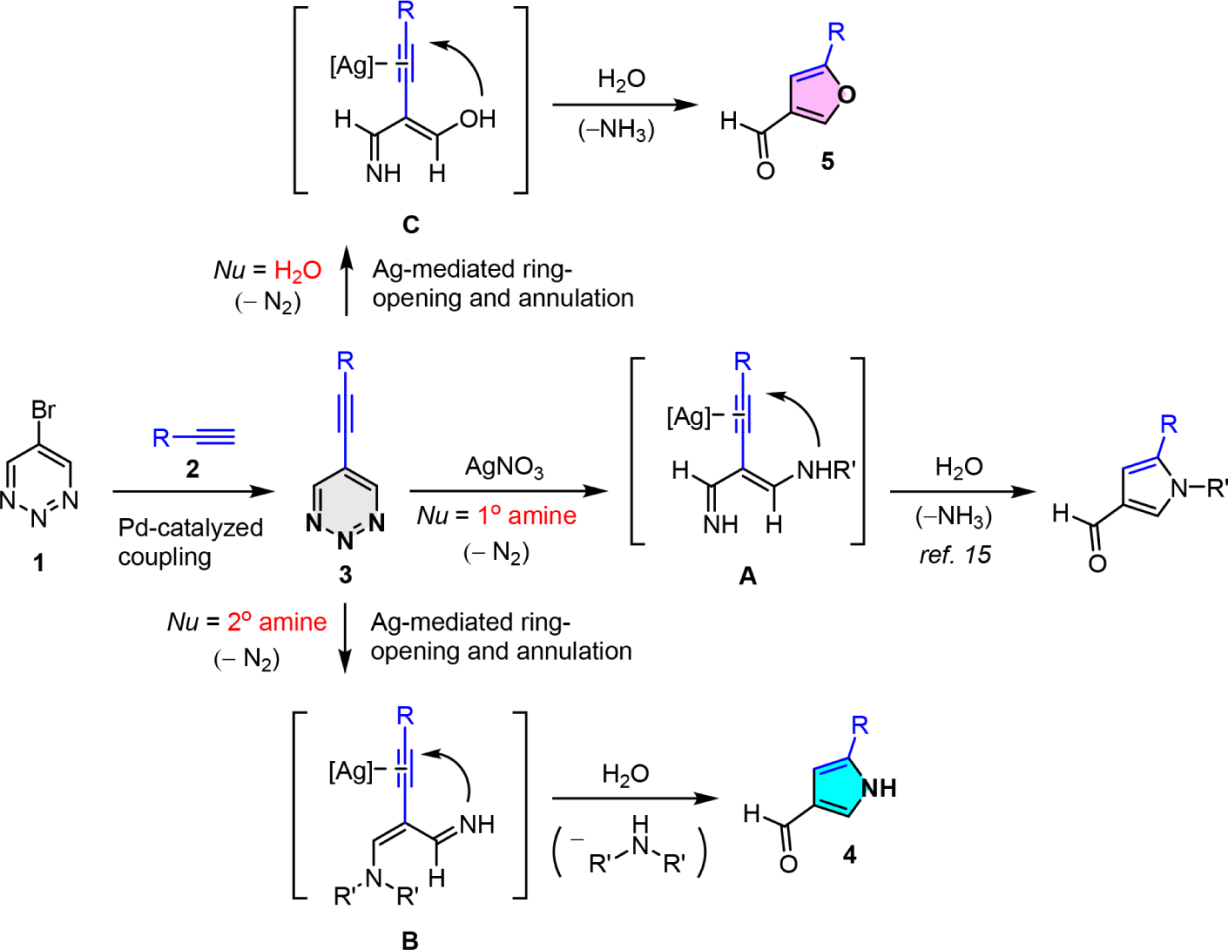
Approach Employed for the Divergent Synthesis of 5-Substituted Pyrrole-3-carbaldehydes (**4**) and Furan-3-carbaldehydes (**5**) from 5-Alkynyl-1,2,3-triazines (**3**) through Silver-Mediated Skeletal Remodeling Reaction

## Results and Discussion

To demonstrate the viability of the present strategy, we initially focused on converting 5-alkynyl-1,2,3-triazine **3** to pyrrole-3-carbaldehyde **4**. The cascade ring-opening and cyclization-annulation conditions were optimized by employing 5-phenylethynyl-1,2,3-triazine **3a** as a model substrate in the presence of silver salts in THF, as summarized in Table 1. After several attempts with various silver salts and secondary amines, we found that diethylamine accelerates the nucleophilic ring opening of triazine **3a** and promotes pyrrole formation using AgOAc. We initially used 50 mol % AgOAc in wet THF (approximately 500 ppm water content) at 50 °C under an air atmosphere. The desired pyrrole-3-carbaldehyde **4a** was obtained in 74% yield, whereas furan-3-carbaldehyde **5a** was obtained in 17% yield (entry 1). The appearance of furan **5a** in the reaction can be attributed to the water molecules in the solvent. Notably, the solvent plays a crucial role in this transformation, and almost exclusive formation of a considerable amount of **4a** (50% yield) was observed when wet THF was replaced with anhydrous THF (entry 2). On the other hand, a disordered result was observed in the absence of diethylamine, indicating that the use of diethylamine as a nucleophile is essential for this ring-opening process (entry 3). Therefore, we envision that increasing the water content in the reaction solvent may facilitate the conversion of **3a** to furan **5a**. Changing the solvent from wet THF to 10% aqueous THF resulted in an increased formation of **5a** when AgOAc was used, while the 32% yield of **5a** was lower than that expected (entry 4). These results prompted us to investigate the use of silver salts to favor the formation of compound **5a**. Among the different silver salts screened in the experiment, AgNO_3_ and AgClO_4_ exhibited superior results (entries 9 and 10), while AgOTf, AgF, and AgBF_4_ produced **5a**, and the corresponding yields were slightly lower (62–70%) (entries 5–7). In contrast, the reactions using AgOAc and AgSbF_6_ were less effective, yielding **5a** in 32% and 45% yield, respectively (entries 4 and 8). Notably, the reactions also proceeded effectively at an elevated temperature (80 °C) in 10% aqueous THF with AgNO_3_ and AgClO_4_, resulting in the formation of **5a** in 85% and 73% yields, respectively (entries 11–12). However, a lower yield of **5a** was observed when 25 mol % AgNO_3_ was used (entry 13). These results indicate that the nature of the anion plays a critical role in the distinct formation of pyrrole and furan.^16^

**Table 1:**
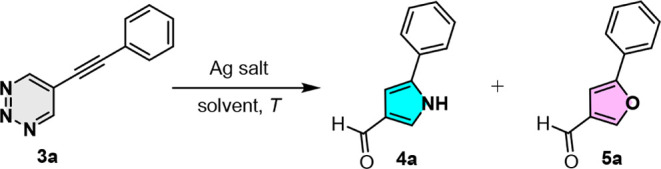
Screening of Solvent and Silver Salts for Optimizing Silver-Mediated Selective Annulation Reactions^a^

Entry	Silver salt	Solvent	*T* (°C)	Time (h)	Product (yield, %)^b^
1	AgOAc	Wet THF	50	15	**4a** (74%); **5a** (17%)
2	AgOAc	Anhydrous THF	50	48	**4a** (50%); **5a** (0%)
3^c^	AgOAc	Anhydrous THF	50	48	**4a** (0%); **5a** (0%)
4^c^	AgOAc	THF:H_2_O (9:1)	50	4	**4a** (14%); **5a** (32%)
5^c^	AgOTf	THF:H_2_O (9:1)	50	4	**4a** (4%); **5a** (68%)
6^c^	AgF	THF:H_2_O (9:1)	50	4	**4a** (0%); **5a** (70%)
7^c^	AgBF_4_	THF:H_2_O (9:1)	50	4	**4a** (4%); **5a** (62%)
8^c^	AgSbF_6_	THF:H_2_O (9:1)	50	4	**4a** (4%); **5a** (45%)
9^c^	AgNO_3_	THF:H_2_O (9:1)	50	4	**4a** (6%); **5a** (78%)
10^c^	AgClO_4_	THF:H_2_O (9:1)	50	4	**4a** (0%); **5a** (79%)
11^c^	AgNO_3_	THF:H_2_O (9:1)	80	4	**4a** (13%); **5a** (85%)
12^c^	AgClO_4_	THF:H_2_O (9:1)	80	4	**4a** (11%); **5a** (73%)
13^c^	AgNO_3_ ^d^	THF:H_2_O (9:1)	50	4	**4a** (5%); **5a** (38%)

a Reaction reagents and conditions: 5-phenylethynyl-1,2,3-triazine **3a** (0.5 mmol), diethylamine (0.5 mmol), and silver salt (0.25 mmol) in solvent (5 mL) under an air atmosphere.

b Isolated yield.

c Without using diethylamine.

d AgNO_3_ (0.125 mmol).

Based on the above results, we chose AgOAc in wet THF in the presence of diethylamine at 50 °C as the optimal conditions (condition A) to investigate the substrate scope in the formation of pyrrole. As shown in Table 2, the reaction exhibited excellent functional group tolerance across a wide range of substituents. The 5-arylethynyl-1,2,3-triazines (**3a**–**3h**), possessing different substituted groups with various substitution patterns on the phenyl ring, yielded the corresponding products (**4a**–**4h**) in 54–74% yields. Furthermore, the reaction of alkyl-bearing alkynyl triazines proceeded efficiently with the current protocol, and the corresponding 5-alkyl pyrrole-3-carbaldehydes (**4r** and **4s**) were obtained in 73% and 69% yields, respectively. Notably, various substituted alkyl groups, possessing valuable functionalities such as *O*-protected (**4i**–**4m**), *N*-protected (**4n**–**4p**), chloro (**4v**), cyano (**4w**), ester (**4x**), and acetate (**4y**, **4z,** and **4a’**) groups, were well tolerated in this approach. We examined the effects of sterically hindered alkyl moieties in alkynyl-1,2,3-triazine substrates (**3q** and **3t**), and the corresponding pyrrole-3-carbaldehyde **4t** was obtained in 70% yield from 5-[(*tert*-butyl)­ethynyl]-1,2,3-triazine **3t**, while the desired product **4q** could not be obtained from **3q** due to an unexpected desilylation reaction. The incorporation of a 1,3-enyne group onto the 1,2,3-triazine ring as a substrate (**3u**) is particularly noteworthy because it enables the efficient synthesis of 5-vinyl pyrrole-3-carbaldehyde **4u** (in 72% yield), which is not easily synthesized via traditional methods.

**Table 2:**
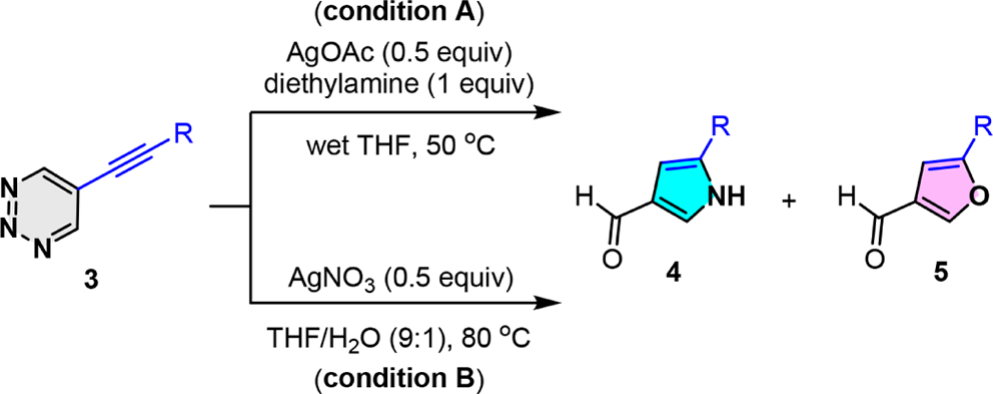

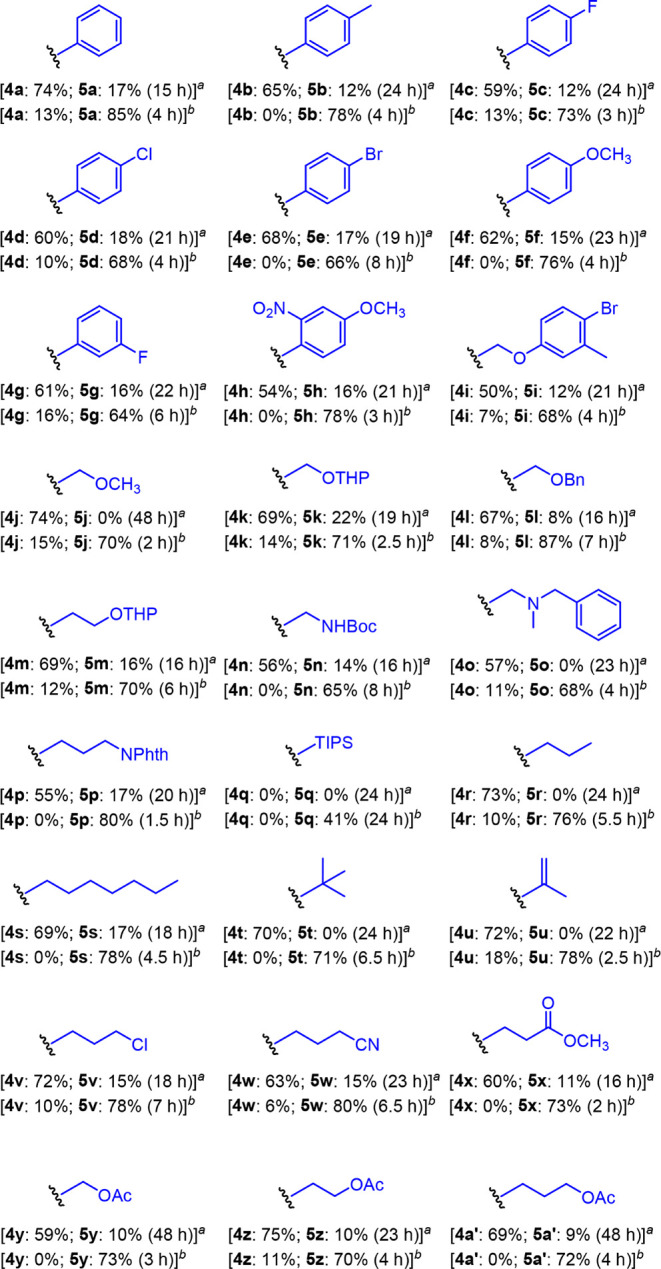
Substrate Scope of Silver-Mediated Annulation Reaction of Various 5-Alkynyl-1,2,3-Triazines **3** Using Reaction Conditions A and B^a^ ^b^ ^c^

a Reaction reagents and condition A: 5-alkynyl-1,2,3-triazine **3** (0.5 mmol), diethylamine (0.5 mmol), and AgOAc (0.25 mmol) in wet THF (5 mL) at 50 °C under an air atmosphere.

b Reaction reagents and condition B: 5-alkynyl-1,2,3-triazine **3** (0.5 mmol) and AgNO_3_ (0.25 mmol) in 10% aqueous THF (5 mL) at 80 °C under an air atmosphere.

c All yields were determined by column chromatography purification.

We then focused on applying our silver-mediated 5-alkynyl-1,2,3-triazine remodeling strategy to access furan-3-carbaldehydes **5**. The substrate scope for the formation of furan products was investigated under the optimized reaction conditions (condition B). As shown in Table 2, the reaction proceeded successfully with a series of 5-alkynyl-1,2,3-triazines (**3a**–**3z** and **3a’**) and generated the desired furan products (**5a**–**5z** and **5a’**) in moderate to good yields. For example, the reaction proceeded well in the case of 5-[(triisopropylsilyl)­ethynyl]-1,2,3-triazine **3q**, providing the desired furan product **5q** in a satisfactory yield. Interestingly, various functional groups that can be further modified, including phthalimide (**5p**), vinyl (**5u**), cyano (**5w**), methyl ester (**5x**), and acetate (**5y**, **5z,** and **5a’**), coexisted efficiently in this AgNO_3_-mediated reaction in 10% aqueous THF, resulting in the production of the corresponding furan-3-carbaldehydes. This result illustrates the excellent functional group tolerance of the reaction.

The series of reaction scope experiments presented above illustrate the skeletal remodeling reaction of 5-alkynyl-1,2,3-triazines, enabling the switchable construction of pyrrole and furan rings. To gain further insight into the corresponding reaction mechanism, several control experiments were conducted under standard conditions, as shown in Table 1. To prevent the production of furans via nucleophilic addition, the reaction was performed in anhydrous THF to produce the single pyrrole product **4a** with a longer reaction time (48 h) and a significantly lower yield (50%) than the standard reaction conditions (entry 2). Next, we carried out the reaction in the absence of diethylamine, which failed to yield any of the desired products (entry 3). These results suggest that the solvent significantly influences both the reactivity and the selectivity of the skeletal remodeling reaction. In particular, the presence of water can facilitate the nucleophilic ring opening of triazine and the hydrolysis of the imine intermediate under the selected reaction conditions, thereby further enhancing the conversion process.

A possible mechanism for the present silver-mediated skeletal remodeling reaction of 5-alkynyl-1,2,3-triazines for the solvent-controlled divergent synthesis of furans and pyrroles is proposed in Scheme 2. This transformation involves two key steps: first, the nucleophilic addition of diethylamine and water to the 5-alkynyl-1,2,3-triazine **3**, followed by coordination of the π-bond of the alkyne group to the silver­(I) species, which generates the intermediate adducts **I** and **I’**. The subsequent tandem intramolecular heteroannulation of intermediates **I** and **I’** results in the formation of pyrroles and furans, respectively. Under reaction condition A, the imine intermediate **I** undergoes AgOAc-mediated intramolecular cyclization, followed by hydrolysis to form the intermediate **II’** and tautomerization, finally yielding the pyrrole product **4**. This reaction uses wet THF as a solvent to suppress the competitive formation of the imine intermediate **I’** from **3**. On the other hand, when the reaction is performed in 10% aqueous THF, **3** tends to form intermediate **I’** under reaction condition B. The AgNO_3_-mediated intramolecular cyclization of **I’** generates an imine furan intermediate **III’**, followed by hydrolysis to yield the furan-3-carbaldehydes **5** (path a). For the formation of small amounts of pyrrole, we speculate that the cyclization of the intermediate **I’** via C–N bond formation may occur in the subsequent step and then generate intermediate **II’**, followed by sequential tautomerization to produce the pyrrole-3-carbaldehydes **4** (path b). These results show that the selectivity can be effectively controlled by adjusting the silver-mediated conditions and solvent systems.

**Scheme 2:**
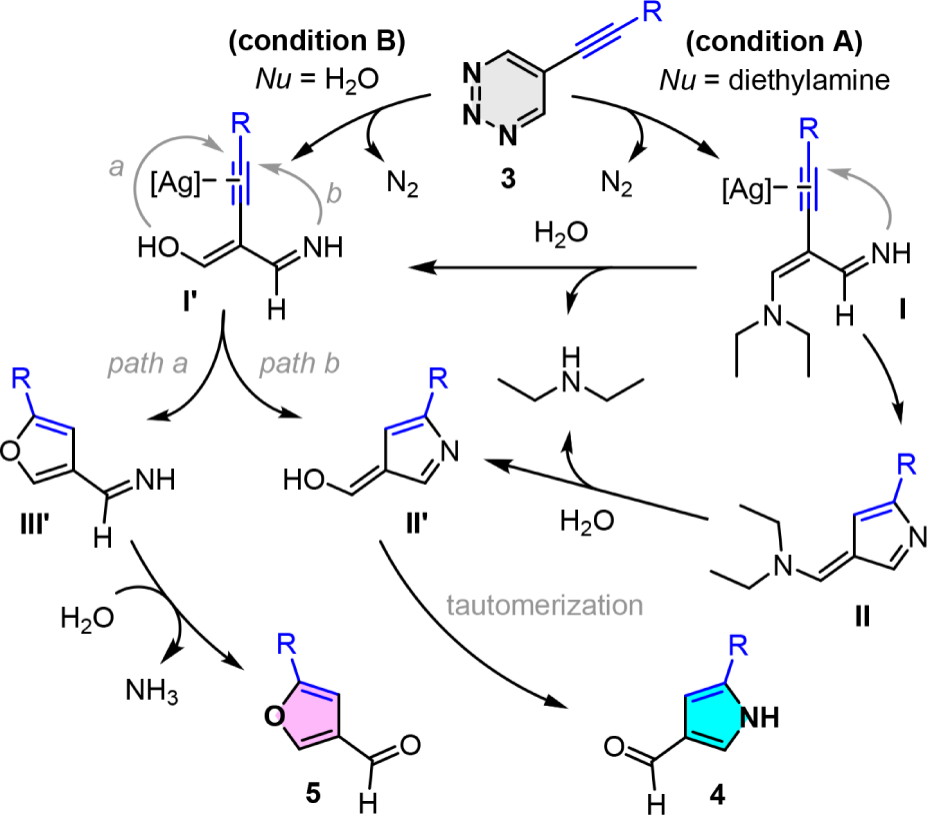
Possible Reaction Mechanism for the Solvent-Controlled Switchable Synthesis of Pyrroles and Furans

To avoid the tedious separation of the Sonogashira coupling products in the first step and improve the overall operational efficiency, we explored whether the first palladium-catalyzed cross-coupling step and the subsequent silver-mediated cyclization-annulation could be combined in a one-pot process. Encouragingly, the 5-bromo-1,2,3-triazine **1** could be consumed completely when the reaction was carried out in wet THF at room temperature for 1 h via a palladium-catalyzed coupling reaction, providing 5-phenylethynyl-1,2,3-triazine **3a**. The addition of AgOAc to the reaction mixture at an elevated temperature (50 °C) afforded pyrrole-3-carbaldehyde **4a** in 70% yield in a one-pot reaction. In the furan synthesis, the one-pot reaction also proceeded well, yielding the desired furan product **5** from **1** in an 81% yield (Scheme 3a). These results indicate that this one-pot protocol can be used to synthesize various pyrrole-3-carbaldehydes **4** and furan-3-carbaldehydes **5** from 5-bromo-1,2,3-triazine **1**. The yield of the one-pot reaction is comparable to the overall yield of the two-step synthesis in the examined cases. Notably, this type of multicatalytic approach could be applied on a 10 mmol scale in a nonanhydrous solvent under an air atmosphere.

**Scheme 3:**
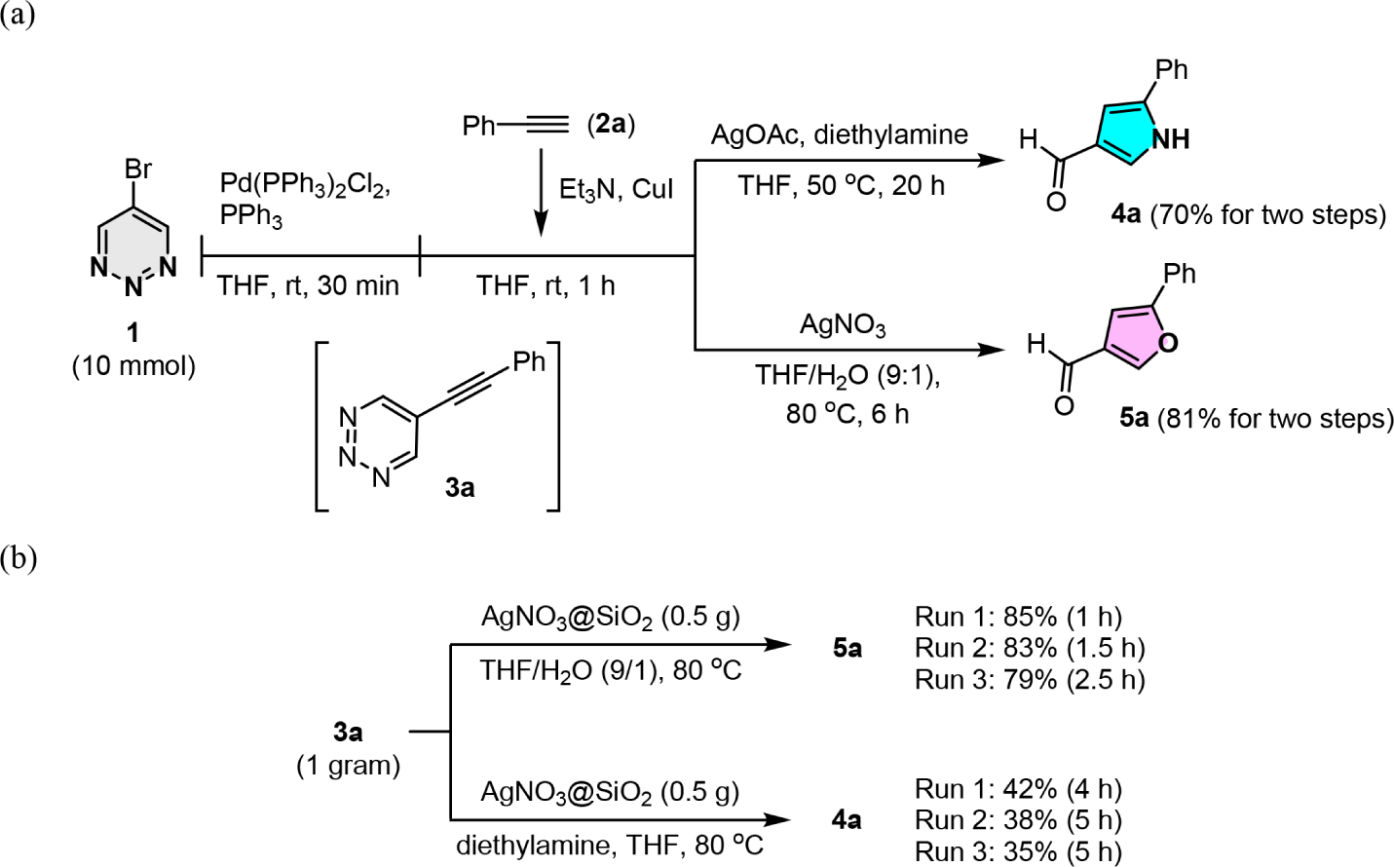
(a) One-Pot, Gram-Scale Switchable Synthesis of Pyrrole (**4a**) and Furan (**5a**) from 5-Bromo-1,2,3-triazine (**1**) and Phenylacetylene (**2a**). (b) Reaction Apparatus for Synthesis of Pyrrole (**4a**) and Furan (**5a**) from 5-Phenylethynyl-1,2,3-triazine (**3a**) in the Presence of AgNO_3_@SiO_2_

While this silver-mediated approach is a reliable method for the switchable construction of functionalized pyrroles and furans, the requirement for stoichiometric amounts (0.5 equiv) of silver promoters can pose challenges when scaling up the synthesis of these compounds. Silver nitrate (AgNO_3_) on silica gel is a well-established catalyst used in the synthesis of functionalized *trans*-cyclooctene derivatives.^17^ Based on the aforementioned success, we envisaged that a silica-gel-supported catalyst could be utilized for the divergent synthesis of pyrroles and furans. This method would enhance process efficiency and cost-effectiveness by facilitating product purification and the recovery and recycling of often-expensive metal catalysts. As shown in Scheme 3b, the reaction was performed using a 0.11 M solution of 5-phenylethynyl-1,2,3-triazine **3a** in 10% aqueous THF, along with 500 mg of 10 wt % AgNO_3_@SiO_2_.^18^ The AgNO_3_-supported silica gel could be recycled without a significant change in yield (82 ± 3%) in three consecutive reactions that each began with 1 g of **3a**. In addition, silica gel was shown to have a significant impact on reaction activity, as the reaction utilizing 0.5 equiv of AgNO_3_ as a catalyst resulted in an extended reaction time of 4 h. We hypothesized that silica gel possesses a good adsorption capacity, which may indirectly enhance the catalytic reaction by adsorbing reactants in close proximity to the supported metal catalysts. In contrast, pyrrole **4a** can also be synthesized using this protocol, but the yield is lower than expected.

## Conclusion

In conclusion, we have developed silver-mediated skeletal remodeling reactions of 5-alkynyl-1,2,3-triazines, providing an efficient method for the divergent synthesis of furans and pyrroles. The chemoselectivity of this transformation is controlled by the choice of silver salts and solvent systems. Notably, this protocol enables access to 5-substituted pyrrole- and furan-3-carbaldehydes from the same starting materials through a one-pot tandem process. Additionally, AgNO_3_-supported silica gel can be used and recycled in these reactions, thereby reducing the amount of silver salt required.

## Experimental Section

### General Synthetic Procedure for the AgOAc-Mediated Annulation of 5-Alkynyl-1,2,3-triazines **(3)** with Diethylamine in Wet THF (Condition A)

To a solution of 5-alkynyl-1,2,3-triazine **3** (0.5 mmol, 1 equiv) in wet THF (5 mL) were added AgOAc (0.25 mmol, 42 mg, 0.5 equiv) and diethylamine (0.5 mmol, 53 μL, 1 equiv). The reaction mixture was heated at 50 °C under an air atmosphere. After the complete consumption of the reactants in 16–48 h, as shown by TLC analysis, the reaction mixture was concentrated by a rotary evaporator. The crude products were purified by column chromatography on silica gel to afford the corresponding 5-substituted 1*H*-pyrrole-3-carboxaldehyde **4** and 5-substituted furan-3-carboxaldehyde **5**.

### General Synthetic Procedure for the AgNO_3_-Mediated Annulation of 5-Alkynyl-1,2,3-triazines **(3)** in Aqueous THF (THF/H_2_O: 9:1, V/V) (Condition B)

To a solution of 5-alkynyl-1,2,3-triazine **3** (0.5 mmol, 1 equiv) in a mixture of THF (4.5 mL) and H_2_O (0.5 mL) was added AgNO_3_ (0.25 mmol, 43 mg, 0.5 equiv). The reaction mixture was heated at 80 °C under an air atmosphere. After the complete consumption of the reactants in 2–24 h, as shown by TLC analysis, the reaction mixture was concentrated by a rotary evaporator. The crude products were purified by column chromatography on silica gel to afford the corresponding 5-substituted 1*H*-pyrrole-3-carboxaldehyde **4** and 5-substituted furan-3-carboxaldehyde **5**.

### General Synthetic Procedure for One-Pot, Gram-Scale Synthesis of 5-Phenyl-1*H*-pyrrole-3-carboxaldehyde **(4a)** from 5-Bromo-1,2,3-triazine and Phenylacetylene **(2a)**

Pd­(PPh_3_)_2_Cl_2_ (4 mol %, 280 mg, 0.04 equiv) and PPh_3_ (2.5 mol %, 70 mg, 0.025 equiv) were added to a solution of 5-bromo-1,2,3-triazine **1** (10 mmol, 1.6 g, 1 equiv) in wet THF (50 mL). After degassing for 30 min by bubbling with nitrogen, CuI (4 mol %, 77 mg, 0.04 equiv), triethylamine (36 mmol, 5 mL, 3.6 equiv), and phenylacetylene **2a** (15 mmol, 1.53 g, 1.5 equiv) were added under a nitrogen atmosphere. After stirring at room temperature for 1 h, diethylamine (10 mmol, 1.01 mL, 1 equiv) and AgOAc (5 mmol, 835 mg, 0.5 equiv) were added. The reaction mixture was heated at 50 °C under an air atmosphere. After the complete consumption of the reactants in 20 h, as shown by TLC analysis, the reaction mixture was filtered through a Celite pad; the filtrate was washed with brine (100 mL), and then, the aqueous layer was extracted with CH_2_Cl_2_ (3 × 100 mL). The combined organic extracts were dried over anhydrous MgSO_4_, filtered, and concentrated with a rotary evaporator. The crude product was purified by column chromatography on silica gel to afford 5-phenyl-1*H*-pyrrole-3-carboxaldehyde **4a** (1.20 g, 70% yield).

### General Synthetic Procedure for One-Pot, Gram-Scale Synthesis of 5-Phenyl-1*H*-furan-3-carboxaldehyde **(5a)** from 5-Bromo-1,2,3-triazine and Phenylacetylene **(2a)**

Pd­(PPh_3_)_2_Cl_2_ (4 mol %, 280 mg, 0.04 equiv) and PPh_3_ (2.5 mol %, 70 mg, 0.025 equiv) were added to a solution of 5-bromo-1,2,3-triazine **1** (10 mmol, 1.6 g, 1 equiv) in wet THF (45 mL). After degassing for 30 min by bubbling with nitrogen, CuI (4 mol %, 77 mg, 0.04 equiv), triethylamine (36 mmol, 5 mL, 3.6 equiv), and phenylacetylene **2a** (15 mmol, 1.53 g, 1.5 equiv) were added under a nitrogen atmosphere. After the mixture was stirred at room temperature for 1 h, water (5 mL) and AgNO_3_ (5 mmol, 850 mg, 0.5 equiv) were added. The reaction mixture was heated at 80 °C under an air atmosphere. After complete consumption of the reactants in 6 h, as shown by TLC analysis, the reaction mixture was filtered through a Celite pad; the filtrate was washed with brine (100 mL), and then, the aqueous layer was extracted with CH_2_Cl_2_ (3 × 100 mL). The combined organic extracts were dried over anhydrous MgSO_4_, filtered, and concentrated by a rotary evaporator. The crude product was purified by column chromatography on silica gel to afford 5-phenyl-1*H*-furan-3-carboxaldehyde **5a** (1.39 g, 81% yield).

### Typical Procedure for the AgNO_3_@SiO_2_-Mediated Skeletal Remodeling Approach Used to Synthesize 5-Phenyl-1*H*-pyrrole-3-carboxaldehyde **(4a)** and 5-Phenyl-1*H*-furan-3-carboxaldehyde **(5a)** from 5-Phenylethynyl-1,2,3-triazine **(3a)**, Respectively

#### Pyrrole Synthesis

To a solution of 5-phenylethynyl-1,2,3-triazine **3a** (1 g, 5.5 mmol, 1 equiv) in a mixture of wet THF (25 mL) were added 10 wt % AgNO_3_@SiO_2_ (500 mg, from Merck Chemicals Ltd.) and diethylamine (5.5 mmol, 0.57 mL, 1 equiv). The reaction mixture was heated at 80 °C under an air atmosphere. After complete consumption of the reactants in 4–5 h, as shown by TLC analysis, the reaction mixture was poured onto filter paper. The resulting AgNO_3_@SiO_2_ powder was washed with THF and then dried under a vacuum at 50 °C for the next run. In each reaction run, the filtrate was extracted with EtOAc, and the collected organic layers were washed with brine, dried over MgSO_4_, and concentrated. The residue was purified by column chromatography on silica gel to determine the yield of 5-phenyl-1*H*-pyrrole-3-carboxaldehyde **4a**.

#### Furan Synthesis

To a solution of 5-phenylethynyl-1,2,3-triazine **3a** (1 g, 5.5 mmol, 1 equiv) in a mixture of THF (25 mL) and H_2_O (25 mL) was added 10 wt % AgNO_3_@SiO_2_ (500 mg, from Merck Chemicals Ltd.). The reaction mixture was heated at 80 °C under an air atmosphere. After complete consumption of the reactants in 1–2.5 h, as shown by TLC analysis, the reaction mixture was poured onto filter paper. The resulting AgNO_3_@SiO_2_ powder was washed with THF and then dried under vacuum at 50 °C for the next run. In each reaction run, the filtrate was extracted with EtOAc, and the collected organic layers were washed with brine, dried over MgSO_4_, and concentrated. The residue was purified by column chromatography on silica gel to determine the yield of 5-phenyl-1*H*-furan-3-carboxaldehyde **5a**.

##### 5-Phenyl-1*H*-pyrrole-3-carboxaldehyde (**4a**) and 5-Phenyl Furan-3-carboxaldehyde (**5a**)

Compounds **4a** and **5a** were synthesized from 5-(phenylethynyl)-1,2,3-triazine **3a** (0.5 mmol, 91 mg) under reaction conditions A and B, respectively. The crude product was purified by column chromatography on silica gel (CH_2_Cl_2_/toluene/hexane = 2:2:3 to EtOAc/toluene/hexane = 2:2:3) to afford the desired pyrrole **4a** (74% for condition A; 13% for condition B) and the desired furan **5a** (17% for condition A; 85% for condition B).

Compound **4a**: brown solid; C_11_H_9_NO; mp 139–141 °C; TLC (EtOAc/hexane = 2:3) *R*_*f*_ = 0.33; IR ν_max_ (neat) 3284, 1640, 1608, 1565, 1514, 1492, 1435, 1415, 1192, 1120, 806, 756, 723, 690, 655, 610 cm^–1^; ^1^H NMR (500 MHz, CDCl_3_) δ 9.79 (s, 1H), 9.48 (br s, NH, 1H), 7.51–7.47 (m, 3H), 7.38 (dd, *J =* 7.8 Hz, 7.8 Hz, 2H), 7.27 (t, *J* = 7.5 Hz, 1H), 6.91 (s, 1H); ^13^C­{^1^H} NMR (125 MHz, CDCl_3_) δ 186.0, 134.8, 131.2, 129.1, 128.14, 128.10, 128.0, 127.5, 124.4, 103.9; HRMS (EI) *m*/*z*: [M]^+^ Calcd for C_11_H_9_NO 171.0684; Found 171.0679.

Compound **5a**: yellow solid; C_11_H_8_O_2_; mp 86–88 °C; TLC (EtOAc/hexane = 2:3) *R*_*f*_ = 0.66; IR ν_max_ (neat) 2970, 1738, 1659, 1538, 1383, 1230, 1217, 1182, 1137, 906, 827, 753, 688, 663, 597 cm^–1^; ^1^H NMR (500 MHz, CDCl_3_) δ 9.93 (s, 1H), 8.06 (s, 1H), 7.67 (d, *J* = 7.5 Hz, 2H), 7.40 (dd, *J* = 7.8 Hz, 7.8 Hz, 2H), 7.31 (t, *J* = 7.4 Hz, 1H), 6.98 (s, 1H); ^13^C­{^1^H} NMR (125 MHz, CDCl_3_) δ 184.4, 156.5, 150.6, 130.3, 129.3, 128.8, 128.6, 124.3, 101.2; HRMS (EI) *m*/*z*: [M]^+^ Calcd for C_11_H_8_O_2_ 172.0524; Found 172.0523.

##### 5-(*p*-Tolyl)-1*H*-pyrrole-3-carboxaldehyde (**4b**) and 5-(*p*-Tolyl) Furan-3-carboxaldehyde (**5b**)

Compounds **4b** and **5b** were synthesized from 5-(*p*-tolylethynyl)-1,2,3-triazine **3b** (0.5 mmol, 98 mg) under reaction conditions A and B, respectively. The crude product was purified by column chromatography on silica gel (CH_2_Cl_2_/toluene/hexane = 2:2:3) to afford the desired pyrrole **4b** (65% for condition A; 0% for condition B) and the desired furan **5b** (12% for condition A; 78% for condition B).

Compound **4b**: brown syrup; C_12_H_11_NO; TLC (EtOAc/hexane = 2:3) *R*_*f*_ = 0.28; IR ν_max_ (neat) 3199, 3005, 1640, 1521, 1496, 1455, 1420, 1333, 1275, 1260, 1189, 1141, 1044, 925, 824, 806, 750, 660, 612 cm^–1^; ^1^H NMR (400 MHz, CDCl_3_) δ 9.79 (s, 1H), 9.39 (br s, NH, 1H), 7.45 (s, 1H), 7.39 (d, *J* = 7.2 Hz, 2H), 7.18 (d, *J* = 7.6 Hz, 2H), 6.86 (s, 1H), 2.34 (s, 3H); ^13^C­{^1^H} NMR (100 MHz, CDCl_3_) δ 185.93, 185.88, 137.4, 135.0, 129.7, 128.5, 128.0, 124.3, 103.4, 21.1; HRMS (EI) *m*/*z*: [M]^+^ Calcd for C_12_H_11_NO 185.0841; Found 185.0836.

Compound **5b**: yellow solid; C_12_H_10_O_2_; mp 100–103 °C; TLC (EtOAc/hexane = 2:3) *R*_*f*_ = 0.63; IR ν_max_ (neat) 3284, 1640, 1608, 1515, 1454, 1435, 1415, 1192, 1120, 807, 756, 723, 690, 655, 614 cm^–1^; ^1^H NMR (500 MHz, CDCl_3_) δ 9.92 (s, 1H), 8.04 (d, *J* = 0.5 Hz, 1H), 7.55 (d, *J* = 8.5 Hz, 2H), 7.20 (d, *J* = 8.0 Hz, 2H), 6.92 (s, 1H), 2.36 (s, 3H); ^13^C­{^1^H} NMR (125 MHz, CDCl_3_) δ 184.4, 156.6, 150.3, 138.6, 130.3, 129.4, 126.6, 124.1, 100.4, 21.2; HRMS (EI) *m*/*z*: [M]^+^ Calcd for C_12_H_10_O_2_ 186.0681; Found 186.0679.

##### 5-(4-Fluorophenyl)-1*H*-pyrrole-3-carboxaldehyde (**4c**) and 5-(4-Fluorophenyl) Furan-3-carboxaldehyde (**5c**)

Compounds **4c** and **5c** were synthesized from 5-((4-fluorophenyl)­ethynyl)-1,2,3-triazine **3c** (0.5 mmol, 100 mg) under reaction conditions A and B, respectively. The crude product was purified by column chromatography on silica gel (CH_2_Cl_2_/toluene/hexane = 2:2:3 to EtOAc/toluene/hexane = 2:2:3) to afford the desired pyrrole **4c** (59% for condition A; 13% for condition B) and the desired furan **5c** (12% for condition A; 73% for condition B).

Compound **4c**: yellow solid; C_11_H_8_FNO; mp 138–141 °C; TLC (EtOAc/hexane = 2:3) *R*_*f*_ = 0.23; IR ν_max_ (neat) 3140, 1642, 1573, 1496, 1452, 1336, 1187, 1164, 1143, 925, 832, 800, 768, 652, 605 cm^–1^; ^1^H NMR (500 MHz, CDCl_3_) δ 9.79 (s, 1H), 9.32 (br s, NH, 1H), 7.48–7.45 (m, 3H), 7.10–7.06 (m, 2H), 6.84–6.83 (m, 1H); ^13^C­{^1^H} NMR (125 MHz, CDCl_3_) δ 185.9, 162.2 (*J*_C–F_ = 246.3 Hz), 134.0, 128.0 (*J*_C–F_ = 10.0 Hz, 2 ×), 127.6 (*J*_C–F_ = 3.8 Hz), 126.25, 126.18, 116.1 (*J*_C–F_ = 22.5 Hz, 2 ×), 103.9; ^19^F­{^1^H} NMR (470 MHz, CDCl_3_) δ −113.9; HRMS (EI) *m*/*z*: [M]^+^ Calcd for C_11_H_8_FNO 189.0590; Found 189.0594.

Compound **5c**: white solid; C_11_H_7_FO_2_; mp 115–117 °C; TLC (EtOAc/hexane = 2:3) *R*_*f*_ = 0.59; IR ν_max_ (neat) 2970, 1738, 1659, 1581, 1497, 1365, 1229, 1217, 1177, 1135, 1095, 968, 838, 808, 760, 597 cm^–1^; ^1^H NMR (500 MHz, CDCl_3_) δ 9.93 (s, 1H), 8.06 (d, *J* = 0.5 Hz, 1H), 7.67–7.63 (m, 2H), 7.12–7.08 (m, 2H), 6.92 (s, 1H); ^13^C­{^1^H} NMR (125 MHz, CDCl_3_) δ 184.3, 162.7 (*J*_C–F_ = 250.0 Hz), 155.5, 150.5, 130.3, 126.1 (*J*_C–F_ = 8.8 Hz), 125.6, 115.8 (*J*_C–F_ = 22.5 Hz), 100.9; ^19^F­{^1^H} NMR (470 MHz, CDCl_3_) δ −112.1; HRMS (EI) *m*/*z*: [M]^+^ Calcd for C_11_H_7_FO_2_ 190.0430; Found 190.0428.

##### 5-(4-Chlorophenyl)-1*H*-pyrrole-3-carboxaldehyde (**4d**) and 5-(4-Chlorophenyl) Furan-3-carboxaldehyde (**5d**)

Compounds **4d** and **5d** were synthesized from 5-((4-chlorophenyl)­ethynyl)-1,2,3-triazine **3d** (0.5 mmol, 108 mg) under reaction conditions A and B, respectively. The crude product was purified by column chromatography on silica gel (CH_2_Cl_2_/toluene/hexane = 2:2:3 to EtOAc/toluene/hexane = 2:2:3) to afford the desired pyrrole **4d** (60% for condition A; 10% for condition B) and the desired furan **5d** (18% for condition A; 68% for condition B).

Compound **4d**: brown solid; C_11_H_8_ClNO; mp 178–180 °C; TLC (EtOAc/hexane = 2:3) *R*_*f*_ = 0.18; IR ν_max_ (neat) 3287, 1647, 1487, 1182, 1138, 1096, 823, 798, 769, 724, 608 cm^–1^; ^1^H NMR (500 MHz, CDCl_3_) δ 9.81 (s, 1H), 8.99 (br s, NH, 1H), 7.48 (s, 1H), 7.41 (d, *J* = 8.5 Hz, 2H), 7.36 (d, *J* = 8.5 Hz, 2H), 6.90 (s, 1H); ^13^C­{^1^H} NMR (125 MHz, CDCl_3_) δ 185.6, 133.6, 133.4, 129.8, 129.3, 128.3, 127.7, 125.6, 104.6; HRMS (EI) *m*/*z*: [M]^+^ Calcd for C_11_H_8_ClNO 205.0294; Found 205.0297.

Compound **5d**: yellowish solid; C_11_H_7_ClO_2_; mp 120–122 °C; TLC (EtOAc/hexane = 2:3) *R*_*f*_ = 0.59; IR ν_max_ (neat) 3114, 1673, 1533, 1482, 1416, 1288, 1189, 1176, 1143, 1093, 1014, 908, 822, 768, 752, 597 cm^–1^; ^1^H NMR (500 MHz, CDCl_3_) δ 9.93 (s, 1H), 8.07 (s, 1H), 7.60 (d, *J* = 8.5 Hz, 2H), 7.38 (d, *J* = 8.5 Hz, 2H), 6.98 (s, 1H); ^13^C­{^1^H} NMR (100 MHz, CDCl_3_) δ 184.2, 155.3, 150.6, 134.3, 130.3, 129.0, 127.7, 125.4, 101.7; HRMS (EI) *m*/*z*: [M]^+^ Calcd for C_11_H_7_ClO_2_ 206.0135; Found 206.0137.

##### 5-(4-Bromophenyl)-1*H*-pyrrole-3-carboxaldehyde (**4e**) and 5-(4-Bromophenyl) Furan-3-carboxaldehyde (**5e**)

Compounds **4e** and **5e** were synthesized from 5-((4-bromophenyl)­ethynyl)-1,2,3-triazine **3e** (0.5 mmol, 130 mg) under reaction conditions A and B, respectively. The crude product was purified by column chromatography on silica gel (CH_2_Cl_2_/toluene/hexane = 2:2:3) to afford desired pyrrole **4e** (68% for condition A; 0% for condition B) and desired furan **5e** (17% for condition A; 66% for condition B).

Compound **4e**: yellow solid; C_11_H_8_BrNO; mp 190–193 °C; TLC (EtOAc/hexane = 2:3) *R*_*f*_ = 0.25; IR ν_max_ (neat) 3294, 1645, 1561, 1483, 1447, 1422, 1392, 1183, 1137, 1074, 1007, 924, 819, 790, 768, 705, 646 cm^–1^; ^1^H NMR (500 MHz, CDCl_3_) δ 9.81 (s, 1H), 8.95 (br s, NH, 1H), 7.53–7.50 (m, 2H), 7.48 (dd, *J* = 3.1, 1.6 Hz, 1H), 7.36–7.33 (m, 2H), 6.91 (dd, *J* = 2.6, 1.6 Hz, 1H); ^13^C­{^1^H} NMR (125 MHz, CDCl_3_) δ 185.5, 133.5, 132.3, 130.2, 128.4, 127.7, 125.8, 121.4, 104.7; HRMS (EI) *m*/*z*: [M]^+^ Calcd for C_11_H_8_BrNO 248.9789; Found 248.9788.

Compound **5e**: brown solid; C_11_H_7_BrO_2_; mp 119–121 °C; TLC (EtOAc/hexane = 2:3) *R*_*f*_ = 0.53; IR ν_max_ (neat) 3114, 2859, 1673, 1534, 1479, 1414, 1288, 1177, 1072, 1018, 907, 840, 823, 774, 598 cm^–1^; ^1^H NMR (500 MHz, CD_3_OD) δ 9.92 (s, 1H), 8.42 (d, *J* = 0.8 Hz, 1H), 7.68 (dd, *J* = 6.7, 2.0 Hz, 2H), 7.60 (dd, *J* = 6.8, 2.1 Hz, 2H), 7.15 (s, 1H); ^13^C­{^1^H} NMR (125 MHz, CDCl_3_) δ 184.2, 155.4, 150.6, 132.0, 130.3, 128.2, 125.7, 122.6, 101.8; HRMS (EI) *m*/*z*: [M]^+^ Calcd for C_11_H_7_BrO_2_ 249.9629; Found 249.9621.

##### 5-(4-Methoxyphenyl)-1*H*-pyrrole-3-carboxaldehyde (**4f**) and 5-(4-Methoxyphenyl) Furan-3-carboxaldehyde (**5f**)

Compounds **4f** and **5f** were synthesized from 5-((4-methoxyphenyl)­ethynyl)-1,2,3-triazine **3f** (0.5 mmol, 106 mg) under reaction conditions A and B, respectively. The crude product was purified by column chromatography on silica gel (CH_2_Cl_2_/toluene/hexane = 2:2:3) to afford the desired pyrrole **4f** (62% for condition A; 0% for condition B) and the desired furan **5f** (15% for condition A; 76% for condition B).

Compound **4f**: brown solid; C_12_H_11_NO_2_; mp 191–193 °C; TLC (EtOAc/hexane = 2:3) *R*_*f*_ = 0.19; IR ν_max_ (neat) 3019, 1645, 1499, 1442, 1291, 1247, 1178, 1139, 1022, 802, 761, 610 cm^–1^; ^1^H NMR (500 MHz, CDCl_3_) δ 9.80 (s, 1H), 8.82 (br s, NH, 1H), 7.44–7.43 (m, 1H), 7.42–7.39 (m, 2H), 6.95–6.92 (m, 2H), 6.81–6.80 (m, 1H), 3.82 (s, 3H); ^13^C­{^1^H} NMR (125 MHz, CDCl_3_) δ 185.7, 159.2, 134.7, 128.2, 127.2, 125.8, 124.1, 114.6, 103.0, 55.4; HRMS (EI) *m*/*z*: [M]^+^ Calcd for C_12_H_11_NO_2_ 201.0790; Found 201.0789.

Compound **5f**: brown solid; C_12_H_10_O_3_; mp 100–103 °C; TLC (EtOAc/hexane = 2:3) *R*_*f*_ = 0.55; IR ν_max_ (neat) 3111, 1658, 1614, 1574, 1498, 1464, 1315, 1301, 1252, 1174, 1139, 1108, 1034, 1017, 1102, 908, 826 cm^–1^; ^1^H NMR (500 MHz, CDCl_3_) δ 9.91 (s, 1H), 8.02 (d, *J* = 1.0 Hz, 1H), 7.59 (d, *J* = 8.5 Hz, 2H), 6.92 (d, *J* = 9.0 Hz, 2H), 6.84 (s, 1H), 3.82 (s, 3H); ^13^C­{^1^H} NMR (125 MHz, CDCl_3_) δ 184.5, 159.9, 156.5, 150.2, 130.4, 125.7, 122.2, 114.2, 99.5, 55.3; HRMS (EI) *m*/*z*: [M]^+^ Calcd for C_12_H_10_O_3_ 202.0630; Found 202.0631.

##### 5-(3-Fluorophenyl)-1*H*-pyrrole-3-carboxaldehyde (**4g**) and 5-(3-Fluorophenyl) Furan-3-carboxaldehyde (**5g**)

Compounds **4g** and **5g** were synthesized from 5-((3-fluorophenyl)­ethynyl)-1,2,3-triazine **3g** (0.5 mmol, 100 mg) under reaction conditions A and B, respectively. The crude product was purified by column chromatography on silica gel (CH_2_Cl_2_/toluene/hexane = 2:2:3 to EtOAc/toluene/hexane = 2:2:3) to afford the desired pyrrole **4g** (61% for condition A; 16% for condition B) and the desired furan **5g** (16% for condition A; 64% for condition B).

Compound **4g**: brown solid; C_11_H_8_FNO; mp 154–156 °C; TLC (EtOAc/hexane = 2:3) *R*_*f*_ = 0.16; IR ν_max_ (neat) 3315, 2921, 1634, 1615, 1510, 1493, 1428, 1184, 1123, 958, 869, 848, 825, 782, 757, 713, 679, 607 cm^–1^; ^1^H NMR (500 MHz, CDCl_3_) δ 9.82 (s, 1H), 9.05 (br s, NH, 1H), 7.49 (dd, *J* = 1.5, 1.5 Hz, 1H), 7.38–7.33 (m, 1H), 7.26–7.25 (m, 1H), 7.18 (dt, *J* = 9.8, 2.3 Hz, 1H), 6.99–6.96 (m, 1H), 6.94 (dd, *J =* 7.5, 1.7 Hz, 1H); ^13^C­{^1^H} NMR (125 MHz, CDCl_3_) δ 185.7, 163.3 (*J*_C–F_ = 237.5 Hz), 152.4, 133.4 (*J*_C–F_ = 5.8 Hz), 130.8 (*J*_C–F_ = 8.5 Hz), 128.2, 127.8, 119.9 (*J*_C–F_ = 2.4 Hz), 114.3 (*J*_C–F_ = 21.1 Hz), 111.3 (*J*_C–F_ = 22.9 Hz), 105.00; ^19^F­{^1^H} NMR (470 MHz, CDCl_3_) δ −112.0; HRMS (EI) *m*/*z*: [M]^+^ Calcd for C_11_H_8_FNO 189.0590; Found 189.0587.

Compound **5g**: brown solid; C_11_H_7_FO_2_; mp 80–82 °C; TLC (EtOAc/hexane = 2:3) *R*_*f*_ = 0.51; IR ν_max_ (neat) 3116, 1676, 1614, 1598, 1540, 1485, 1405, 1382, 1304, 1266, 1189, 1158, 1077, 1028, 928, 875, 842, 804, 790, 754, 690, 670, 595 cm^–1^; ^1^H NMR (500 MHz, CDCl_3_) δ 9.94 (s, 1H), 8.08 (d, *J* = 0.5 Hz, 1H), 7.46–7.44 (m, 1H), 7.39–7.34 (m, 2H), 7.04–7.00 (m, 2H); ^13^C­{^1^H} NMR (125 MHz, CDCl_3_) δ 184.2, 163.0 (*J*_C–F_ = 250 Hz), 155.1 (*J*_C–F_ = 2.5 Hz), 150.7, 131.3 (*J*_C–F_ = 8.8 Hz), 130.5 (*J*_C–F_ = 8.8 Hz), 130.3, 119.9 (*J*_C–F_ = 2.5 Hz), 115.4 (*J*_C–F_ = 21.3 Hz), 111.2 (*J*_C–F_ = 23.8 Hz), 102.3; ^19^F­{^1^H} NMR (470 MHz, CDCl_3_) δ −112.2; HRMS (EI) *m*/*z*: [M]^+^ Calcd for C_11_H_7_FO_2_ 190.0430; Found 190.0427.

##### 5-(4-Methoxy-2-nitrophenyl)-1*H*-pyrrole-3-carboxaldehyde (**4h**) and 5-(4-Methoxy-2-nitrophenyl) Furan-3-carboxaldehyde (**5h**)

Compounds **4h** and **5h** were synthesized from 5-((4-methoxy-2-nitrophenyl)­ethynyl)-1,2,3-triazine **3h** (0.5 mmol, 128 mg) under reaction conditions A and B, respectively. The crude product was purified by column chromatography on silica gel (EtOAc/toluene/hexane = 2:2:3) to afford the desired pyrrole **4h** (54% for condition A; 0% for condition B) and the desired furan **5h** (16% for condition A; 78% for condition B).

Compound **4h**: yellow solid; C_12_H_10_N_2_O_4_; mp 180–182 °C; TLC (EtOAc/hexane = 2:3) *R*_*f*_ = 0.10; IR ν_max_ (neat) 3244, 2922, 2851, 1641, 1530, 1487, 1422, 1382, 1344, 1296, 1270, 1226, 1180, 1147, 1043, 1026, 980, 898, 874, 818, 803, 757, 610 cm^–1^; ^1^H NMR (400 MHz, CDCl_3_) δ 9.80 (s, 1H), 9.26 (br s, NH, 1H), 7.49–7.47 (m, 2H), 7.36 (d, *J* = 2.7 Hz, 1H), 7.15 (dd, *J* = 8.8, 2.6 Hz, 1H), 6.77 (dd, *J* = 2.5, 1.8 Hz, 1H), 3.89 (s, 3H); ^13^C­{^1^H} NMR (125 MHz, CDCl_3_) δ 185.4, 159.6, 133.3, 129.3, 128.8, 127.51, 127.46, 119.5, 118.4, 109.6, 108.9, 56.0; HRMS (EI) *m*/*z*: [M]^+^ Calcd for C_12_H_10_N_2_O_4_ 246.0641; Found 246.0638.

Compound **5h**: dark red solid; C_12_H_9_NO_5_; mp 127–130 °C; TLC (EtOAc/hexane = 2:3) *R*_*f*_ = 0.28; IR ν_max_ (neat) 3113, 1688, 1591, 1519, 1311, 1284, 1179, 1149, 1036, 1003, 841, 804, 765, 601 cm^–1^; ^1^H NMR (500 MHz, CDCl_3_) δ 9.93 (s, 1H), 8.06 (s, 1H), 7.56 (d, *J* = 9.0 Hz, 1H), 7.32 (d, *J* = 2.5 Hz, 1H), 7.14 (dd, *J* = 9.0, 2.5 Hz, 1H), 6.88 (s, 1H), 3.89 (s, 3H); ^13^C­{^1^H} NMR (125 MHz, CDCl_3_) δ 184.1, 160.4, 151.6, 151.1, 148.6, 131.2, 130.0, 118.5, 115.7, 109.5, 105.0, 56.0; HRMS (EI) *m*/*z*: [M]^+^ Calcd for C_12_H_9_NO_5_ 247.0481; Found 247.0482.

##### 5-((4-Bromo-3-methylphenoxy)­methyl)-1*H*-pyrrole-3-carboxaldehyde (**4i**) and 5-((4-Bromo-3-methylphenoxy)­methyl) Furan-3-carboxaldehyde (**5i**)

Compounds **4i** and **5i** were synthesized from 5-(3-(4-bromo-3-methylphenoxy)­prop-1-yn-1-yl)-1,2,3-triazine **3i** (0.5 mmol, 152 mg) under reaction conditions A and B, respectively. The crude product was purified by column chromatography on silica gel (CH_2_Cl_2_/toluene/hexane = 2:2:3 to EtOAc/toluene/hexane = 2:2:3) to afford the desired pyrrole **4i** (50% for condition A; 7% for condition B) and the desired furan **5i** (12% for condition A; 68% for condition B).

Compound **4i**: yellow solid; C_13_H_12_BrNO_2_; mp 147–149 °C; TLC (EtOAc/hexane = 2:3) *R*_*f*_ = 0.19; IR ν_max_ (neat) 3209, 2917, 2849, 1640, 1558, 1506, 1418, 1198, 1108, 888, 817, 763, 720, 611 cm^–1^; ^1^H NMR (400 MHz, CDCl_3_) δ 9.77 (s, 1H), 8.91 (br s, NH, 1H), 7.42 (dd, *J* = 3.2, 1.6 Hz, 1H), 7.39 (d, *J* = 8.8 Hz, 1H), 6.82 (d, *J* = 2.9 Hz, 1H), 6.65–6.62 (m, 2H), 4.98 (s, 2H), 2.34 (s, 3H); ^13^C­{^1^H} NMR (100 MHz, CDCl_3_) δ 185.5, 157.2, 139.2, 133.0, 129.6, 127.6, 127.2, 117.4, 116.5, 113.7, 106.8, 63.0, 23.2; HRMS (EI) *m*/*z*: [M]^+^ Calcd for C_13_H_12_BrNO_2_ 293.0051; Found 293.0056.

Compound **5i**: brown solid; C_13_H_11_BrO_3_; mp 59–62 °C; TLC (EtOAc/hexane = 2:3) *R*_*f*_ = 0.50; IR ν_max_ (neat) 3116, 1675, 1576, 1542, 1478, 1456, 1406, 1377, 1302, 1237, 1173, 1146, 1016, 998, 911, 839, 856, 818, 795, 754, 692, 601 cm^–1^; ^1^H NMR (500 MHz, CDCl_3_) δ 9.89 (s, 1H), 8.05 (s, 1H), 7.39 (d, *J* = 8.7 Hz, 1H), 6.83 (d, *J* = 3.0 Hz, 1H), 6.78 (s, 1H), 6.65 (dd, *J* = 8.7, 3.0 Hz, 1H), 4.96 (s, 2H), 2.34 (s, 3H); ^13^C­{^1^H} NMR (125 MHz, CDCl_3_) δ 184.1, 157.1, 152.8, 151.5, 139.1, 132.9, 129.2, 117.5, 116.5, 113.7, 106.9, 62.1, 23.1; HRMS (EI) *m*/*z*: [M]^+^ Calcd for C_13_H_11_BrO_3_ 293.9892; Found 293.9897.

##### 5-(Methoxymethyl)-1*H*-pyrrole-3-carboxaldehyde (**4j**) and 5-(Methoxymethyl) Furan-3-carboxaldehyde (**5j**)

Compounds **4j** and **5j** were synthesized from 5-(3-methoxyprop-1-yn-1-yl)-1,2,3-triazine **3j** (0.5 mmol, 75 mg) under reaction conditions A and B, respectively. The crude product was purified by column chromatography on silica gel (EtOAc/toluene/hexane = 2:2:3 to EtOAc/hexane = 2:3) to afford the desired pyrrole **4j** (74% for condition A; 15% for condition B) and the desired furan **5j** (0% for condition A; 70% for condition B).

Compound **4j**: brown syrup; C_7_H_9_NO_2_; TLC (EtOAc/hexane = 2:3) *R*_*f*_ = 0.06; IR ν_max_ (neat) 3251, 2853, 1658, 1514, 1282, 1134, 829 cm^–1^; ^1^H NMR (400 MHz, CDCl_3_) δ 9.74 (s, 1H), 9.11 (br s, NH, 1H), 7.39 (dd, *J* = 2.8, 1.6 Hz, 1H), 6.55 (s, 1H), 4.41 (s, 2H), 3.32 (s, 3H); ^13^C­{^1^H} NMR (100 MHz, CDCl_3_) δ 185.7, 131.1, 127.6, 126.7, 106.5, 66.7, 57.7; HRMS (EI) *m*/*z*: [M]^+^ Calcd for C_7_H_9_NO_2_ 139.0633; Found 139.0632.

Compound **5j**: brown syrup; C_7_H_8_O_3_; TLC (EtOAc/hexane = 2:3) *R*_*f*_ = 0.38; IR ν_max_ (neat) 2853, 1683, 1542, 1286, 1135, 1085, 909, 833, 770, 604 cm^–1^; ^1^H NMR (500 MHz, CDCl_3_) δ 9.89 (s, 1H), 8.02 (s, 1H), 6.69 (s, 1H), 4.40 (s, 2H), 3.36 (s, 3H); ^13^C­{^1^H} NMR (125 MHz, CDCl_3_) δ 184.3, 154.3, 151.5, 129.1, 106.0, 65.9, 58.0; HRMS (EI) *m*/*z*: [M]^+^ Calcd for C_7_H_8_O_3_ 140.0473; Found 140.0471.

##### 5-(((Tetrahydro-2*H*-pyran-2-yl)­oxy)­methyl)-1*H*-pyrrole-3-carboxaldehyde (**4k**) and 5-(((Tetrahydro-2*H*-pyran-2-yl)­oxy)­methyl) Furan-3-carboxaldehyde (**5k**)

Compounds **4k** and **5k** were synthesized from 5-(3-((tetrahydro-2*H*-pyran-2-yl)­oxy)­prop-1-yn-1-yl)-1,2,3-triazine **3k** (0.5 mmol, 110 mg) under reaction conditions A and B, respectively. The crude product was purified by column chromatography on silica gel (CH_2_Cl_2_/toluene/hexane = 2:2:3 to EtOAc/toluene/hexane = 2:2:3) to afford the desired pyrrole **4k** (69% for condition A; 14% for condition B) and the desired furan **5k** (22% for condition A; 71% for condition B).

Compound **4k**: brown syrup; C_11_H_15_NO_3_; TLC (EtOAc/hexane = 2:3) *R*_*f*_ = 0.10; IR ν_max_ (neat) 3252, 2940, 1652, 1518, 1262, 1131, 1024, 1024, 772, 609, 588 cm^–1^; ^1^H NMR (400 MHz, CDCl_3_) δ 9.74 (s, 1H), 9.33 (br s, NH, 1H), 7.38 (dd, *J* = 2.8, 1.6 Hz, 1H), 6.53 (s, 1H), 4.62–4.60 (m, 3H), 3.95–3.90 (m, 1H), 3.57–3.51 (m, 1H), 1.83–1.71 (m, 2H), 1.57–1.51 (m, 4H); ^13^C­{^1^H} NMR (100 MHz, CDCl_3_) δ 185.6, 131.6, 127.3, 126.9, 106.2, 99.7, 63.7, 62.8, 30.7, 25.2, 20.2; HRMS (EI) *m*/*z*: [M]^+^ Calcd for C_11_H_15_NO_3_ 209.1052; Found 209.1053.

Compound **5k**: brown syrup; C_11_H_14_O_4_; TLC (EtOAc/hexane = 2:3) *R*_*f*_ = 0.48; IR ν_max_ (neat) 2970, 1738, 1683, 1542, 1365, 1217, 1135, 1023, 904, 870, 815, 772, 604, 527 cm^–1^; ^1^H NMR (500 MHz, CDCl_3_) δ 9.87 (s, 1H), 8.01 (s, 1H), 6.68 (s, 1H), 4.69 (t, *J* = 3.5 Hz, 1H), 4.65 (d, *J* = 13.1 Hz, 1H), 4.48 (d, *J* = 13.2 Hz, 1H), 3.87–3.83 (m, 1H), 3.52 (dt, *J* = 11.1, 4.2 Hz, 1H), 1.83–1.69 (m, 2H), 1.62–1.52 (m, 3H), 1.51–1.49 (m, 1H); ^13^C­{^1^H} NMR (125 MHz, CDCl_3_) δ 184.3, 154.6, 151.4, 129.2, 105.8, 97.5, 61.9, 60.2, 30.1, 25.2, 18.9; HRMS (EI) *m*/*z*: [M]^+^ Calcd for C_11_H_14_O_4_ 210.0892; Found 210.0887.

##### 5-((Benzyloxy)­methyl)-1*H*-pyrrole-3-carboxaldehyde (**4l**) and 5-((Benzyloxy)­methyl) Furan-3-carboxaldehyde (**5l**)

Compounds **4l** and **5l** were synthesized from 5-(3-(benzyloxy)­prop-1-yn-1-yl)-1,2,3-triazine **3l** (0.5 mmol, 113 mg) under reaction conditions A and B, respectively. The crude product was purified by column chromatography on silica gel (EtOAc/toluene/hexane = 2:2:3 to EtOAc/hexane = 2:3) to afford the desired pyrrole **4l** (67% for condition A; 8% for condition B) and the desired furan **5l** (8% for condition A; 87% for condition B).

Compound **4l**: brown syrup; C_13_H_13_NO_2_; TLC (EtOAc/hexane = 2:3) *R*_*f*_ = 0.19; IR ν_max_ (neat) 3250, 2857, 1651, 1514, 1452, 1326, 1132, 1068, 872, 774, 736, 698, 614 cm^–1^; ^1^H NMR (400 MHz, CDCl_3_) δ 9.73 (s, 1H), 9.16 (br s, NH, 1H), 7.36–7.27 (m, 6H), 6.55 (s, 1H), 4.50 (s, 2H), 4.48 (s, 2H); ^13^C­{^1^H} NMR (100 MHz, CDCl_3_) δ 185.7, 137.4, 131.1, 128.6 (2 ×), 128.0 (2 ×), 127.8, 126.9, 106.6 (2 ×), 71.9, 64.3; HRMS (EI) *m*/*z*: [M]^+^ Calcd for C_13_H_13_NO_2_ 215.0946; Found 215.0942.

Compound **5l**: brown syrup; C_13_H_12_O_3_; TLC (EtOAc/hexane = 2:3) *R*_*f*_ = 0.49; IR ν_max_ (neat) 2853, 1683, 1542, 1453, 1408, 1254, 1136, 1069, 910, 833, 770, 734, 697, 602 cm^–1^; ^1^H NMR (500 MHz, CDCl_3_) δ 9.89 (s, 1H), 8.03 (d, *J* = 0.9 Hz, 1H), 7.36–7.27 (m, 5H), 6.70 (s, 1H), 4.55 (s, 2H), 4.48 (s, 2H); ^13^C­{^1^H} NMR (125 MHz, CDCl_3_) δ 184.3, 154.5, 151.4, 137.3, 129.2, 128.5, 127.9, 127.8, 106.1, 72.2, 63.5; HRMS (EI) *m*/*z*: [M]^+^ Calcd for C_13_H_12_O_3_ 216.0786; Found 216.0784.

##### 5-(2-((Tetrahydro-2*H*-pyran-2-yl)­oxy)­ethyl)-1*H*-pyrrole-3-carboxaldehyde (**4m**) and 5-(2-((Tetrahydro-2*H*-pyran-2-yl)­oxy)­ethyl) Furan-3-carboxaldehyde (**5m**)

Compounds **4m** and **5m** were synthesized from 5-(4-((tetrahydro-2*H*-pyran-2-yl)­oxy)­but-1-yn-1-yl)-1,2,3-triazine **3m** (0.5 mmol, 117 mg) under reaction conditions A and B, respectively. The crude product was purified by column chromatography on silica gel (EtOAc/toluene/hexane = 2:2:3 to EtOAc/hexane = 2:3) to afford the desired pyrrole **4m** (69% for condition A; 12% for condition B) and the desired furan **5m** (16% for condition A; 70% for condition B).

Compound **4m**: brown syrup; C_12_H_17_NO_3_; TLC (EtOAc/hexane = 2:3) *R*_*f*_ = 0.11; IR ν_max_ (neat) 3265, 2870, 1651, 1516, 1260, 1120, 1029, 813, 767 cm^–1^; ^1^H NMR (500 MHz, CDCl_3_) δ 9.70 (s, 1H), 9.41 (br s, NH, 1H), 7.29 (dd, *J* = 3.0, 1.6 Hz, 1H), 6.35 (s, 1H), 4.57 (t, *J* = 2.9 Hz, 1H), 3.99–3.94 (m, 1H), 3.83–3.80 (m, 1H), 3.68–3.63 (m, 1H), 3.50–3.47 (m, 1H), 2.88–2.84 (m, 2H), 1.81–1.72 (m, 2H), 1.57–1.53 (m, 4H); ^13^C­{^1^H} NMR (125 MHz, CDCl_3_) δ 185.7, 133.6, 127.0, 126.7, 104.4, 99.9, 67.4, 63.3, 30.9, 27.7, 25.2, 20.2; HRMS (EI) *m*/*z*: [M]^+^ Calcd for C_12_H_17_NO_3_ 223.1208; Found 223.1202.

Compound **5m**: brown syrup; C_12_H_16_O_4_; TLC (EtOAc/hexane = 2:3) *R*_*f*_ = 0.46; IR ν_max_ (neat) 2934, 1682, 1543, 1276, 1132, 1073, 1029, 970, 906, 868, 812, 762, 602 cm^–1^; ^1^H NMR (500 MHz, CDCl_3_) δ 9.85 (s, 1H), 7.92 (s, 1H), 6.47 (s, 1H), 4.59 (t, *J* = 3.8 Hz, 1H), 3.97 (dt, *J* = 9.9, 6.7 Hz, 1H), 3.76 (ddd, *J* = 10.3, 8.1, 2.8 Hz, 1H), 3.65 (dt, *J* = 9.9, 6.7 Hz, 1H), 3.49–3.45 (m, 1H), 2.94 (t, *J* = 6.7 Hz, 2H), 1.81–1.73 (m, 1H), 1.70–1.65 (m, 2H), 1.59–1.47 (m, 3H); ^13^C­{^1^H} NMR (125 MHz, CDCl_3_) δ 184.4, 156.3, 150.4, 129.4, 102.9, 98.6, 64.6, 62.0, 30.3, 28.5, 25.1, 19.2; HRMS (EI) *m*/*z*: [M]^+^ Calcd for C_12_H_16_O_4_ 224.1049; Found 224.1044.

##### *tert*-Butyl ((4-Formyl-1*H*-pyrrol-2-yl)­methyl) Carbamate (**4n**) and *tert*-Butyl ((4-Formyl Furan-2-yl)­methyl) Carbamate (**5n**)

Compounds **4n** and **5n** were synthesized from *tert*-butyl­(3-(1,2,3-triazin-5-yl)­prop-2-yn-1-yl)­carbamate **3n** (0.5 mmol, 117 mg) under reaction conditions A and B, respectively. The crude product was purified by column chromatography on silica gel (EtOAc/toluene/hexane = 2:2:3) to afford the desired pyrrole **4n** (56% for condition A; 0% for condition B) and the desired furan **5n** (14% for condition A; 65% for condition B).

Compound **4n**: brown solid; C_11_H_16_N_2_O_3_; mp 108–110 °C; TLC (EtOAc/hexane = 2:3) *R*_*f*_ = 0.15; IR ν_max_ (neat) 3294, 3127, 1724, 1640, 1580, 1520, 1423, 1382, 1329, 1275, 1191, 1171, 1121, 988, 899, 847, 825, 749, 642, 619 cm^–1^; ^1^H NMR (400 MHz, CDCl_3_) δ 9.73 (s, 1H), 9.68 (br s, NH, 1H), 7.33 (q, *J* = 1.5 Hz, 1H), 6.42 (s, 1H), 5.08 (br s, BocNH, 1H), 4.15 (q, *J* = 6.0 Hz, 2H), 1.44 (s, 9H); ^13^C­{^1^H} NMR (100 MHz, CDCl_3_) δ 185.5, 158.1, 133.4, 127.6, 126.4, 105.0, 80.5, 37.3, 28.3; HRMS (EI) *m*/*z*: [M]^+^ Calcd for C_11_H_16_N_2_O_3_ 224.1161; Found 224.1155.

Compound **5n**: yellow solid; C_11_H_15_NO_4_; mp 96–99 °C; TLC (EtOAc/hexane = 2:3) *R*_*f*_ = 0.48; IR ν_max_ (neat) 3362, 1673, 1575, 1518, 1434, 1367, 1294, 1251, 1150, 1124, 1048, 1028, 956, 898, 842, 774, 748, 607 cm^–1^; ^1^H NMR (500 MHz, CDCl_3_) δ 9.86 (s, 1H), 7.96 (s, 1H), 6.58 (s, 1H), 4.85 (br s, BocNH, 1H), 4.31 (d, *J* = 5.0 Hz, 2H), 1.44 (s, 9H); ^13^C­{^1^H} NMR (125 MHz, CDCl_3_) δ 184.3, 155.5, 155.3, 150.8, 129.3, 103.7, 80.0, 37.4, 28.2; HRMS (EI) *m*/*z*: [M]^+^ Calcd for C_11_H_15_NO_4_ 225.1001; Found 225.0996.

##### 5-((Benzyl­(methyl)­amino)­methyl)-1*H*-pyrrole-3-carboxaldehyde (**4o**) and 5-((Benzyl­(methyl)­amino)­methyl) Furan-3-carboxaldehyde (**5o**)

Compounds **4o** and **5o** were synthesized from *N*-benzyl-*N*-methyl-3-(1,2,3-triazin-5-yl)­prop-2-yn-1-amine **3o** (0.5 mmol, 119 mg) under reaction conditions A and B, respectively. The crude product was purified by column chromatography on silica gel (EtOAc/toluene/hexane = 2:2:3 to EtOAc/hexane = 1:1) to afford the desired pyrrole **4o** (57% for condition A; 11% for condition B) and the desired furan **5o** (0% for condition A; 68% for condition B).

Compound **4o**: brown syrup; C_14_H_16_N_2_O; TLC (EtOAc/hexane = 2:3) *R*_*f*_ = 0.07; IR ν_max_ (neat) 3244, 2923, 2852, 2359, 1652, 1516, 1453, 1323, 1128, 1024, 824, 776, 737, 699, 617 cm^–1^; ^1^H NMR (500 MHz, CD_3_OD) δ 9.61 (s, 1H), 7.55 (dd, *J* = 1.5, 0.5 Hz, 1H), 7.35–7.30 (m, 4H), 7.27–7.24 (m, 1H), 6.49 (d, *J* = 1.0 Hz, 1H), 3.57 (s, 2H), 3.53 (s, 2H), 2.17 (s, 3H); ^13^C­{^1^H} NMR (125 MHz, CDCl_3_) δ 185.6, 137.4, 131.7, 129.2, 128.4, 127.6, 127.5, 126.8, 106.3, 61.3, 53.6, 41.8; HRMS (EI) *m*/*z*: [M]^+^ Calcd for C_14_H_16_N_2_O 228.1263; Found 228.1260.

Compound **5o**: brown syrup; C_14_H_15_NO_2_; TLC (EtOAc/hexane = 2:3) *R*_*f*_ = 0.25; IR ν_max_ (neat) 2794, 1683, 1540, 1435, 1134, 1024, 908, 825, 773, 734, 698, 603 cm^–1^; ^1^H NMR (500 MHz, CDCl_3_) δ 9.88 (s, 1H), 8.00 (d, *J* = 0.5 Hz, 1H), 7.31–7.30 (m, 4H), 7.27–7.22 (m, 1H), 6.59 (s, 1H), 3.57 (s, 2H), 3.54 (s, 2H), 2.24 (s, 3H); ^13^C­{^1^H} NMR (125 MHz, CDCl_3_) δ 184.4, 155.3, 151.1, 138.0, 129.1, 128.8, 128.2, 127.1, 105.3, 61.0, 52.7, 41.8; HRMS (EI) *m*/*z*: [M]^+^ Calcd for C_14_H_15_NO_2_ 229.1103; Found 229.1108.

##### 5-(3-(1,3-Dioxoisoindolin-2-yl) Propyl)-1*H*-pyrrole-3-carboxaldehyde (**4p**) and 5-(3-(1,3-Dioxoisoindolin-2-yl) Propyl) Furan-3-carboxaldehyde (**5p**)

Compounds **4p** and **5p** were synthesized from 2-(5-(1,2,3-triazin-5-yl)­pent-4-yn-1-yl)­isoindoline-1,3-dione **3p** (0.5 mmol, 146 mg) under reaction conditions A and B, respectively. The crude product was purified by column chromatography on silica gel (CH_2_Cl_2_/toluene/hexane = 2:2:3) to afford the desired pyrrole **4p** (55% for condition A; 0% for condition B) and the desired furan **5p** (17% for condition A; 80% for condition B).

Compound **4p**: yellow solid; C_16_H_14_N_2_O_3_; mp 199–201 °C; TLC (EtOAc/hexane = 3:2) *R*_*f*_ = 0.28; IR ν_max_ (neat) 3259, 2936, 1768, 1706, 1633, 1518, 1437, 1399, 1373, 1339, 1188, 1131, 1101, 1028, 993, 875, 850, 798, 769, 721, 625, 607 cm^–1^; ^1^H NMR (500 MHz, CDCl_3_) δ 9.77 (br s, NH, 1H), 9.71 (s, 1H), 7.85 (dd, *J* = 5.5, 3.0 Hz, 2H), 7.74 (dd, *J* = 5.4, 3.0 Hz, 2H), 7.34 (dd, *J* = 3.0, 1.6 Hz, 1H), 6.38 (s, 1H), 3.74–3.72 (m, 2H), 2.59 (t, *J* = 6.5 Hz, 2H), 1.99–1.94 (m, 2H); ^13^C­{^1^H} NMR (125 MHz, CDCl_3_) δ 185.6, 169.2, 134.7, 134.3, 131.9, 127.3, 126.6, 123.5, 104.5, 36.9, 29.3, 23.9; HRMS (EI) *m*/*z*: [M]^+^ Calcd for C_16_H_14_N_2_O_3_ 282.1004; Found 282.1006.

Compound **5p**: yellow crystal; C_16_H_13_NO_4_; mp 140–143 °C; TLC (EtOAc/hexane = 3:2) *R*_*f*_ = 0.63; IR ν_max_ (neat) 3284, 1767, 1704, 1678, 1545, 1437, 1399, 1372, 1333, 1138, 1100, 1025, 914, 875, 806, 778, 759, 725, 599 cm^–1^; ^1^H NMR (500 MHz, CDCl_3_) δ 9.79 (s, 1H), 7.87 (d, *J* = 0.7 Hz, 1H), 7.82 (dd, *J* = 5.2, 3.0 Hz, 2H), 7.70 (dd, *J* = 5.5, 3.1 Hz, 2H), 6.42 (s, 1H), 3.75 (t, *J* = 7.0 Hz, 2H), 2.72–2.69 (m, 2H), 2.05 (quintet, *J* = 7.4 Hz, 2H); ^13^C­{^1^H} NMR (125 MHz, CDCl_3_) δ 184.3, 168.1, 157.6, 150.4, 133.9, 131.8, 129.3, 123.1, 102.3, 37.1, 26.2, 25.2; HRMS (EI) *m*/*z*: [M]^+^ Calcd for C_16_H_13_NO_4_ 283.0845; Found 283.0844.

##### 5-(Triisopropylsilyl) Furan-3-carboxaldehyde (**5q**)

Compound **5q** was synthesized from 5-(triisopropylsilyl)-1-yn-1-yl)-1,2,3-triazine **3q** (0.5 mmol, 131 mg) under reaction conditions A and B, respectively. The crude product was purified by column chromatography on silica gel (CH_2_Cl_2_/hexane = 1:2) to afford the desired furan **5q** (0% for condition A; 41% for condition B).

Compound **5q**: yellow syrup; C_14_H_24_O_2_Si; TLC (EtOAc/hexane = 2:3) *R*_*f*_ = 0.75; IR ν_max_ (neat) 2944, 2866, 1687, 1567, 1463, 1370, 1134, 1050, 996, 882, 837, 751, 678, 659, 598 cm^–1^; ^1^H NMR (500 MHz, CDCl_3_) δ 9.95 (s, 1H), 8.26 (d, *J* = 0.3 Hz, 1H), 7.02 (s, 1H), 1.29 (heptet, *J* = 7.7 Hz, 3H), 1.07 (d, *J* = 7.5 Hz, 18H); ^13^C­{^1^H} NMR (125 MHz, CDCl_3_) δ 184.6, 161.3, 155.5, 128.7, 118.1, 18.4, 10.9; HRMS (EI) *m*/*z*: [M]^+^ Calcd for C_14_H_24_O_2_Si 252.1546; Found 252.1541.

##### 5-Propyl-1*H*-pyrrole-3-carboxaldehyde (**4r**) and 5-Propyl Furan-3-carboxaldehyde (**5r**)

Compounds **4r** and **5r** were synthesized from 5-(pent-1-yn-1-yl)-1,2,3-triazine **3r** (0.5 mmol, 74 mg) under reaction conditions A and B, respectively. The crude product was purified by column chromatography on silica gel (CH_2_Cl_2_/toluene/hexane = 2:2:3 to EtOAc/toluene/hexane = 2:2:3) to afford the desired pyrrole **4r** (73% for condition A; 10% for condition B) and the desired furan **5r** (0% for condition A; 76% for condition B).

Compound **4r**: brown solid; C_8_H_11_NO; mp 63–66 °C; TLC (EtOAc/hexane = 2:3) *R*_*f*_ = 0.25; IR ν_max_ (neat) 3234, 2956, 1634, 1516, 1419, 1133. 817, 777, 713, 615 cm^–1^; ^1^H NMR (400 MHz, CDCl_3_) δ 9.70 (s, 1H), 8.67 (br s, NH, 1H), 7.29 (dd, *J* = 2.8, 1.6 Hz, 1H), 6.35 (s, 1H), 2.54 (t, *J* = 7.6 Hz, 2H), 1.63 (hextet, *J* = 7.6 Hz, 2H), 0.94 (t, *J* = 7.6 Hz, 3H); ^13^C­{^1^H} NMR (100 MHz, CDCl_3_) δ 185.7, 135.7, 127.2, 126.6, 103.8, 29.4, 22.3, 13.7; HRMS (EI) *m*/*z*: [M]^+^ Calcd for C_8_H_11_NO 137.0841; Found 137.0841.

Compound **5r**: brown syrup; C_8_H_10_O_2_ TLC (EtOAc/hexane = 2:3) *R*_*f*_ = 0.60; IR ν_max_ (neat) 2961, 2873, 1678, 1547, 1462, 1408, 1275, 1141, 815 cm^–1^; ^1^H NMR (400 MHz, CDCl_3_) δ 9.84 (s, 1H), 7.90 (s, 1H), 6.36 (s, 1H), 2.61–2.57 (m, 2H), 1.65 (sext, *J* = 7.4 Hz, 2H), 0.94 (t, *J* = 7.3 Hz, 3H); ^13^C­{^1^H} NMR (100 MHz, CDCl_3_) δ 184.6, 159.4, 150.3, 129.5, 101.9, 29.7, 20.9, 13.5; HRMS (EI) *m*/*z*: [M]^+^ Calcd for C_8_H_10_O_2_ 138.0681; Found 138.0681.

##### 5-Heptyl-1*H*-pyrrole-3-carboxaldehyde (**4s**) and 5-Heptyl Furan-3-carboxaldehyde (**5s**)

Compounds **4s** and **5s** were synthesized from 5-(non-1-yn-1-yl)-1,2,3-triazine **3s** (0.5 mmol, 102 mg) under reaction conditions A and B, respectively. The crude product was purified by column chromatography on silica gel (CH_2_Cl_2_/toluene/hexane = 2:2:3) to afford the desired pyrrole **4s** (69% for condition A; 0% for condition B) and the desired furan **5s** (17% for condition A; 78% for condition B).

Compound **4s**: brown syrup; C_12_H_19_NO; TLC (EtOAc/hexane = 1:4) *R*_*f*_ = 0.14; IR ν_max_ (neat) 3264, 2918, 2849, 1645, 1518, 1422, 1276, 1260, 1127, 815, 750, 611 cm^–1^; ^1^H NMR (500 MHz, CDCl_3_) δ 9.70 (s, 1H), 8.66 (br s, NH, 1H), 7.29 (dd, *J* = 3.1, 1.7 Hz, 1H), 6.34 (d, *J* = 0.8 Hz, 1H), 2.57–2.54 (m, 2H), 1.60 (quintet, *J* = 7.5 Hz, 2H), 1.33–1.22 (m, 8H), 0.85 (t, *J* = 7.0 Hz, 3H); ^13^C­{^1^H} NMR (125 MHz, CDCl_3_) δ 185.7, 135.9, 127.2, 126.6, 103.7, 31.7, 29.1, 29.04, 29.00, 27.4, 22.6, 14.0; HRMS (EI) *m*/*z*: [M]^+^ Calcd for C_12_H_19_NO 193.1467; Found 193.1464.

Compound **5s**: brown syrup; C_12_H_18_O_2_; TLC (EtOAc/hexane = 2:3) *R*_*f*_ = 0.7; IR ν_max_ (neat) 2927, 1737, 1564, 1365, 1217, 1131, 776, 724, 528 cm^–1^; ^1^H NMR (500 MHz, CDCl_3_) δ 9.84 (s, 1H), 7.90 (s, 1H), 6.35 (s, 1H), 2.61 (t, *J* = 7.5 Hz, 2H), 1.62 (quintet, *J* = 7.6 Hz, 2H), 1.30–1.21 (m, 8H), 0.86 (t, *J* = 6.7 Hz, 3H); ^13^C­{^1^H} NMR (125 MHz, CDCl_3_) δ 184.7, 159.6, 150.4, 129.4, 101.7, 31.6, 28.9, 28.8, 27.7, 27.5, 22.5, 14.0; HRMS (EI) *m*/*z*: [M]^+^ Calcd for C_12_H_18_O_2_ 194.1307; Found 194.1305.

##### 5-(*tert*-Butyl)-1*H*-pyrrole-3-carboxaldehyde (**4t**) and 5-(*tert*-Butyl) Furan-3-carboxaldehyde (**5t**)

Compounds **4t** and **5t** were synthesized from 5-(3,3-dimethylbut-1-yn-1-yl)-1,2,3-triazine **3t** (0.5 mmol, 81 mg) under reaction conditions A and B, respectively. The crude product was purified by column chromatography on silica gel (CH_2_Cl_2_/hexane = 1:2) to afford desired pyrrole **4t** (70% for condition A; 0% for condition B) and desired furan **5t** (0% for condition A; 71% for condition B).

Compound **4t**: brown solid; C_9_H_13_NO; mp 123–125 °C; TLC (EtOAc/hexane = 2:3) *R*_*f*_ = 0.34; IR ν_max_ (neat) 3201, 2963, 1633, 1514, 1438, 1384, 1363, 1275, 1258, 1222, 1191, 1143, 1102, 821, 759, 621 cm^–1^; ^1^H NMR (500 MHz, CDCl_3_) δ 9.73 (s, 1H), 8.42 (br s, NH, 1H), 7.31–7.30 (m, 1H), 6.39–6.38 (m, 1H), 1.29 (s, 9H); ^13^C­{^1^H} NMR (125 MHz, CDCl_3_) δ 186.0, 145.1, 127.4, 126.4, 101.0, 31.3, 30.0; HRMS (EI) *m*/*z*: [M]^+^ Calcd for C_9_H_13_NO 151.0997; Found 151.0996.

Compound **5t**: yellowish syrup; C_9_H_12_O_2_; TLC (EtOAc/hexane = 2:3) *R*_*f*_ = 0.63; IR ν_max_ (neat) 2969, 1738, 1683, 1462, 1365, 1235, 1141, 1052, 918, 822, 755, 604 cm^–1^; ^1^H NMR (400 MHz, CDCl_3_) δ 9.85 (s, 1H), 7.92 (d, *J* = 0.8 Hz, 1H), 6.34 (s, 1H), 1.27 (s, 9H); ^13^C­{^1^H} NMR (100 MHz, CDCl_3_) δ 184.7, 167.2, 150.3, 129.3, 99.1, 32.7, 28.7; HRMS (EI) *m*/*z*: [M]^+^ Calcd for C_9_H_12_O_2_ 152.0837; Found 152.0832.

Note: these compounds are easily volatile.

##### 5-(Prop-1-en-2-yl)-1*H*-pyrrole-3-carboxaldehyde (**4u**) and 5-(Prop-1-en-2-yl) Furan-3-carboxaldehyde (**5u**)

Compounds **4u** and **5u** were synthesized from 5-(3-methylbut-3-en-1-yn-1-yl)-1,2,3-triazine **3u** (0.5 mmol, 73 mg) under reaction conditions A and B, respectively. The crude product was purified by column chromatography on silica gel (CH_2_Cl_2_/toluene/hexane = 2:2:3 to EtOAc/toluene/hexane = 2:2:3) to afford the desired pyrrole **4u** (72% for condition A; 18% for condition B) and the desired furan **5u** (0% for condition A; 78% for condition B).

Compound **4u**: yellow solid; C_8_H_9_NO; mp 64–66 °C; TLC (EtOAc/hexane = 2:3) *R*_*f*_ = 0.26; IR ν_max_ (neat) 3209, 2918, 2849, 1640, 1558, 1506, 1418, 1198, 1108, 888, 817, 763, 720, 611 cm^–1^; ^1^H NMR (400 MHz, CDCl_3_) δ 9.76 (s, 1H), 8.94 (br s, NH, 1H), 7.38 (dd, *J* = 2.8, 1.2 Hz, 1H), 6.64 (t, *J* = 2.0 Hz, 1H), 5.11 (s, 1H), 4.96 (d, *J* = 1.6 Hz, 1H), 2.06 (s, 3H); ^13^C­{^1^H} NMR (100 MHz, CDCl_3_) δ 185.6, 135.4, 134.2, 127.62, 127.56, 108.3, 105.1, 20.4; HRMS (EI) *m*/*z*: [M]^+^ Calcd for C_8_H_9_NO 135.0684; Found 135.0685.

Compound **5u**: yellow syrup; C_8_H_8_O_2_; TLC (EtOAc/hexane = 2:3) *R*_f_ = 0.72; IR ν_max_ (neat) 3370, 2876, 1678, 1630, 1547, 1260, 1147, 1064, 759, 603 cm^–1^; ^1^H NMR (400 MHz, CDCl_3_) δ 9.88 (s, 1H), 7.95 (s, 1H), 6.61 (s, 1H), 5.57 (s, 1H), 5.08 (t, *J* = 1.6 Hz, 1H), 2.01 (s, 3H); ^13^C­{^1^H} NMR (100 MHz, CDCl_3_) δ 184.4, 157.4, 150.6, 131.9, 130.0, 112.6, 102.5, 19.1; HRMS (EI) *m*/*z*: [M]^+^ Calcd for C_8_H_8_O_2_ 136.0524; Found 136.0521.

Note: these compounds are easily volatile.

##### 5-(3-Chloropropyl)-1*H*-pyrrole-3-carboxaldehyde (**4v**) and 5-(3-Chloropropyl) Furan-3-carboxaldehyde (**5v**)

Compounds **4v** and **5v** were synthesized from 5-(5-chloropent-1-yn-1-yl)-1,2,3-triazine **3v** (0.5 mmol, 91 mg) under reaction conditions A and B, respectively. The crude product was purified by column chromatography on silica gel (CH_2_Cl_2_/toluene/hexane = 2:2:3 to EtOAc/toluene/hexane = 2:2:3) to afford the desired pyrrole **4v** (72% for condition A; 10% for condition B) and the desired furan **5v** (15% for condition A; 78% for condition B).

Compound **4v**: brown syrup; C_8_H_10_ClNO; TLC (EtOAc/hexane = 2:3) *R*_*f*_ = 0.15; IR ν_max_ (neat) 2920, 1715, 1293, 1049, 650 cm^–1^; ^1^H NMR (500 MHz, CDCl_3_) δ 9.71 (s, 1H), 8.91 (br s, NH, 1H), 7.32 (q, *J* = 2.0 Hz, 1H), 6.38 (s, 1H), 3.54 (t, *J* = 6.0 Hz, 2H), 2.77 (t, *J* = 7.0 Hz, 2H), 2.06 (quintet, *J* = 6.3 Hz, 2H); ^13^C­{^1^H} NMR (125 MHz, CDCl_3_) δ 185.8, 133.9, 127.3, 127.1, 104.2, 44.0, 31.7, 24.3; HRMS (EI) *m*/*z*: [M]^+^ Calcd for C_8_H_10_ClNO 171.0451; Found 171.0452.

Compound **5v**: brown syrup; C_8_H_9_ClO_2_; TLC (EtOAc/hexane = 2:3) *R*_*f*_ = 0.53; IR ν_max_ (neat) 3284, 1640, 1608, 1515, 1454, 1435, 1415, 1192, 1120, 807, 756, 723, 690, 655, 614 cm^–1^; ^1^H NMR (500 MHz, CDCl_3_) δ 9.85 (s, 1H), 7.30 (d, *J* = 0.3 Hz, 1H), 6.43 (s, 1H), 3.55 (t, *J* = 6.5 Hz, 2H), 2.82 (t, *J* = 7.5 Hz, 2H), 2.10 (quintet, *J* = 6.5 Hz, 2H); ^13^C­{^1^H} NMR (125 MHz, CDCl_3_) δ 184.5, 157.4, 150.6, 129.4, 102.6, 43.6, 30.2, 24.9; HRMS (EI) *m*/*z*: [M]^+^ Calcd for C_8_H_9_ClO_2_ 172.0291; Found 172.0287.

##### 4-(4-Formyl-1H-pyrrol-2-yl) Butanenitrile (**4w**) and 4-(4-Formyl Furan-2-yl) Butanenitrile (**5w**)

Compounds **4w** and **5w** were synthesized from 6-(1,2,3-triazin-5-yl) hex-5-ynenitrile **3w** (0.5 mmol, 86 mg) under reaction conditions A and B, respectively. The crude product was purified by column chromatography on silica gel (CH_2_Cl_2_/toluene/hexane = 2:2:3 to EtOAc/toluene/hexane = 2:2:3) to afford the desired pyrrole **4w** (63% for condition A; 6% for condition B) and the desired furan **5w** (15% for condition A; 80% for condition B).

Compound **4w**: brown syrup; C_9_H_10_N_2_O; TLC (EtOAc/hexane = 3:2) *R*_*f*_ = 0.23; IR ν_max_ (neat) 3295, 2359, 1644, 1514, 1421, 1296, 1127, 821, 762 cm^–1^; ^1^H NMR (500 MHz, CDCl_3_) δ 9.72 (s, 1H), 8.93 (br s, NH, 1H), 7.33 (d, *J* = 1.5 Hz, 1H), 6.39 (s, 1H), 2.77 (t, *J* = 7.5 Hz, 2H), 2.38 (t, *J* = 7.0 Hz, 2H), 1.98 (quintet, *J* = 7.1 Hz, 2H); ^13^C­{^1^H} NMR (125 MHz, CDCl_3_) δ 185.6, 132.7, 127.3, 127.1, 119.2, 104.5, 26.0, 24.8, 16.4; HRMS (EI) *m*/*z*: [M]^+^ Calcd for C_9_H_10_N_2_O 162.0793; Found 162.0792.

Compound **5w**: brown syrup; C_9_H_9_NO_2_; TLC (EtOAc/hexane = 3:2) *R*_*f*_ = 0.45; IR ν_max_ (neat) 2360, 1682, 1545, 1422, 1250, 1134, 758 cm^–1^; ^1^H NMR (500 MHz, CDCl_3_) δ 9.85 (s, 1H), 7.95 (d, *J* = 0.5 Hz, 1H), 6.46 (s, 1H), 2.82 (t, *J* = 7.5 Hz, 2H), 2.38 (t, *J* = 7.5 Hz, 2H), 2.01 (quintet, *J* = 7.5 Hz, 2H); ^13^C­{^1^H} NMR (125 MHz, CDCl_3_) δ 184.4, 156.3, 150.8, 129.2, 118.8, 103.0, 26.3, 23.3, 16.2; HRMS (EI) *m*/*z*: [M]^+^ Calcd for C_9_H_9_NO_2_ 163.0633; Found 163.0630.

##### Methyl 3-(4-Formyl-1*H*-pyrrol-2-yl) Propanoate (**4x**) and Methyl 3-(4-Formyl Furan-2-yl) Propanoate (**5x**)

Compounds **4x** and **5x** were synthesized from methyl 5-(1,2,3-triazin-5-yl)­pent-4-ynoate **3x** (0.5 mmol, 96 mg) under reaction conditions A and B, respectively. The crude product was purified by column chromatography on silica gel (EtOAc/toluene/hexane = 2:2:3) to afford the desired pyrrole **4x** (60% for condition A; 0% for condition B) and the desired furan **5x** (11% for condition A; 73% for condition B).

Compound **4x**: brown solid; C_9_H_11_NO_3_; mp 73–75 °C; TLC (EtOAc/hexane = 2:3) *R*_*f*_ = 0.10; IR ν_max_ (neat) 3380, 2981, 1682, 1633, 1504, 1453, 1420, 1390, 1366, 1330, 1244, 1157, 1120, 1045, 1026, 991, 863, 820, 757, 691, 642 cm^–1^; ^1^H NMR (500 MHz, CDCl_3_) δ 9.70 (s, 1H), 9.30 (br s, NH, 1H), 7.28 (dd, *J* = 3.0, 1.5 Hz, 1H), 6.35 (s, 1H), 3.70 (s, 3H), 2.88–2.86 (m, 2H), 2.65–2.63 (m, 2H); ^13^C­{^1^H} NMR (125 MHz, CDCl_3_) δ 185.5, 174.9, 134.0, 127.0, 126.9, 104.5, 52.1, 33.8, 22.1; HRMS (EI) *m*/*z*: [M]^+^ Calcd for C_9_H_11_NO_3_ 181.0739; Found 181.0739.

Compound **5x**: brown syrup; C_9_H_10_O_4_; TLC (EtOAc/hexane = 2:3) *R*_*f*_ = 0.43; IR ν_max_ (neat) 2924, 1732, 1682, 1544, 1438, 1366, 1134, 763, 601 cm^–1^; ^1^H NMR (500 MHz, CDCl_3_) δ 9.83 (s, 1H), 7.92 (d, *J* = 0.5 Hz, 1H), 6.41 (s, 1H), 3.67 (s, 3H), 2.97 (t, *J* = 7.5 Hz, 2H), 2.65 (t, *J* = 7.5 Hz, 2H); ^13^C­{^1^H} NMR (125 MHz, CDCl_3_) δ 184.4, 172.4, 157.2, 150.5, 129.5, 102.6, 51.9, 31.9, 23.2; HRMS (EI) *m*/*z*: [M]^+^ Calcd for C_9_H_10_O_4_ 182.0579; Found 182.0583.

##### (4-Formyl-1*H*-pyrrol-2-yl) Methyl Acetate (**4y**) and (4-Formyl Furan-2-yl) Methyl Acetate (**5y**)

Compounds **4y** and **5y** were synthesized from 3-(1,2,3-triazin-5-yl)­prop-2-yn-1-yl acetate **3y** (0.5 mmol, 90 mg) under reaction conditions A and B, respectively. The crude product was purified by column chromatography on silica gel (EtOAc/toluene/hexane = 2:2:3) to afford the desired pyrrole **4y** (59% for condition A; 0% for condition B) and the desired furan **5y** (10% for condition A; 73% for condition B).

Compound **4y**: brown syrup; C_8_H_9_NO_3_; TLC (EtOAc/hexane = 2:3) *R*_*f*_ = 0.09; IR ν_max_ (neat) 3274, 2927, 1732, 1652, 1517, 1419, 1378, 1236, 1134, 1021, 958, 828, 749, 617, 609, 597 cm^–1^; ^1^H NMR (500 MHz, CDCl_3_) δ 9.75 (s, 1H), 9.21 (br s, NH, 1H), 7.38 (q, *J* = 1.6 Hz, 1H), 6.66 (t, *J* = 1.9 Hz, 1H), 5.01 (s, 2H), 2.07 (s, 3H); ^13^C­{^1^H} NMR (125 MHz, CDCl_3_) δ 185.5, 172.9, 129.6, 127.7, 126.7, 109.0, 58.9, 20.9; HRMS (EI) *m*/*z*: [M]^+^ Calcd for C_8_H_9_NO_3_ 167.0582; Found 167.0582.

Compound **5y**: brown syrup; C_8_H_8_O_4_; TLC (EtOAc/hexane = 2:3) *R*_*f*_ = 0.38; IR ν_max_ (neat) 2924, 1744, 1686, 1542, 1378, 1229, 1140, 1028, 913, 843, 774 cm^–1^; ^1^H NMR (500 MHz, CDCl_3_) δ 9.88 (s, 1H), 8.02 (d, *J* = 0.5 Hz, 1H), 6.76 (s, 1H), 5.05 (s, 2H), 2.07 (s, 3H); ^13^C­{^1^H} NMR (125 MHz, CDCl_3_) δ 184.1, 170.3, 152.2, 151.4, 129.3, 107.3, 57.5, 20.6; HRMS (EI) *m*/*z*: [M]^+^ Calcd for C_8_H_8_O_4_ 168.0423; Found 168.0422.

##### 2-(4-Formyl-1*H*-pyrrol-2-yl) Ethyl Acetate (**4z**) and 2-(4-Formyl Furan-2-yl) Ethyl Acetate (**5z**)

Compounds **4z** and **5z** were synthesized from 4-(1,2,3-triazin-5-yl)­but-3-yn-1-yl acetate **3z** (0.5 mmol, 96 mg) under reaction conditions A and B, respectively. The crude product was purified by column chromatography on silica gel (EtOAc/toluene/hexane = 2:2:3 to EtOAc/hexane = 2:3) to afford the desired pyrrole **4z** (75% for condition A; 11% for condition B) and the desired furan **5z** (10% for condition A; 70% for condition B).

Compound **4z**: brown syrup; C_9_H_11_NO_3_; TLC (EtOAc/hexane = 2:3) *R*_*f*_ = 0.13; IR ν_max_ (neat) 3304, 1739, 1659, 1518, 1239, 1129, 1035, 821, 767, 608 cm^–1^; ^1^H NMR (500 MHz, CDCl_3_) δ 9.70 (s, 1H), 9.24 (br s, NH, 1H), 7.32 (dd, *J* = 3.2, 1.7 Hz, 1H), 6.41 (s, 1H), 4.26 (t, *J* = 6.5 Hz, 2H), 2.91 (t, *J* = 6.5 Hz, 2H), 2.05 (s, 3H); ^13^C­{^1^H} NMR (125 MHz, CDCl_3_) δ 185.5, 170.9, 131.3, 127.3, 126.8, 105.3, 63.5, 27.1, 21.0; HRMS (EI) *m*/*z*: [M]^+^ Calcd for C_9_H_11_NO_3_ 181.0739; Found 181.0739.

Compound **5z**: brown syrup; C_9_H_10_O_4_; TLC (EtOAc/hexane = 2:3) *R*_*f*_ = 0.50; IR ν_max_ (neat) 2919, 2850, 1711, 1462, 1367, 1237, 1038, 720, 608 cm^–1^; ^1^H NMR (500 MHz, CDCl_3_) δ 9.85 (s, 1H), 7.94 (d, *J* = 0.5 Hz, 1H), 6.48 (s, 1H), 4.30 (t, *J* = 6.5 Hz, 2H), 2.97 (t, *J* = 6.5 Hz, 2H), 2.02 (s, 3H); ^13^C­{^1^H} NMR (125 MHz, CDCl_3_) δ 184.4, 170.7, 154.9, 150.7, 129.4, 103.5, 61.4, 27.4, 20.7; HRMS (FAB) *m*/*z*: [M]^+^ Calcd for C_9_H_11_O_4_ 183.0657; Found 183.0659.

##### 3-(4-Formyl-1*H*-pyrrol-2-yl) Propyl Acetate (**4a’**) and 3-(4-Formyl Furan-2-yl) Propyl Acetate (**5a’**)

Compounds **4a’** and **5a’** were synthesized from 5-(1,2,3-triazin-5-yl)­pent-4-yn-1-yl acetate **3a’** (0.5 mmol, 103 mg) under reaction conditions A and B, respectively. The crude product was purified by column chromatography on silica gel (EtOAc/toluene/hexane = 2:2:3) to afford the desired pyrrole **4a’** (69% for condition A; 0% for condition B) and the desired furan **5a’** (9% for condition A; 72% for condition B).

Compound **4a’**: brown syrup; C_10_H_13_NO_3_; TLC (EtOAc/hexane = 2:3) *R*_*f*_ = 0.06; IR ν_max_ (neat) 3257, 2925, 1717, 1645, 1518, 1422, 1367, 1259, 1128, 1038, 818, 750, 625 cm^–1^; ^1^H NMR (500 MHz, CDCl_3_) δ 9.72 (s, 1H), 8.92 (br s, NH, 1H), 7.30 (dd, *J* = 3.0, 1.6 Hz, 1H), 6.37 (s, 1H), 4.13 (t, *J* = 6.2 Hz, 2H), 2.64 (t, *J* = 7.2 Hz, 2H), 2.07 (s, 3H), 1.96–1.91 (m, 2H); ^13^C­{^1^H} NMR (125 MHz, CDCl_3_) δ 185.6, 171.7, 134.2, 127.3, 126.7, 104.3, 63.3, 28.7, 23.6, 21.0; HRMS (EI) *m*/*z*: [M]^+^ Calcd for C_10_H_13_NO_3_ 195.0895; Found 195.0901.

Compound **5a’**: yellow syrup; C_10_H_12_O_4_; TLC (EtOAc/hexane = 2:3) *R*_*f*_ = 0.52; IR ν_max_ (neat) 2928, 1731, 1681, 1545, 1367, 1239, 1135, 1039, 759, 603 cm^–1^; ^1^H NMR (500 MHz, CDCl_3_) δ 9.84 (s, 1H), 7.92 (d, *J* = 0.5 Hz, 1H), 6.40 (s, 1H), 4.08 (t, *J* = 6.5 Hz, 2H), 2.72 (t, *J* = 7.5 Hz, 2H), 2.03 (s, 3H), 1.97 (quintet, *J* = 7.5 Hz, 2H); ^13^C­{^1^H} NMR (125 MHz, CDCl_3_) δ 184.4, 170.9, 157.8, 150.5, 129.3, 102.2, 63.1, 26.5, 24.2, 20.7; HRMS (EI) *m*/*z*: [M]^+^ Calcd for C_10_H_12_O_4_ 196.0736; Found 196.0730.

## Supplementary Material



## Data Availability

The data underlying this study are available in the published article and its Supporting Information.

## Notes

Synthesis and characterization of detailed experimental procedures and ^1^H and ^13^C­{^1^H} NMR spectra for all compounds (PDF)
