# Supplementary material for: Molecular Editing of 5‑Alkynyl-1,2,3-triazines via a Silver-Mediated Skeletal Remodeling Approach: Solvent-Controlled Switchable Synthesis of Functionalized Pyrroles and Furans
Source: J Org Chem. 2025 Oct 22;90(43):15191–203. doi: 10.1021/acs.joc.5c01536 (PMC12584106; doi:10.1021/acs.joc.5c01536)
Supplement: Supplementary file 1 [file jo5c01536_si_001.pdf]

Supporting Information

Molecular Editing of 5-Alkynyl-1,2,3-triazines via a Silver-Mediated Skeletal Remodeling Approach: Solvent-Controlled Switchable Synthesis of Functionalized Pyrroles and Furans

Hsiang-Wen Chen, Wan-Hsuan Liu, Chia-Hao Chang and Jiun-Jie Shie*

[†]Institute of Chemistry, Academia Sinica, Taipei, 11529, Taiwan.

E-mail: shiejj@gate.sinica.edu.tw

**Table of Contents**	**Page number**
**1. Materials and Methods**	**S2**
**2. Synthetic Procedures and Product Characterization**	**S2**
**2-1. Synthetic procedure and product characterization of 5-bromo-1,2,3-triazines (1)**	**S2**
**2-1. General synthetic procedure and product characterization of 5-alkynyl-1,2,3-triazines (3a–3a')**	**S3**
**Table S1. Palladium-catalyzed Sonogashira coupling reactions of 5-bromo-1,2,3-triazines (1) with various terminal alkynes (2)**	**S4**
**4. Reference**	**S15**
**5. Copies of ^{1}H and ^{13}C NMR spectra**	**S16–S99**

1. Materials and Methods.

All reagents were commercially available and used without further purification unless otherwise indicated. All solvents were anhydrous grade unless indicated otherwise. All nonaqueous reactions were carried out in an oven-dried glassware under a slight positive pressure of argon unless noted otherwise. The reactions were magnetically stirred and monitored by thin-layer chromatography on silica gel. Flash chromatography was performed on silica gel with a 60–200 μm particle size. All reactions requiring heating were performed using a heating mantle. The yields are reported for spectroscopically pure compounds. Melting points were recorded on a Fargo MP-2D melting point apparatus and were not corrected. ^{1}H and ^{13}C NMR spectra were recorded on Bruker AV 600 (600 MHz), Bruker AV 500 (500 MHz), and Bruker AVIII 400 (400 MHz) spectrometers. Chemical shifts are given in δ values relative to tetramethylsilane (TMS, δ_H = 0); coupling constants *J* are given in Hz. The internal standards used for ^{1}H NMR spectroscopy were $CDCl_3$ (δ_H = 7.24) or CD_3OD (δ_H = 3.31). $CDCl_3$ (δ_C = 77.0, central line of triplet) or CD_3OD (δ_C = 49.0) was used for ^{13}C NMR spectroscopy. The splitting patterns are reported as s (singlet), d (doublet), t (triplet), q (quartet), m (multiplet), br (broad), and dd (double of doublets). IR spectra were recorded on a Thermo Nicolet iS5 FT-IR spectrometer. High-resolution electrospray ionization (ESI) mass spectra were recorded on a JMS-T100LP AccuTOF LC-plus 4G mass spectrometer. High-resolution fast atom bombardment (FAB) mass spectra were recorded on a JMS-700 double focusing mass spectrometer (JEOL, Tokyo, Japan).

2. Synthetic Procedures and Product Characterization

2-1. Synthetic procedure and product characterization of 5-bromo-1,2,3-triazines (1)

5-Bromo-1,2,3-triazine (1)[S1]

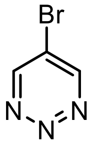

Preparation of NH_2Cl in ether:

A cold (–8 °C) solution of NH_4Cl (112 mmol, 6.0 g, 5.6 equiv) and NH_4OH (9.4 mL) in ether (170 mL) was added dropwise NaClO (114 mL) for 15 min. After stirring for 40 min, the reaction mixture was quenched with brine. The organic layer was dried over $MgSO_4$, filtrated and the fresh prepared solution of NH_2Cl in ether was used in the next step without further treatment.

A solution of 4-bromo-1*H*-pyrazole (20 mmol, 2.94 g, 1 equiv) in anhydrous DMF (30 mL) was added NaH

(22 mmol, 0.88 g, 60% dispersion in mineral oil). After stirring for 45 min from 0 °C to room temperature, the reaction mixture was added dropwise a solution of NH_2Cl in ether and then stirring for 30 min. The reaction mixture was quenched with saturated $Na_2S_2O_3$ solution and extracted with CH_2Cl_2. The organic layer was washed with brine, dried over $MgSO_4$ and concentrated. The crude residue was dissolved in CH_2Cl_2 (225 mL), and then H_2O (300 mL) was added. The reaction mixture was cooled to 0 °C, $NaIO_4$ (40 mmol, 8.57 g) was added, and the reaction mixture was stirred for 4.5 h from 0 °C to room temperature. The reaction mixture was extracted with CH_2Cl_2. The organic layer was washed with brine, dried over $MgSO_4$ and concentrated. The crude product was purified by flash column chromatography on silica gel (CH_2Cl_2) to afford the desired product **1** (2.19 g, 69%) as a light brown solid. $C_3H_2BrN_3$; mp 111–113 °C; TLC (CH_2Cl_2) R_f = 0.35; ^{1}H NMR (300 MHz, $CDCl_3$) δ 9.19 (s, 2H); ^{13}C NMR (125 MHz, $CDCl_3$) δ 151.8 (2 ×), 123.7.

2-1. General synthetic procedure and product characterization of 5-alkynyl-1,2,3-triazines (3a–3a'). $Pd(PPh_3)_2Cl_2$ (4 mol%, 28 mg, 0.04 equiv) and PPh_3 (2.5 mol%, 7 mg, 0.025 equiv) were added to a solution of 5-bromo-1,2,3-triazine **1** (1 mmol, 160 mg, 1 equiv) in wet THF (10 mL). After degassing for 10 min by bubbling with nitrogen, CuI (4 mol%, 7.62 mg, 0.04 equiv), triethylamine (3.6 mmol, 0.5 mL, 3.6 equiv) and substituted alkyne **2** (2 mmol, 2 equiv) were added under a nitrogen atmosphere. After complete consumption of the reactants in 1−24 h, as shown by TLC analysis, the reaction mixture was filtered through a Celite pad, the filtrate was washed with brine (10 mL), and then the aqueous layer was extracted with EtOAc (3 × 10 mL). The combined organic extracts were dried over anhydrous $MgSO_4$, filtered, and concentrated by a rotary evaporator. The crude product was purified by column chromatography on silica gel to afford the corresponding 5-alkynyl-1,2,3-triazine product (**3a–3a'**).

Table S1. Palladium-catalyzed Sonogashira coupling reactions of 5-bromo-1,2,3-triazine **1** with various terminal alkynes **2**[a]

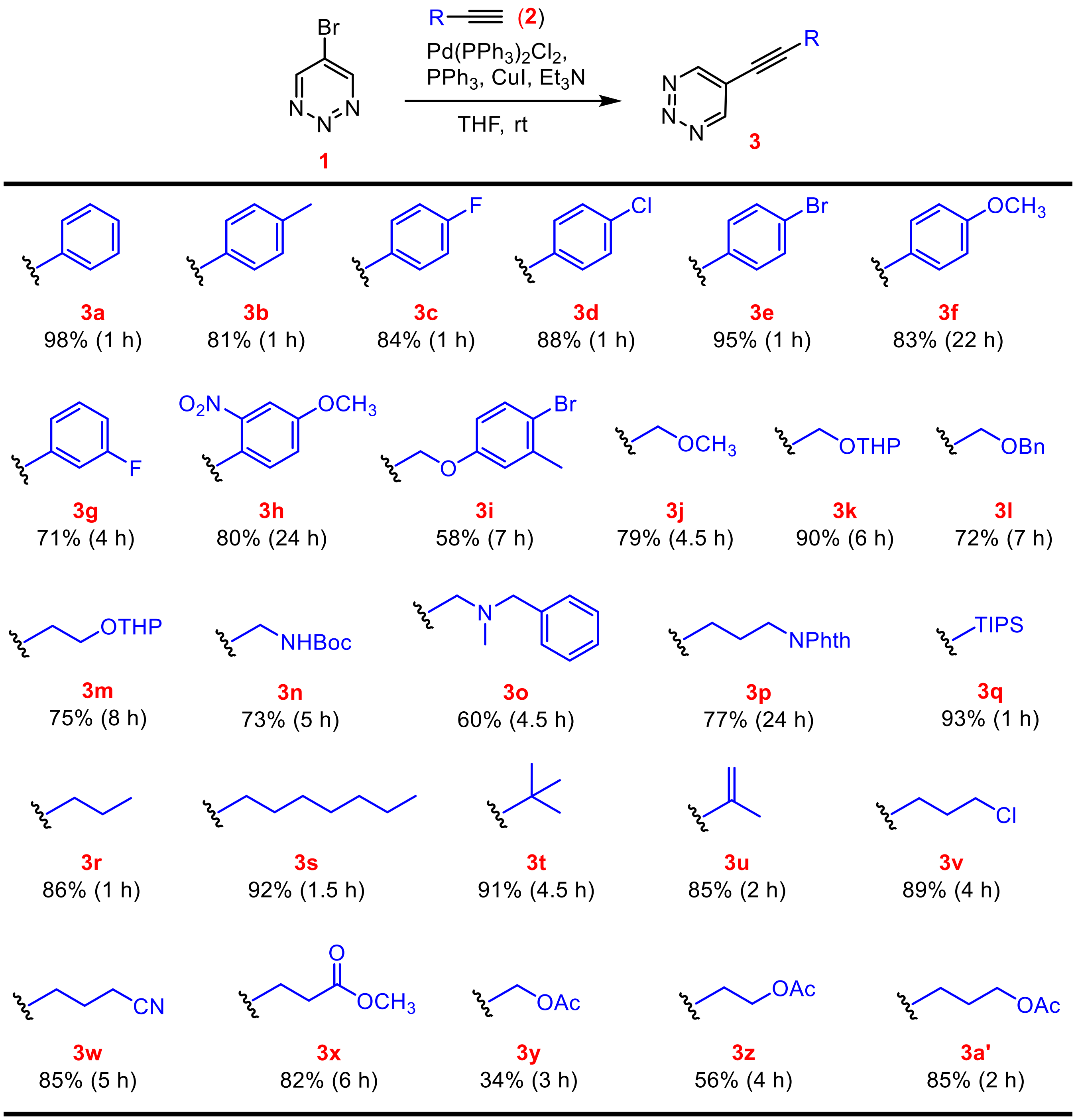

[a]All yields were determined by column chromatography purification.

5-Phenylethynyl-1,2,3-triazine (3a)

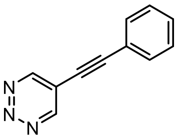

Compound **3a** was prepared from 5-bromo-1,2,3-triazine **1** (1 mmol, 160 mg) and phenylacetylene **2a** (2 mmol, 220 μL). The crude product was purified by column chromatography on silica gel (20% EtOAc in hexane) to afford the desired product **3a** (178 mg, 98%) as a brown solid. mp 118–121 °C; TLC (30% EtOAc in hexane) R_f = 0.55; IR ν_{max} (neat) 3051, 2225, 2033, 1549, 1489, 1448, 1356, 1163, 1070, 1032, 997, 916, 776, 755, 725 cm^{-1}; ^{1}H NMR (500 MHz, $CDCl_3$) δ 9.05 (s, 2H), 7.58–7.56 (m, 2H), 7.46–7.43 (m, 1H), 7.41–7.38 (m, 2H); ^{13}C{^{1}H} NMR (125 MHz, $CDCl_3$) δ 150.0, 132.1, 130.5, 128.7, 120.3, 117.2, 102.6, 80.5; HRMS (ESI) *m/z*: $[M+H]^+$ Calcd for $C_{11}H_8N_3$ 182.0718; Found 182.0719.

5-(*p*-Tolylethynyl)-1,2,3-triazine (3b)

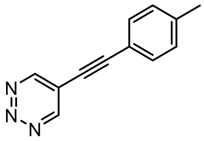

Compound **3b** was prepared from 5-bromo-1,2,3-triazine **1** (1 mmol, 160 mg) and 4-ethynyltoluene **2b** (2 mmol, 254 μL). The crude product was purified by column chromatography on silica gel (20% EtOAc in hexane) to afford the desired product **3b** (158 mg, 81%) as a brown solid. mp 140–143 °C; TLC (20% EtOAc in hexane) R_f = 0.45; IR ν_{max} (neat) 3058, 2983, 2205, 1599, 1467, 1539, 1411, 1321, 1253, 1200, 871, 758, 725, 665 cm^{-1}; ^{1}H NMR (500 MHz, $CDCl_3$) δ 9.05 (s, 2H), 7.47 (d, *J* = 8.1 Hz, 2H), 7.22 (d, *J* = 8.0 Hz, 2H), 2.39 (s, 3H); ^{13}C{^{1}H} NMR (125 MHz, $CDCl_3$) δ 150.1 (2 ×), 141.3, 132.2 (2 ×), 129.6 (2 ×), 117.6, 117.4, 103.3, 80.3, 21.7; HRMS (EI) *m/z*: $[M]^+$ Calcd for $C_{12}H_9N_3$ 195.0796; Found 195.0795.

5-[(4-Fluorophenyl)ethynyl]-1,2,3-triazine (3c)

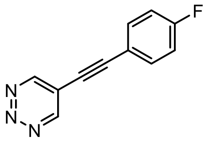

Compound **3c** was prepared from 5-bromo-1,2,3-triazine **1** (1 mmol, 160 mg) and 1-ethynyl-4-fluorobenzene **2c** (2 mmol, 230 μL). The crude product was purified by column chromatography on silica gel (20% EtOAc in hexane) to afford the desired product **3c** (167 mg, 84%) as a brown solid. mp 122–124 °C; TLC (20% EtOAc in hexane) R_f = 0.34; IR ν_{max} (neat) 3089, 2981, 2209, 1602, 1459, 1444, 1411, 1328, 1259, 1195, 879, 763, 711, 653 cm^{-1}; ^{1}H NMR (500 MHz, $CDCl_3$) δ 9.05 (s, 2H), 7.58 (qt, *J* = 4.7, 2.5 Hz, 2H), 7.11 (tt, *J* = 8.7, 2.3 Hz, 2H); ^{13}C{^{1}H} NMR (125 MHz, $CDCl_3$) δ 163.8 (J_{C-F} = 252.1 Hz), 150.1 (2 ×), 134.5 (J_{C-F} = 8.9

Hz, 2 ×), 117.2, 116.6 (J_{C-F} = 3.5 Hz), 116.3 (J_{C-F} = 22.3 Hz, 2 ×), 101.6, 80.5; HRMS (EI) *m/z*: $[M]^+$ Calcd for $C_{11}H_6FN_3$ 199.0546; Found 199.0546.

5-[(4-Chlorophenyl)ethynyl]-1,2,3-triazine (3d)

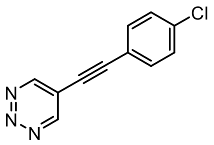

Compound **3d** was prepared from 5-bromo-1,2,3-triazine **1** (1 mmol, 160 mg) and 1-chloro-4-ethynylbenzene **2d** (2 mmol, 274 mg). The crude product was purified by column chromatography on silica gel (20% EtOAc in hexane) to afford the desired product **3d** (191 mg, 88%) as a brown solid. mp 142–145 °C; TLC (20% EtOAc in hexane) R_f = 0.32; IR ν_{max} (neat) 3056, 2990, 2214, 1612, 1467, 1417, 1329, 1129, 1095, 790, 711, 452 cm^{-1}; ^1H NMR (500 MHz, $CDCl_3$) δ 9.06 (s, 2H), 7.52 (dt, *J* = 8.5, 2.1 Hz, 2H), 7.39 (dt, *J* = 8.6, 2.1 Hz, 2H); $^{13}C\{^1H\}$ NMR (125 MHz, $CDCl_3$) δ 150.0 (2 ×), 137.0, 133.4 (2 ×), 129.2 (2 ×), 118.9, 117.0, 101.3, 81.5; HRMS (EI) *m/z*: $[M]^+$ Calcd for $C_{11}H_6ClN_3$ 215.0250; Found 215.0254.

5-[(4-Bromophenyl)ethynyl]-1,2,3-triazine (3e)

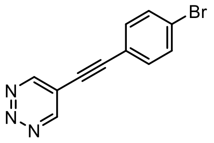

Compound **3e** was prepared from 5-bromo-1,2,3-triazine **1** (1 mmol, 160 mg) and 1-bromo-4-ethynylbenzene **2e** (2 mmol, 363 mg). The crude product was purified by column chromatography on silica gel (20% EtOAc in hexane) to afford the desired product **3e** (248 mg, 95%) as a brown solid. mp 158–160 °C; TLC (20% EtOAc in hexane) R_f = 0.37; IR ν_{max} (neat) 3056, 2920, 2223, 1696, 1466, 1403, 1313, 1118, 1077, 698, 352 cm^{-1}; ^1H NMR (500 MHz, $CDCl_3$) δ 9.06 (s, 2H), 7.56 (d, *J* = 7.3 Hz, 2H), 7.44 (d, *J* = 7.1 Hz, 2H); $^{13}C\{^1H\}$ NMR (125 MHz, $CDCl_3$) δ 150.0 (2 ×), 133.6 (2 ×), 132.2 (2 ×), 125.4, 119.3, 117.0, 101.4, 81.6; HRMS (EI) *m/z*: $[M]^+$ Calcd for $C_{11}H_6BrN_3$ 258.9745; Found 258.9738.

5-[(4-Methoxyphenyl)ethynyl]-1,2,3-triazine (3f)

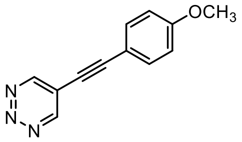

Compound **3f** was prepared from 5-bromo-1,2,3-triazine **1** (1 mmol, 160 mg) and 4-ethynylanisole **2f** (2 mmol, 260 μL). The crude product was purified by column chromatography on silica gel (10% EtOAc in hexane to 20% EtOAc in hexane) to afford the desired product **3f** (175 mg, 83%) as a brown solid. mp 145–148 °C; TLC (20% EtOAc in hexane) R_f = 0.20; IR ν_{max} (neat) 3101, 2934, 2209, 1656, 1493, 1328, 1255, 1068, 739 cm^{-1}; ^{1}H NMR (500 MHz, $CDCl_3$) δ 9.03 (s, 2H), 7.52 (d, J = 8.9 Hz, 2H), 6.92 (d, J = 8.9 Hz, 2H), 3.84 (s, 3H); ^{13}C{^{1}H} NMR (125 MHz, $CDCl_3$) δ 161.5, 150.0 (2 ×), 134.1 (2 ×), 117.7, 114.5 (2 ×), 112.4, 103.5, 80.0, 55.4; HRMS (ESI) *m/z*: [M+H]$^+$ Calcd for $C_{12}H_{10}N_3O$ 212.0824; Found 212.0821.

5-[(3-Fluorophenyl)ethynyl]-1,2,3-triazine (3g)

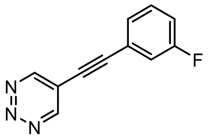

Compound **3g** was prepared from 5-bromo-1,2,3-triazine **1** (1 mmol, 160 mg) and 1-ethynyl-3-fluorobenzene **2g** (2 mmol, 230 μL). The crude product was purified by column chromatography on silica gel (20% EtOAc in hexane) to afford the desired product **3g** (141 mg, 71%) as a brown solid. mp 100–101 °C; TLC (40% EtOAc in hexane) R_f = 0.50; IR ν_{max} (neat) 2214, 1606, 1578, 1552, 1484, 1429, 1217, 1129, 1037, 937, 875, 862, 819, 791, 706 cm^{-1}; ^{1}H NMR (500 MHz, $CDCl_3$) δ 9.07 (s, 2H), 7.41–7.36 (m, 2H), 7.29–7.27 (m, 1H), 7.20–7.15 (m, 1H); ^{13}C{^{1}H} NMR (125 MHz, $CDCl_3$) δ 162.0 ($J_{C\text{-}F}$ = 246.3 Hz), 149.9 (2 ×), 130.3 ($J_{C\text{-}F}$ = 8.4 Hz), 128.0, 122.0 ($J_{C\text{-}F}$ = 9.3 Hz), 118.7 ($J_{C\text{-}F}$ = 23.1 Hz), 117.8 ($J_{C\text{-}F}$ = 2.5 Hz), 116.6, 100.6, 81.0; ^{19}F{^{1}H} NMR (470 MHz, $CDCl_3$) δ –111.4; HRMS (EI) *m/z*: [M]$^+$ Calcd for $C_{11}H_6FN_3$ 199.0546; Found 199.0539.

5-[(4-Methoxy-2-nitrophenyl)ethynyl]-1,2,3-triazine (3h)

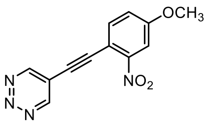

Compound **3h** was prepared from 5-bromo-1,2,3-triazine **1** (1 mmol, 160 mg) and 1-ethynyl-4-methoxy-2-nitrobenzene **2h** (2 mmol, 355 mg). The crude product was purified by column chromatography on silica gel (40% EtOAc in hexane) to afford the desired product **3h** (206 mg, 80%) as a brown solid. mp 142–144 °C;

TLC (40% EtOAc in hexane) R_f = 0.24; IR ν_{max} (neat) 3046, 2921, 2216, 1604, 1491, 1331, 1255, 1068, 728 cm^{-1}; ^{1}H NMR (600 MHz, $CDCl_3$) δ 9.09 (s, 2H), 7.68 (dd, *J* = 5.6, 3.0 Hz, 2H), 7.20 (dd, *J* = 8.7, 2.6 Hz, 1H), 3.94 (s, 3H); $^{13}C\{^{1}H\}$ NMR (150 MHz, $CDCl_3$) δ 161.4, 151.0, 150.0 (2 ×), 136.2, 119.9 (2 ×), 116.9, 110.3, 107.8, 97.7, 85.5, 56.3; HRMS (EI) *m/z*: $[M]^+$ Calcd for $C_{12}H_8N_4O_3$ 256.0596; Found 256.0591.

5-[3-(4-Bromo-3-methylphenoxy)prop-1-yn-1-yl]-1,2,3-triazine (3i)

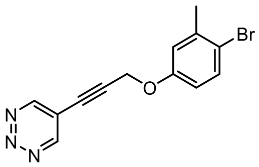

Compound **3i** was prepared from 5-bromo-1,2,3-triazine **1** (1 mmol, 160 mg) and 1-bromo-2-methyl-4-(prop-2-yn-1-yloxy)benzene **2i** (2 mmol, 452 mg). The crude product was purified by column chromatography on silica gel (20% EtOAc in hexane) to afford the desired product **3i** (175 mg, 58%) as a brown solid. mp 111–114 °C; TLC (20% EtOAc in hexane) R_f = 0.22; IR ν_{max} (neat) 3039, 2904, 2221, 1624, 1488, 1356, 1255, 1072, 698 cm^{-1}; ^{1}H NMR (500 MHz, $CDCl_3$) δ 8.98 (s, 2H), 7.44 (d, *J* = 8.8 Hz, 1H), 6.86 (d, *J* = 3.1 Hz, 1H), 6.69 (dd, *J* = 8.8, 3.1 Hz, 1H), 4.93 (s, 2H), 2.37 (s, 3H); $^{13}C\{^{1}H\}$ NMR (125 MHz, $CDCl_3$) δ 156.3, 150.3 (2 ×), 139.4, 133.1, 117.6, 117.1, 116.1, 113.6, 97.3, 78.6, 56.2, 23.2; HRMS (ESI) *m/z*: $[M+H]^+$ Calcd for $C_{13}H_{11}BrN_3O$ 304.0085; Found 304.0081.

5-(3-Methoxyprop-1-yn-1-yl)-1,2,3-triazine (3j)

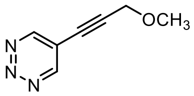

Compound **3j** was prepared from 5-bromo-1,2,3-triazine **1** (1 mmol, 160 mg) and methyl propargyl ether **2j** (2 mmol, 169 μL). The crude product was purified by column chromatography on silica gel (40% EtOAc in hexane) to afford the desired product **3j** (117 mg, 79%) as a brown solid. mp 93–95 °C; TLC (40% EtOAc in hexane) R_f = 0.26; IR ν_{max} (neat) 3042, 2857, 2209, 1556, 1494, 1376, 1256, 1027, 727 cm^{-1}; ^{1}H NMR (500 MHz, $CDCl_3$) δ 8.99 (s, 2H), 4.37 (s, 2H), 3.45 (s, 3H); $^{13}C\{^{1}H\}$ NMR (125 MHz, $CDCl_3$) δ 150.4 (2 ×), 116.6, 99.1, 77.7, 60.1, 58.4; HRMS (EI) *m/z*: $[M]^+$ Calcd for $C_7H_7N_3O$ 149.0589; Found 149.0586.

5-{3-[(Tetrahydro-2*H*-pyran-2-yl)oxy]prop-1-yn-1-yl}-1,2,3-triazine (3k)

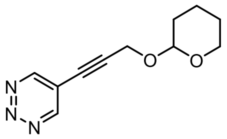

Compound **3k** was prepared from 5-bromo-1,2,3-triazine **1** (1 mmol, 160 mg) and tetrahydro-2-(2-propynyloxy)-2*H*-pyran **2k** (2 mmol, 282 µL). The crude product was purified by column chromatography on silica gel (40% EtOAc in hexane) to afford the desired product **3k** (198 mg, 90%) as a brown syrup. TLC (40% EtOAc in hexane) R_f = 0.37; IR ν_{max} (neat) 3062, 2927, 2210, 1671, 1487, 1287, 1125, 750 cm^{-1}; ^{1}H NMR (500 MHz, $CDCl_3$) δ 8.98 (s, 2H), 4.80 (t, *J* = 3.3 Hz, 1H), 4.51 (q, *J* = 15.8 Hz, 2H), 3.85–3.80 (m, 1H), 3.55 (dtd, *J* = 11.2, 4.2, 1.5 Hz, 1H), 1.84–1.79 (m, 1H), 1.77–1.71 (m, 1H), 1.66–1.60 (m, 2H), 1.58–1.52 (m, 2H); ^{13}C{^{1}H} NMR (125 MHz, $CDCl_3$) δ 150.5 (2 ×), 116.7, 99.6, 97.6, 77.2, 62.1, 54.5, 30.1, 25.2, 18.8; HRMS (ESI) *m/z*: [M+H]$^+$ Calcd for $C_{11}H_{14}N_3O_2$ 220.1086; Found 220.1087.

5-[3-(Benzyloxy)prop-1-yn-1-yl]-1,2,3-triazine (3l)

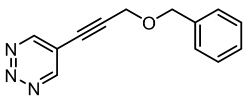

Compound **3l** was prepared from 5-bromo-1,2,3-triazine **1** (1 mmol, 160 mg) and benzyl propargyl ether **2l** (2 mmol, 289 µL). The crude product was purified by column chromatography on silica gel (20% EtOAc in hexane to 40% EtOAc in hexane) to afford the desired product **3l** (163 mg, 72%) as a brown syrup. TLC (20% EtOAc in hexane) R_f = 0.14; IR ν_{max} (neat) 3049, 2918, 2109, 1689, 1456, 1250, 1098, 789 cm^{-1}; ^{1}H NMR (500 MHz, $CDCl_3$) δ 8.97 (s, 2H), 7.37–7.36 (m, 1H), 7.36 (s, 2H), 7.35–7.34 (m, 1H), 7.33–7.30 (m, 1H), 4.65 (s, 2H), 4.44 (s, 2H); ^{13}C{^{1}H} NMR (125 MHz, $CDCl_3$) δ 150.4 (2 ×), 136.7, 128.6 (2 ×), 128.3, 128.1 (2 ×), 116.6, 99.3, 77.8, 72.7, 57.6; HRMS (ESI) *m/z*: [M+H]$^+$ Calcd for $C_{13}H_{12}N_3O$ 226.0980; Found 226.0974.

5-{4-[(Tetrahydro-2*H*-pyran-2-yl)oxy]but-1-yn-1-yl}-1,2,3-triazine (3m)

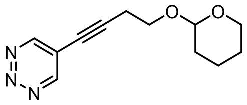

Compound **3m** was prepared from 5-bromo-1,2,3-triazine **1** (1 mmol, 160 mg) and 2-(3-butynyloxy) tetrahydro-2*H*-pyran **2m** (2 mmol, 314 µL). The crude product was purified by column chromatography on silica gel (40% EtOAc in hexane) to afford the desired product **3m** (175 mg, 75%) as a brown syrup. TLC

(40% EtOAc in hexane) R_f = 0.29; IR ν_{max} (neat) 3052, 2938, 2201, 1659, 1500, 1263, 1137, 791 cm^{-1}; ^{1}H NMR (500 MHz, $CDCl_3$) δ 8.93 (s, 2H), 4.64 (t, *J* = 3.6 Hz, 1H), 3.91 (dt, *J* = 9.7, 6.7 Hz, 1H), 3.85 (qd, *J* = 6.5, 3.0 Hz, 1H), 3.64 (dt, *J* = 9.7, 6.7 Hz, 1H), 3.53–3.49 (m, 1H), 2.80 (t, *J* = 6.7 Hz, 2H), 1.82–1.78 (m, 1H), 1.71–1.68 (m, 1H), 1.61–1.56 (m, 2H), 1.54–1.49 (m, 2H); ^{13}C{^{1}H} NMR (125 MHz, $CDCl_3$) δ 150.7 (2 ×), 117.7, 102.6, 98.9, 73.4, 64.7, 62.4, 30.5, 25.3, 21.4, 19.3; HRMS (ESI) *m/z*: [M+H]$^+$ Calcd for $C_{12}H_{15}N_3O_2$ 234.1243; Found 234.1244.

tert-Butyl [3-(1,2,3-triazin-5-yl)prop-2-yn-1-yl] carbamate (3n)

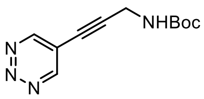

Compound **3n** was prepared from 5-bromo-1,2,3-triazine **1** (1 mmol, 160 mg) and *tert*-butylprop-2-yn-1-ylcarbamate **2n** (2 mmol, 311 µL). The crude product was purified by column chromatography on silica gel (40% EtOAc in hexane) to afford the desired product **3n** (172 mg, 73%) as a brown syrup. TLC (40% EtOAc in hexane) R_f = 0.27; IR ν_{max} (neat) 3359, 3120, 2928, 2207, 1765, 1658, 1499, 1263, 1137, 1027, 689 cm^{-1}; ^{1}H NMR (600 MHz, $CDCl_3$) δ 8.96 (s, 2H), 4.88 (s, 1H), 4.22 (d, *J* = 5.0 Hz, 2H), 1.45 (s, 9H); ^{13}C{^{1}H} NMR (150 MHz, $CDCl_3$) δ 155.1, 150.5 (2 ×), 116.8, 99.9, 80.7, 74.5, 31.1, 28.3 (3 ×); HRMS (ESI) *m/z*: [M+H]$^+$ Calcd for $C_{11}H_{15}N_4O_2$ 235.1195; Found 235.1199.

***N*-Benzyl-*N*-methyl-3-(1,2,3-triazin-5-yl) prop-2-yn-1-amine (3o)**

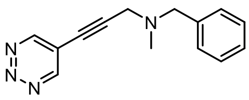

Compound **3o** was prepared from 5-bromo-1,2,3-triazine **1** (1 mmol, 160 mg) and *N*-methyl-*N*-propargylbenzylamine **2o** (2 mmol, 338 µL). The crude product was purified by column chromatography on silica gel (40% EtOAc in hexane) to afford the desired product **3o** (145 mg, 60%) as a brown syrup. TLC (40% EtOAc in hexane) R_f = 0.25; IR ν_{max} (neat) 3056, 2953, 2217, 1637, 1499, 1230, 1030, 724 cm^{-1}; ^{1}H NMR (500 MHz, $CDCl_3$) δ 8.91 (s, 2H), 7.25 (br s, 3H), 7.22–7.17 (m, 2H), 3.54 (s, 2H), 3.51 (s, 2H), 2.33 (s, 3H); ^{13}C{^{1}H} NMR (125 MHz, $CDCl_3$) δ 150.6 (2 ×), 137.7, 129.0 (2 ×), 128.5 (2 ×), 127.5 (2 ×), 117.1, 99.7, 60.5, 45.8, 42.1; HRMS (ESI) *m/z*: [M+H]$^+$ Calcd for $C_{14}H_{15}N_4$ 239.1297; Found 239.1300.

2-[5-(1,2,3-Triazin-5-yl)pent-4-yn-1-yl] isoindoline-1,3-dione (3p)

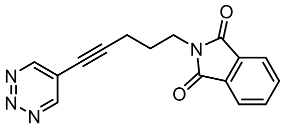

Compound **3p** was prepared from 5-bromo-1,2,3-triazine **1** (1 mmol, 160 mg) and *N*-(4-pentynyl)phthalimide **2p** (2 mmol, 427 mg). The crude product was purified by column chromatography on silica gel (40% EtOAc in hexane) to afford the desired product **3p** (227 mg, 77%) as a yellowish brown solid. mp 136–139 °C; TLC (40% EtOAc in hexane) R_f = 0.18; IR ν_{max} (neat) 3091, 2954, 2210, 1788, 1643, 1510, 1227, 1032, 789 cm^{-1}; ^{1}H NMR (500 MHz, $CDCl_3$) δ 8.76 (s, 2H), 7.79 (dd, *J* = 5.4, 3.1 Hz, 2H), 7.66 (dd, *J* = 5.4, 3.0 Hz, 2H), 3.85 (t, *J* = 6.7 Hz, 2H), 2.59 (t, *J* = 6.8 Hz, 2H), 2.07 (quintet, *J* = 6.7 Hz, 2H); ^{13}C{^{1}H} NMR (125 MHz, $CDCl_3$) δ 168.3 (2 ×), 150.4 (2 ×), 134.2 (2 ×), 131.9 (2 ×), 123.3 (2 ×), 117.4, 104.0, 73.3, 37.1, 26.4, 17.7; HRMS (ESI) *m/z*: [M+H]$^+$ Calcd for $C_{16}H_{13}N_4O_2$ 293.1033; Found 293.1029.

5-[(Triisopropylsilyl)ethynyl]-1,2,3-triazine (3q)

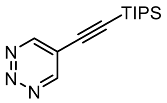

Compound **3q** was prepared from 5-bromo-1,2,3-triazine **1** (1 mmol, 160 mg) and triisopropylsilyl acetynlene **2q** (2 mmol, 447 µL). The crude product was purified by column chromatography on silica gel (20% EtOAc in hexane) to afford the desired product **3q** (243 mg, 93%) as a brown syrup; TLC (40% EtOAc in hexane) R_f = 0.75; IR ν_{max} (neat) 2943, 2865, 1551, 1463, 1346, 1249, 997, 920, 882, 845 cm^{-1}; ^{1}H NMR (500 MHz, $CDCl_3$) δ 8.98 (s, 2H), 1.56 (s, 3H), 1.12 (d, *J* = 6.0 Hz, 18H); ^{13}C{^{1}H} NMR (125 MHz, $CDCl_3$) δ 150.6 (2 ×), 117.0, 108.4, 97.3, 18.5 (3 ×), 11.0 (6 ×); HRMS (EI) *m/z*: [M]$^+$ Calcd for $C_{14}H_{23}N_3Si$ 261.1661; Found 261.1661.

5-(Pentyn-1-yl)-1,2,3-triazine (3r)

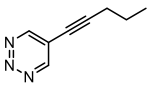

Compound **3r** was prepared from 5-bromo-1,2,3-triazine **1** (1 mmol, 160 mg) and 1-pentyne **2r** (2 mmol, 198 µL). The crude product was purified by column chromatography on silica gel (20% EtOAc in hexane) to afford the desired product **3r** (127 mg, 86%) as a brown syrup. TLC (20% EtOAc in hexane) R_f = 0.28; IR ν_{max} (neat) 3062, 2956, 2201, 1689, 1471, 1332, 727 cm^{-1}; ^{1}H NMR (500 MHz, $CDCl_3$) δ 8.93 (s, 2H), 2.47 (t, *J* = 7.0

Hz, 2H), 1.66 (sixlet, J = 7.3 Hz, 2H), 1.04 (t, J = 7.4 Hz, 3H); $^{13}C\{^1H\}$ NMR (125 MHz, $CDCl_3$) δ 150.7 (2 ×), 118.0, 105.7, 72.9, 21.7, 21.5, 13.5; HRMS (EI) *m/z*: $[M]^+$ Calcd for $C_8H_9N_3$ 147.0796; Found 147.0796.

5-(Non-1-yn-1-yl)-1,2,3-triazine (3s)

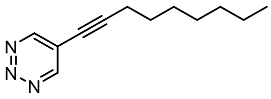

Compound **3s** was prepared from 5-bromo-1,2,3-triazine **1** (1 mmol, 160 mg) and 1-nonyne **2s** (2 mmol, 328 μL). The crude product was purified by column chromatography on silica gel (20% EtOAc in hexane) to afford the desired product **3s** (187 mg, 92%) as a brown syrup. TLC (20% EtOAc in hexane) R_f = 0.53; IR ν_{max} (neat) 3067, 2924, 2205, 1685, 1437, 1382, 734 cm^{-1}; ^1H NMR (500 MHz, $CDCl_3$) δ 8.90 (s, 2H), 2.45 (t, J = 7.2 Hz, 2H), 1.59 (quintet, J = 7.4 Hz, 2H), 1.38 (quintet, J = 7.3 Hz, 2H), 1.30–1.24 (m, 6H), 0.83 (t, J = 6.7 Hz, 3H); $^{13}C\{^1H\}$ NMR (125 MHz, $CDCl_3$) δ 150.7 (2 ×), 118.0, 105.9, 72.7, 31.6, 28.8, 28.6, 27.9, 22.5, 19.8, 14.0; HRMS (EI) *m/z*: $[M]^+$ Calcd for $C_{12}H_{17}N_3$ 203.1422; Found 203.1418.

5-(3,3-Dimethylbutyn-1-yl)-1,2,3-triazine (3t)

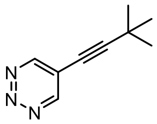

Compound **3t** was prepared from 5-bromo-1,2,3-triazine **1** (1 mmol, 160 mg) and 3,3-dimethyl-1-butyne **2t** (2 mmol, 247 μL). The crude product was purified by column chromatography on silica gel (20% EtOAc in hexane) to afford the desired product **3t** (146 mg, 91%) as an orange solid. mp 57–60 °C; TLC (20% EtOAc in hexane) R_f = 0.40; IR ν_{max} (neat) 3056, 2920, 2207, 1682, 1451, 1364, 735 cm^{-1}; ^1H NMR (500 MHz, $CDCl_3$) δ 8.92 (s, 2H), 1.33 (s, 9H); $^{13}C\{^1H\}$ NMR (125 MHz, $CDCl_3$) δ 150.7 (2 ×), 118.0, 113.2, 71.4, 30.3 (3 ×), 28.6; HRMS (EI) *m/z*: $[M]^+$ Calcd for $C_9H_{11}N_3$ 161.0953; Found 161.0950.

5-(3-Methylbut-3-en-1-yn-1-yl)-1,2,3-triazine (3u)

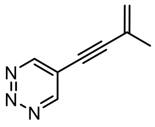

Compound **3u** was prepared from 5-bromo-1,2,3-triazine **1** (1 mmol, 160 mg) and 2-methyl-1-buten-3-yne **2u** (2 mmol, 191 μL). The crude product was purified by column chromatography on silica gel (20% EtOAc in hexane) to afford the desired product **3u** (123 mg, 85%) as a brown solid. mp 67–70 °C; TLC (20% EtOAc in hexane) R_f = 0.37; IR ν_{max} (neat) 3081, 2938, 2203, 1946, 1680, 981, 798 cm^{-1}; ^{1}H NMR (500 MHz, $CDCl_3$) δ 8.97 (s, 2H), 5.60 (t, *J* = 1.1 Hz, 1H), 5.53 (quintet, *J* = 1.6 Hz, 1H), 2.00 (t, *J* = 1.3 Hz, 3H); ^{13}C{^{1}H} NMR (125 MHz, $CDCl_3$) δ 150.2 (2 ×), 126.9, 125.1, 117.3, 103.6, 79.5, 22.6; HRMS (EI) *m/z*: $[M]^+$ Calcd for $C_8H_7N_3$ 145.0640; Found 145.0637.

5-(5-Chloropent-1-yn-1-yl)-1,2,3-triazine (3v)

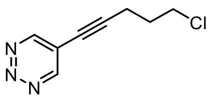

Compound **3v** was prepared from 5-bromo-1,2,3-triazine **1** (1 mmol, 160 mg) and 5-chloro-1-pentyne **2v** (2 mmol, 212 μL). The crude product was purified by column chromatography on silica gel (30% EtOAc in hexane) to afford the desired product **3v** (162 mg, 89%) as a brown syrup. TLC (30% EtOAc in hexane) R_f = 0.34; IR ν_{max} (neat) 3048, 2910, 2201, 1657, 1471, 1384, 1098, 801, 720 cm^{-1}; ^{1}H NMR (500 MHz, $CDCl_3$) δ 8.93 (s, 2H), 3.66 (t, *J* = 6.2 Hz, 2H), 2.71 (t, *J* = 7.0 Hz, 2H), 2.09 (quintet, *J* = 6.6 Hz, 2H); ^{13}C{^{1}H} NMR (125 MHz, $CDCl_3$) δ 150.6 (2 ×), 117.5, 103.3, 73.5, 43.2, 30.5, 17.2; HRMS (EI) *m/z*: $[M]^+$ Calcd for $C_8H_8ClN_3$ 181.0407; Found 181.0405.

6-(1,2,3-Triazin-5-yl) hex-5-ynenitrile (3w)

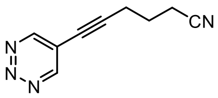

Compound **3w** was prepared from 5-bromo-1,2,3-triazine **1** (1 mmol, 160 mg) and 5-hexynenitrile **2w** (2 mmol, 210 μL). The crude product was purified by column chromatography on silica gel (20% EtOAc in hexane to 50% EtOAc in hexane) to afford the desired product **3w** (146 mg, 85%) as a brown solid. mp 58–63 °C; TLC (40% EtOAc in hexane) R_f = 0.24; IR ν_{max} (neat) 3063, 2919, 2235, 1676, 1423, 1329, 729 cm^{-1}; ^{1}H NMR (500 MHz, $CDCl_3$) δ 8.96 (s, 2H), 2.72 (t, *J* = 6.9 Hz, 2H), 2.54 (t, *J* = 7.0 Hz, 2H), 2.01 (quintet, *J* = 7.0 Hz, 2H); ^{13}C{^{1}H} NMR (125 MHz, $CDCl_3$) δ 150.6 (2 ×), 118.4, 117.1, 101.7, 74.3, 23.8, 18.9, 16.4; HRMS (ESI) *m/z*: $[M+H]^+$ Calcd for $C_9H_9N_4$ 173.0827; Found 173.0833.

Methyl 5-(1,2,3-triazin-5-yl) pent-4-ynoate (3x)

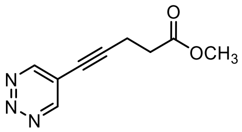

Compound **3x** was prepared from 5-bromo-1,2,3-triazine **1** (1 mmol, 160 mg) and methyl pent-4-ynoate **2x** (2 mmol, 225 mg). The crude product was purified by column chromatography on silica gel (10% EtOAc in hexane to 30% EtOAc in hexane) to afford the desired product **3x** (158 mg, 82%) as a brown solid. mp 50–53 °C; TLC (30% EtOAc in hexane) R_f = 0.24; IR ν_{max} (neat) 3052, 2956, 2219, 1799, 1728, 1649, 1456, 1339, 729 cm^{-1}; ^{1}H NMR (500 MHz, $CDCl_3$) δ 8.93 (s, 2H), 3.72 (s, 3H), 2.82 (t, J = 7.2 Hz, 2H), 2.66 (t, J = 7.2 Hz, 2H); ^{13}C{^{1}H} NMR (125 MHz, $CDCl_3$) δ 171.6, 150.7 (2 ×), 117.5, 103.0, 73.2, 52.1, 32.3, 15.6; HRMS (EI) *m/z*: $[M]^+$ Calcd for $C_9H_9N_3O_2$ 191.0695; Found 191.0700.

3-(1,2,3-Triazin-5-yl) prop-2-yn-1-yl acetate (3y)

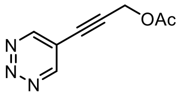

Compound **3y** was prepared from 5-bromo-1,2,3-triazine **1** (1.5 mmol, 240 mg) and prop-2-yn-1-yl acetate **2y** (3 mmol, 294 mg). The crude product was purified by column chromatography on silica gel (40% EtOAc in hexane) to afford the desired product **3y** (90 mg, 34%) as a brown solid. $C_8H_7N_3O_2$; mp 69–71 °C; TLC (40% EtOAc in hexane) R_f = 0.35; IR ν_{max} (neat) 3205, 1736, 1564, 1497, 1452, 1377, 1360, 1220, 1076, 1031, 958, 934, 912, 762, 670, 632, 597 cm^{-1}; ^{1}H NMR (500 MHz, $CDCl_3$) δ 8.99 (s, 2H), 4.92 (s, 2H), 2.12 (s, 3H); ^{13}C{^{1}H} NMR (125 MHz, $CDCl_3$) δ 169.9, 150.3 (2 ×), 116.2, 96.7, 77.6, 51.8, 20.5; HRMS (FAB) *m/z*: $[M+H]^+$ Calcd for $C_8H_8N_3O_2$ 178.0617; Found 178.0617.

4-(1,2,3-Triazin-5-yl) but-3-yn-1-yl acetate (3z)

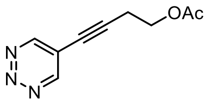

Compound **3z** was prepared from 5-bromo-1,2,3-triazine **1** (1 mmol, 160 mg) and but-3-yn-1-yl acetate **2z** (2 mmol, 225 mg). The crude product was purified by column chromatography on silica gel (40% EtOAc in hexane) to afford the desired product **3z** (107 mg, 56%) as a brown solid. $C_9H_9N_3O_2$; mp 47–50 °C; TLC (40% EtOAc in hexane) R_f = 0.37; IR ν_{max} (neat) 2232, 1732, 1557, 1360, 1240, 1039, 933, 665 cm^{-1}; ^{1}H NMR (500

MHz, $CDCl_3$) δ 8.95 (s, 2H), 4.26 (t, *J* = 7.0 Hz, 2H), 2.84 (t, *J* = 6.5 Hz, 2H), 2.08 (s, 3H); $^{13}C\{^1H\}$ NMR (125 MHz, $CDCl_3$) δ 170.7, 150.6 (2 ×), 117.3, 100.6, 73.9, 61.2, 20.8, 20.3; HRMS (FAB) *m/z*: $[M+H]^+$ Calcd for $C_9H_{10}N_3O_2$ 192.0773; Found 192.0774.

5-(1,2,3-Triazin-5-yl) pent-4-yn-1-yl acetate (3a')

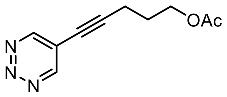

Compound **3a'** was prepared from 5-bromo-1,2,3-triazine **1** (1 mmol, 160 mg) and pent-4-yn-1-yl acetate **2a'** (2 mmol, 253 mg). The crude product was purified by column chromatography on silica gel (40% EtOAc in hexane) to afford the desired product **3a'** (175 mg, 85%) as a brown syrup. TLC (40% EtOAc in hexane) R_f = 0.37; IR ν_{max} (neat) 2230, 1733, 1558, 1366, 1238, 1041, 665 cm^{-1}; ^1H NMR (500 MHz, $CDCl_3$) δ 8.93 (s, 2H), 4.18 (t, *J* = 6.5 Hz, 2H), 2.60 (t, *J* = 7.5 Hz, 2H), 2.04 (s, 3H), 1.97 (quintet, *J* = 7.0 Hz, 2H); $^{13}C\{^1H\}$ NMR (125 MHz, $CDCl_3$) δ 170.9, 150.6 (2 ×), 117.6, 103.8, 73.3, 62.6, 27.1, 20.9, 16.6; HRMS (FAB) *m/z*: $[M+H]^+$ Calcd for $C_{10}H_{12}N_3O_2$ 206.0930; Found 206.0924.

4. Reference

S1. Wu, C.-C.; Ambre, R.; Lee, M.-H.; Shie, J.-J. Flexible Construction Approach to the Synthesis of 1,5-Substituted Pyrrole-3-Carbaldehydes from 5-Bromo-1,2,3-Triazine. *Org. Lett.* **2022**, *24*, 2889–2893.

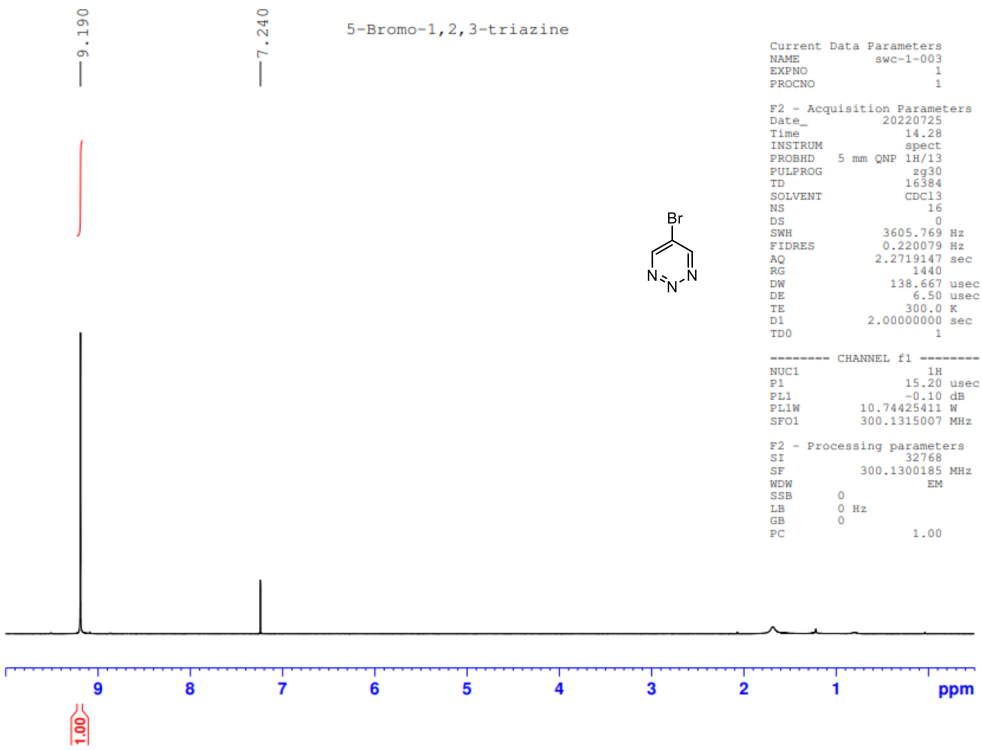

^{1}H NMR spectrum of **1** ($CDCl_3$, 300 MHz)

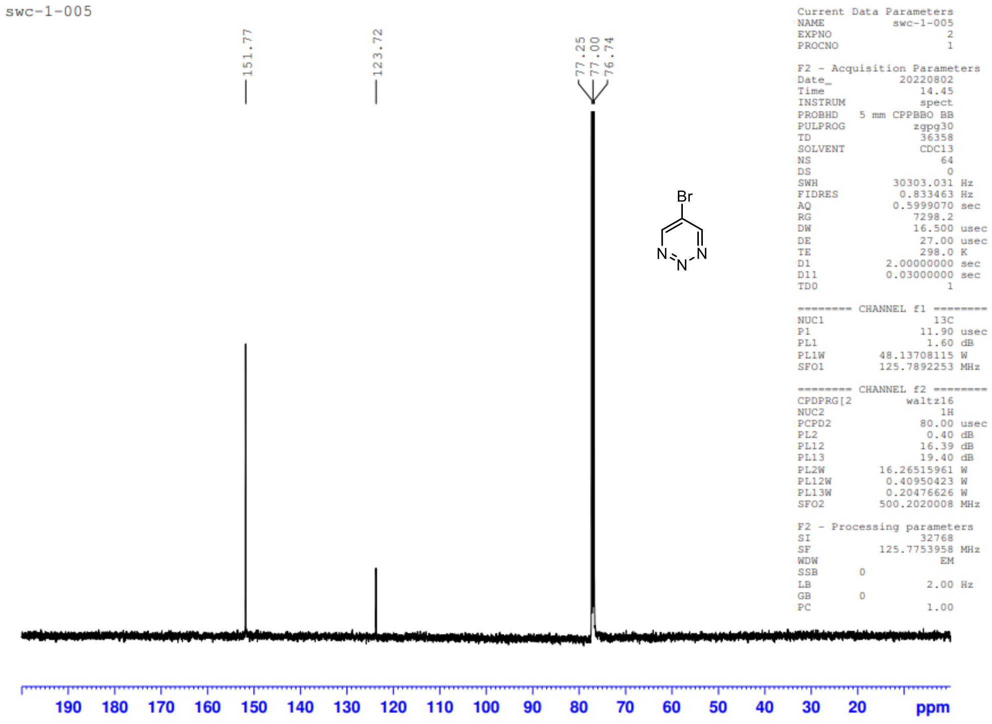

^{13}C{^{1}H} NMR spectrum of **1** ($CDCl_3$, 125 MHz)

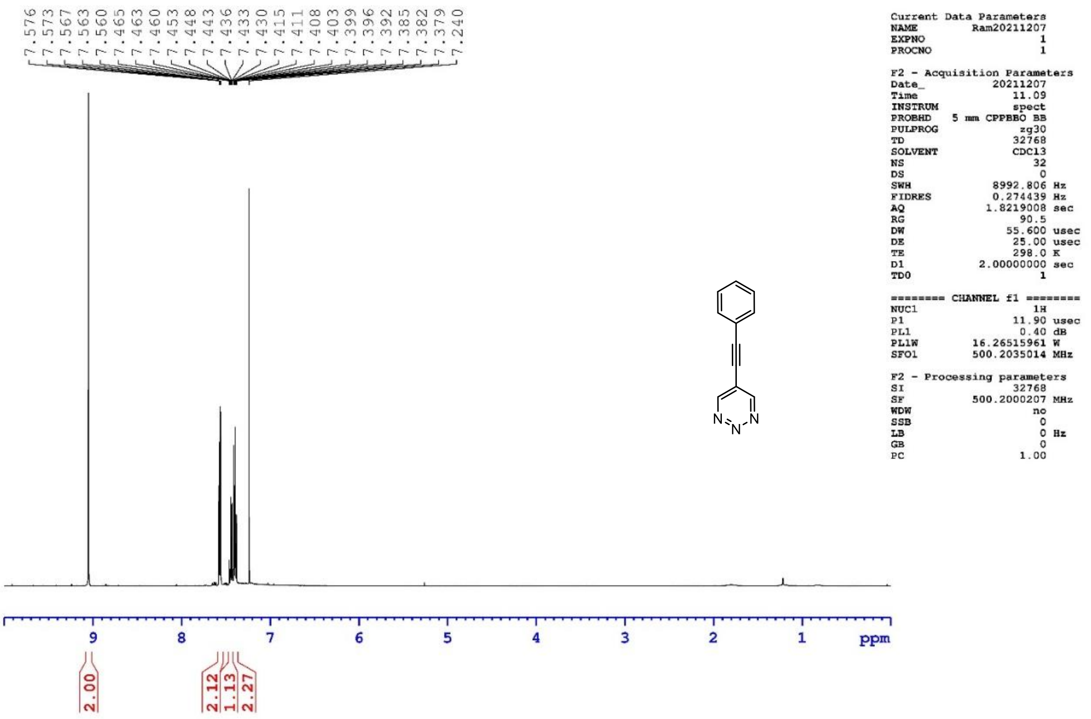

^{1}H NMR spectrum of **3a** ($CDCl_3$, 500 MHz)

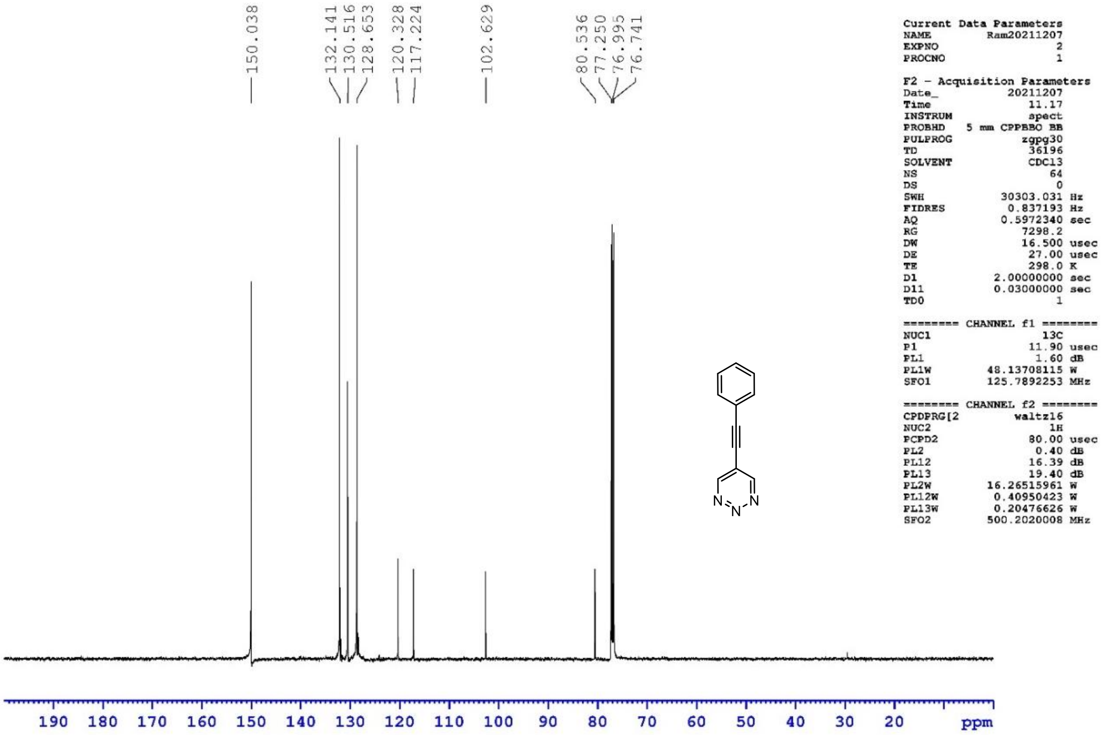

^{13}C{^{1}H} NMR spectrum of **3a** ($CDCl_3$, 125 MHz)

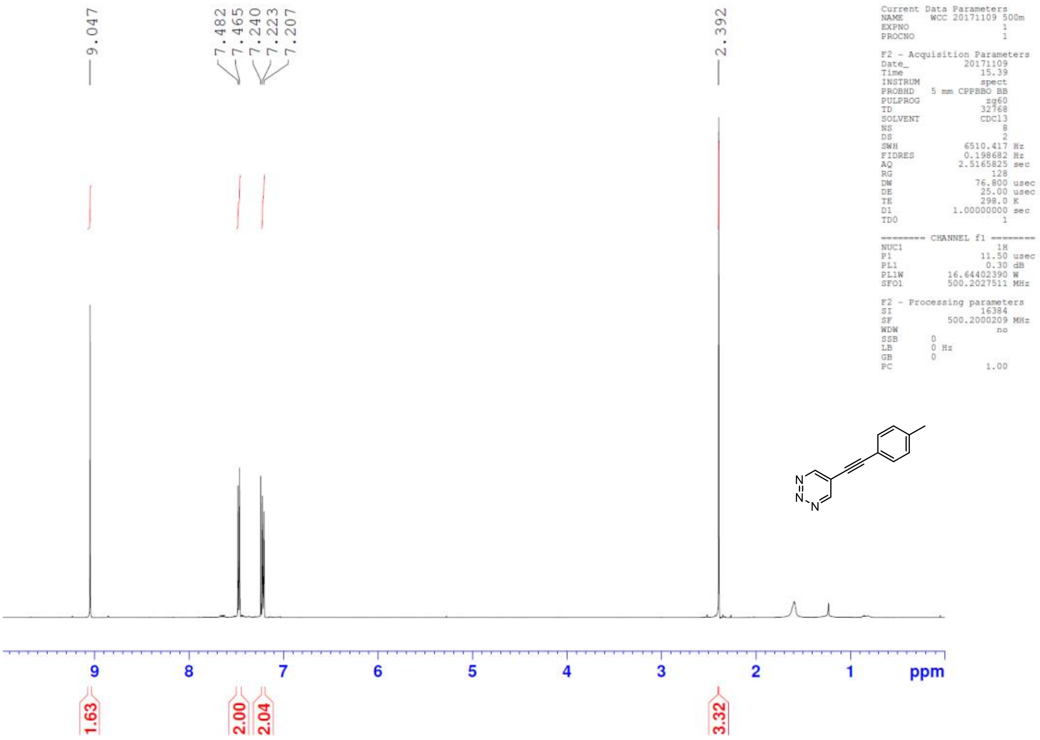

^{1}H NMR spectrum of **3b** ($CDCl_3$, 500 MHz)

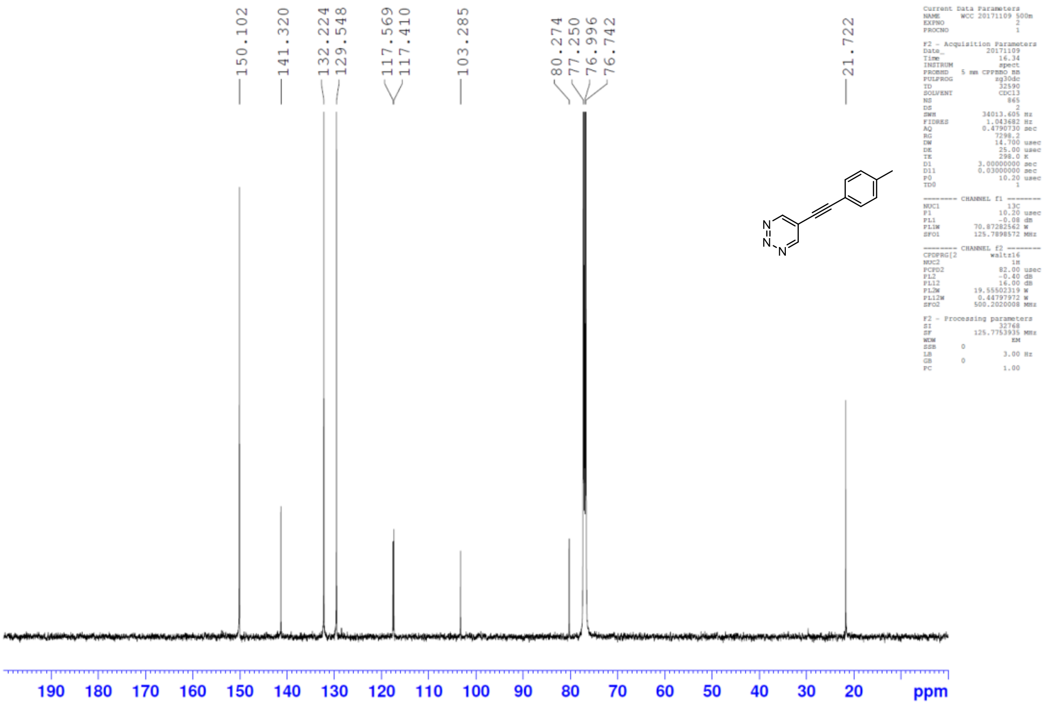

^{13}C{^{1}H} NMR spectrum of **3b** ($CDCl_3$, 125 MHz)

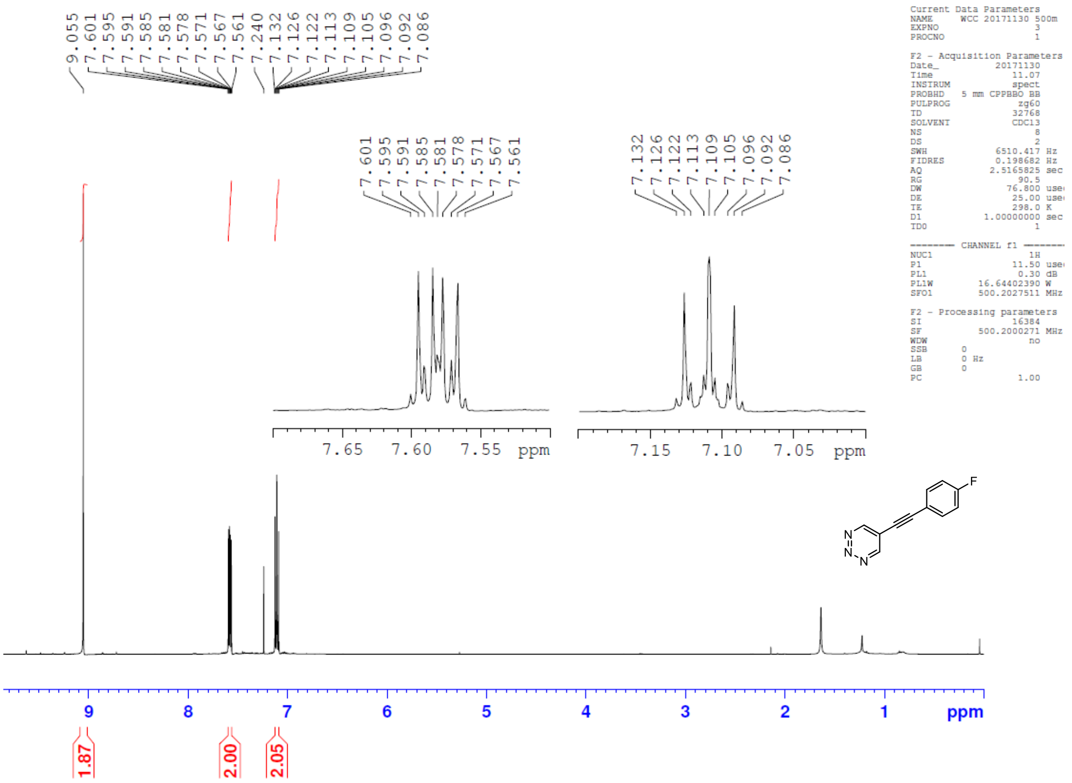

^{1}H NMR spectrum of **3c** ($CDCl_3$, 500 MHz)

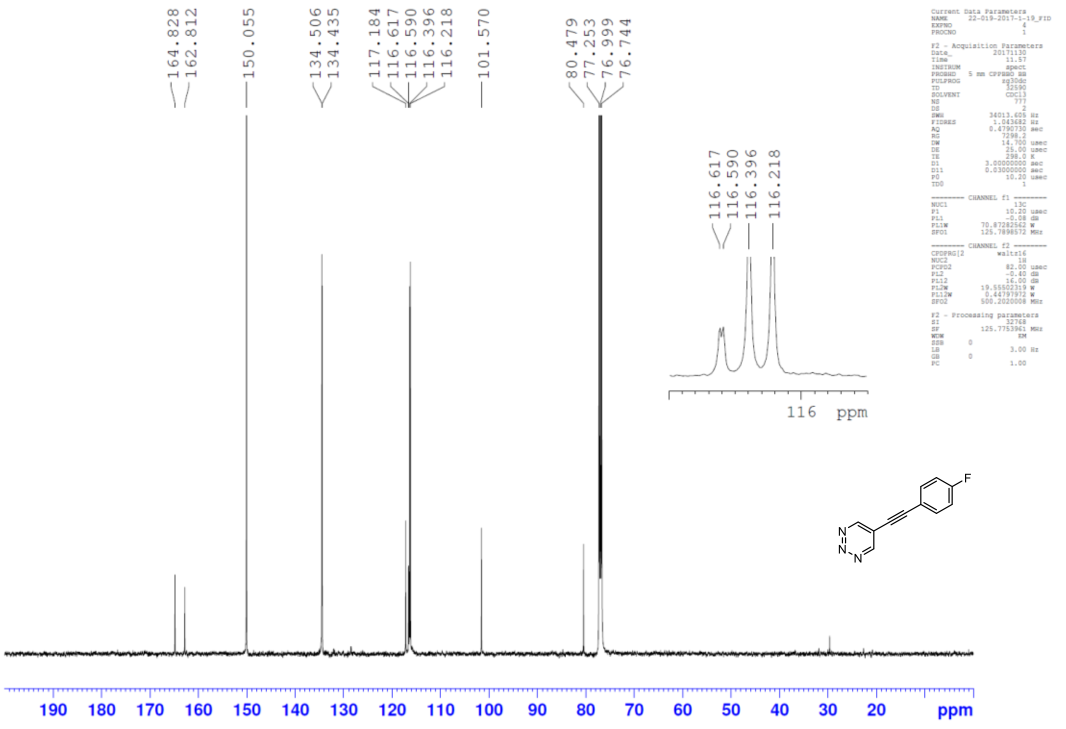

$^{13}C\{^{1}H\}$ NMR spectrum of **3c** ($CDCl_3$, 125 MHz)

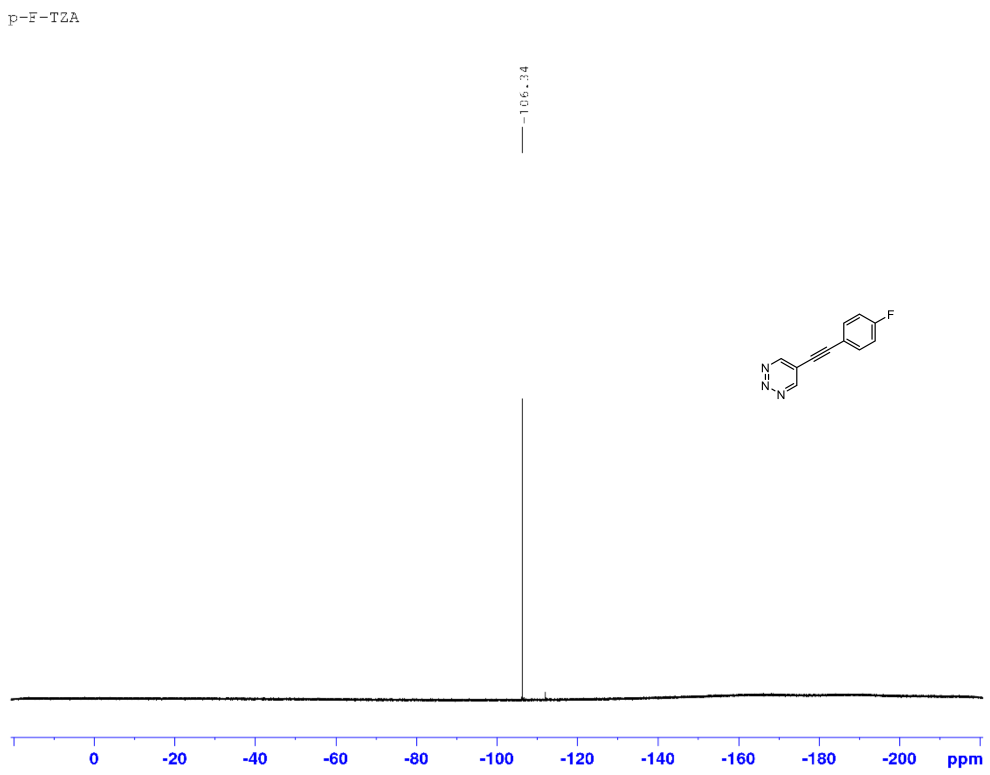

$^{19}F\{^{1}H\}$ NMR spectrum of **3c** ($CDCl_3$, 470 MHz)

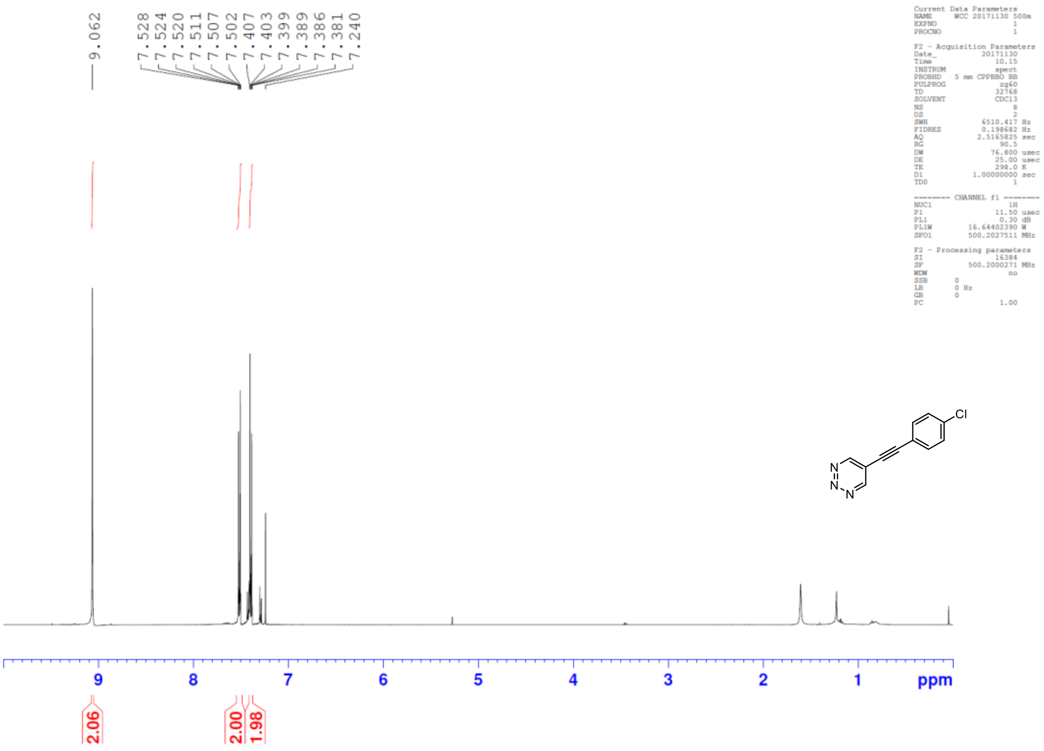

^{1}H NMR spectrum of **3d** ($CDCl_3$, 500 MHz)

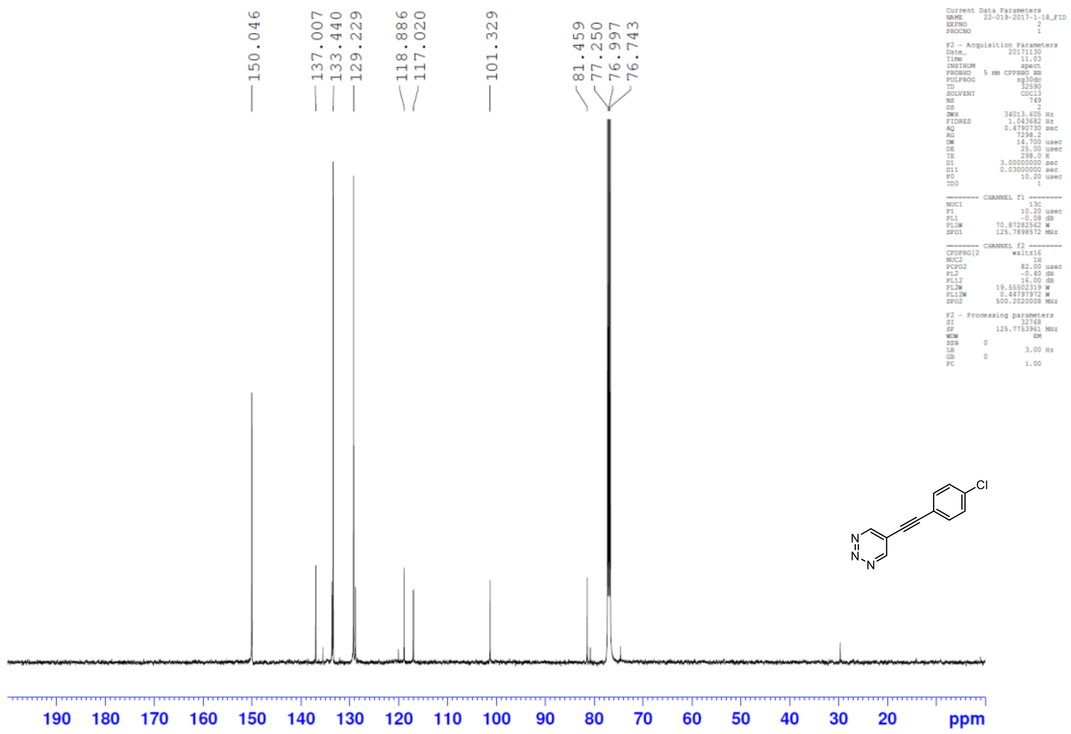

$^{13}C\{^{1}H\}$ NMR spectrum of **3d** ($CDCl_3$, 125 MHz)

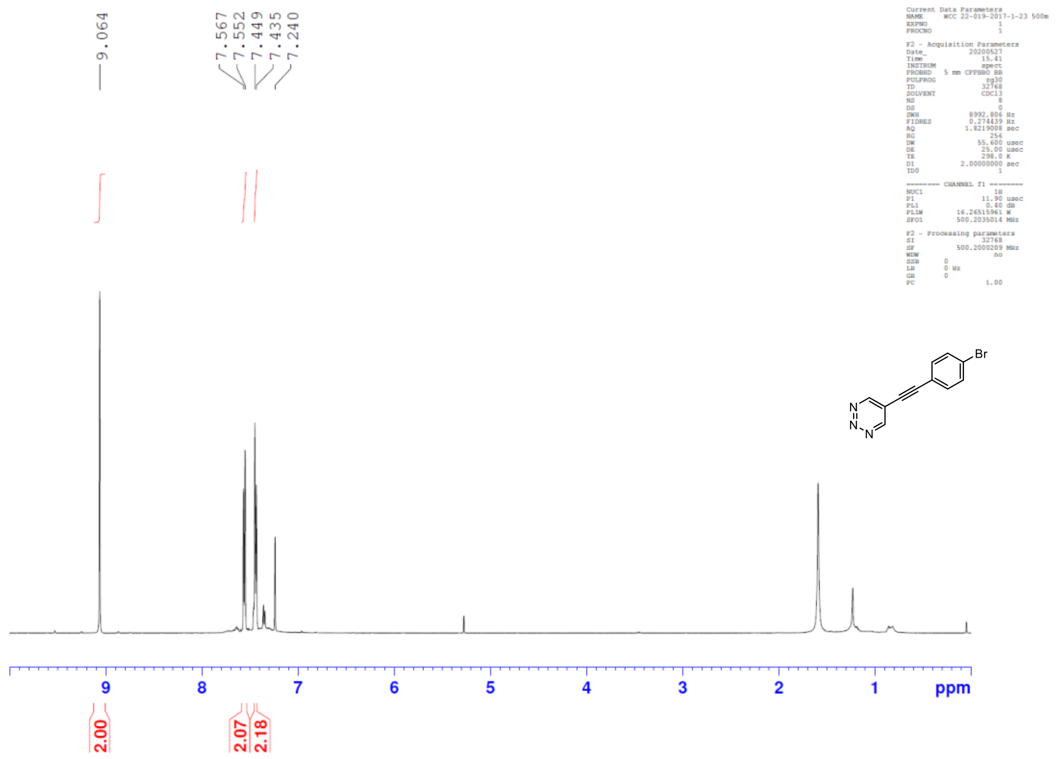

^{1}H NMR spectrum of **3e** ($CDCl_3$, 500 MHz)

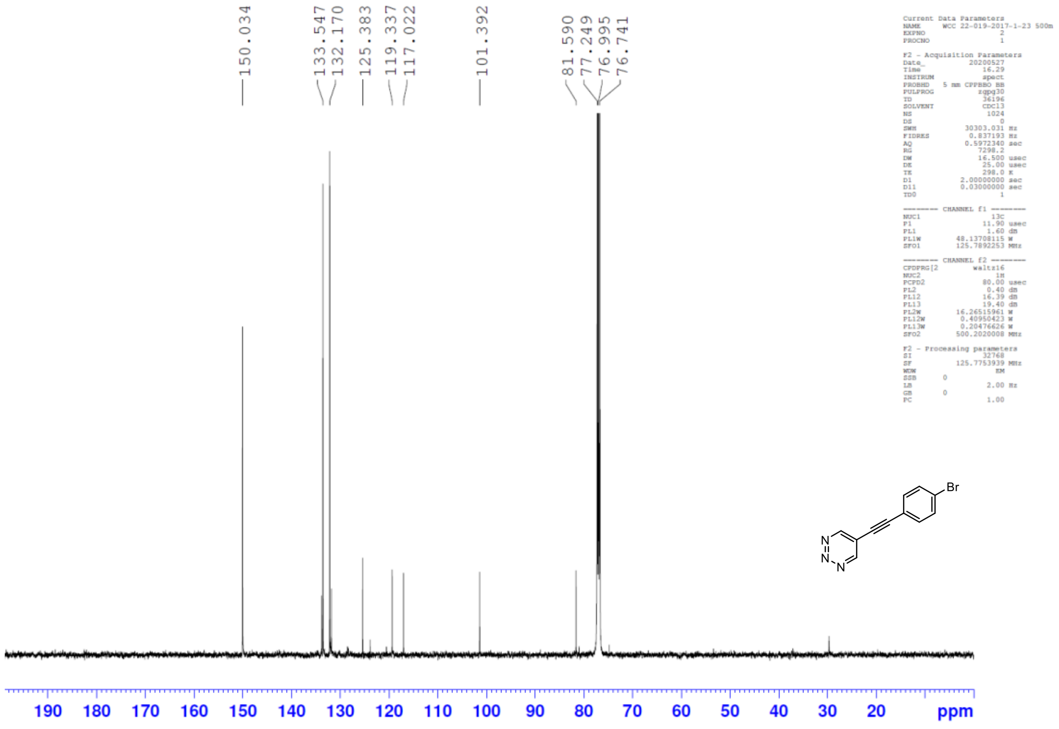

$^{13}C\{^1H\}$ NMR spectrum of **3e** ($CDCl_3$, 125 MHz)

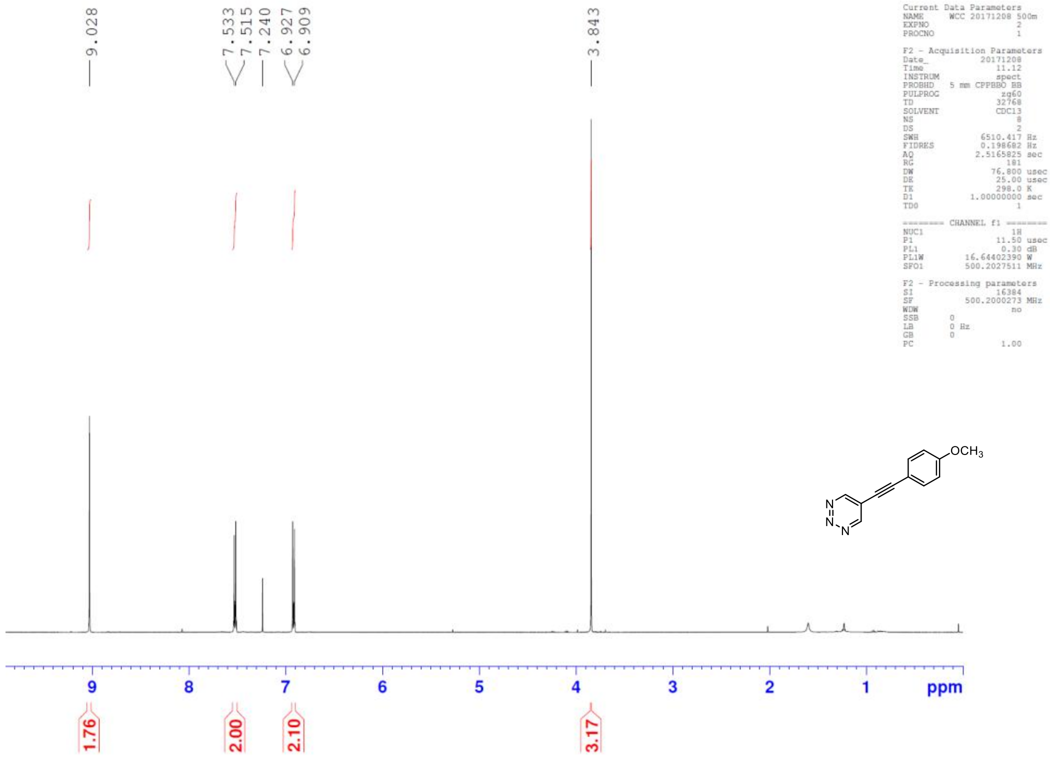

^1H NMR spectrum of **3f** ($CDCl_3$, 500 MHz)

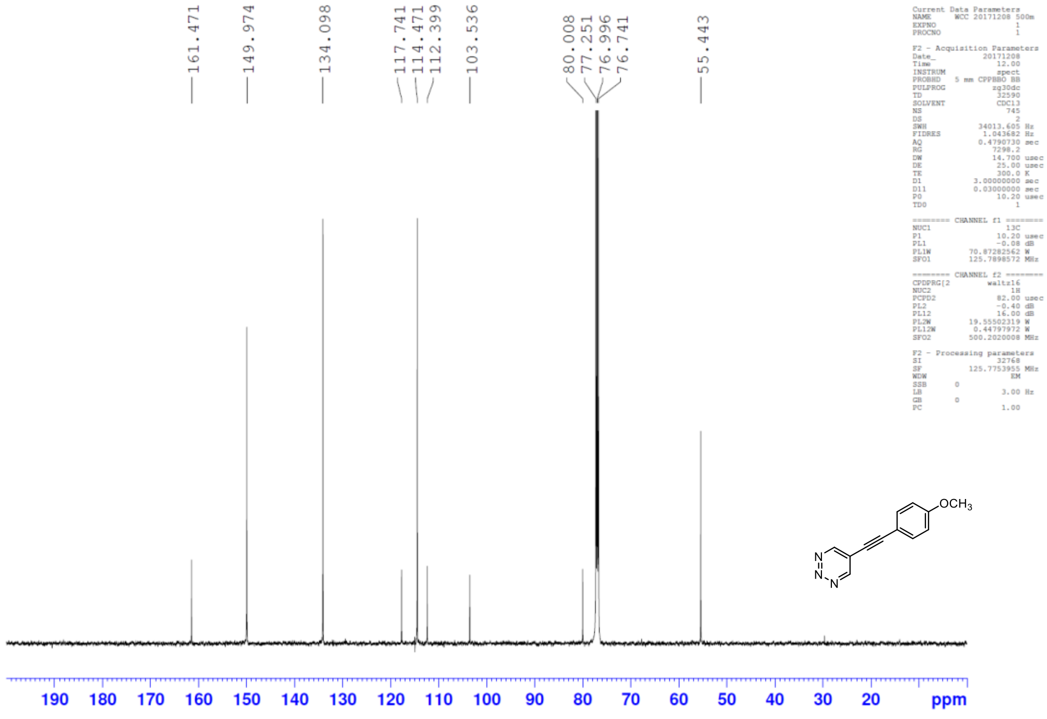

$^{13}C\{^{1}H\}$ NMR spectrum of **3f** ($CDCl_3$, 125 MHz)

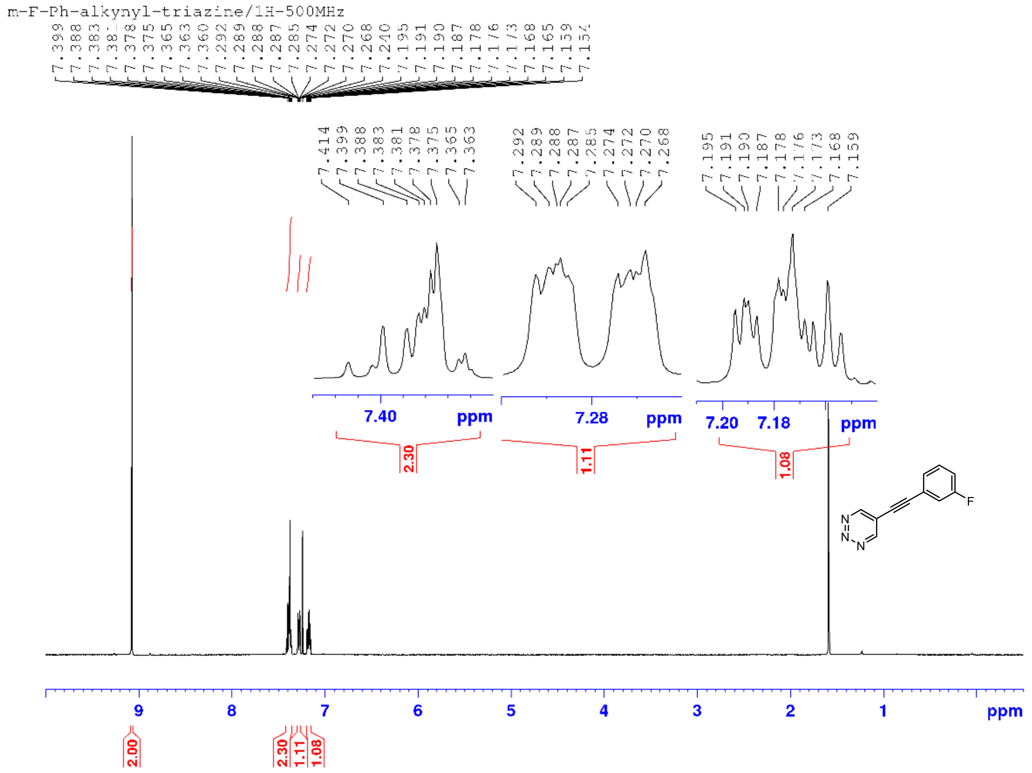

^{1}H NMR spectrum of **3g** ($CDCl_3$, 500 MHz)

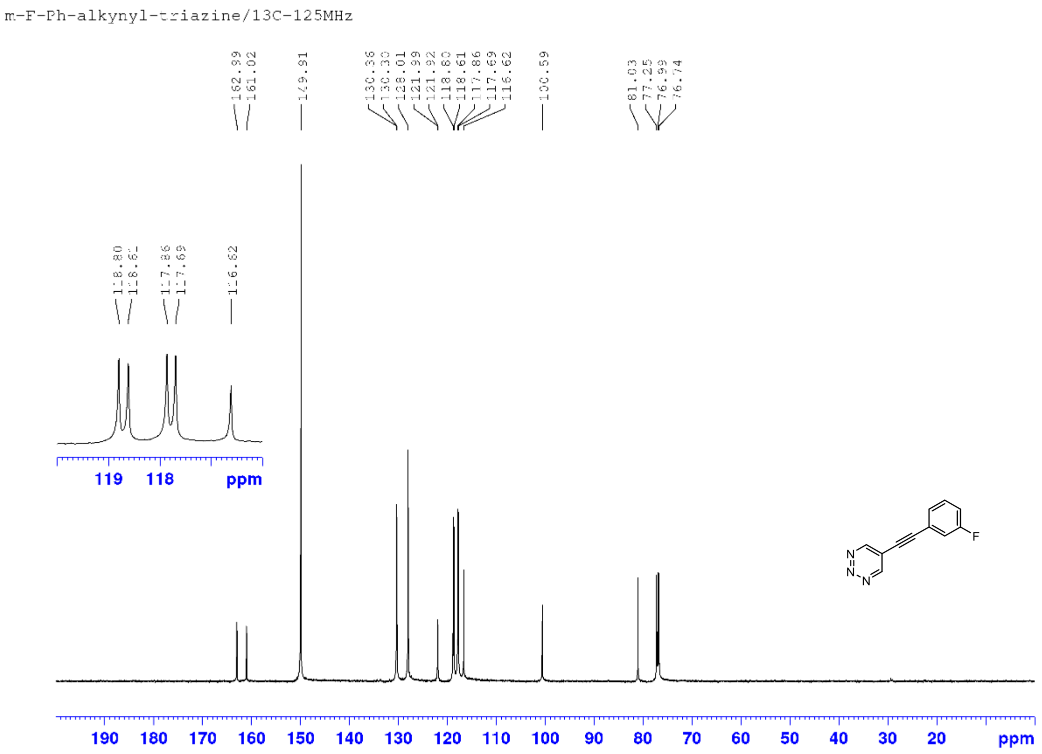

$^{13}C\{^{1}H\}$ NMR spectrum of **3g** ($CDCl_3$, 125 MHz)

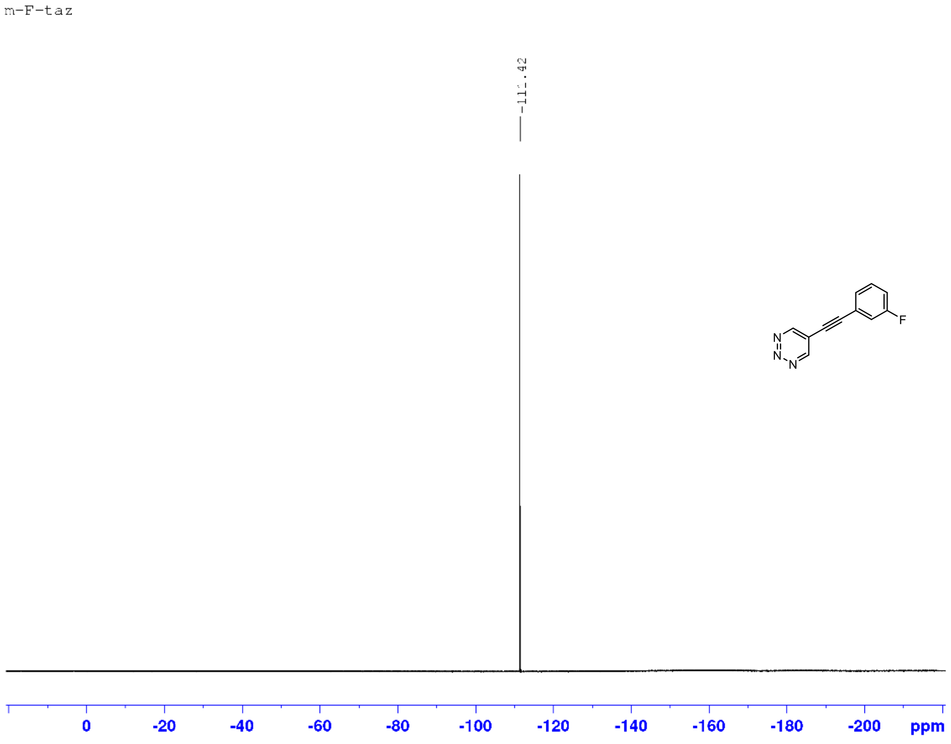

$^{19}F\{^{1}H\}$ NMR spectrum of **3g** ($CDCl_3$, 470 MHz)

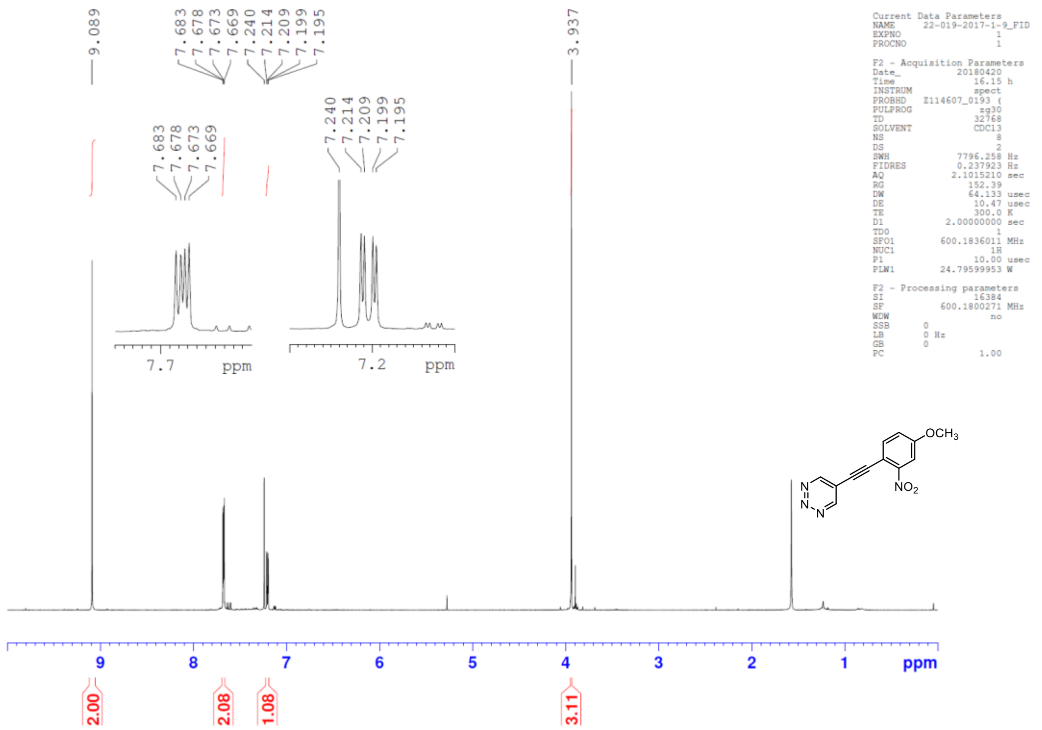

^{1}H NMR spectrum of **3h** ($CDCl_3$, 600 MHz)

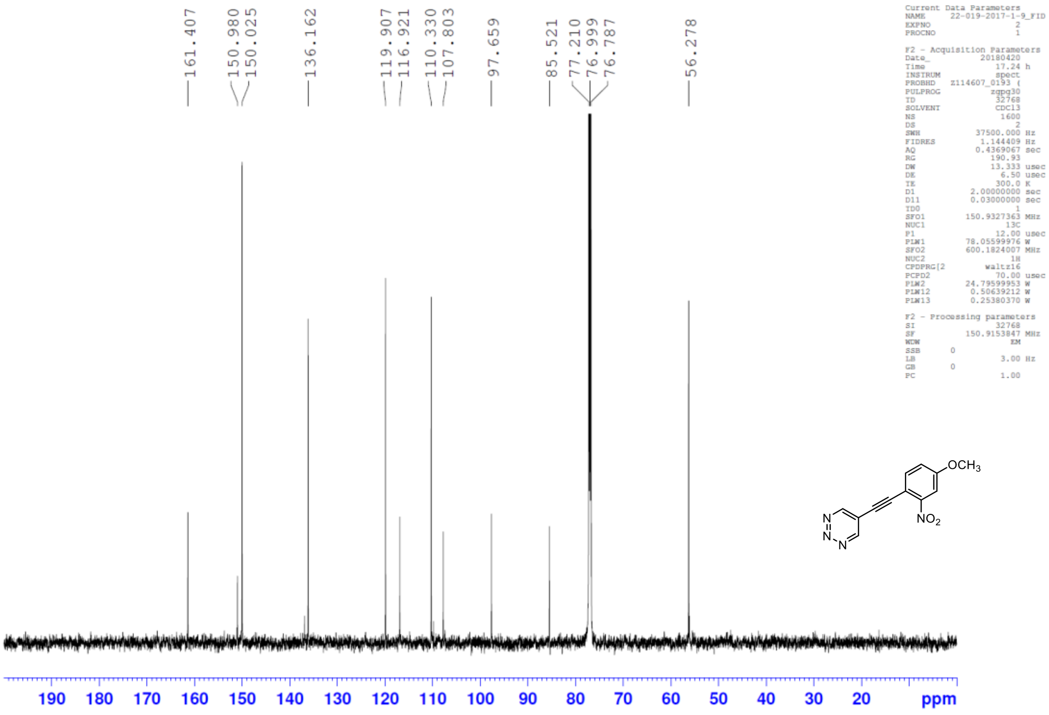

^{13}C{^{1}H} NMR spectrum of **3h** ($CDCl_3$, 150 MHz)

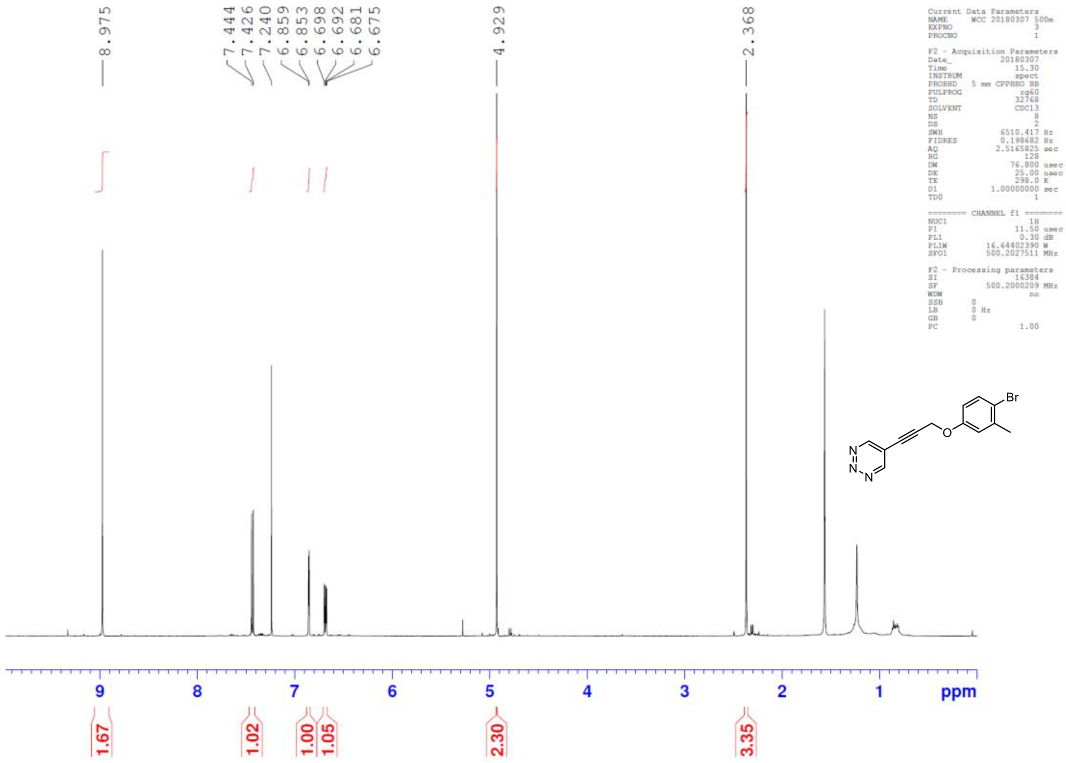

^{1}H NMR spectrum of **3i** ($CDCl_3$, 500 MHz)

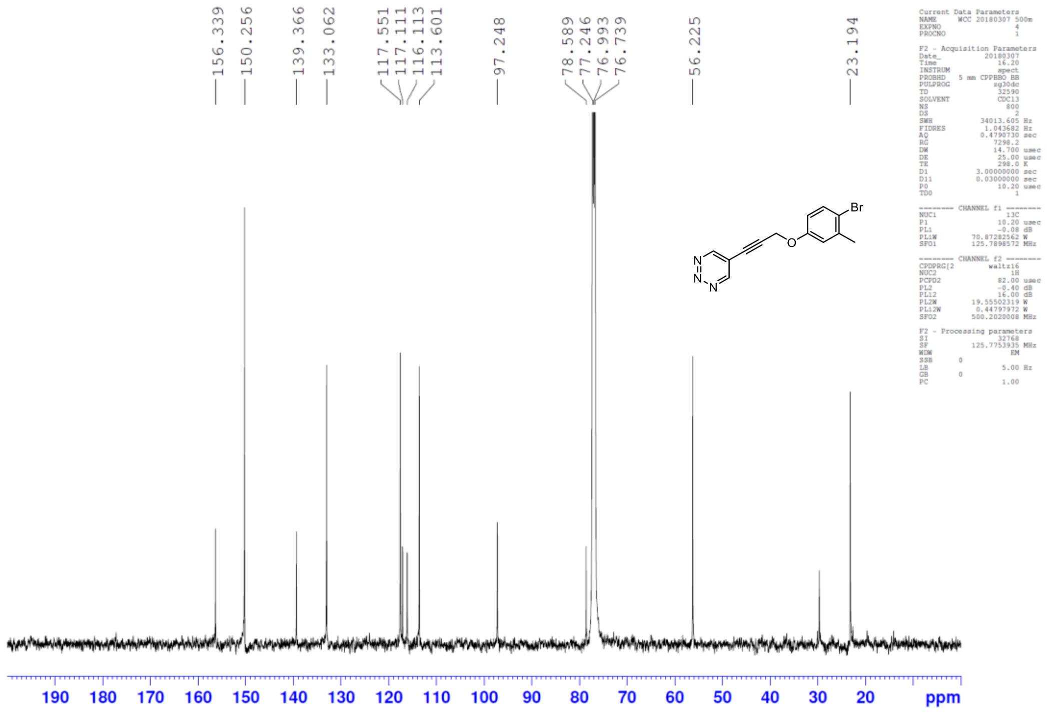

^{13}C{^{1}H} NMR spectrum of **3i** ($CDCl_3$, 125 MHz)

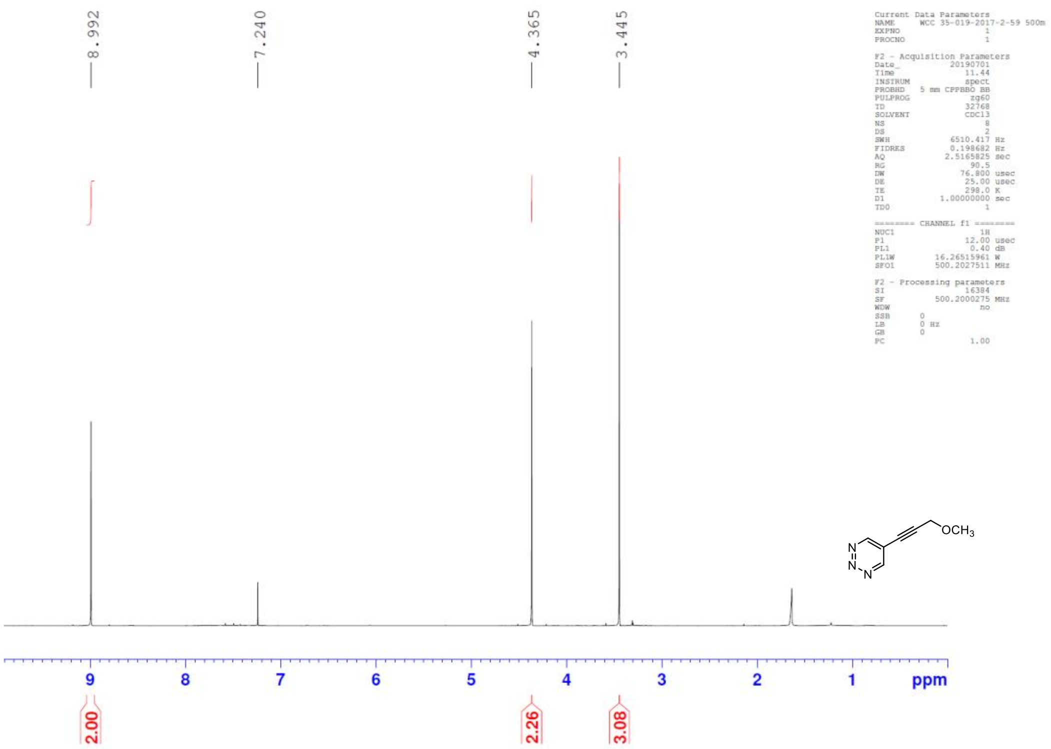

^{1}H NMR spectrum of **3j** ($CDCl_3$, 500 MHz)

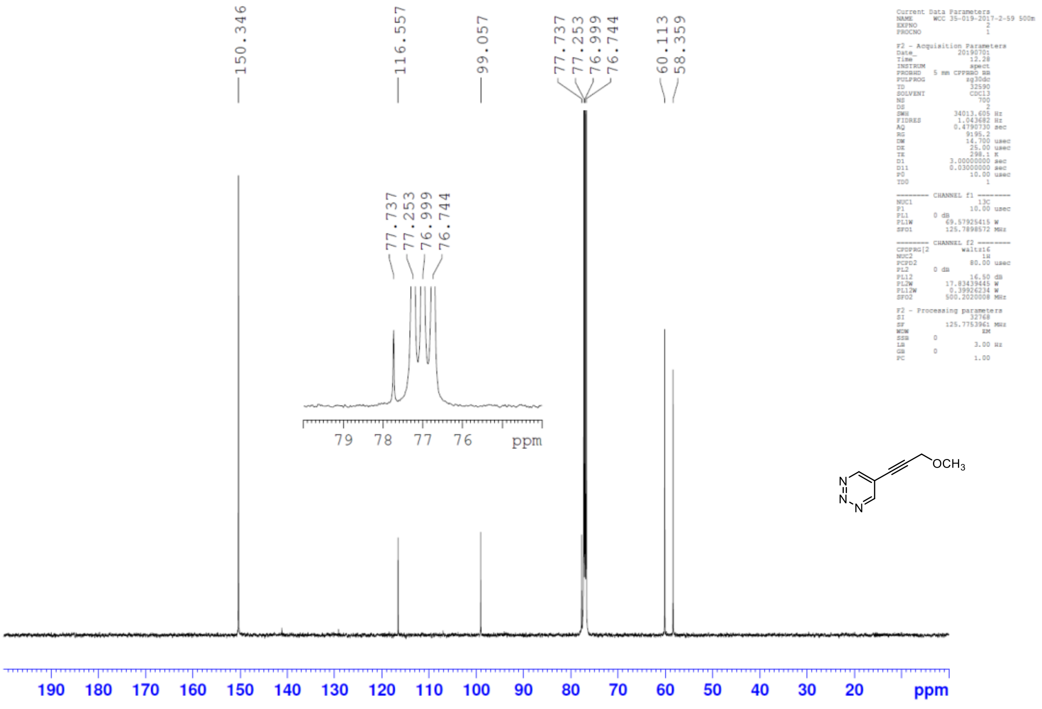

^{13}C{^{1}H} NMR spectrum of **3j** ($CDCl_3$, 125 MHz)

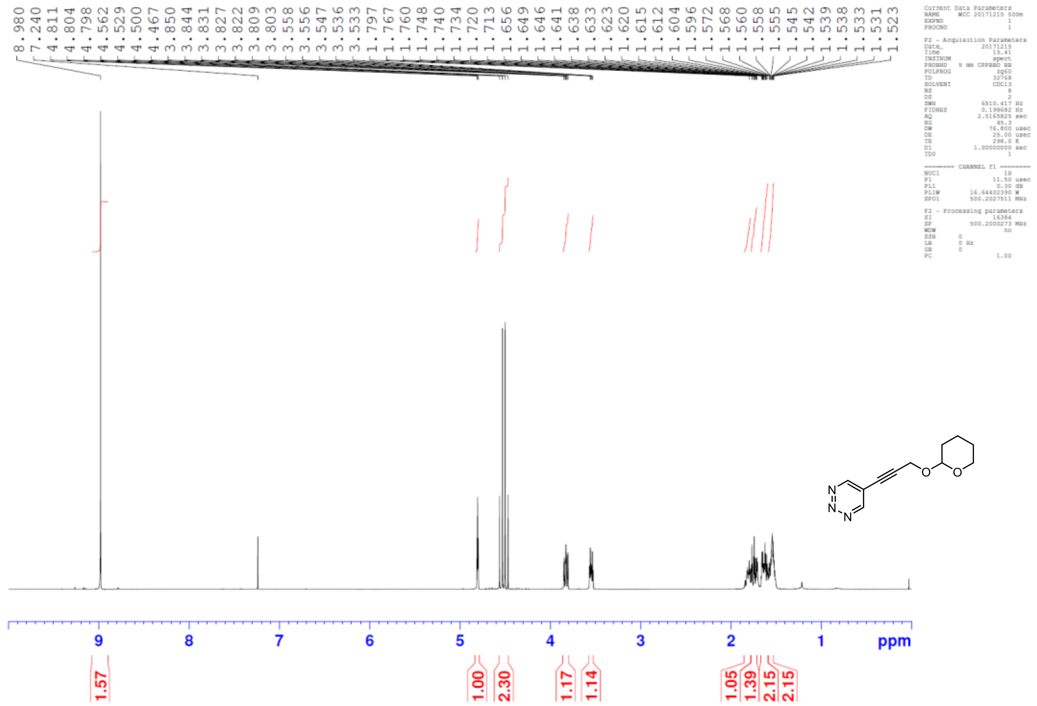

^{1}H NMR spectrum of **3k** ($CDCl_3$, 500 MHz)

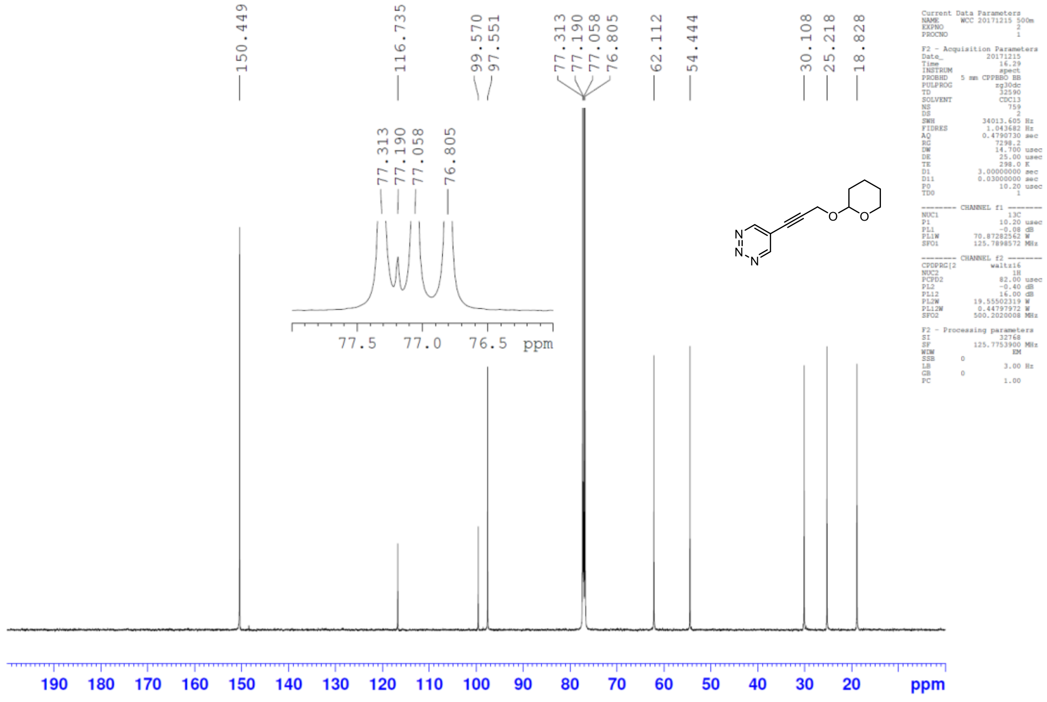

^{13}C{^{1}H} NMR spectrum of **3k** ($CDCl_3$, 125 MHz)

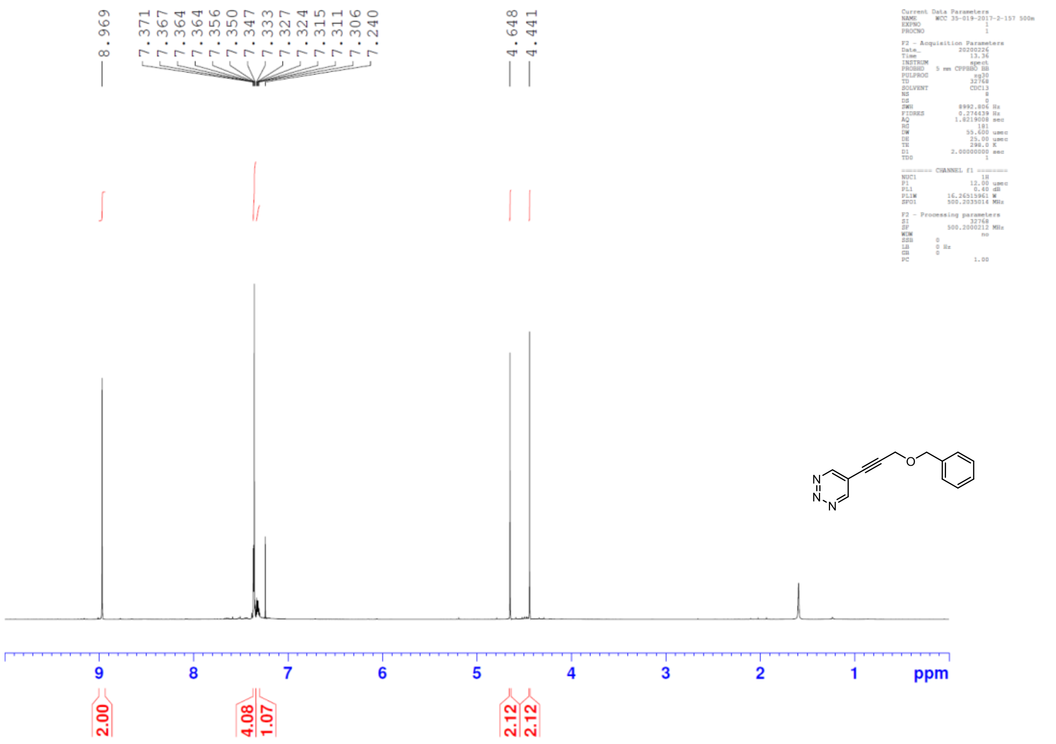

^{1}H NMR spectrum of **3l** ($CDCl_3$, 500 MHz)

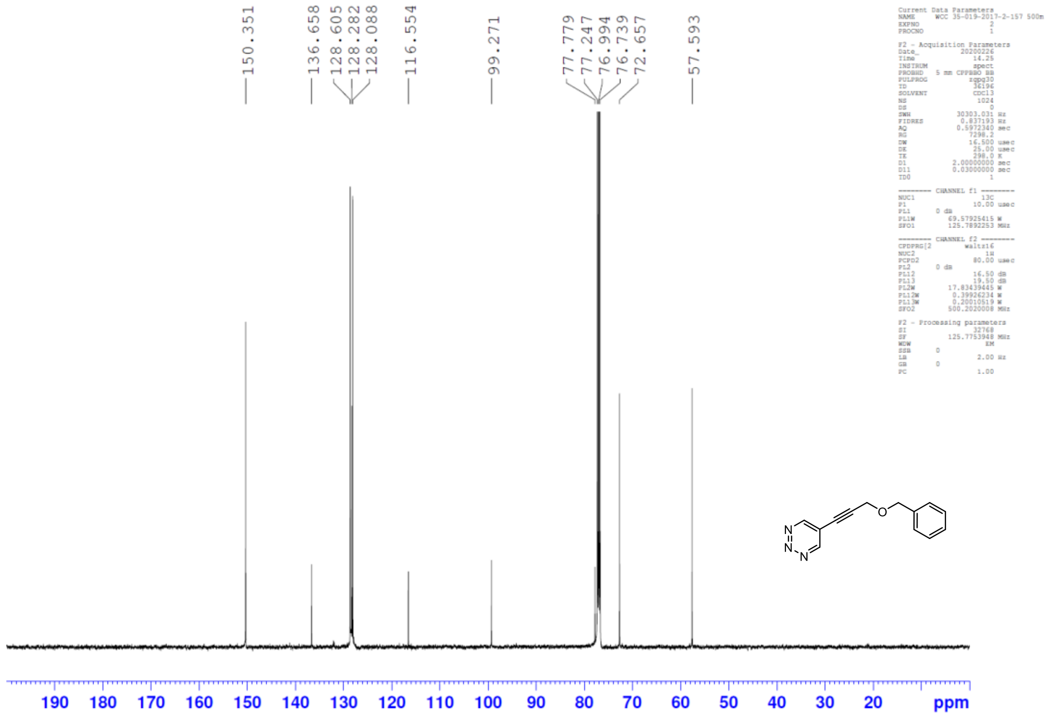

^{13}C{^{1}H} NMR spectrum of **3l** ($CDCl_3$, 125 MHz)

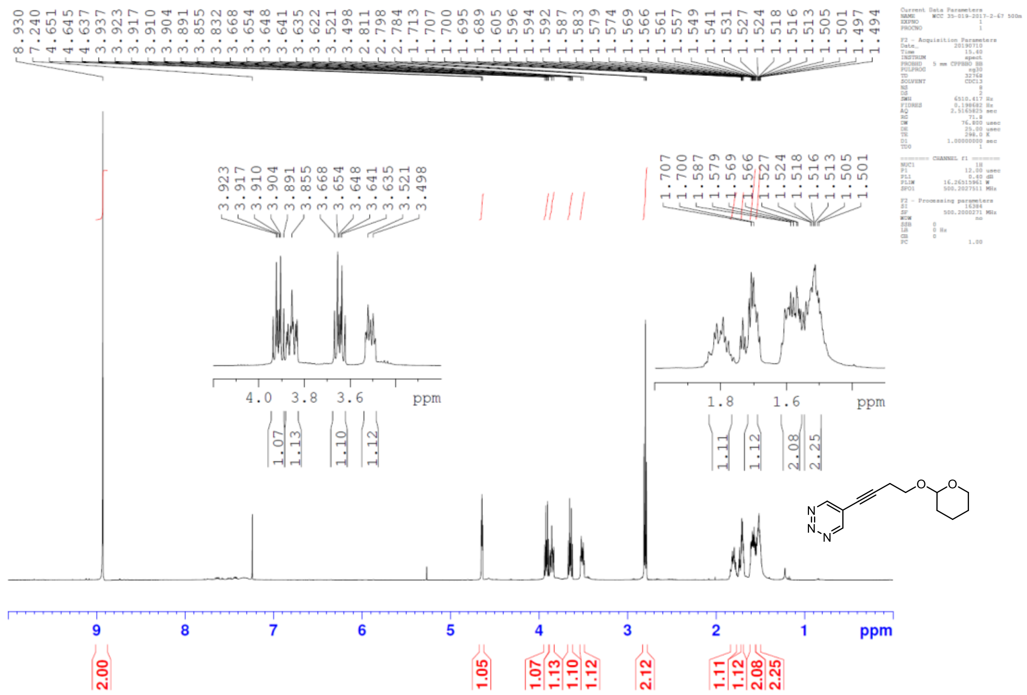

^{1}H NMR spectrum of **3m** ($CDCl_3$, 500 MHz)

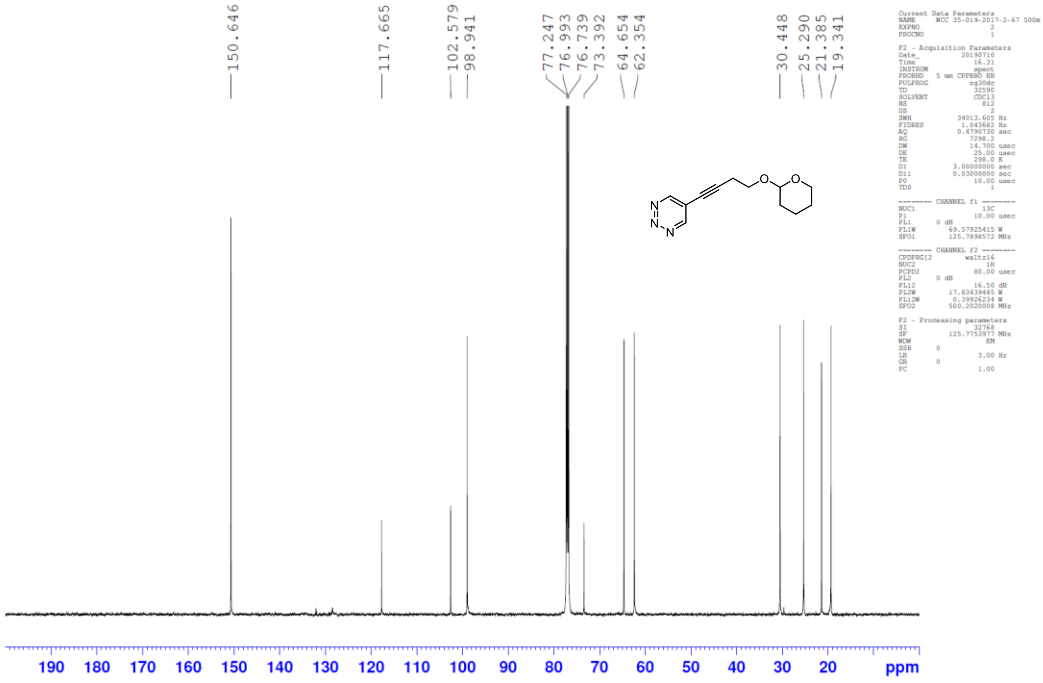

^{13}C{^{1}H} NMR spectrum of **3m** ($CDCl_3$, 125 MHz)

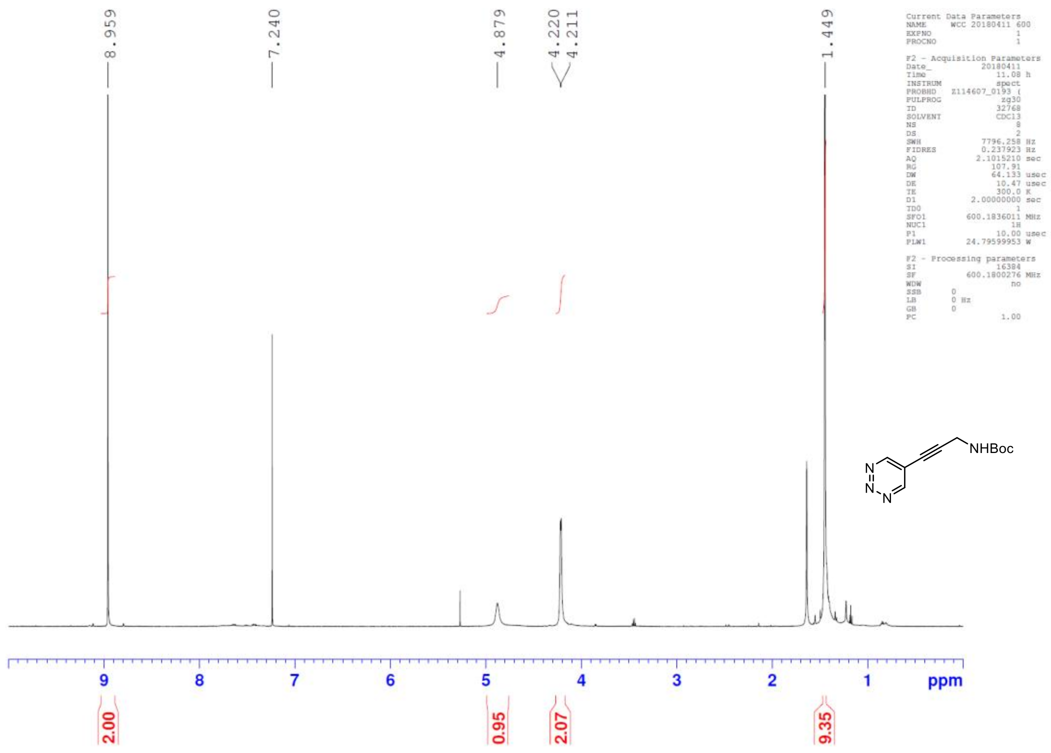

^{1}H NMR spectrum of **3n** ($CDCl_3$, 600 MHz)

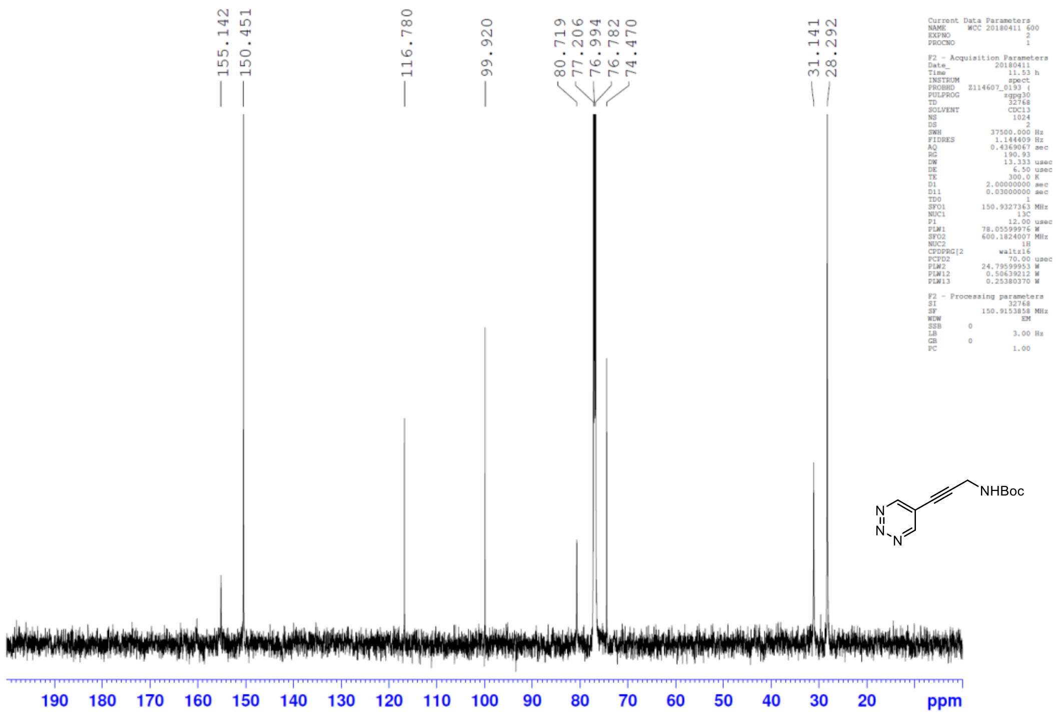

^{13}C{^{1}H} NMR spectrum of **3n** ($CDCl_3$, 150 MHz)

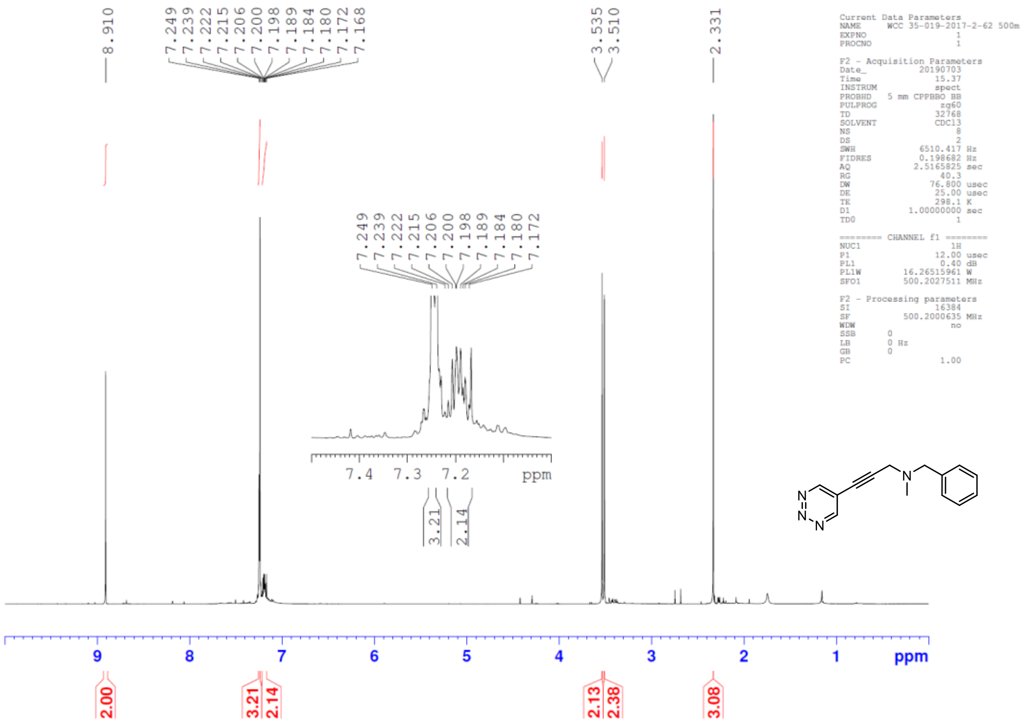

^{1}H NMR spectrum of **3o** ($CDCl_3$, 500 MHz)

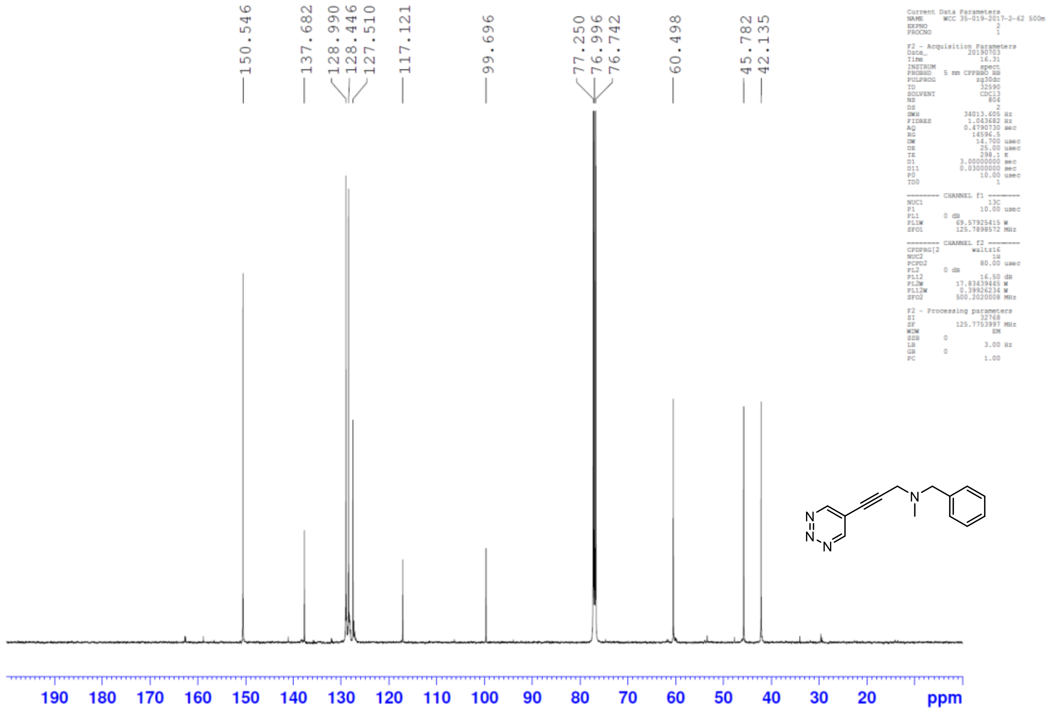

$^{13}C\{^{1}H\}$ NMR spectrum of **3o** ($CDCl_3$, 125 MHz)

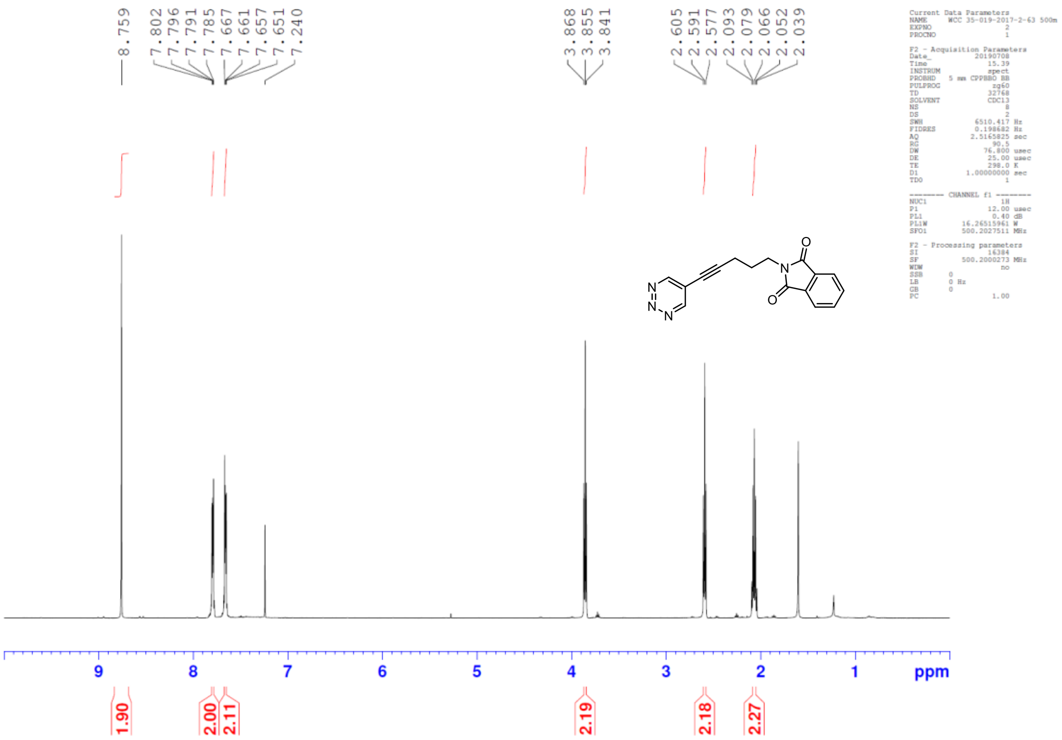

^{1}H NMR spectrum of **3p** ($CDCl_3$, 500 MHz)

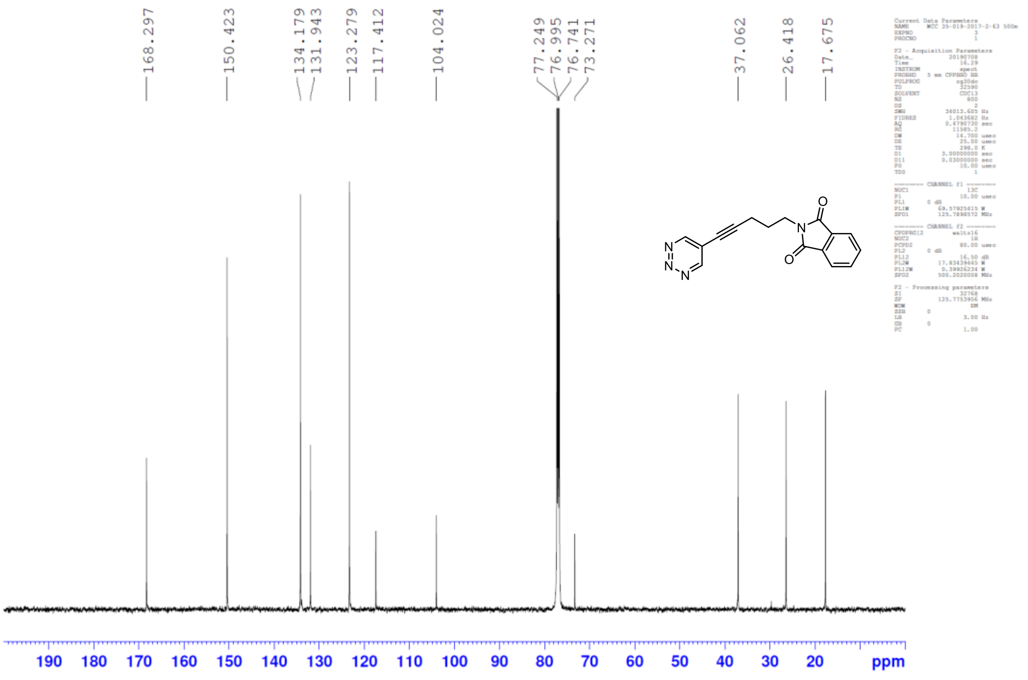

^{13}C{^{1}H} NMR spectrum of **3p** ($CDCl_3$, 125 MHz)

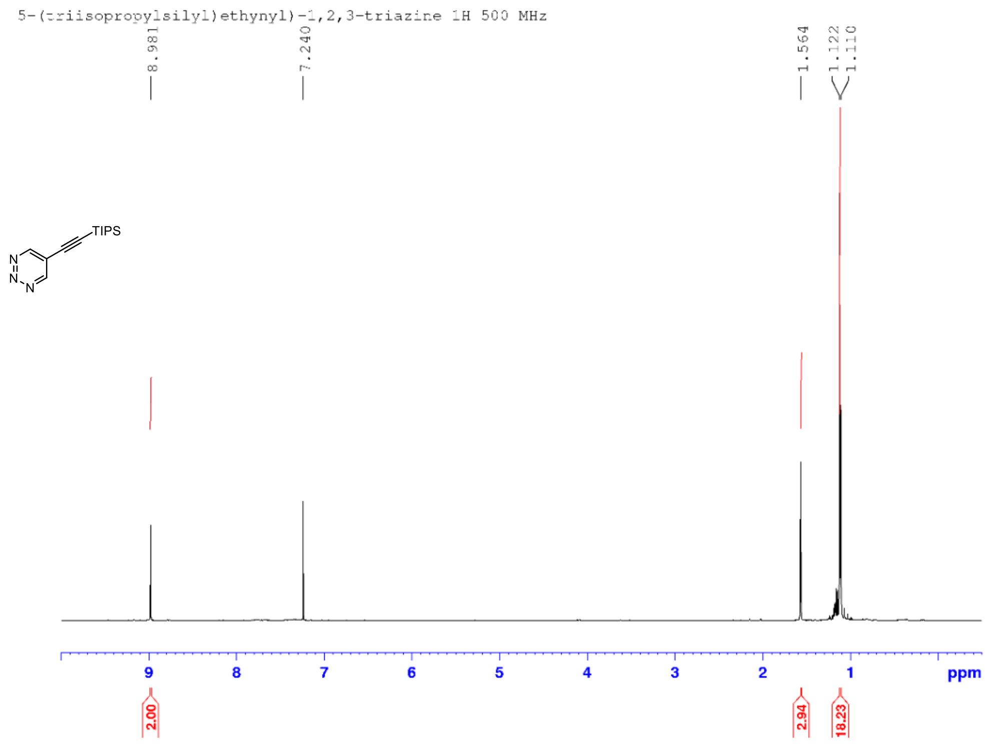

^{1}H NMR spectrum of **3q** ($CDCl_3$, 500 MHz)

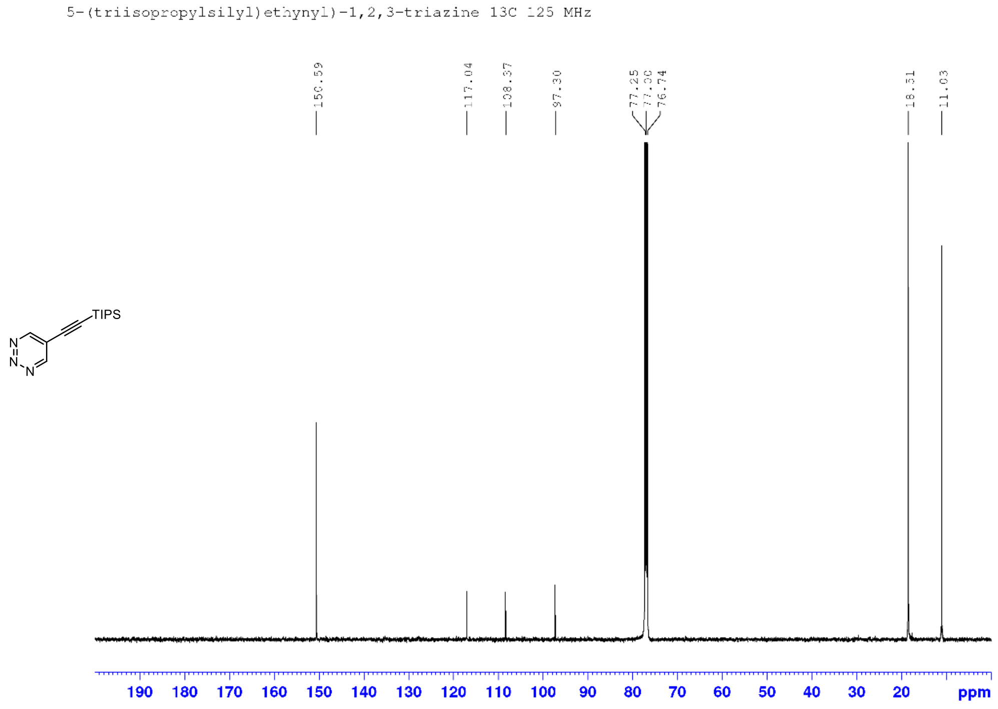

$^{13}C\{^{1}H\}$ NMR spectrum of **3q** ($CDCl_3$, 125 MHz)

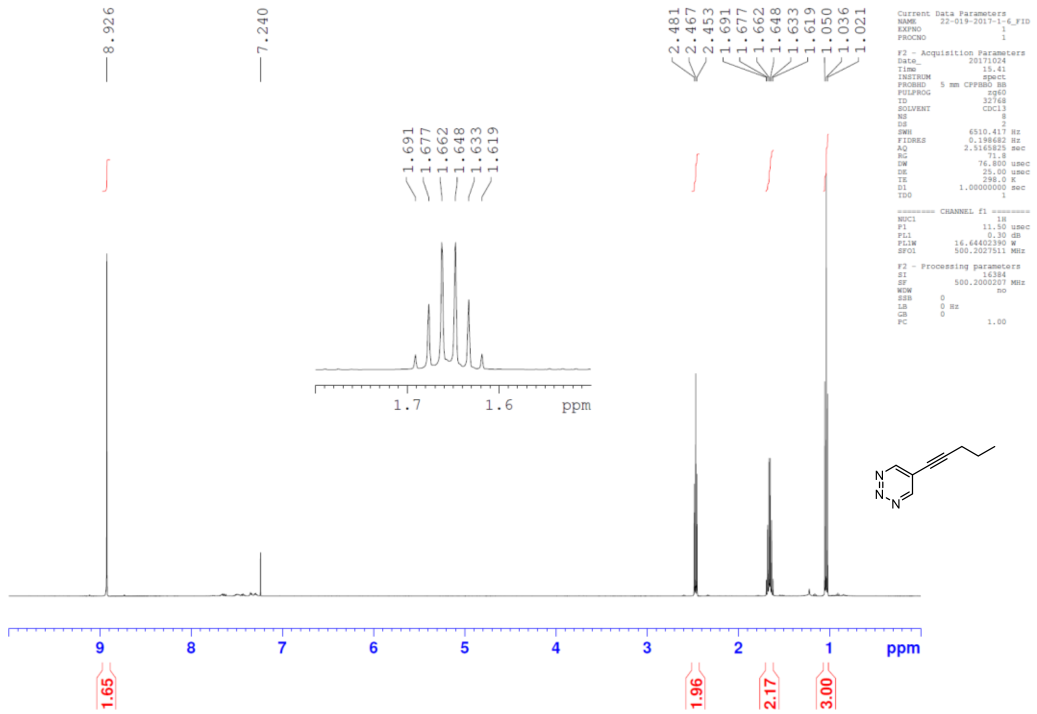

^{1}H NMR spectrum of **3r** ($CDCl_3$, 500 MHz)

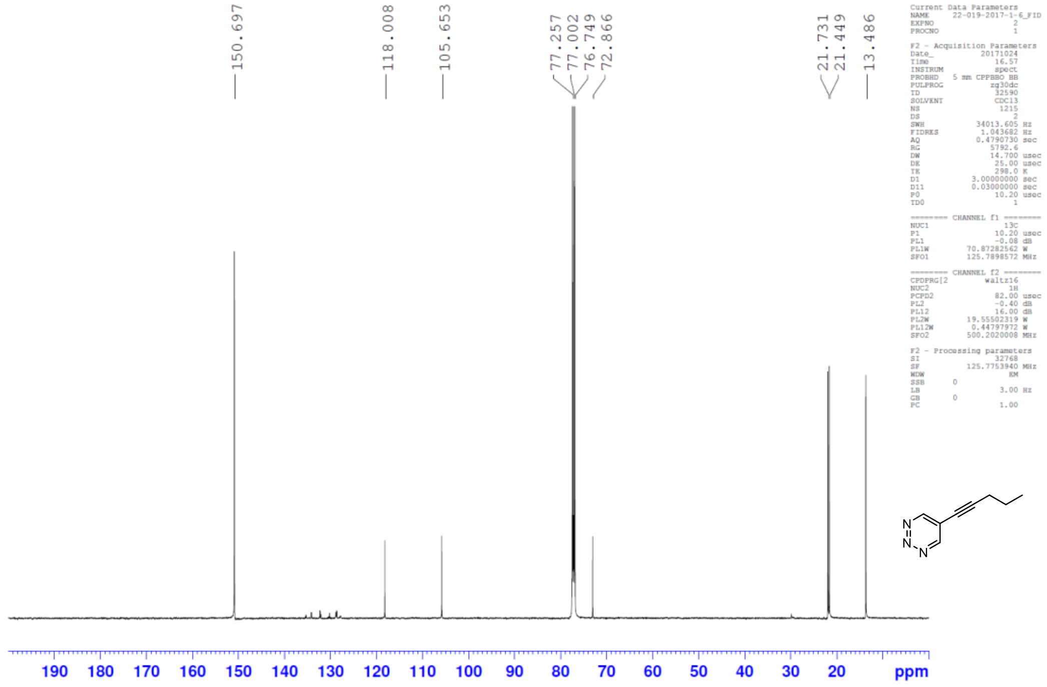

^{13}C{^{1}H} NMR spectrum of **3r** ($CDCl_3$, 125 MHz)

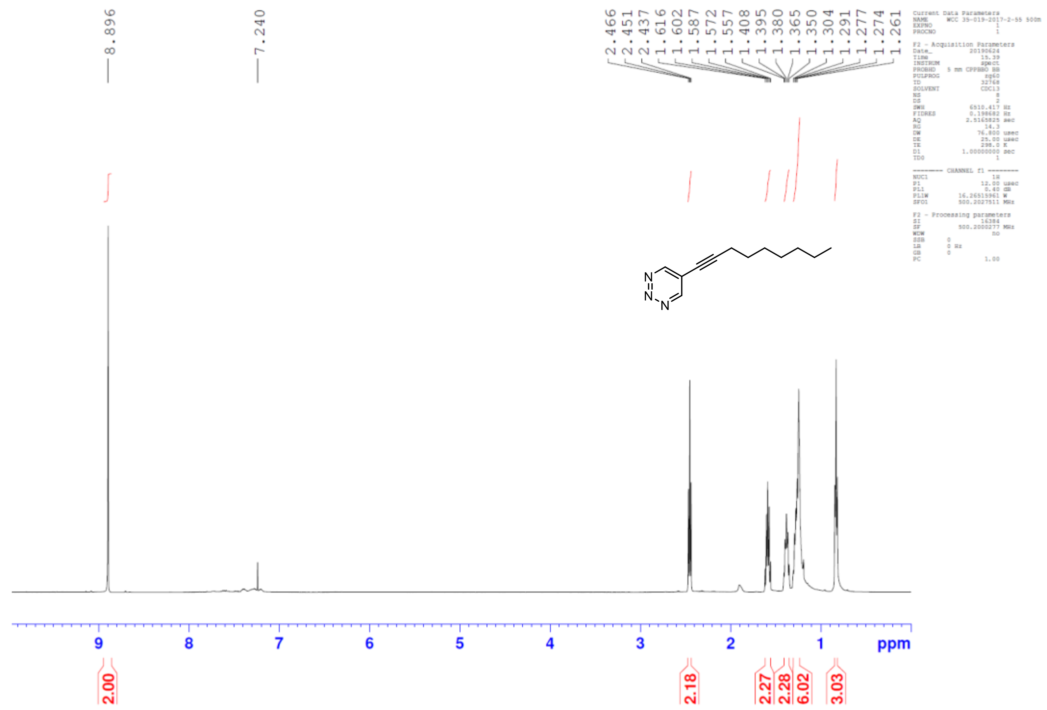

^{1}H NMR spectrum of **3s** ($CDCl_3$, 500 MHz)

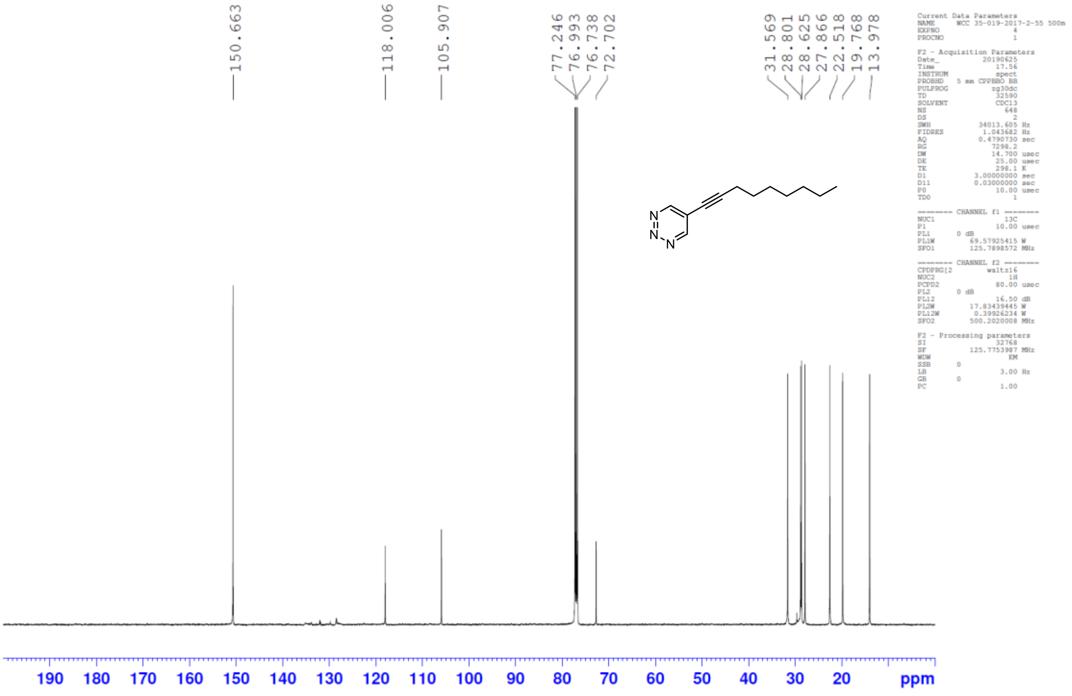

$^{13}C\{^{1}H\}$ NMR spectrum of **3s** ($CDCl_3$, 125 MHz)

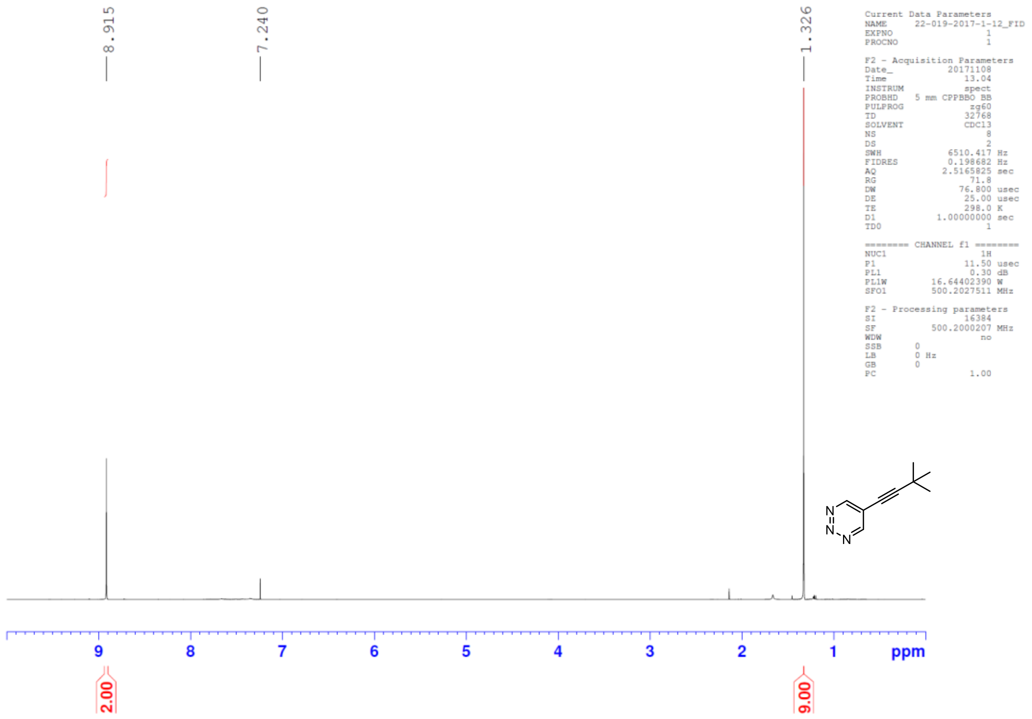

^{1}H NMR spectrum of **3t** ($CDCl_3$, 500 MHz)

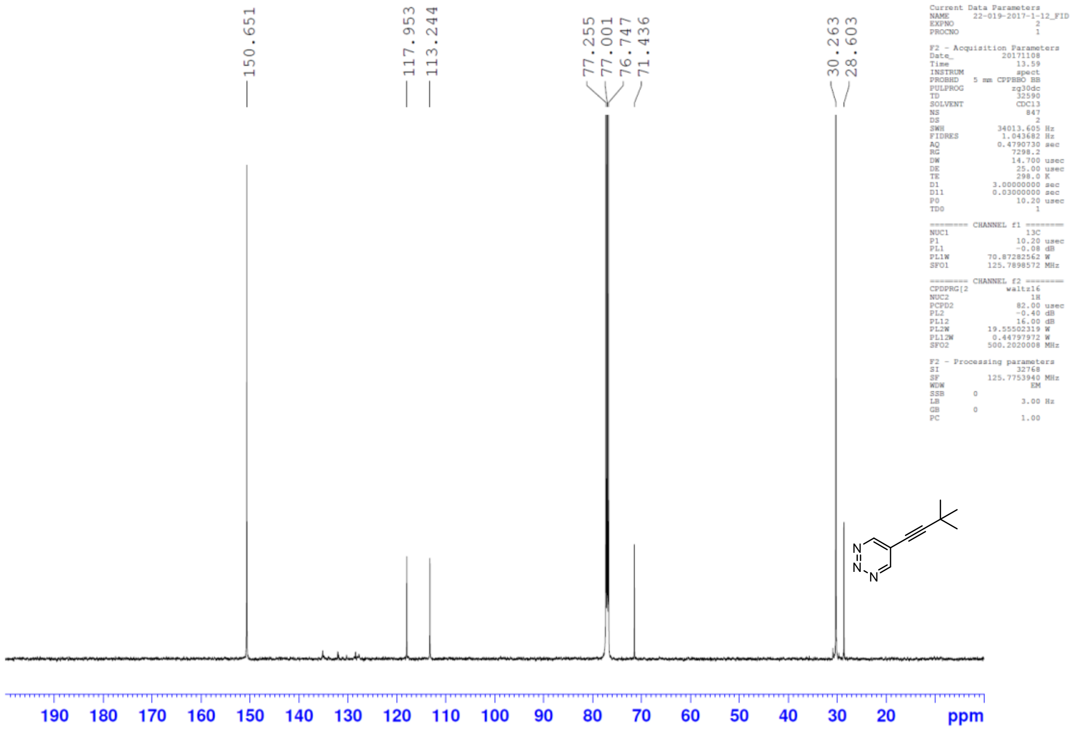

^{13}C{^{1}H} NMR spectrum of **3t** ($CDCl_3$, 125 MHz)

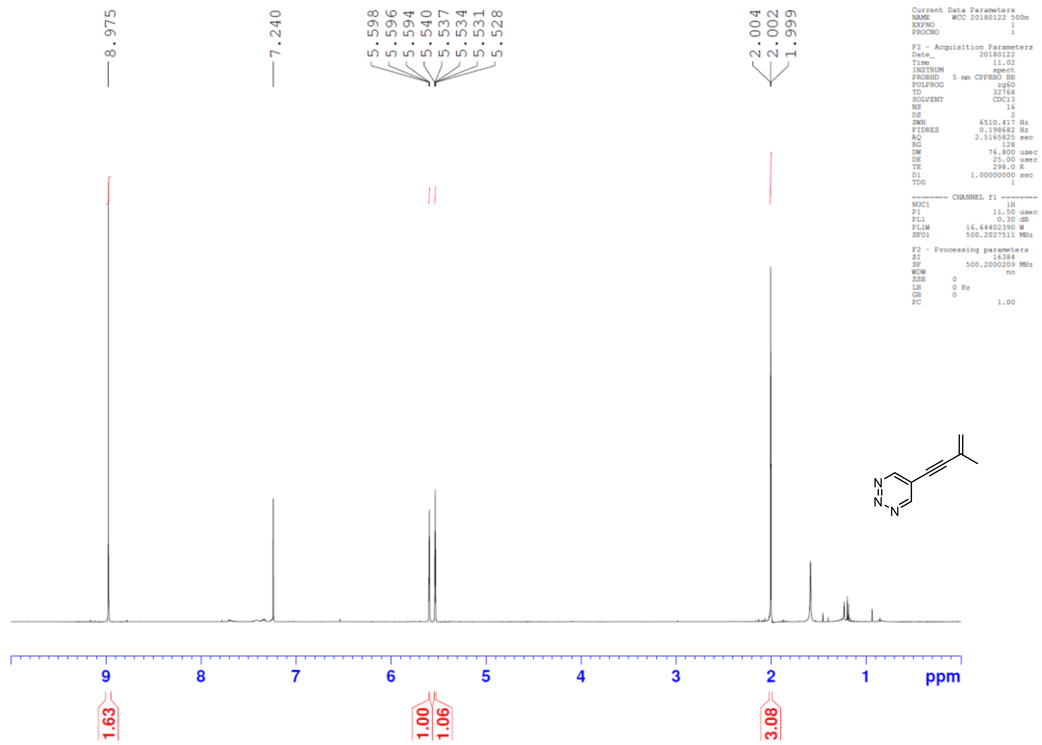

^{1}H NMR spectrum of **3u** ($CDCl_3$, 500 MHz)

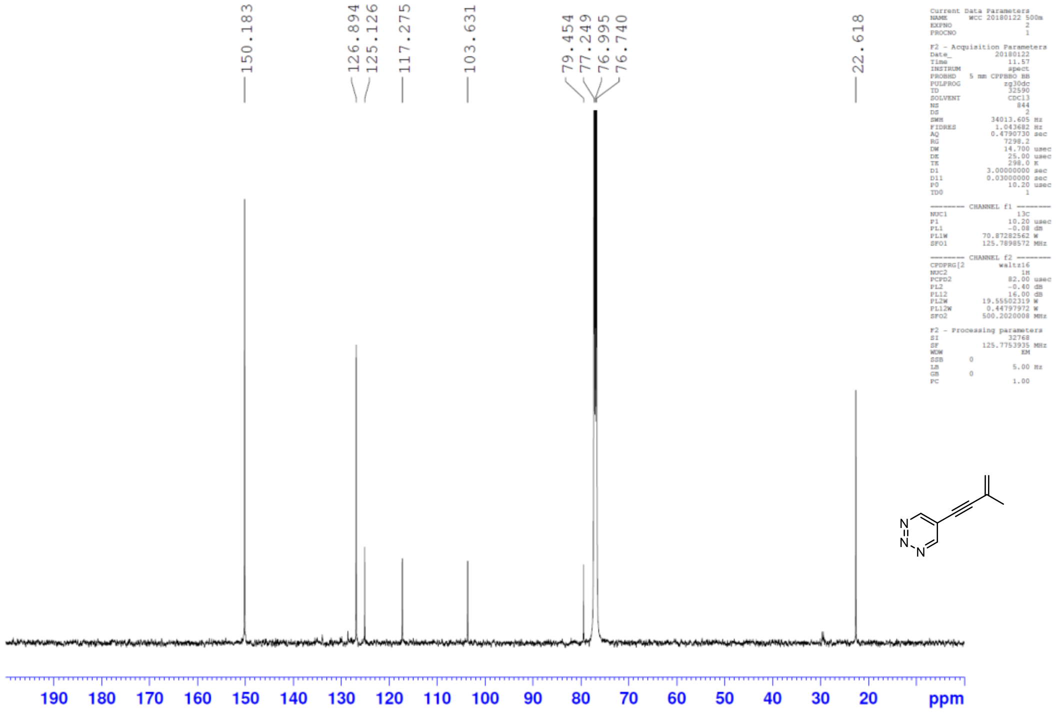

$^{13}C\{^{1}H\}$ NMR spectrum of **3u** ($CDCl_3$, 125 MHz)

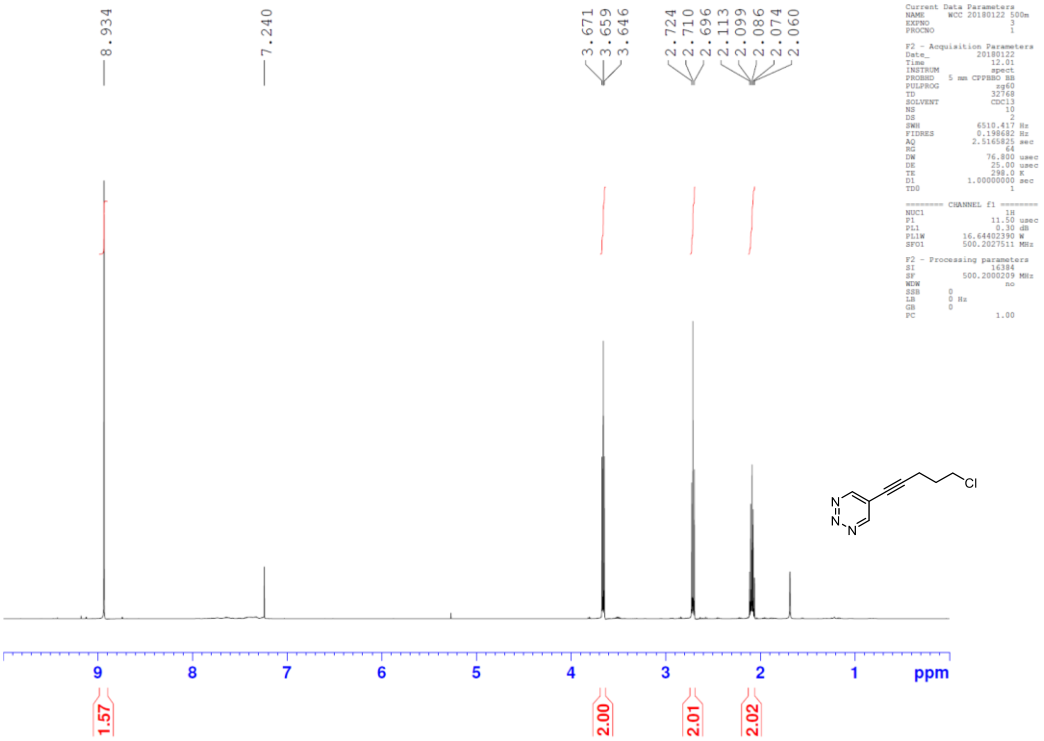

^{1}H NMR spectrum of **3v** ($CDCl_3$, 500 MHz)

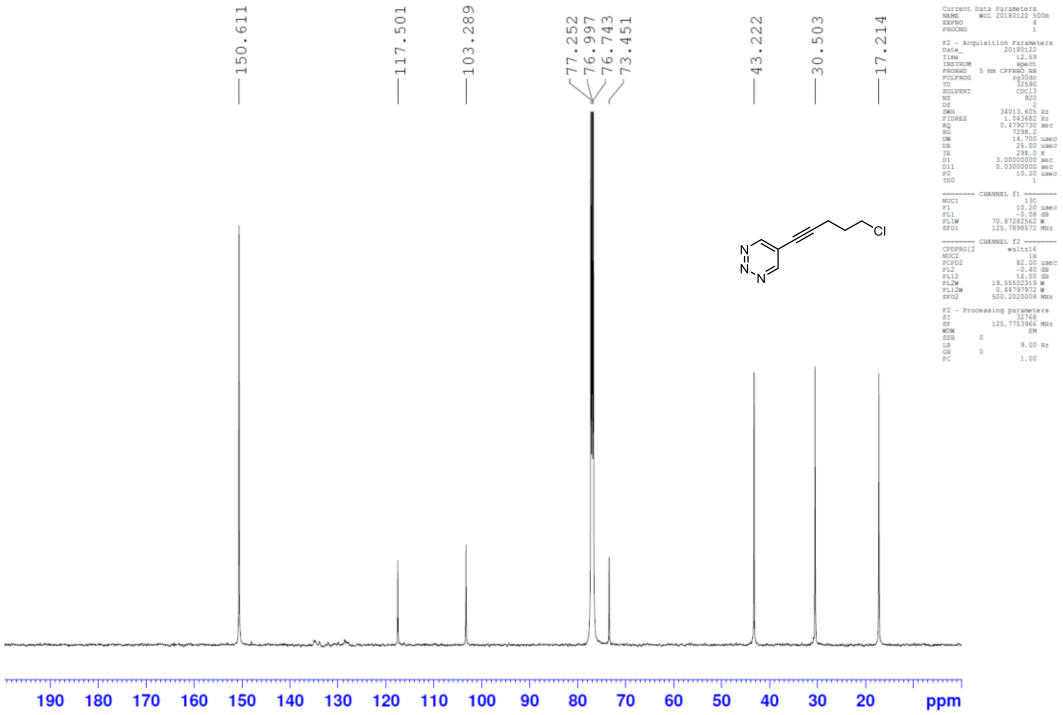

^{13}C{^{1}H} NMR spectrum of **3v** ($CDCl_3$, 125 MHz)

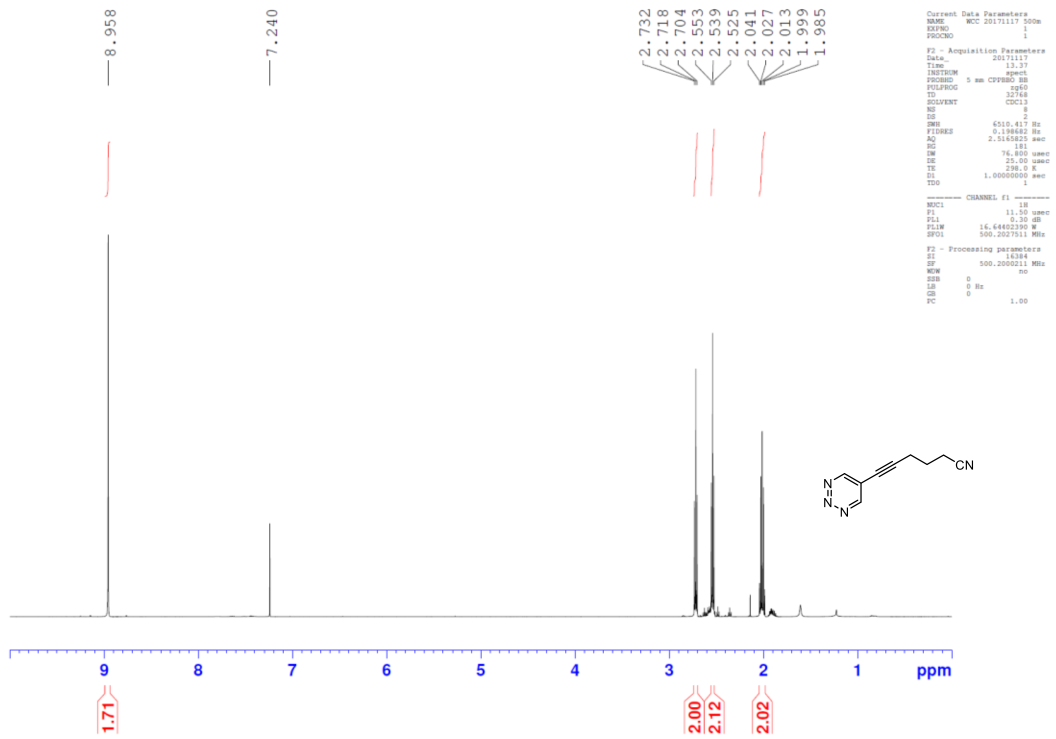

^{1}H NMR spectrum of **3w** ($CDCl_3$, 500 MHz)

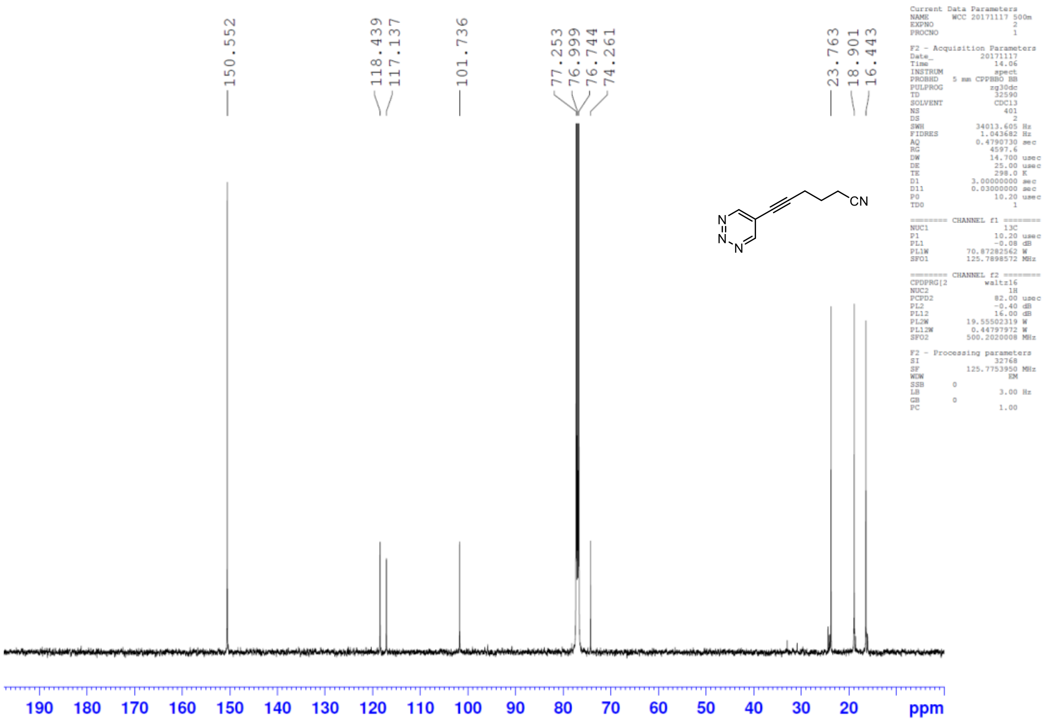

^{13}C{^{1}H} NMR spectrum of **3w** ($CDCl_3$, 125 MHz)

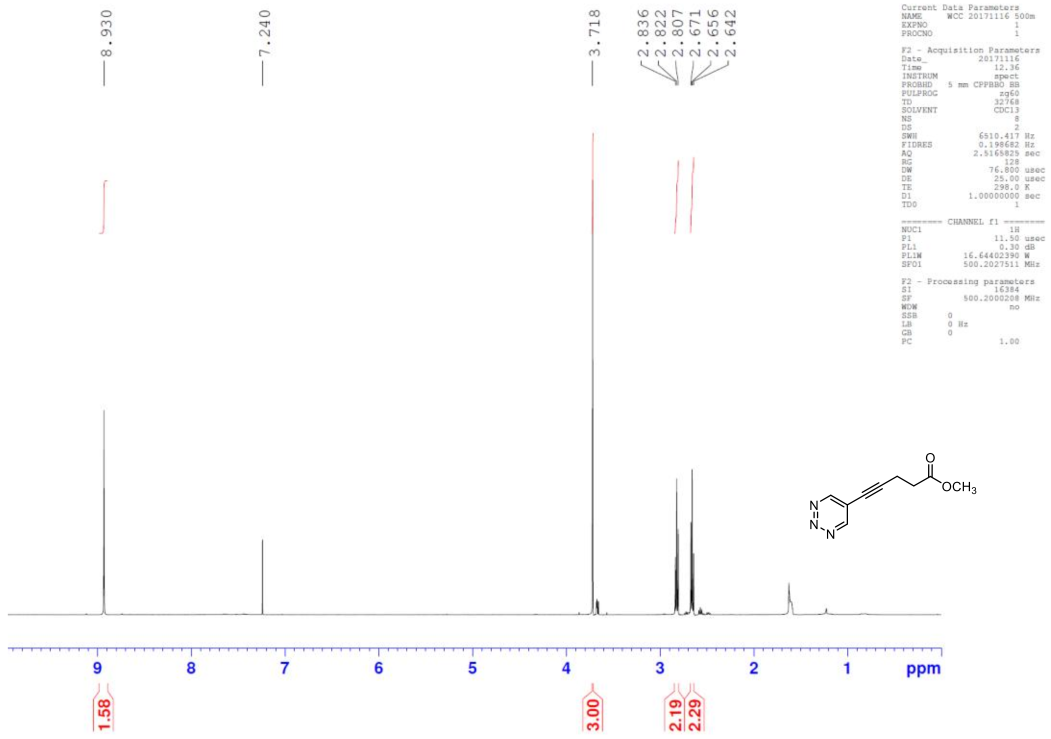

^{1}H NMR spectrum of **3x** ($CDCl_3$, 500 MHz)

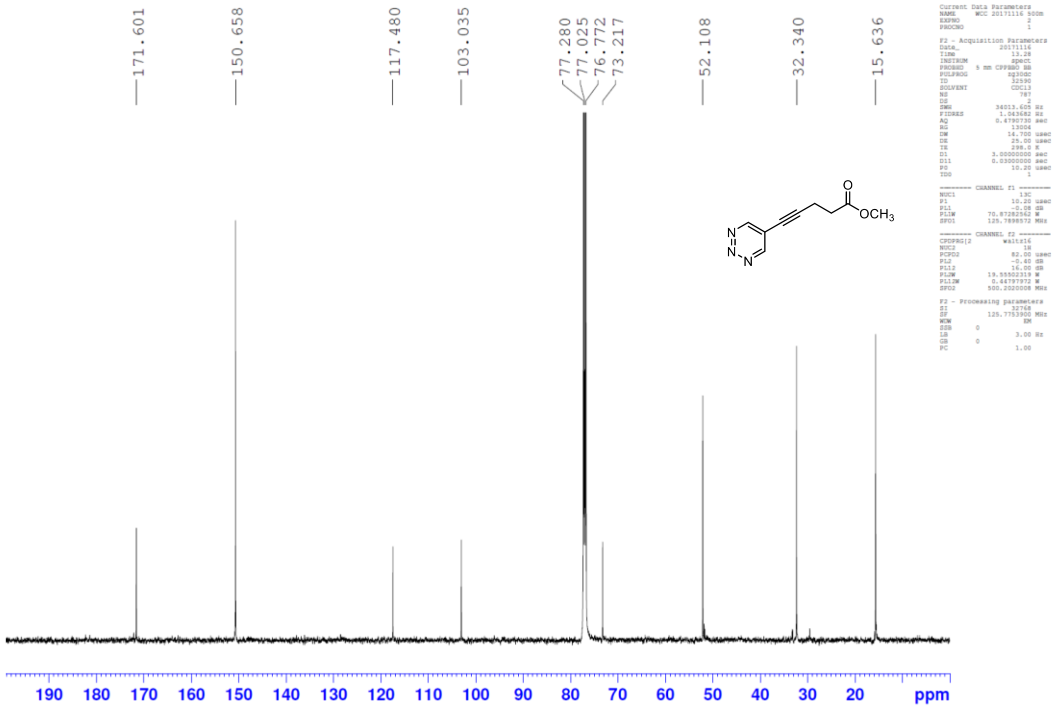

^{13}C{^{1}H} NMR spectrum of **3x** ($CDCl_3$, 125 MHz)

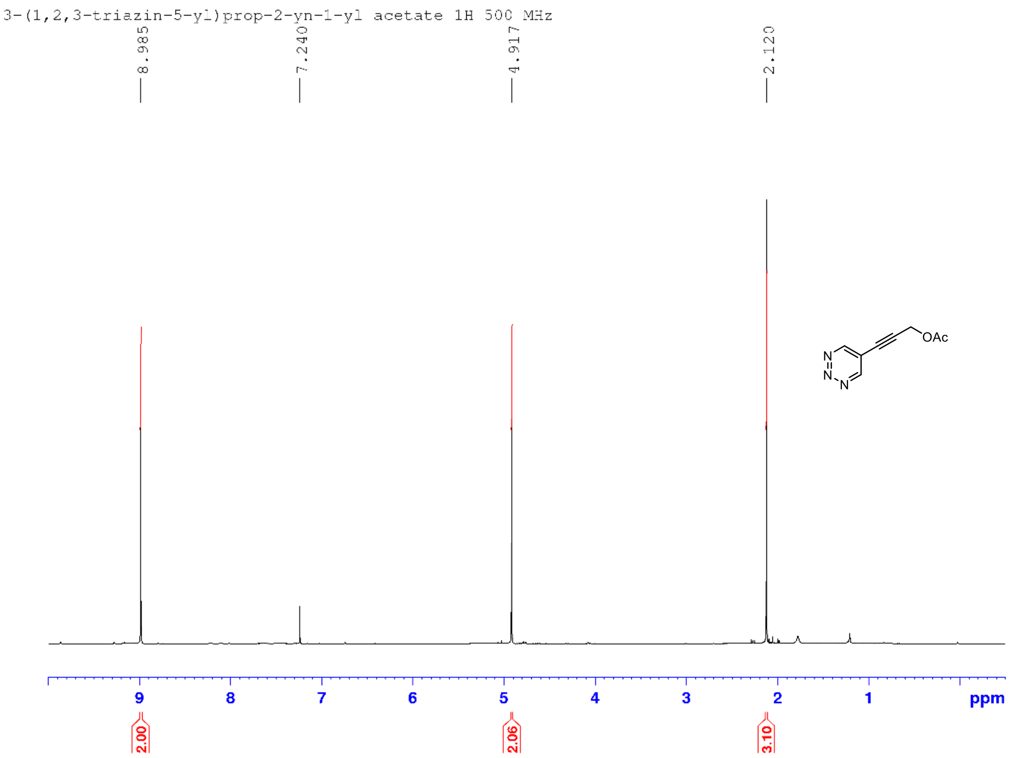

^{1}H NMR spectrum of **3y** ($CDCl_3$, 500 MHz)

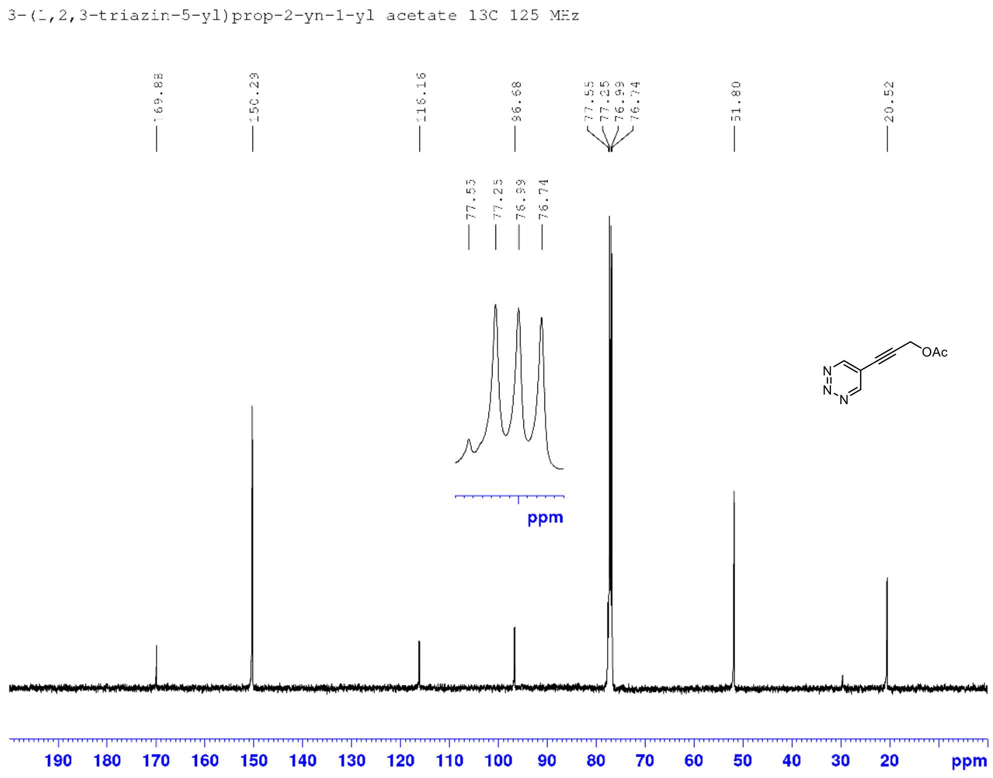

$^{13}C\{^1H\}$ NMR spectrum of **3y** ($CDCl_3$, 125 MHz)

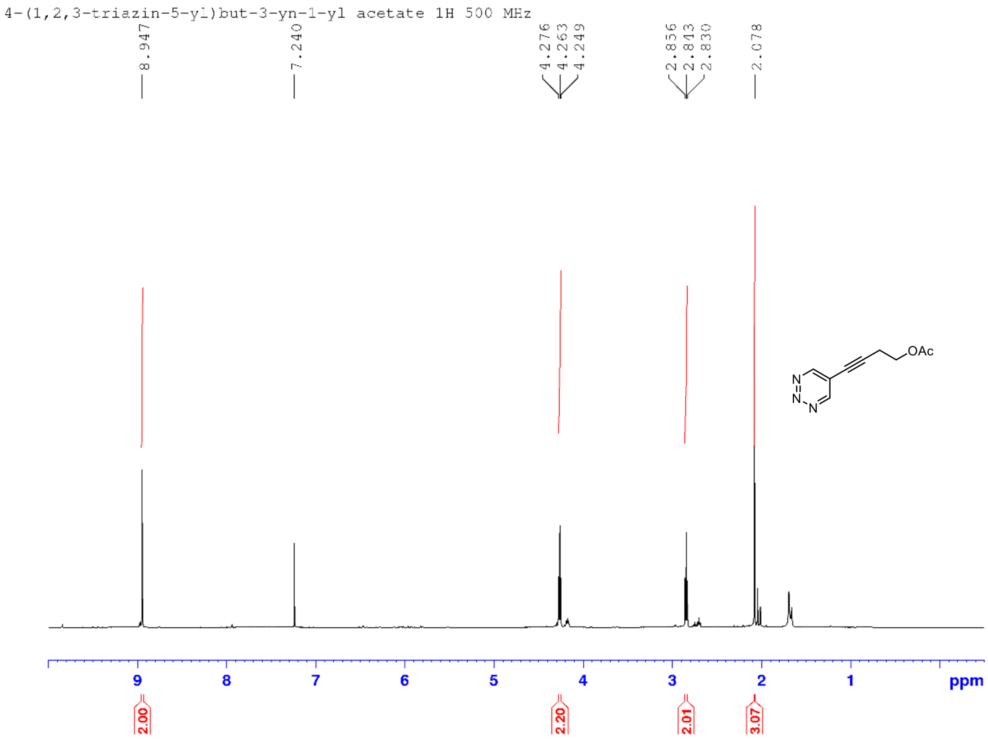

^{1}H NMR spectrum of **3z** ($CDCl_3$, 500 MHz)

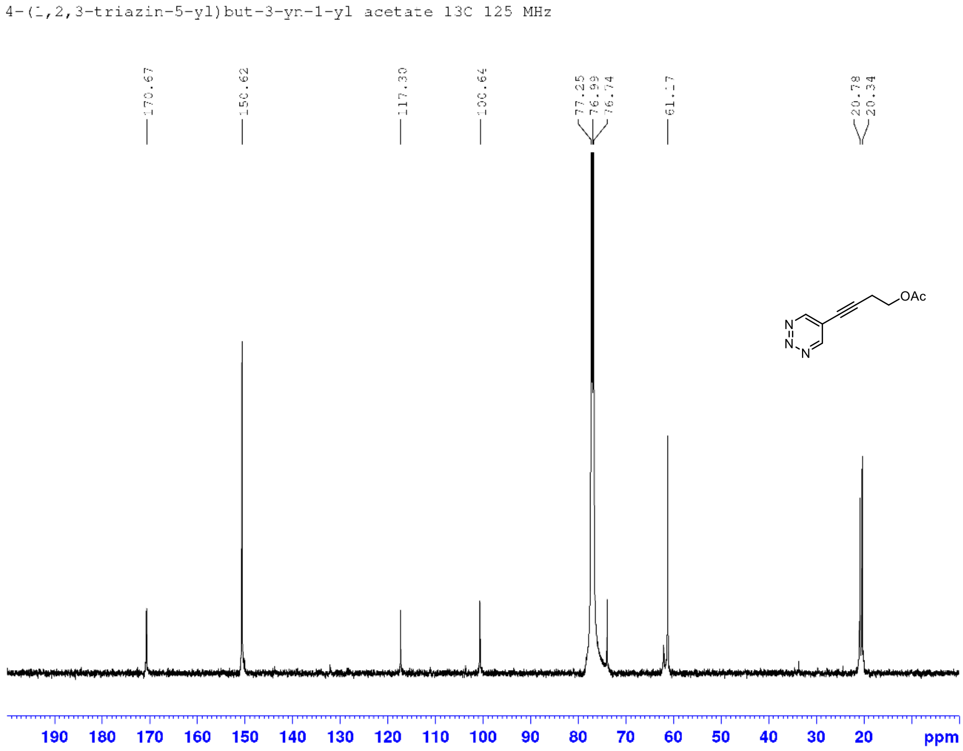

^{13}C{^{1}H} NMR spectrum of **3z** ($CDCl_3$, 125 MHz)

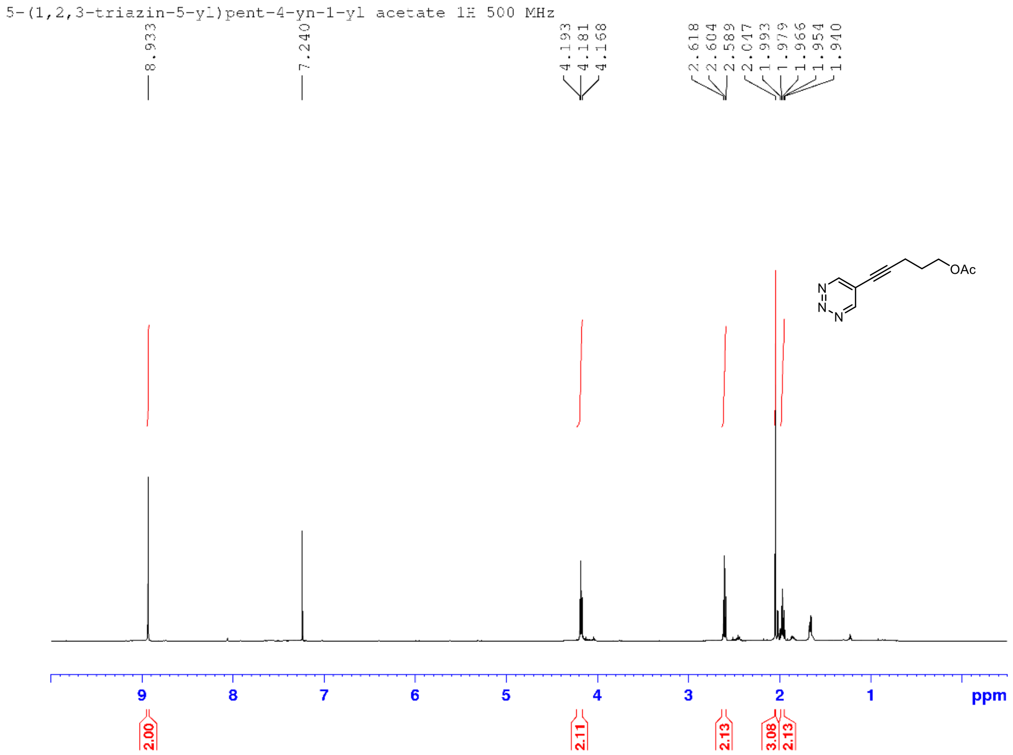

^{1}H NMR spectrum of **3a’** ($CDCl_3$, 500 MHz)

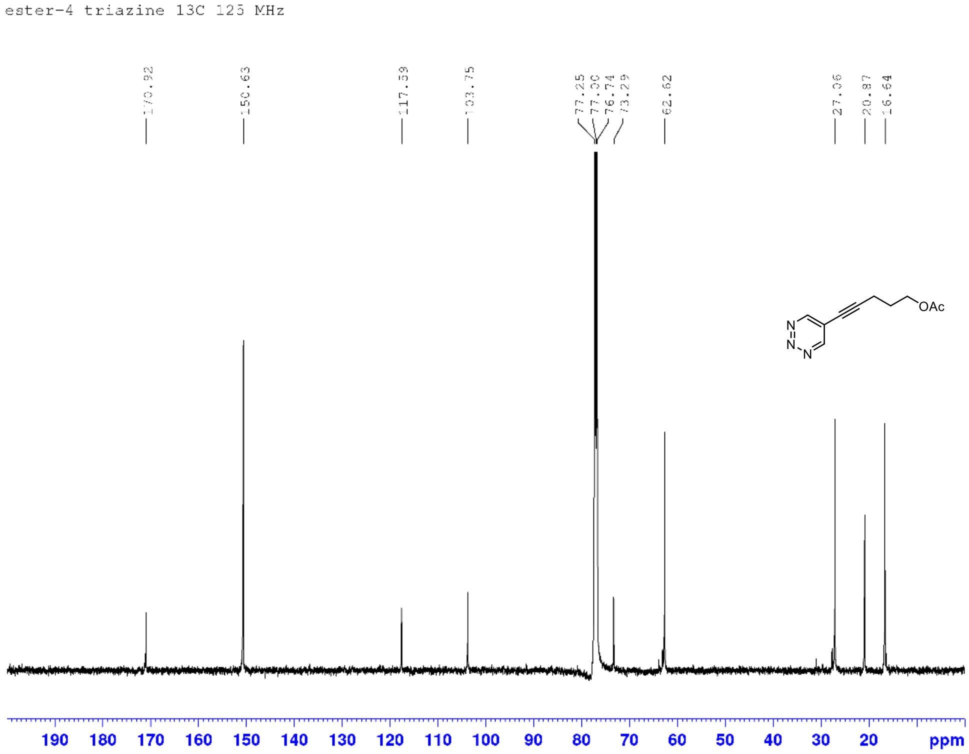

^{13}C{^{1}H} NMR spectrum of **3a’** ($CDCl_3$, 125 MHz)

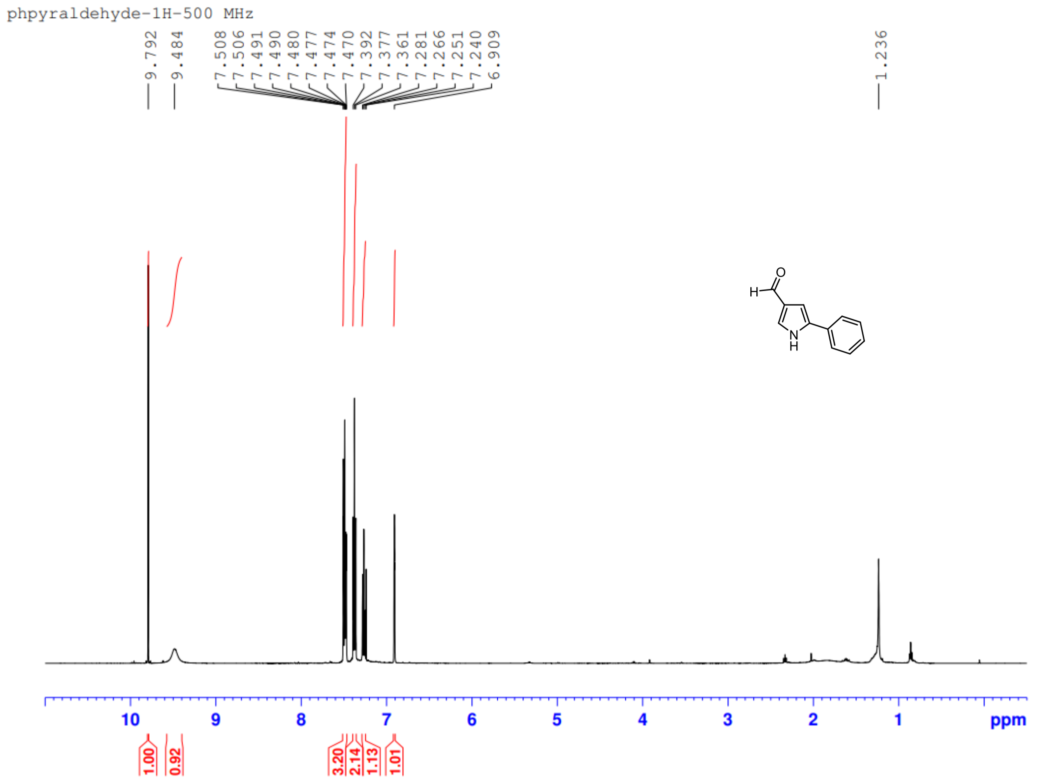

^{1}H NMR spectrum of **4a** ($CDCl_3$, 500 MHz)

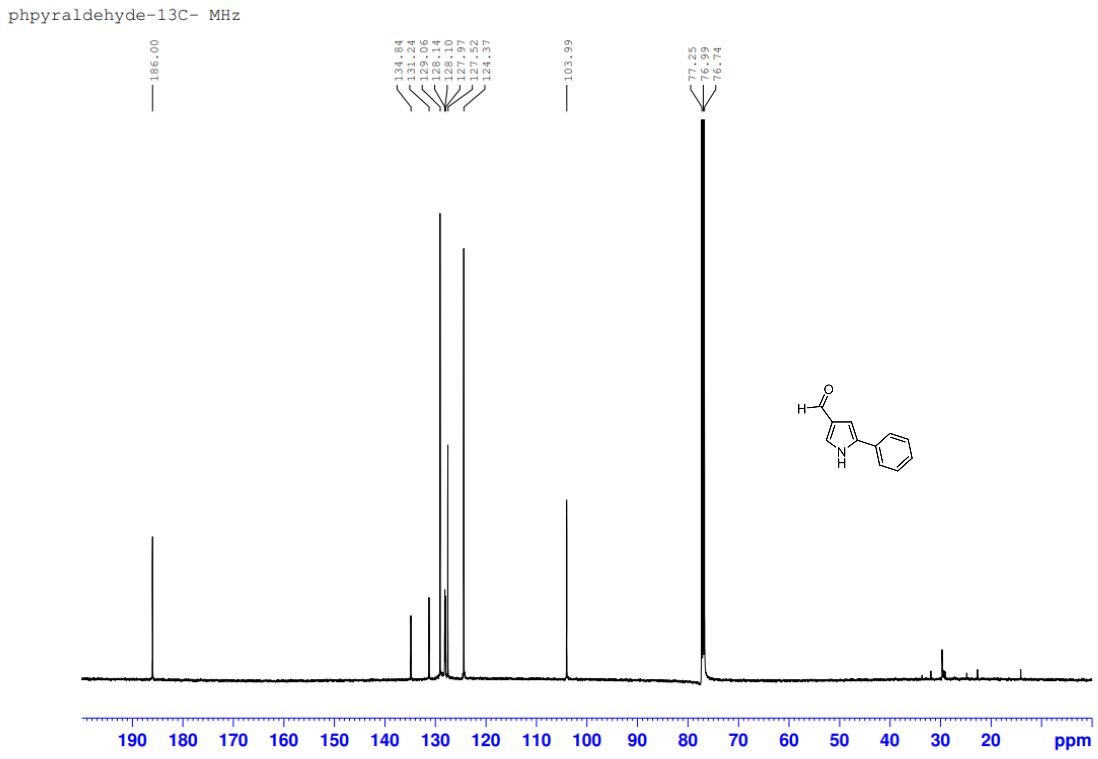

$^{13}C\{^{1}H\}$ NMR spectrum of **4a** ($CDCl_3$, 125 MHz)

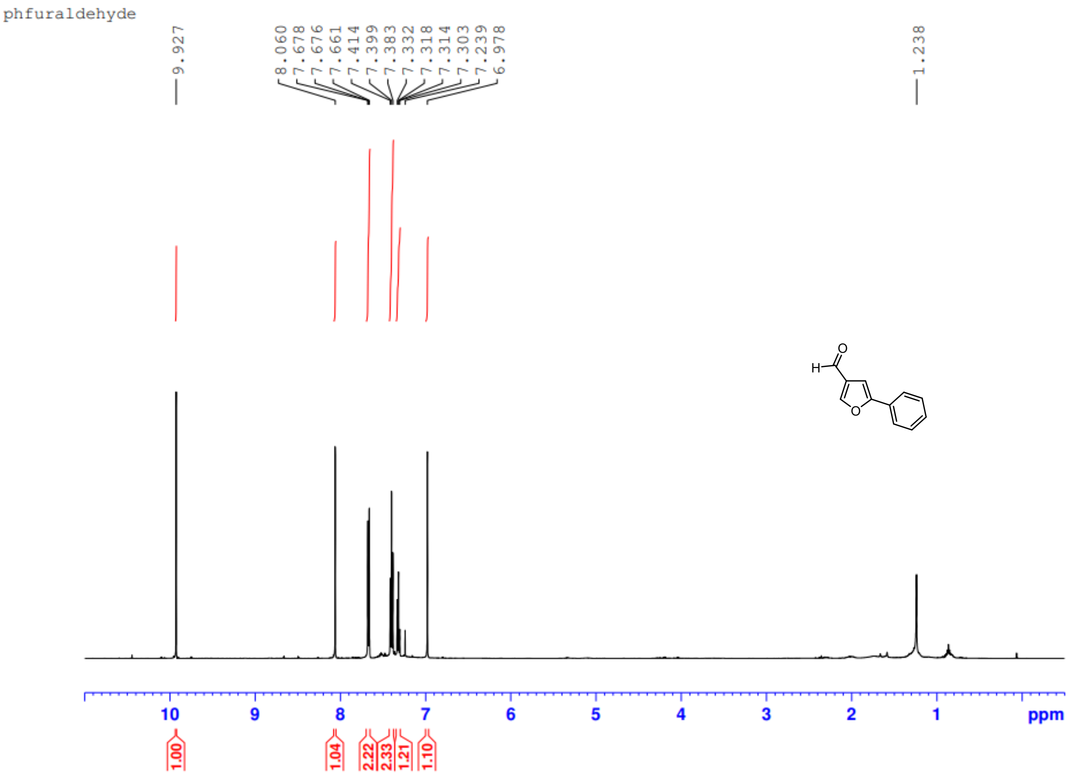

^{1}H NMR spectrum of **5a** ($CDCl_3$, 500 MHz)

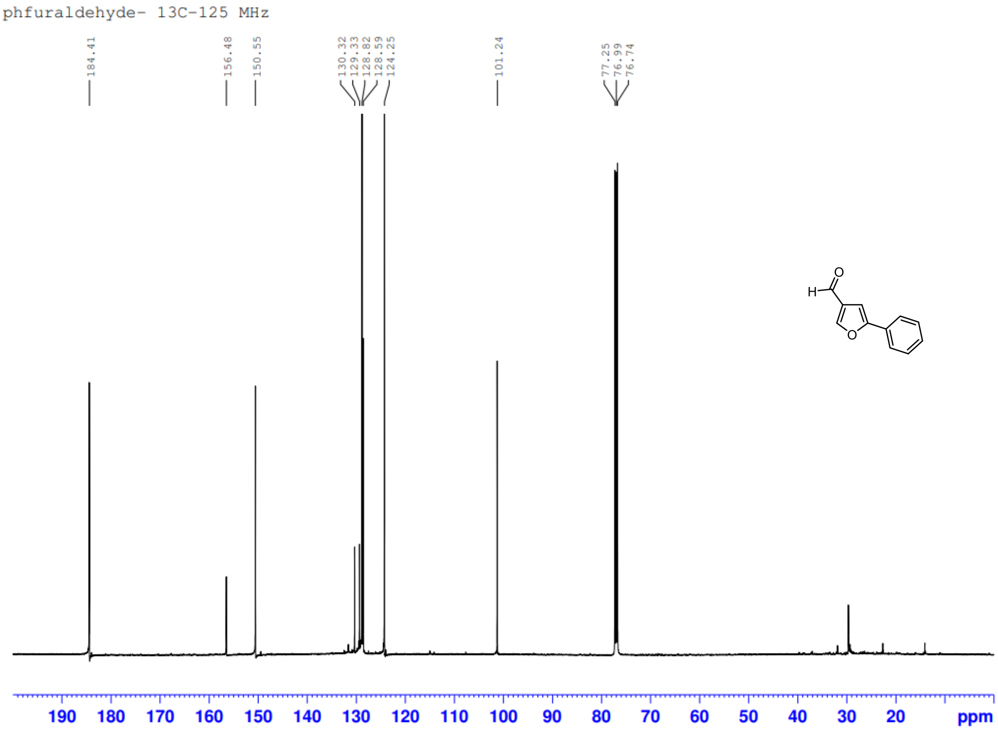

$^{13}C\{^{1}H\}$ NMR spectrum of **5a** ($CDCl_3$, 125 MHz)

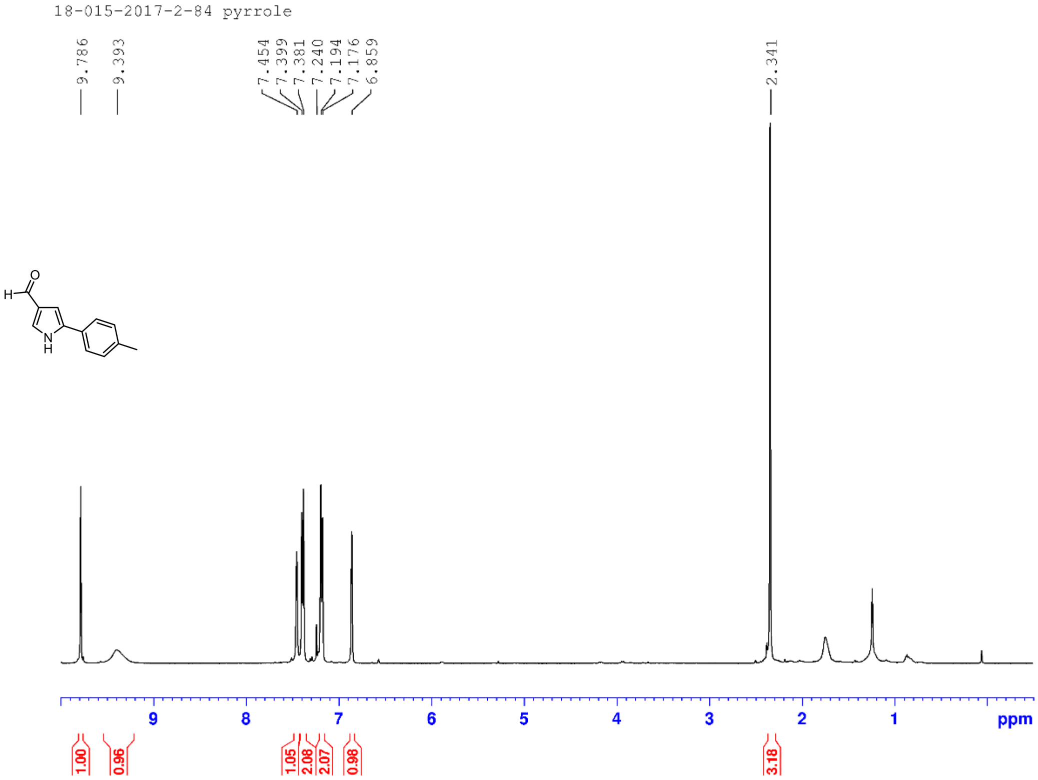

^{1}H NMR spectrum of **4b** ($CDCl_3$, 400 MHz)

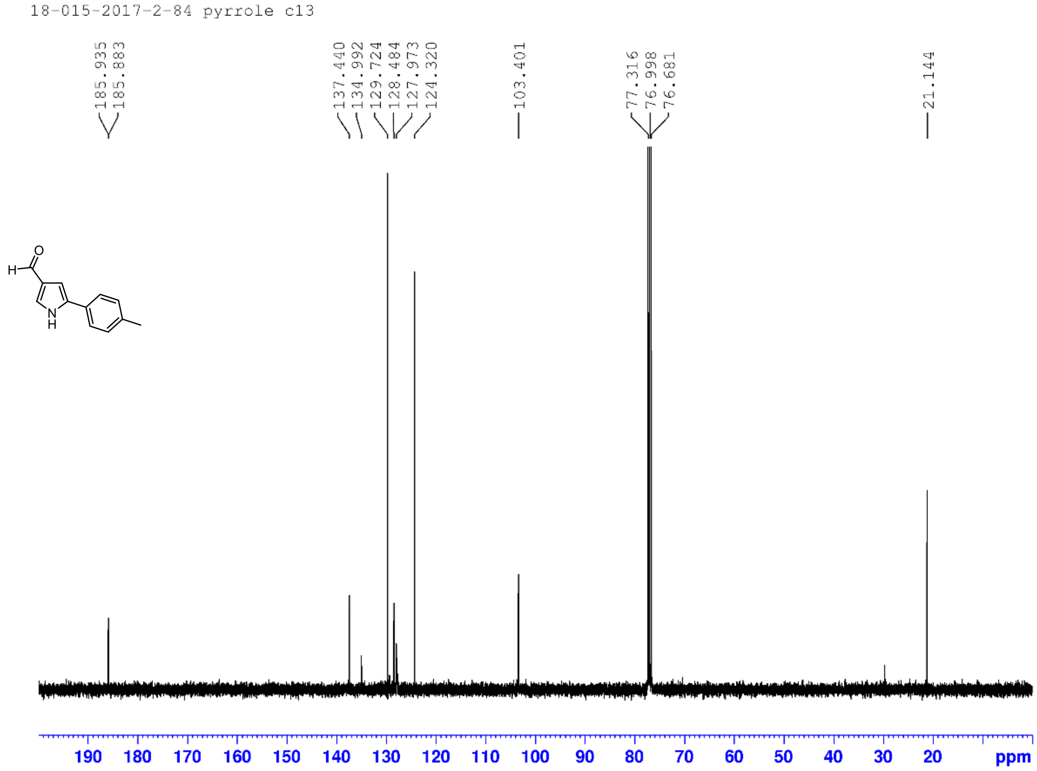

^{13}C{^{1}H} NMR spectrum of **4b** ($CDCl_3$, 100 MHz)

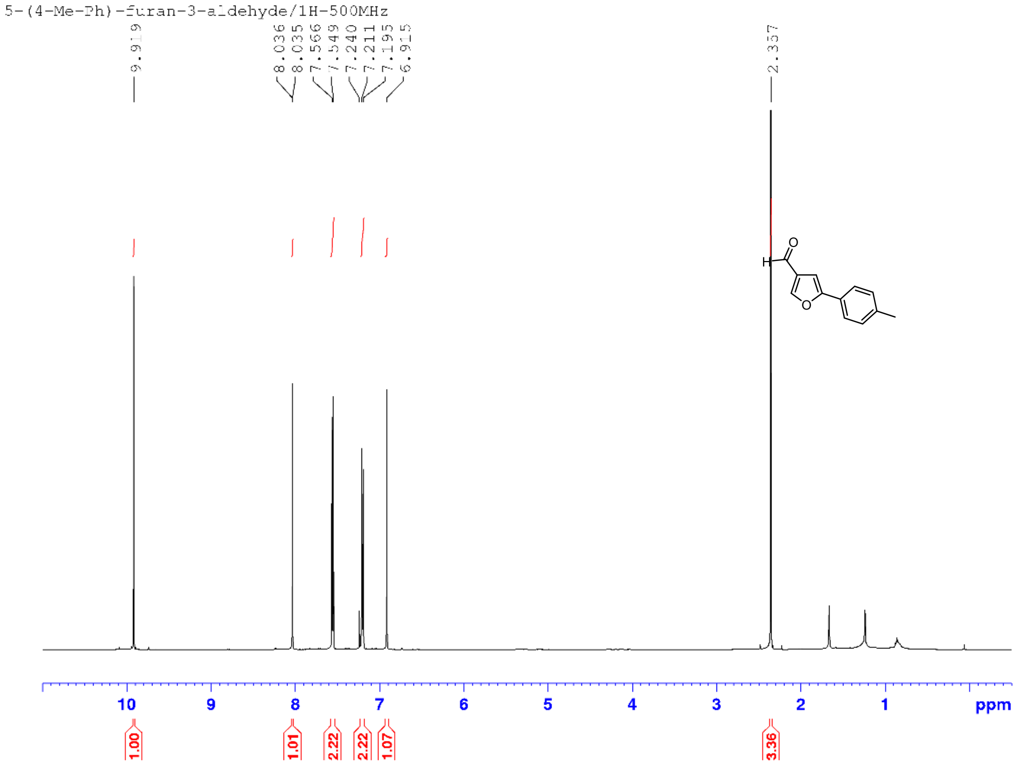

^{1}H NMR spectrum of **5b** ($CDCl_3$, 500 MHz)

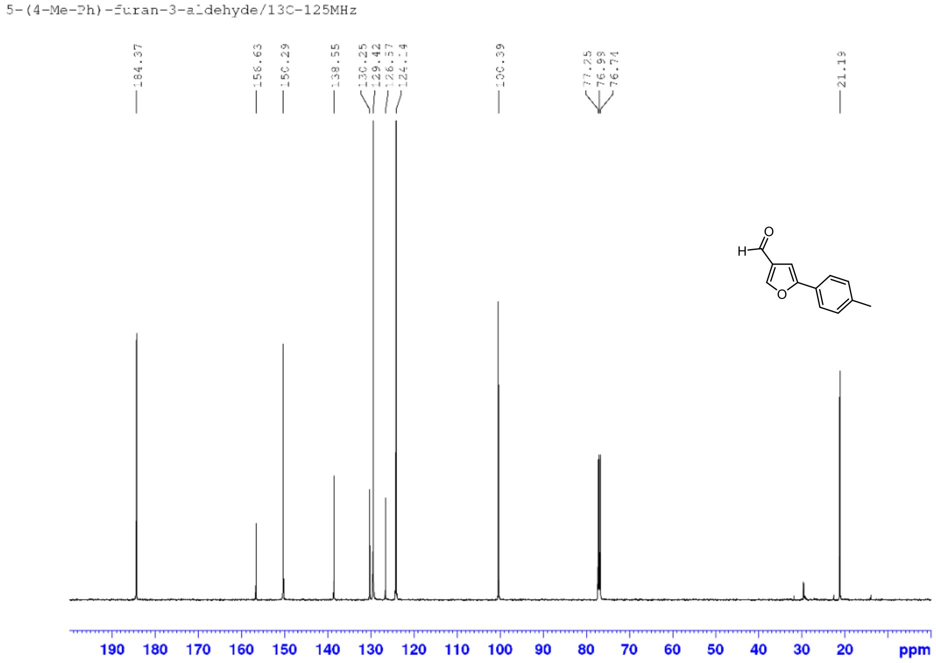

^{13}C{^{1}H} NMR spectrum of **5b** ($CDCl_3$, 125 MHz)

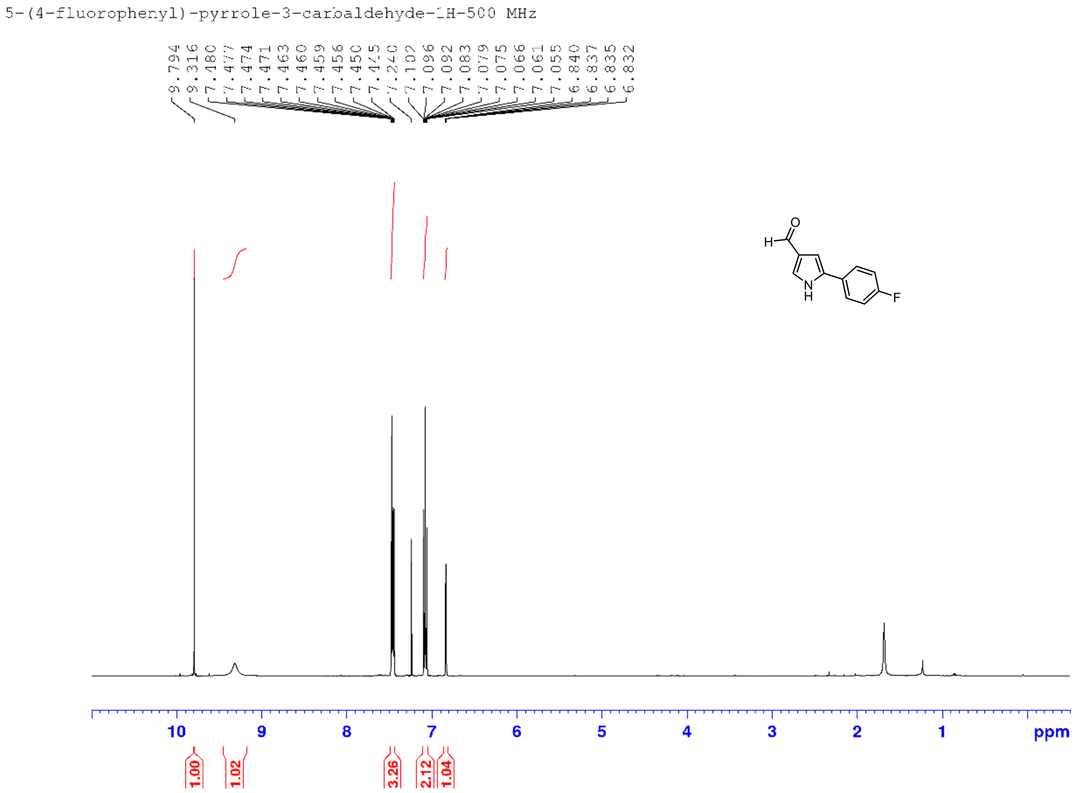

^{1}H NMR spectrum of **4c** ($CDCl_3$, 500 MHz)

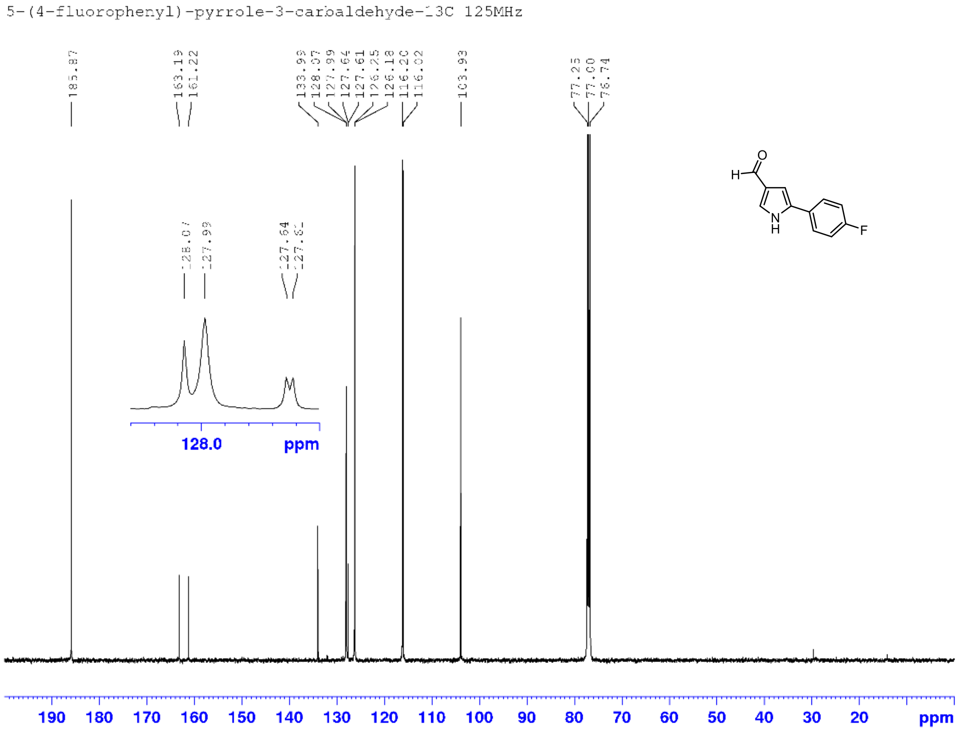

$^{13}C\{^{1}H\}$ NMR spectrum of **4c** ($CDCl_3$, 125 MHz)

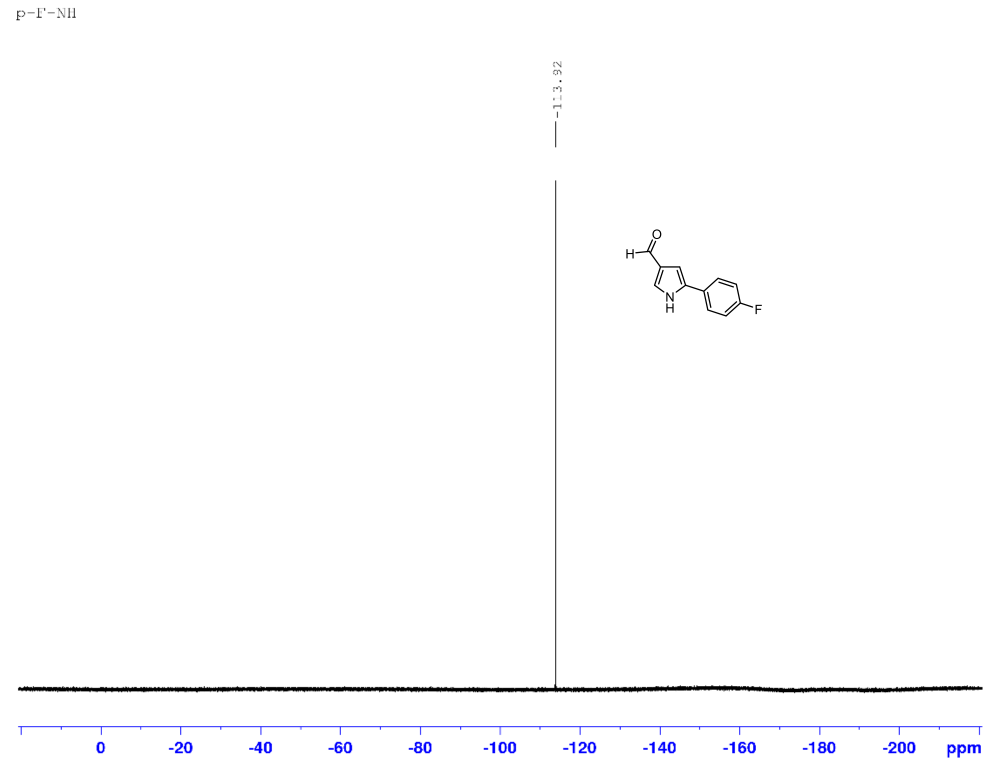

$^{19}F\{^{1}H\}$ NMR spectrum of **4c** ($CDCl_3$, 470 MHz)

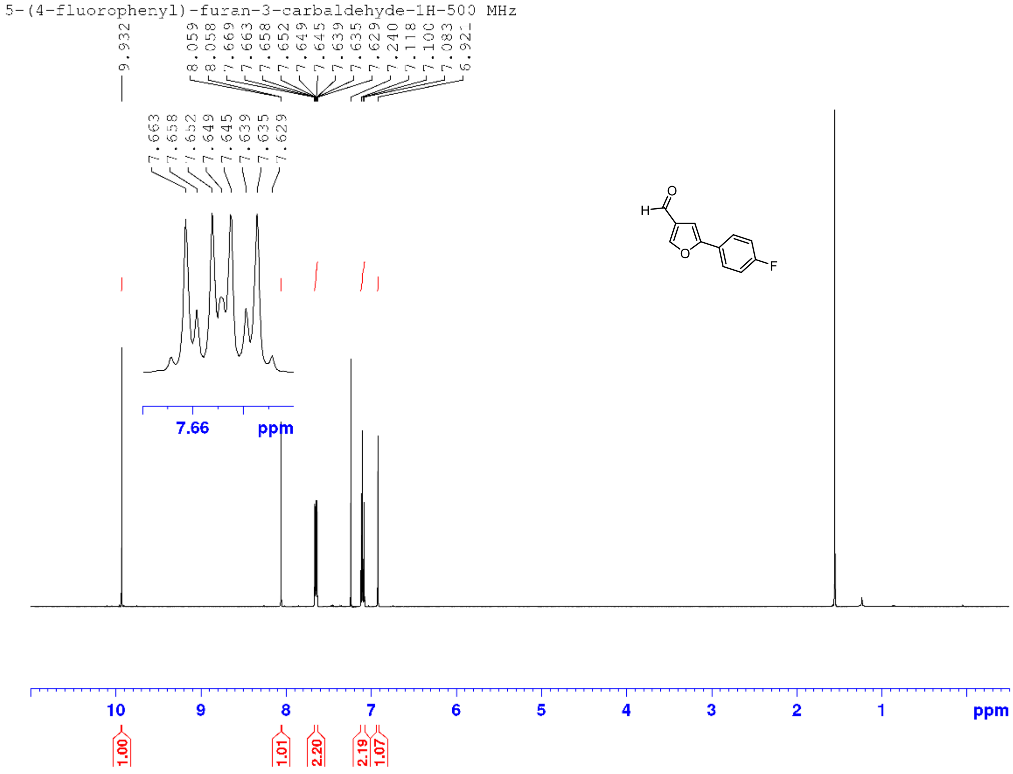

^{1}H NMR spectrum of **5c** ($CDCl_3$, 500 MHz)

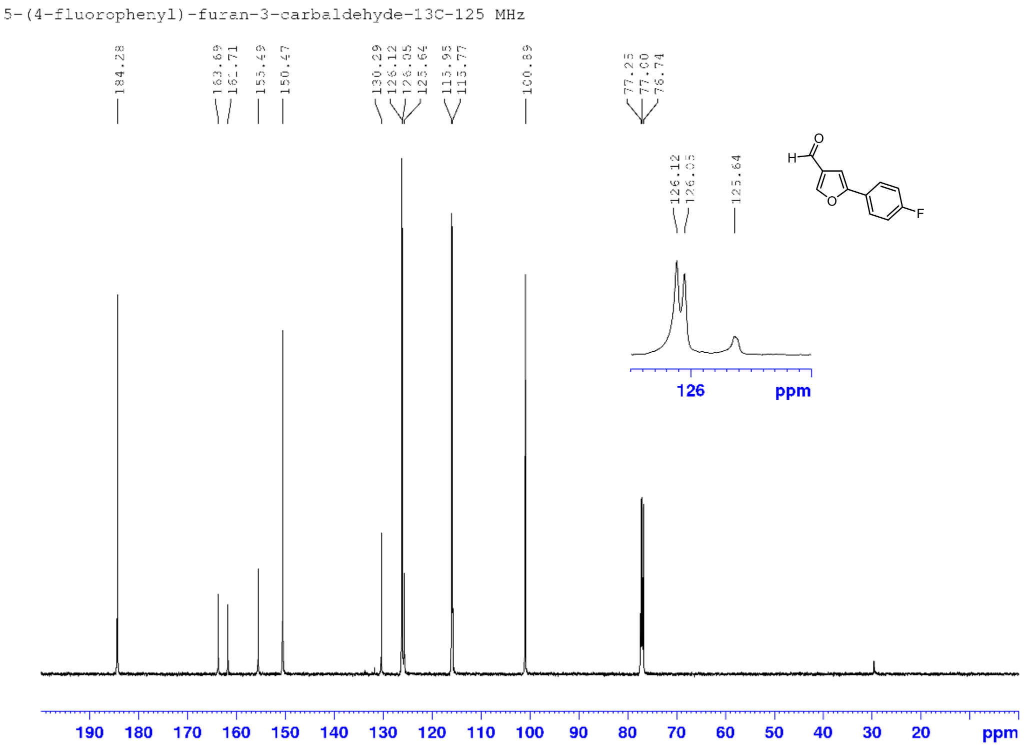

$^{13}C\{^{1}H\}$ NMR spectrum of **5c** ($CDCl_3$, 125 MHz)

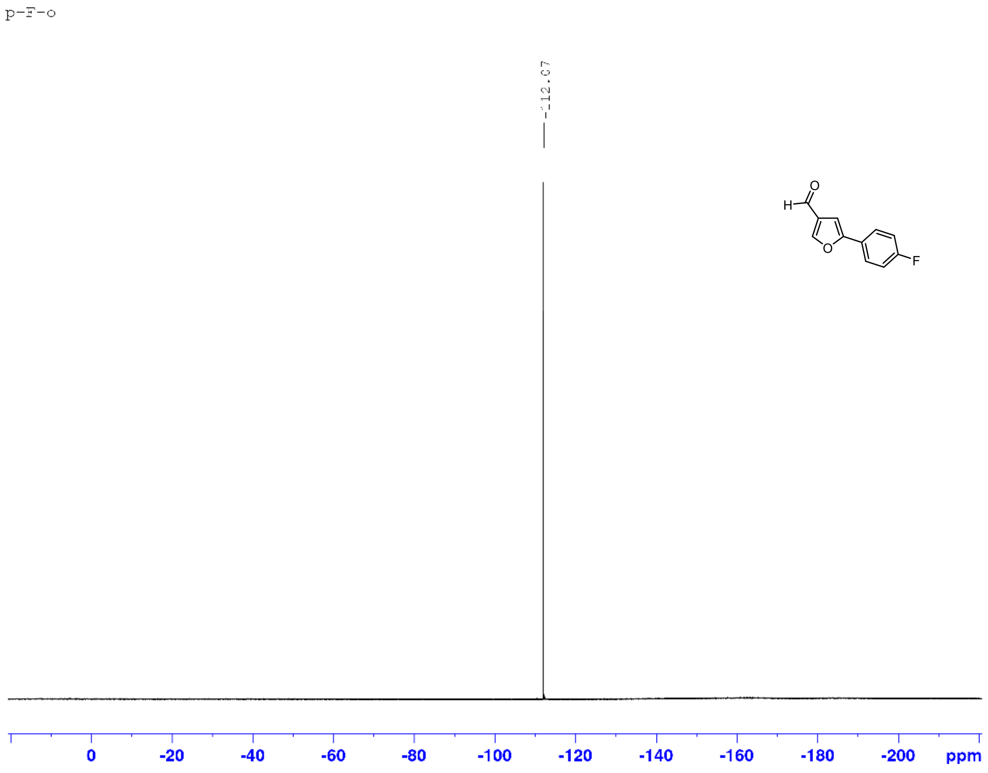

$^{19}F\{^{1}H\}$ NMR spectrum of **5c** ($CDCl_3$, 470 MHz)

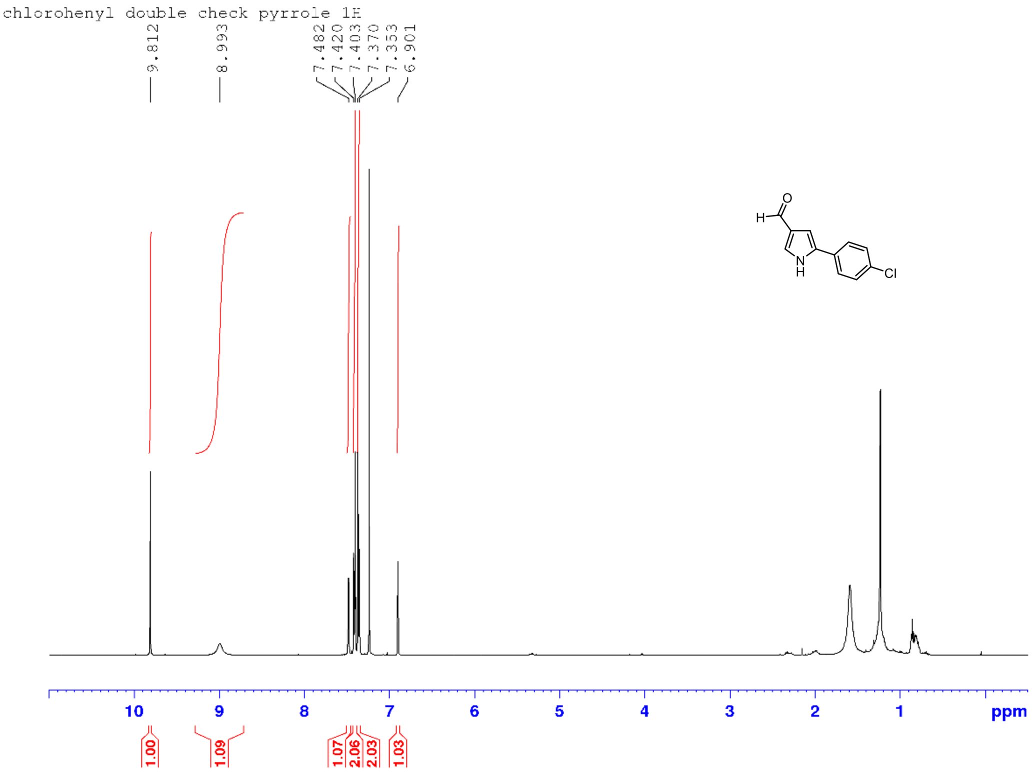

^{1}H NMR spectrum of **4d** ($CDCl_3$, 500 MHz)

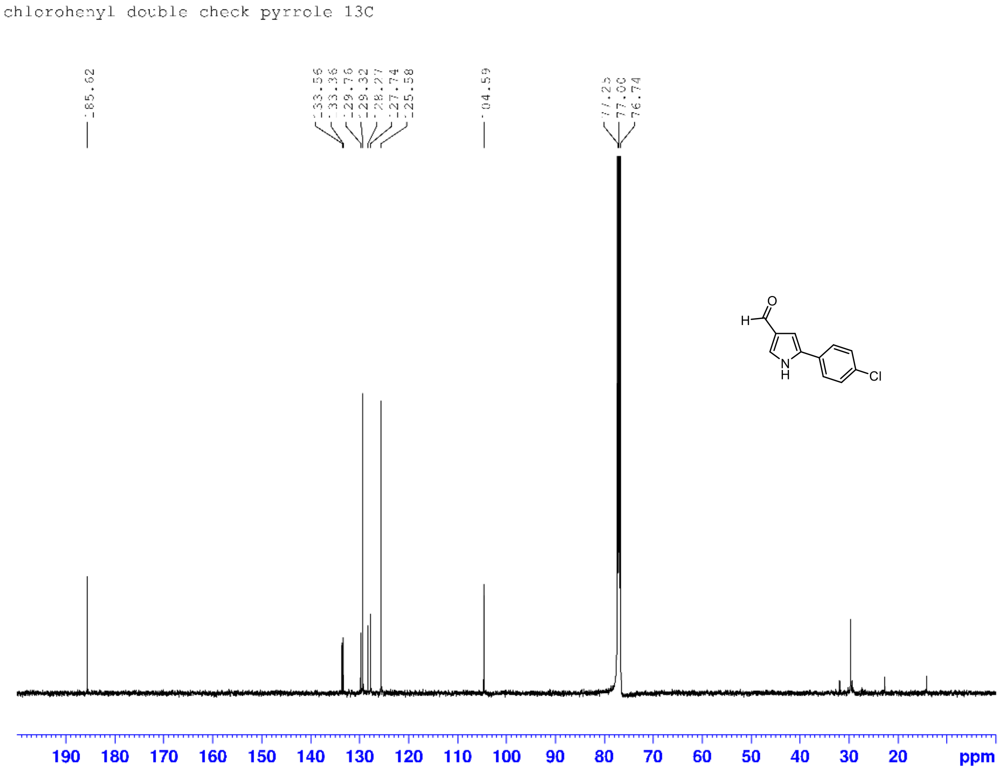

$^{13}C\{^{1}H\}$ NMR spectrum of **4d** ($CDCl_3$, 125 MHz)

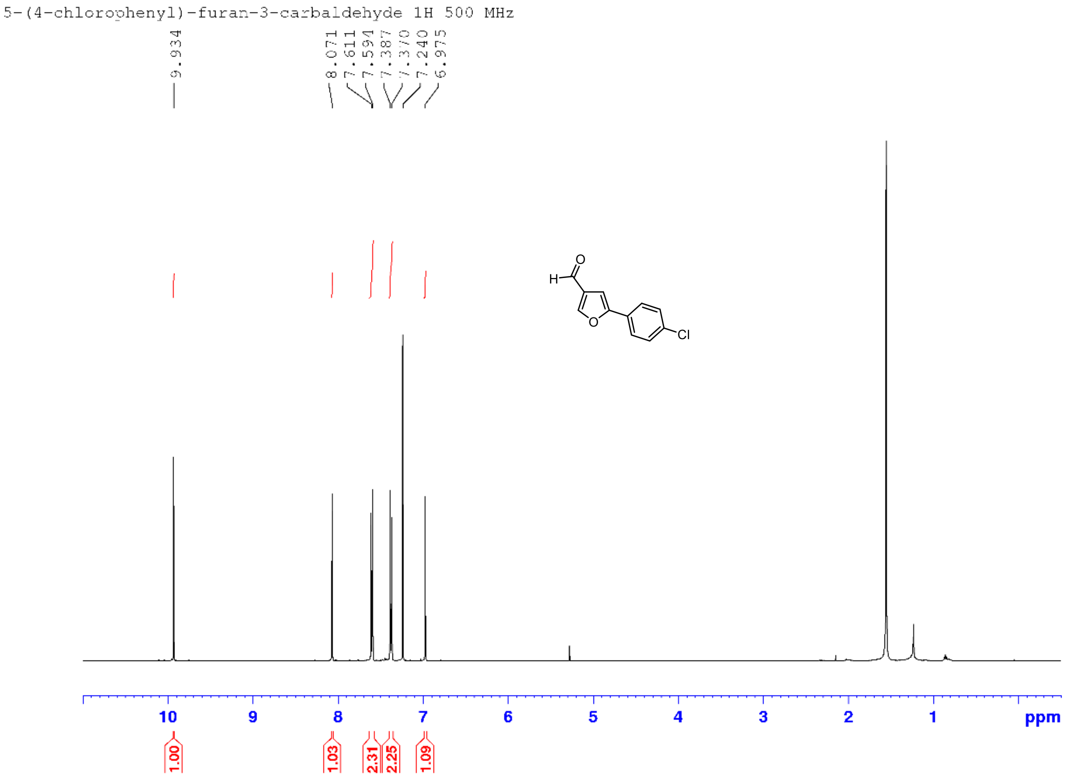

^{1}H NMR spectrum of **5d** ($CDCl_3$, 500 MHz)

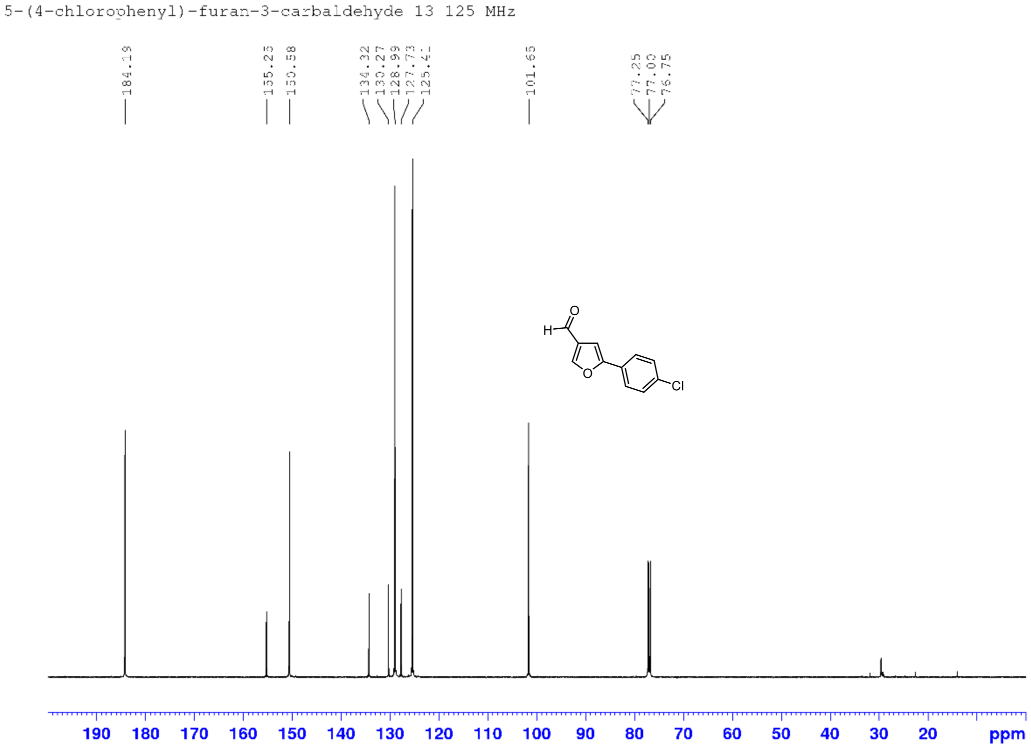

^{13}C{^{1}H} NMR spectrum of **5d** ($CDCl_3$, 125 MHz)

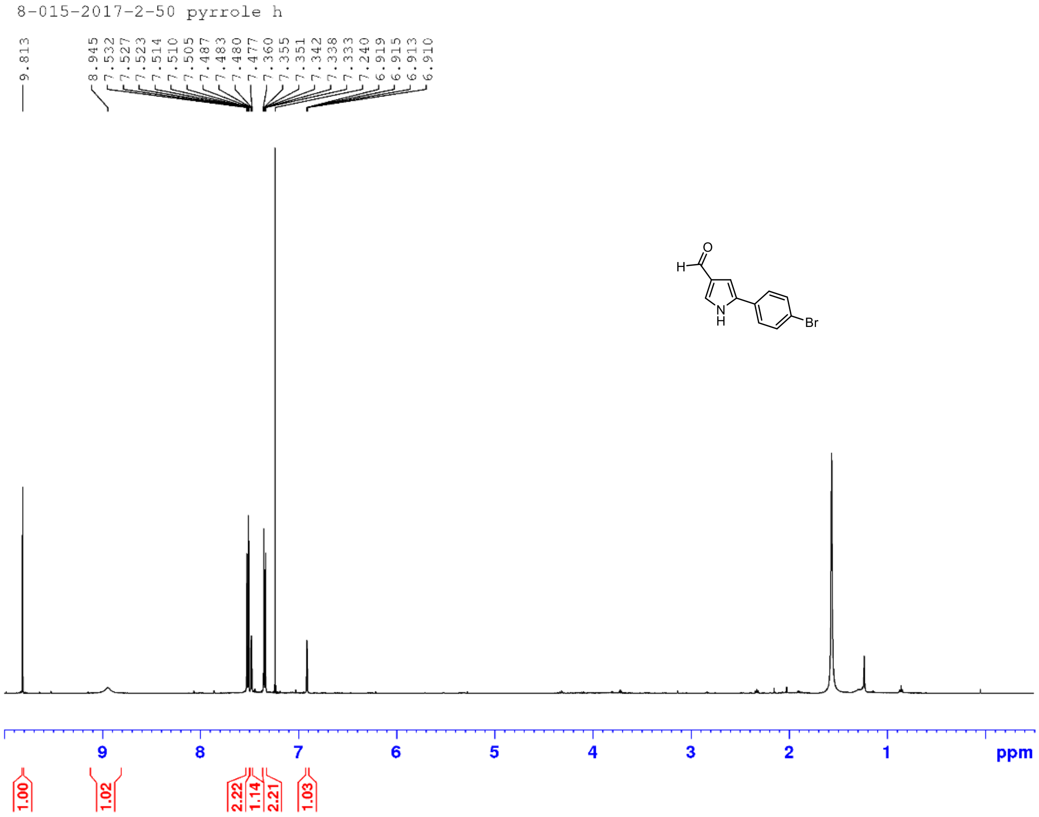

^{1}H NMR spectrum of **4e** ($CDCl_3$, 500 MHz)

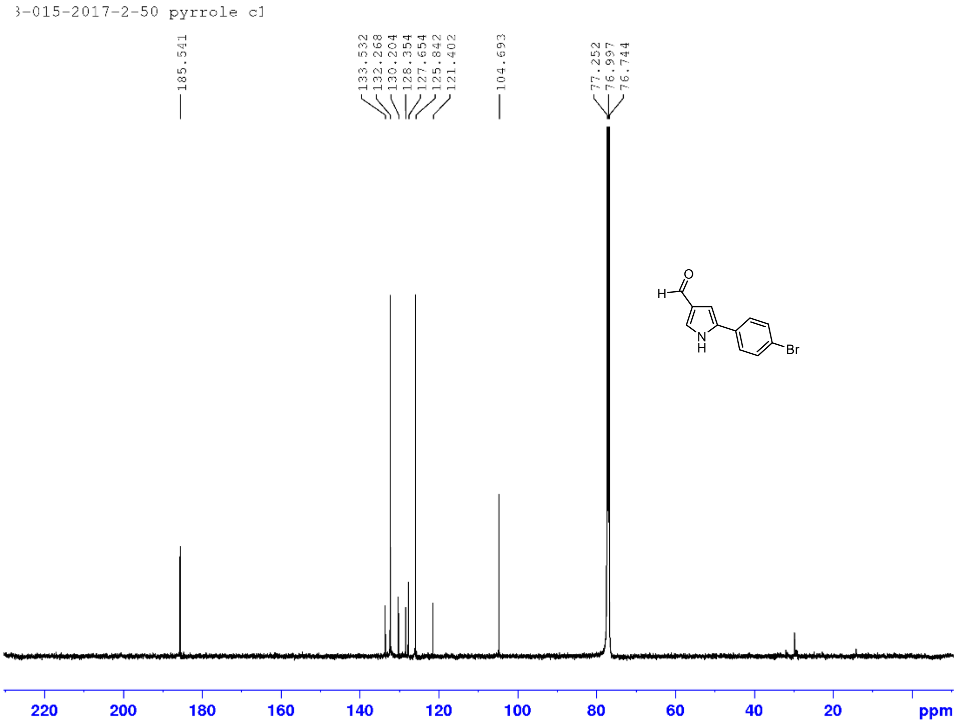

^{13}C{^{1}H} NMR spectrum of **4e** ($CDCl_3$, 125 MHz)

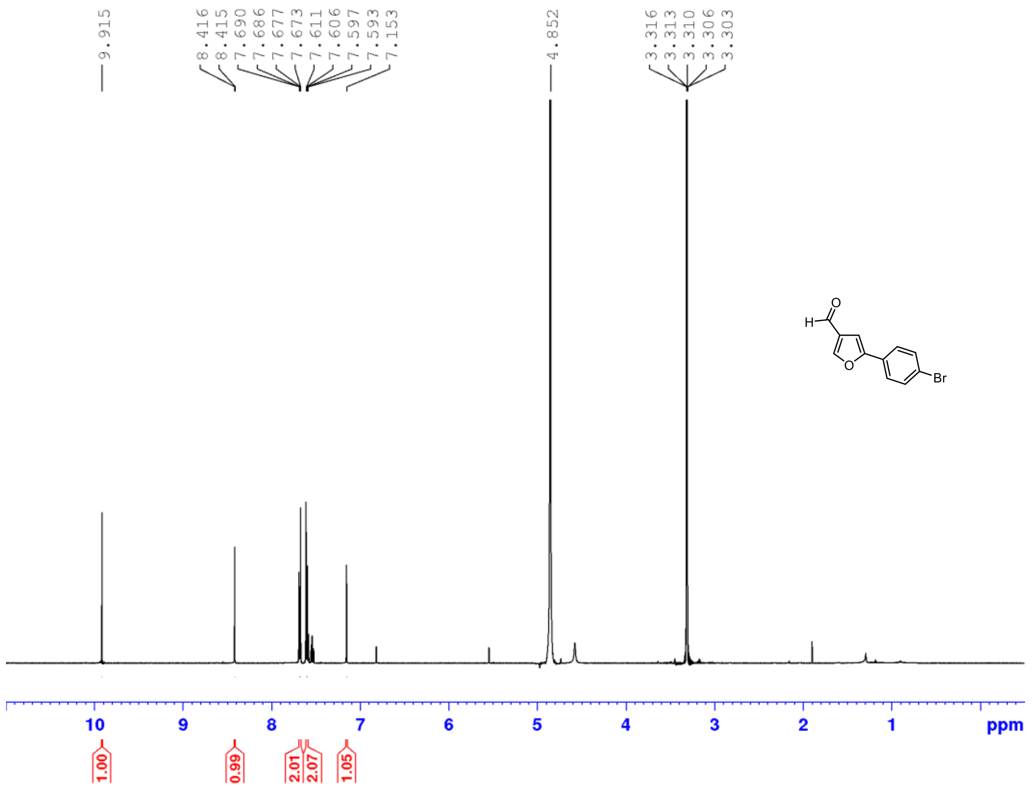

^{1}H NMR spectrum of **5e** (CD_3OD, 500 MHz)

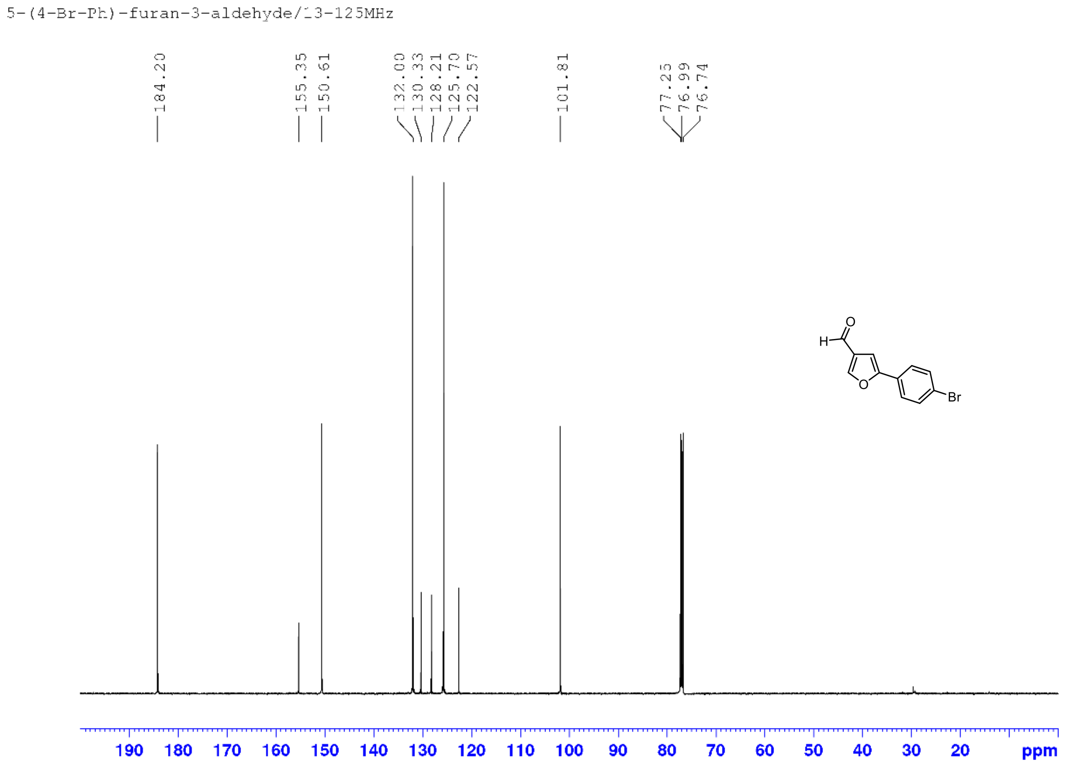

$^{13}C\{^{1}H\}$ NMR spectrum of **5e** ($CDCl_3$, 125 MHz)

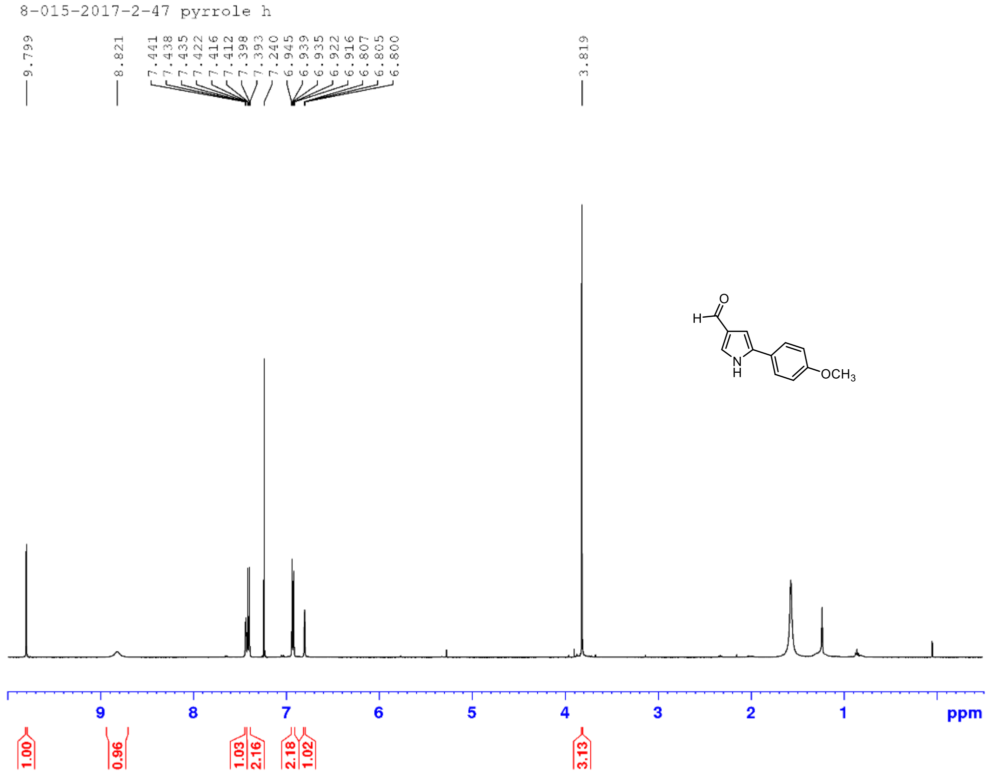

^{1}H NMR spectrum of **4f** ($CDCl_3$, 500 MHz)

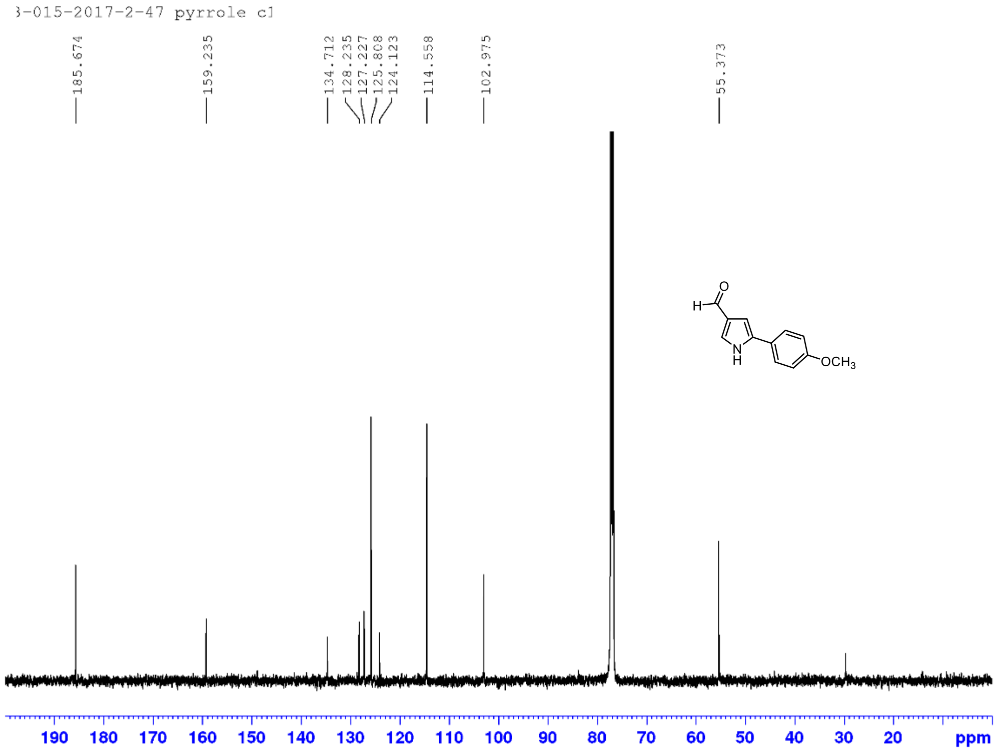

^{13}C{^{1}H} NMR spectrum of **4f** ($CDCl_3$, 125 MHz)

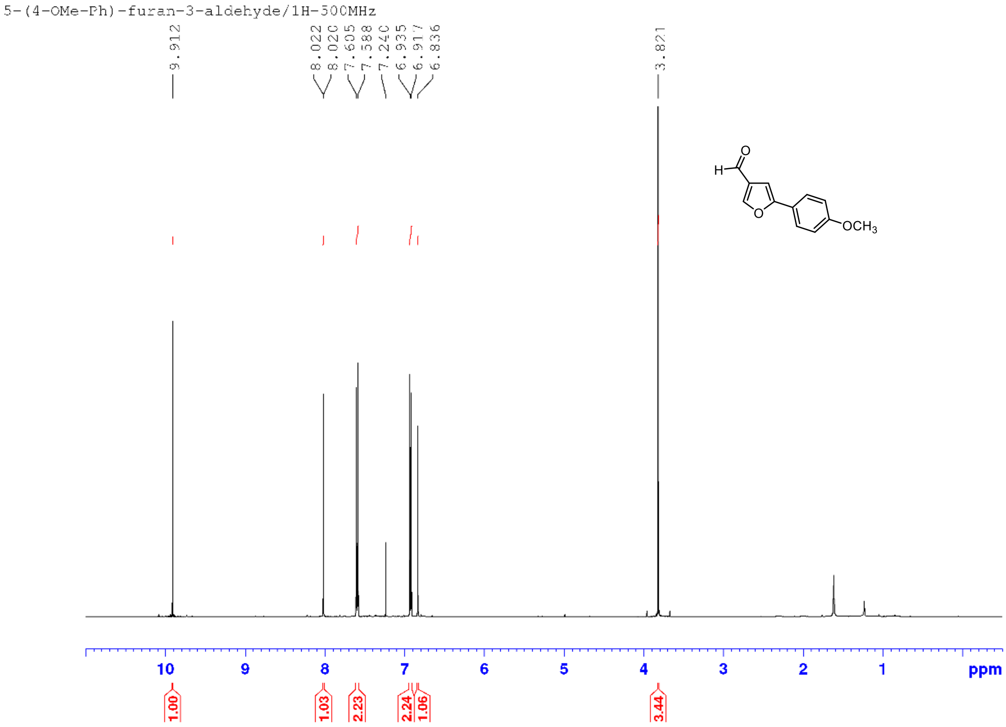

^{1}H NMR spectrum of **5f** ($CDCl_3$, 500 MHz)

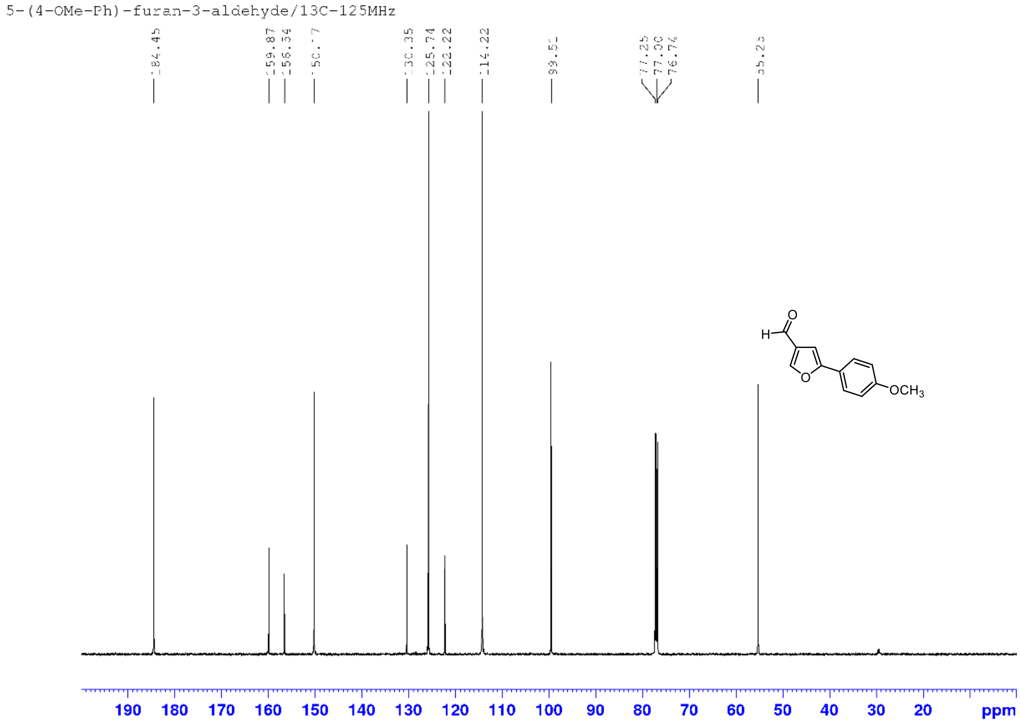

^{13}C{^{1}H} NMR spectrum of **5f** ($CDCl_3$, 125 MHz)

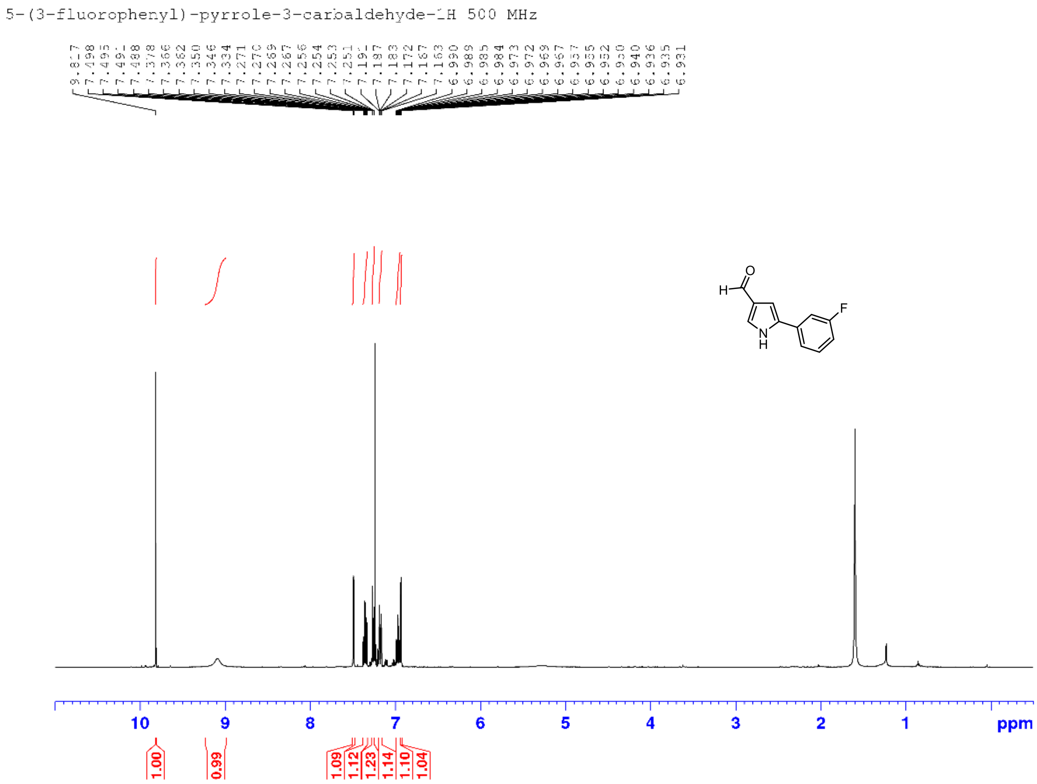

^1H NMR spectrum of **4g** ($CDCl_3$, 500 MHz)

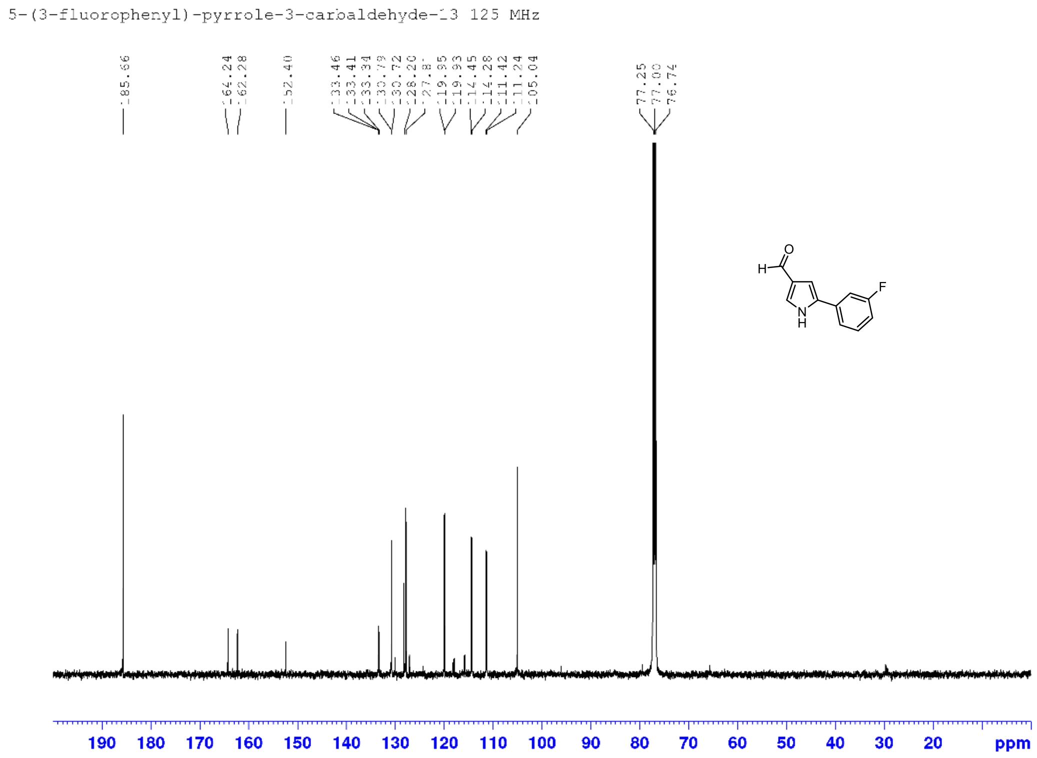

$^{13}C\{^1H\}$ NMR spectrum of **4g** ($CDCl_3$, 125 MHz)

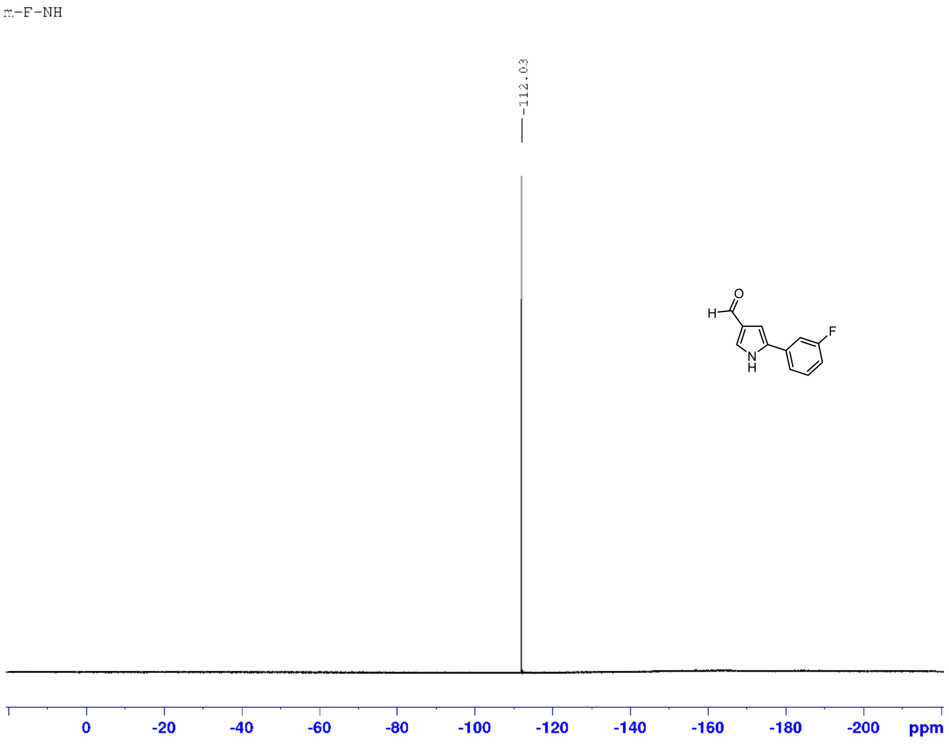

$^{19}F\{^{1}H\}$ NMR spectrum of **4g** ($CDCl_3$, 470 MHz)

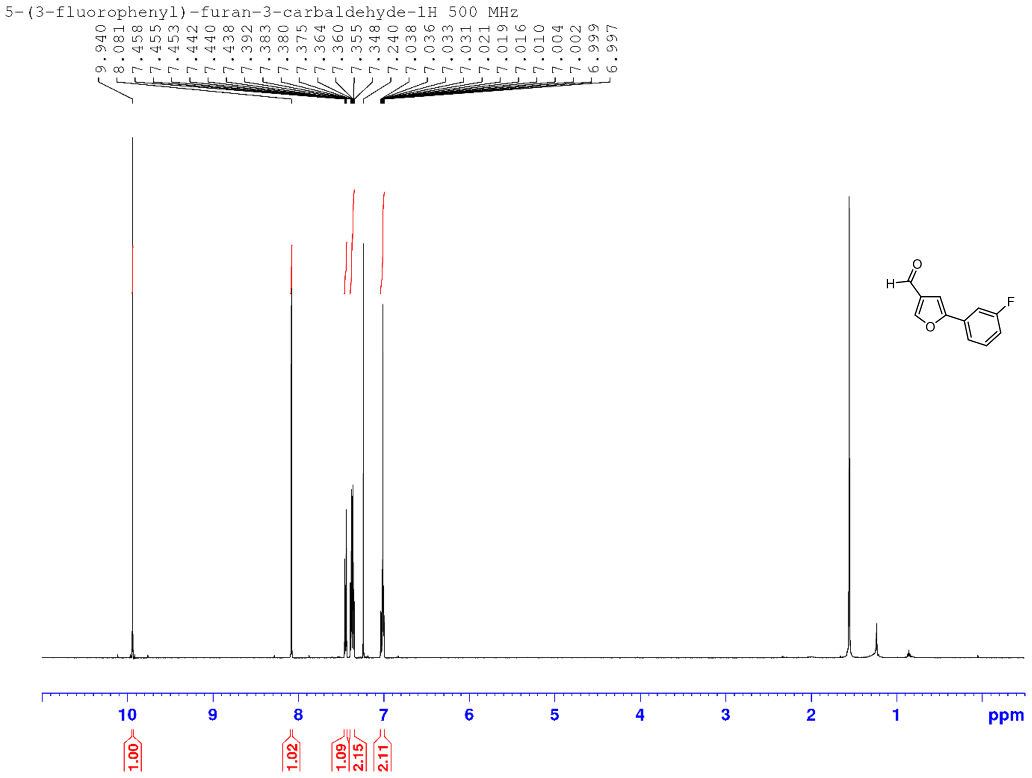

^{1}H NMR spectrum of **5g** ($CDCl_3$, 500 MHz)

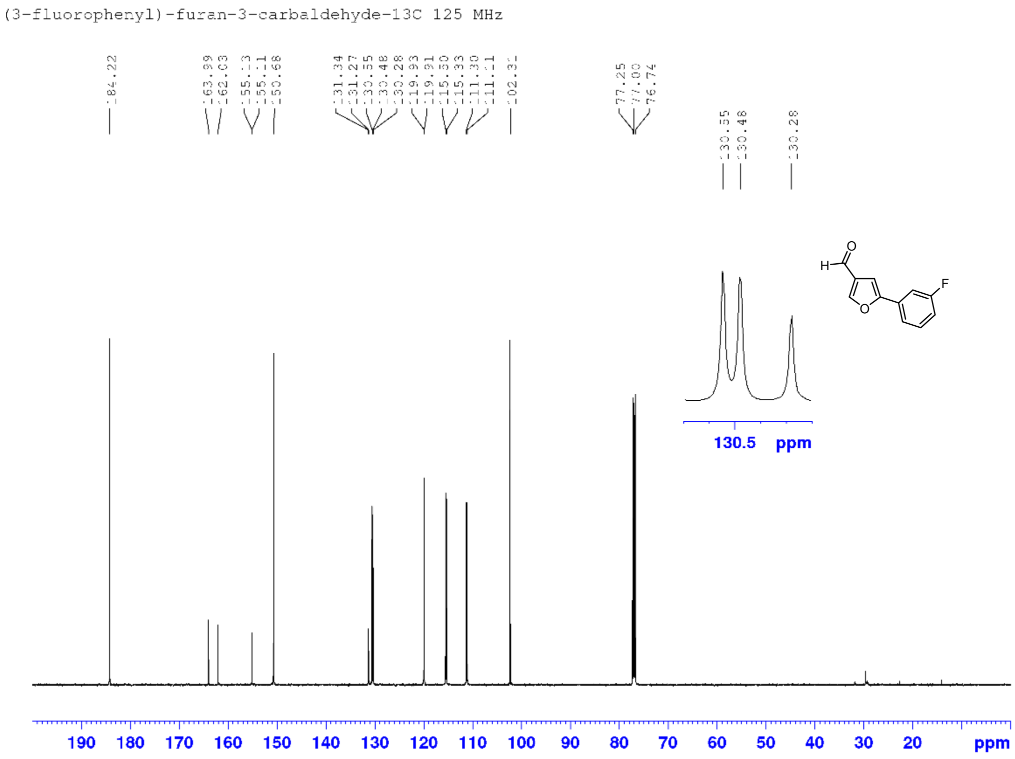

$^{13}C\{^{1}H\}$ NMR spectrum of **5g** ($CDCl_3$, 125 MHz)

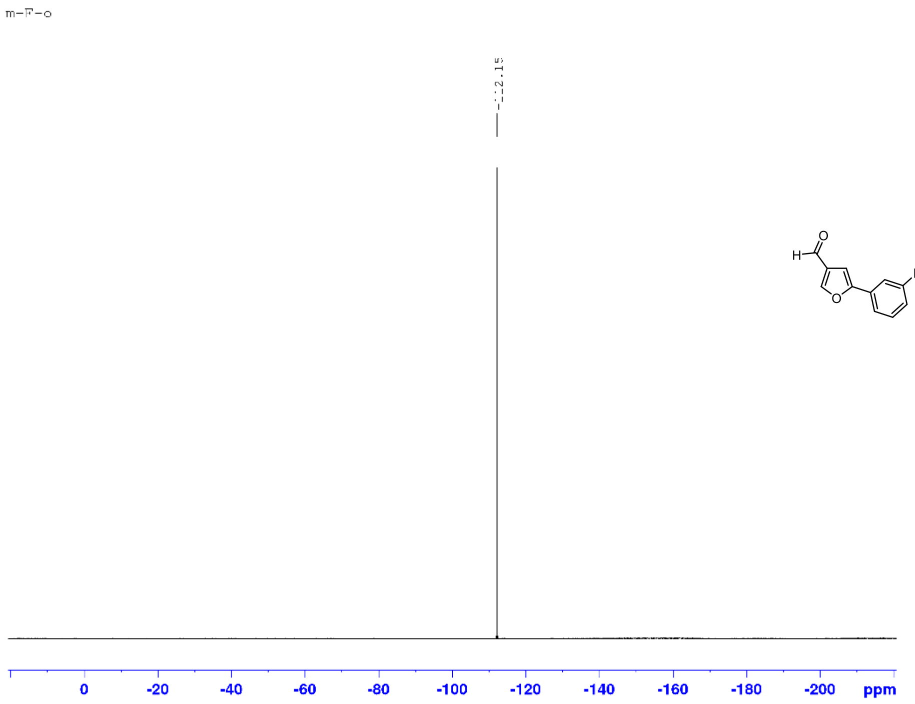

$^{19}F\{^{1}H\}$ NMR spectrum of **5g** ($CDCl_3$, 470 MHz)

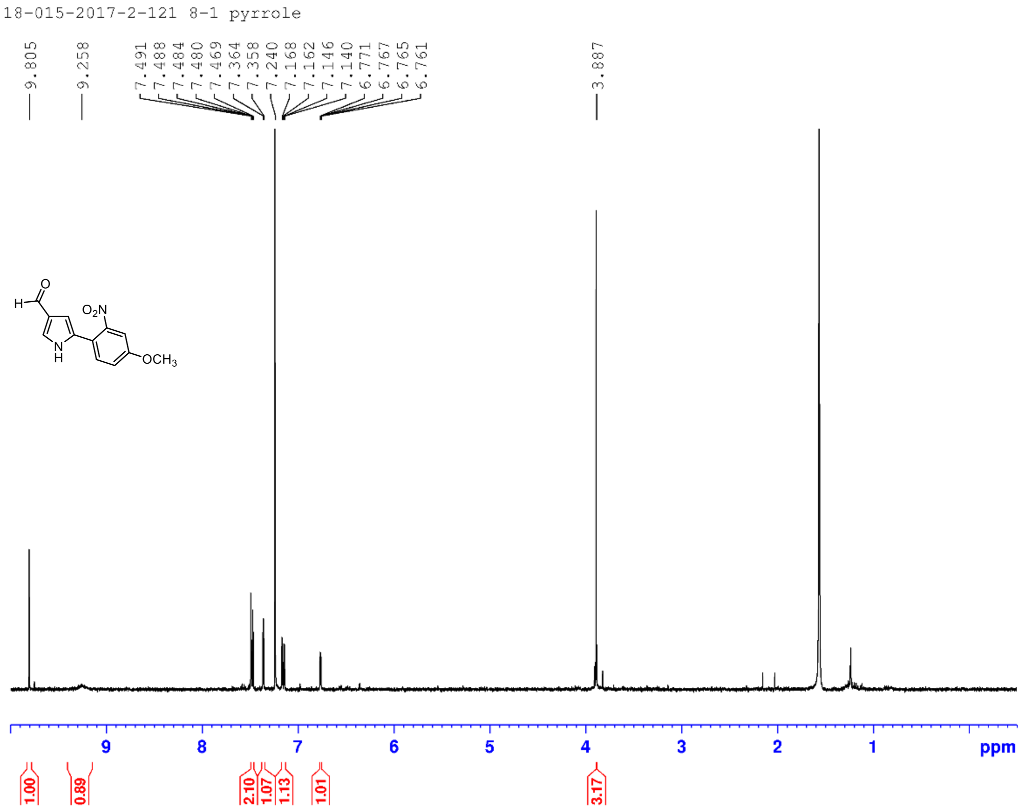

^{1}H NMR spectrum of **4h** ($CDCl_3$, 400 MHz)

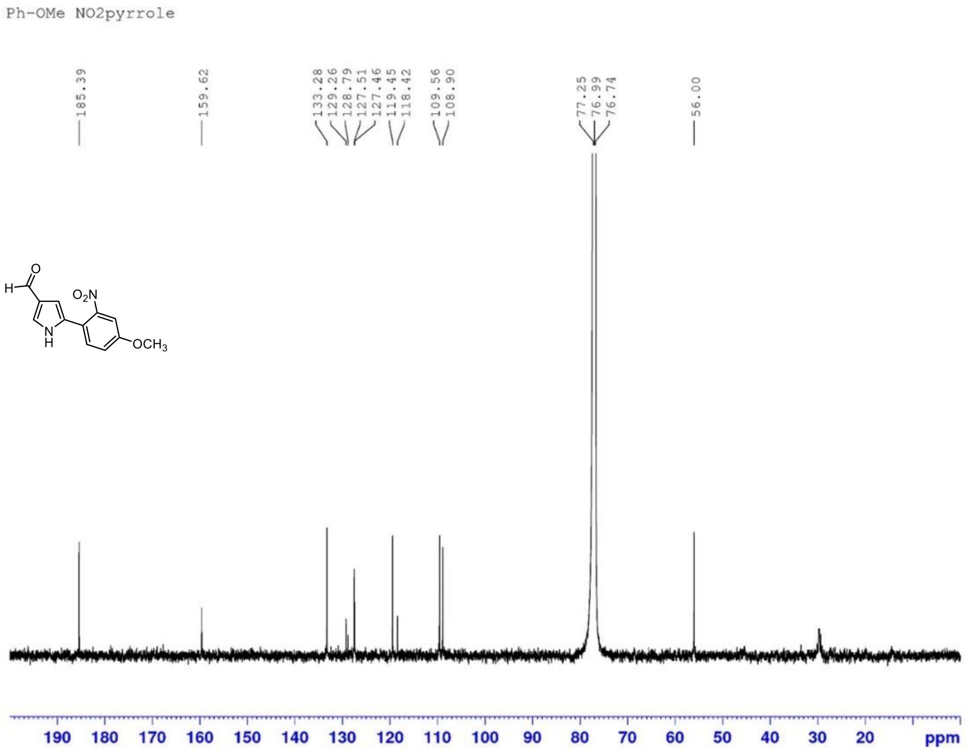

^{13}C{^{1}H} NMR spectrum of **4h** ($CDCl_3$, 125 MHz)

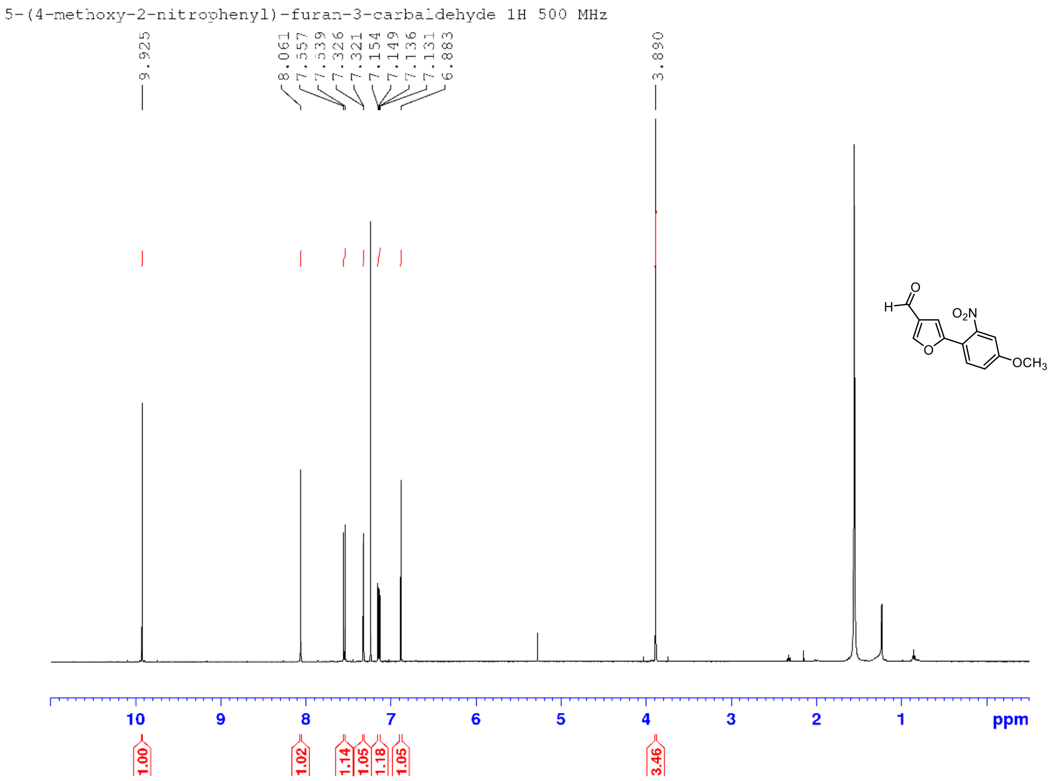

^{1}H NMR spectrum of **5h** ($CDCl_3$, 500 MHz)

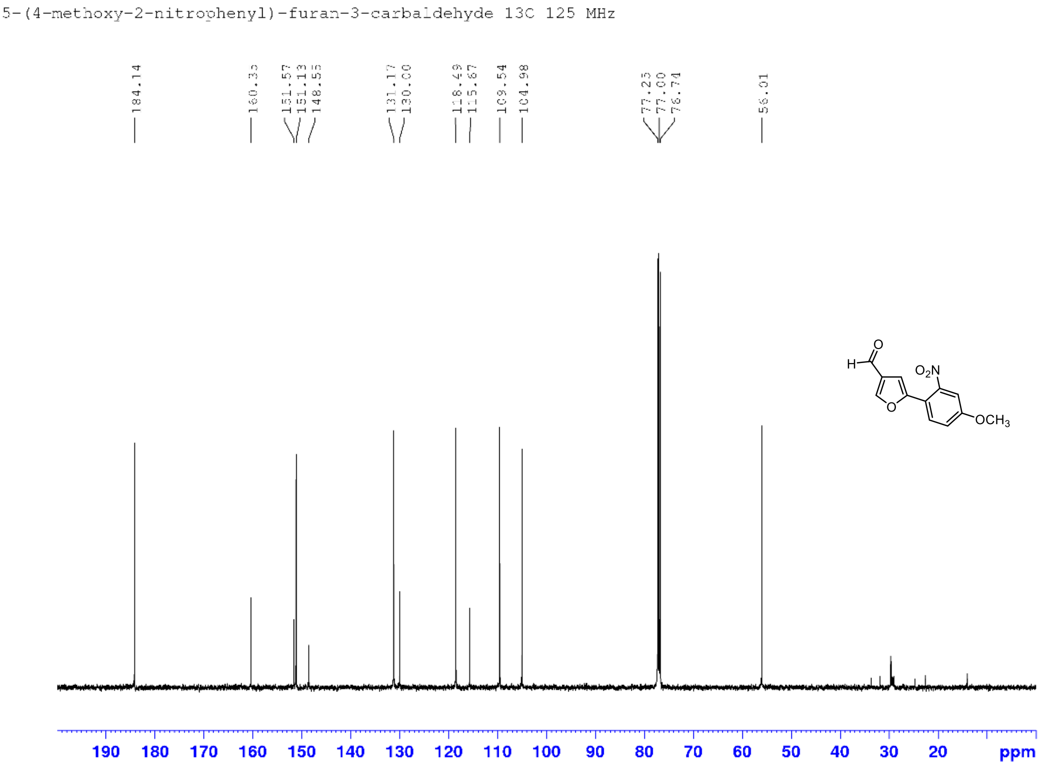

^{13}C{^{1}H} NMR spectrum of **5h** ($CDCl_3$, 125 MHz)

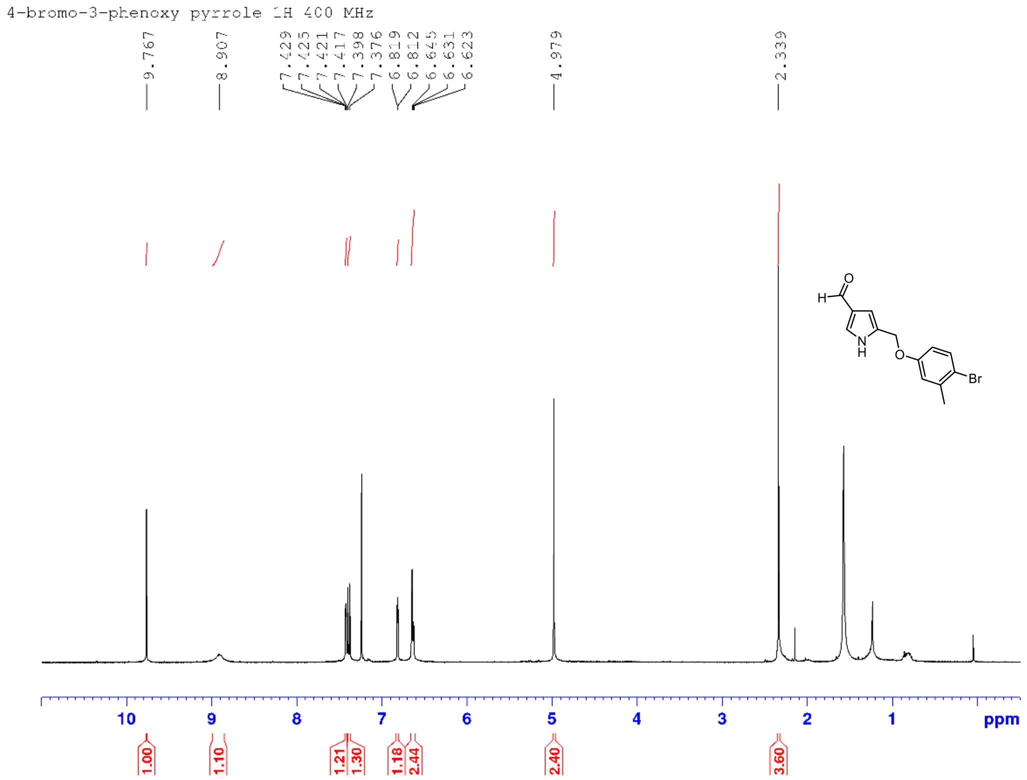

^{1}H NMR spectrum of **4i** ($CDCl_3$, 400 MHz)

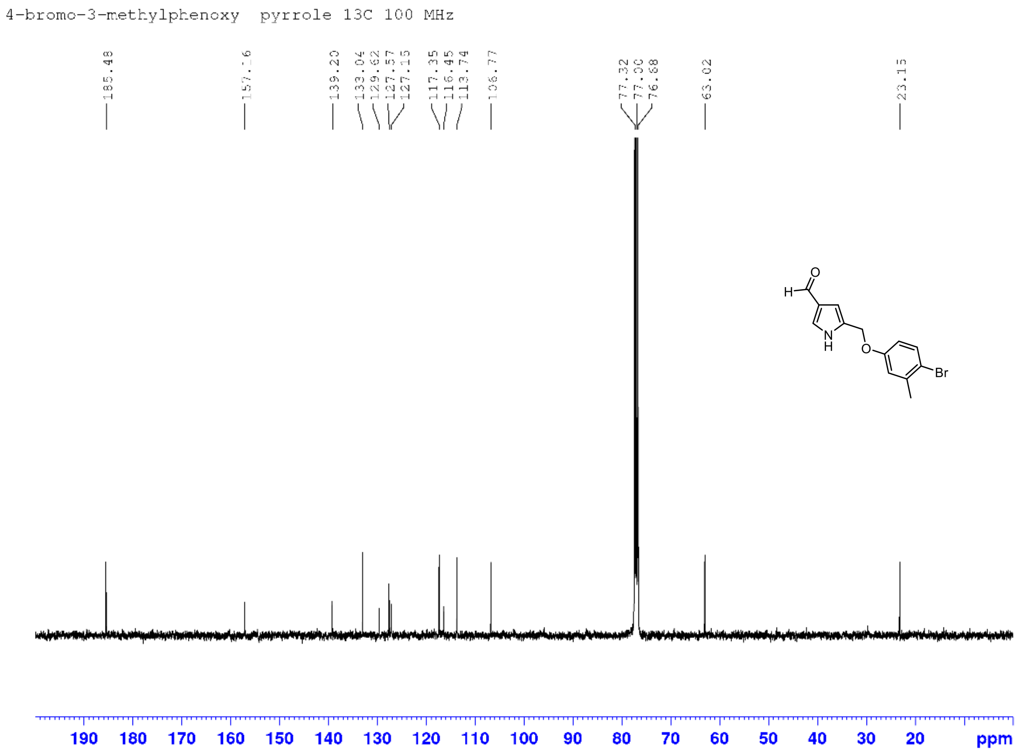

^{13}C{^{1}H} NMR spectrum of **4i** ($CDCl_3$, 100 MHz)

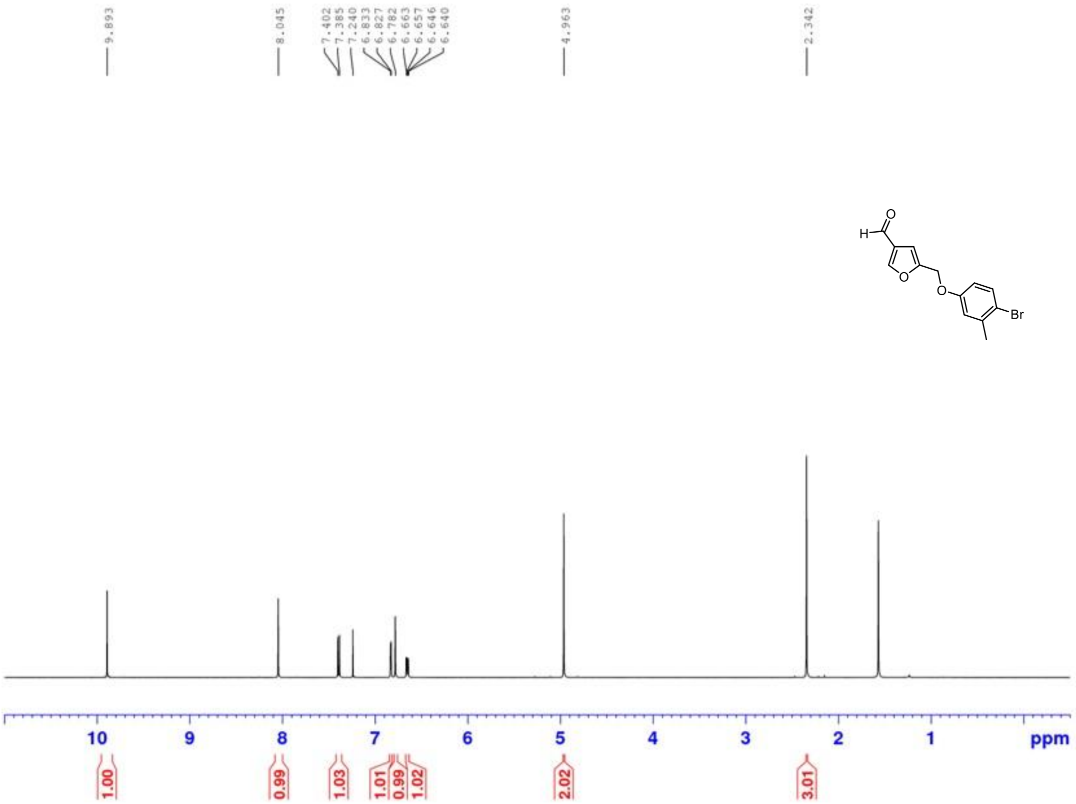

^{1}H NMR spectrum of **5i** ($CDCl_3$, 500 MHz)

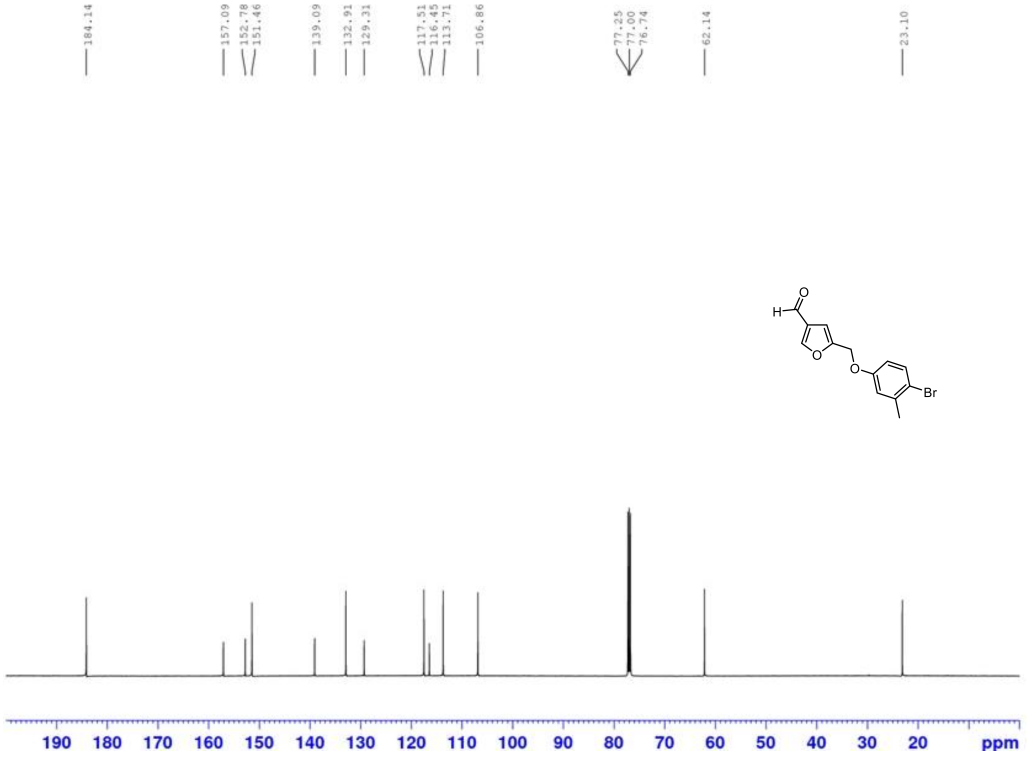

$^{13}C\{^{1}H\}$ NMR spectrum of **5i** ($CDCl_3$, 125 MHz)

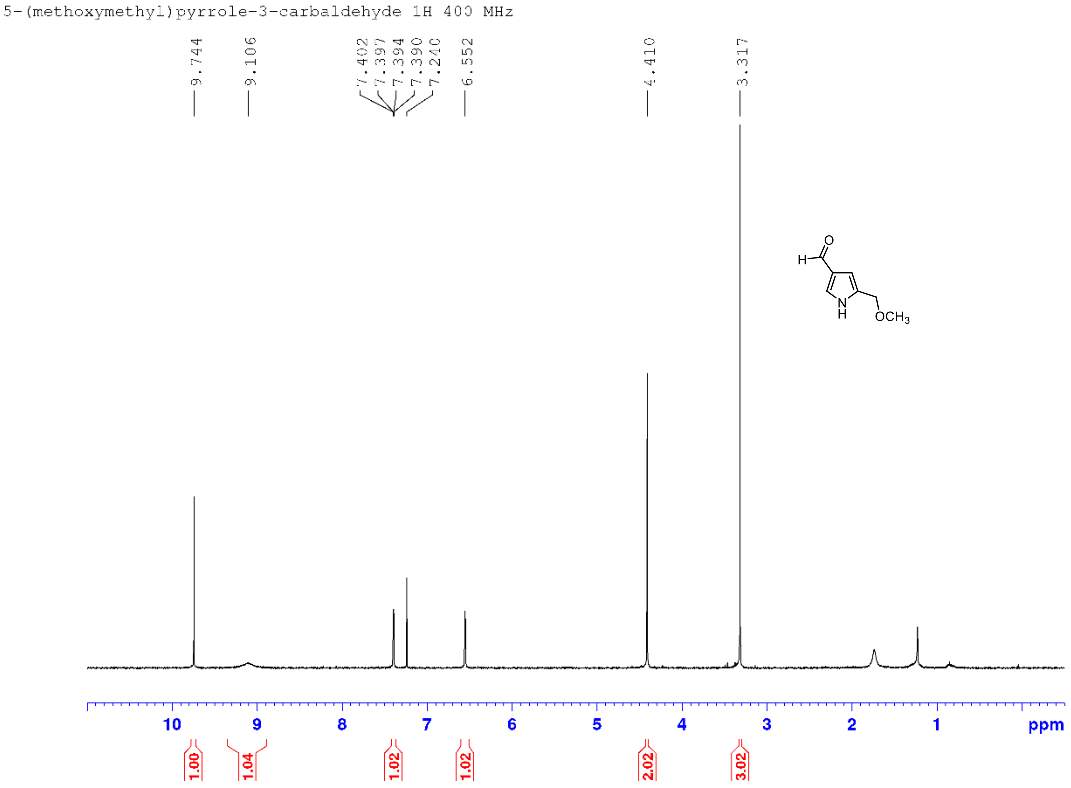

^{1}H NMR spectrum of **4j** ($CDCl_3$, 400 MHz)

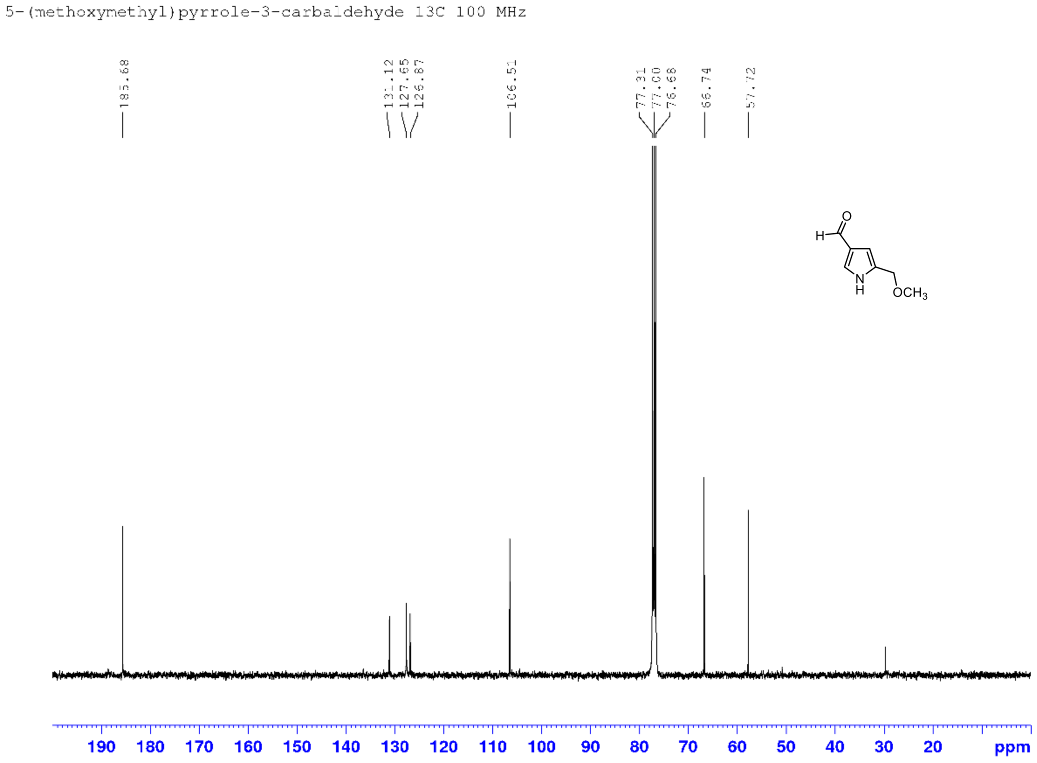

^{13}C{^{1}H} NMR spectrum of **4j** ($CDCl_3$, 100 MHz)

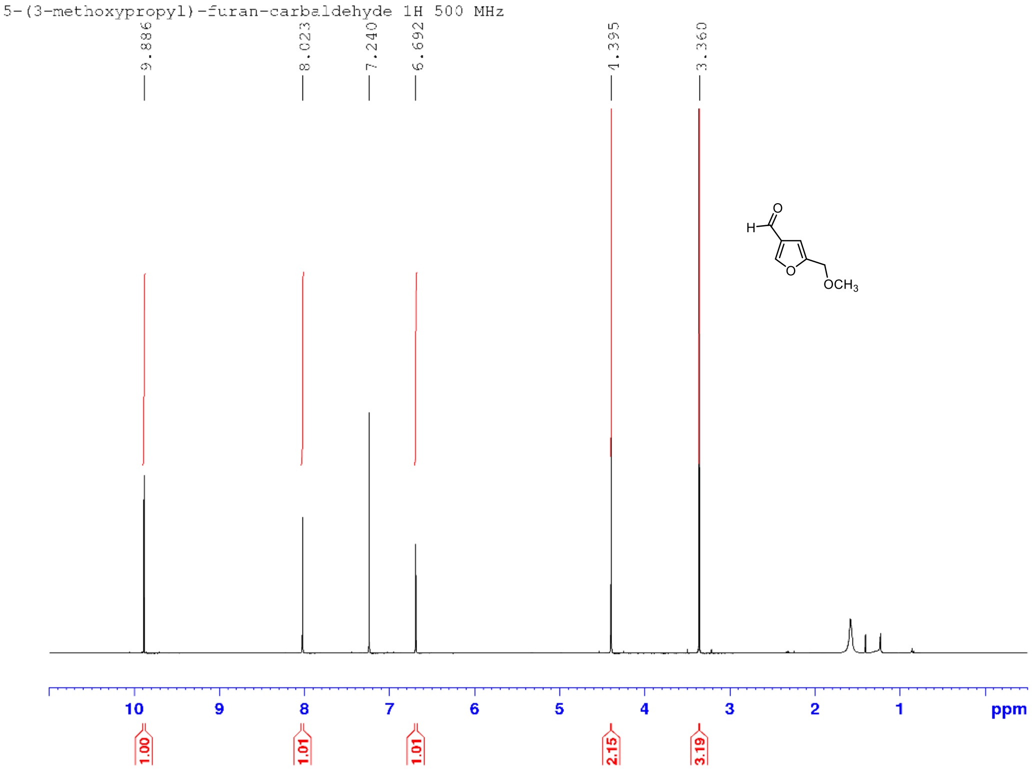

^{1}H NMR spectrum of **5j** ($CDCl_3$, 500 MHz)

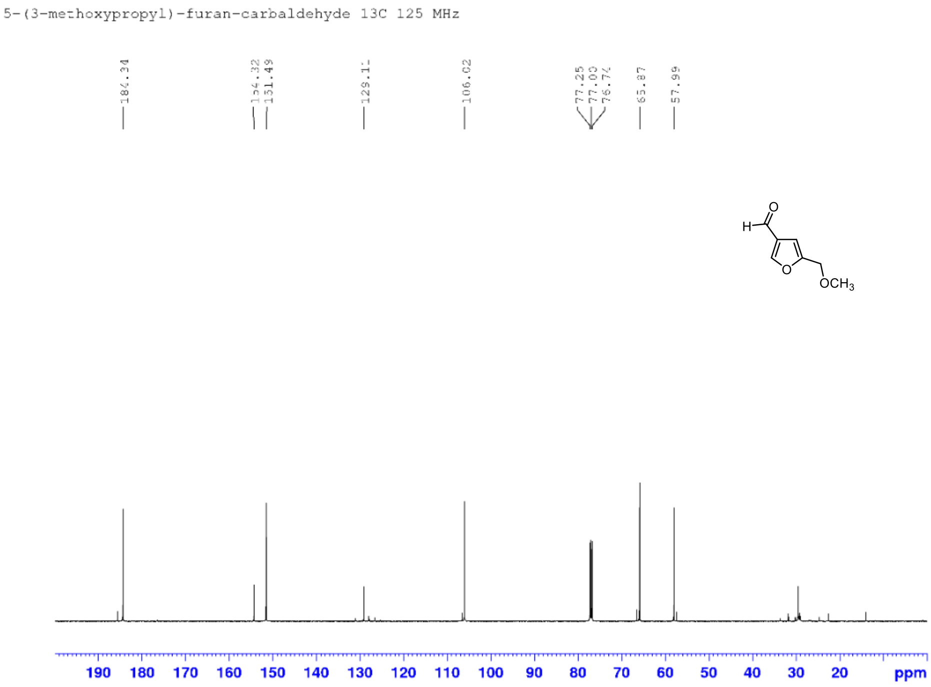

$^{13}C\{^{1}H\}$ NMR spectrum of **5j** ($CDCl_3$, 125 MHz)

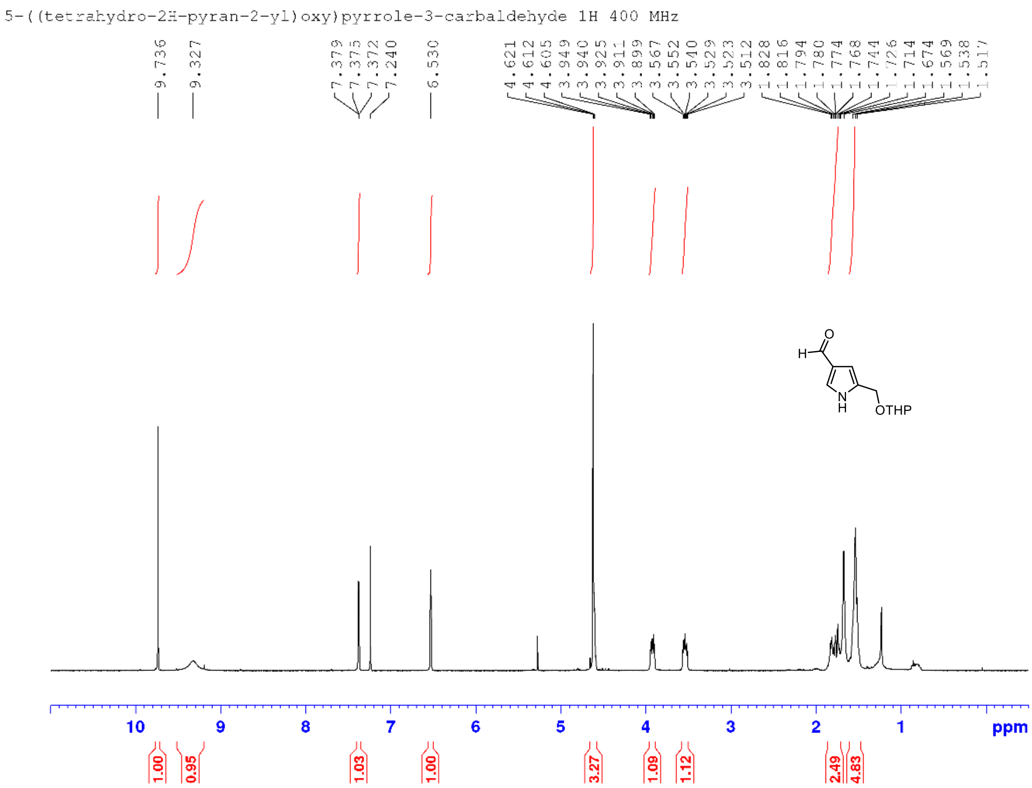

^{1}H NMR spectrum of **4k** ($CDCl_3$, 400 MHz)

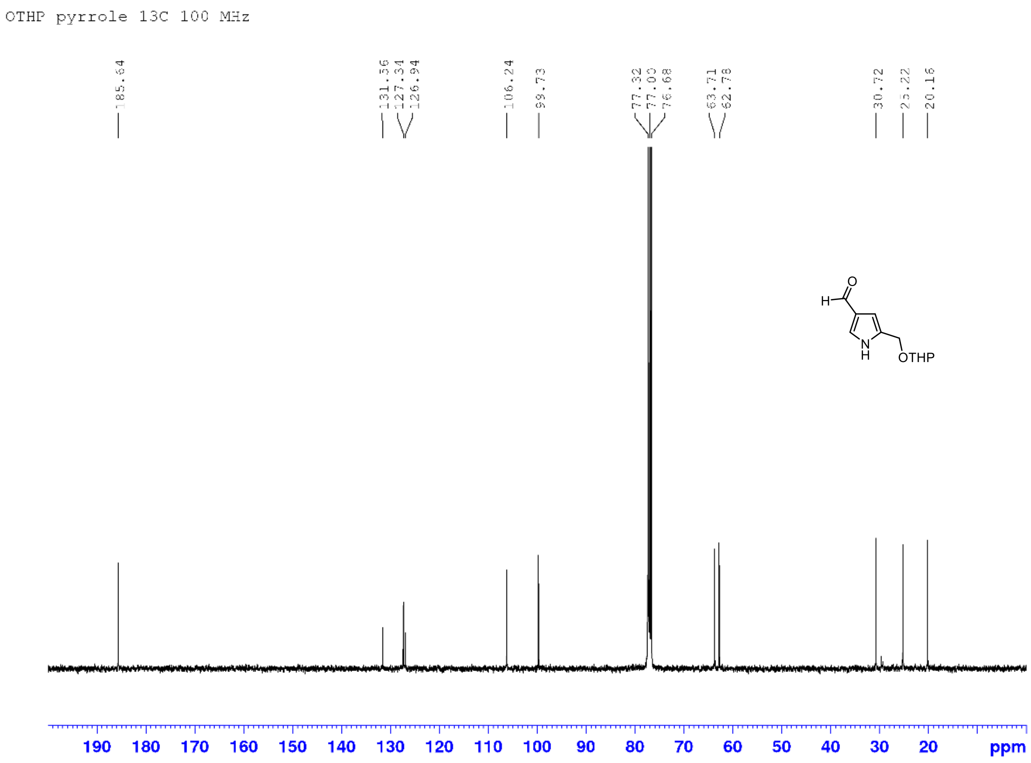

^{13}C{^{1}H} NMR spectrum of **4k** ($CDCl_3$, 100 MHz)

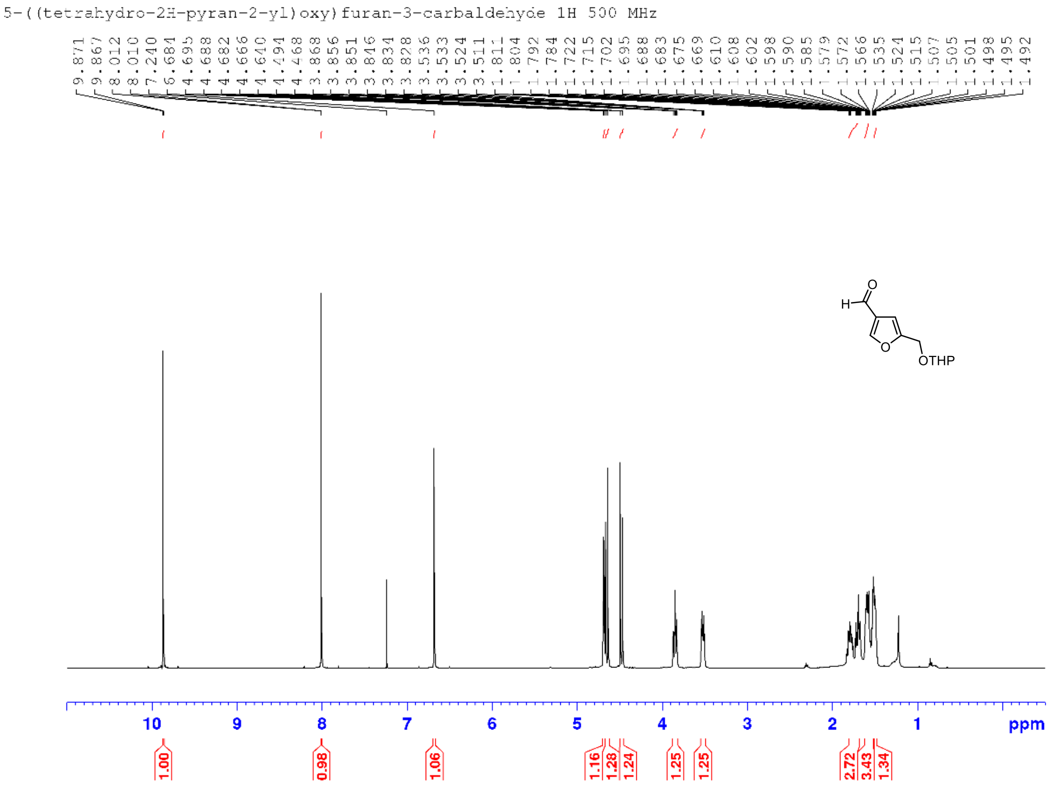

^{1}H NMR spectrum of **5k** ($CDCl_3$, 500 MHz)

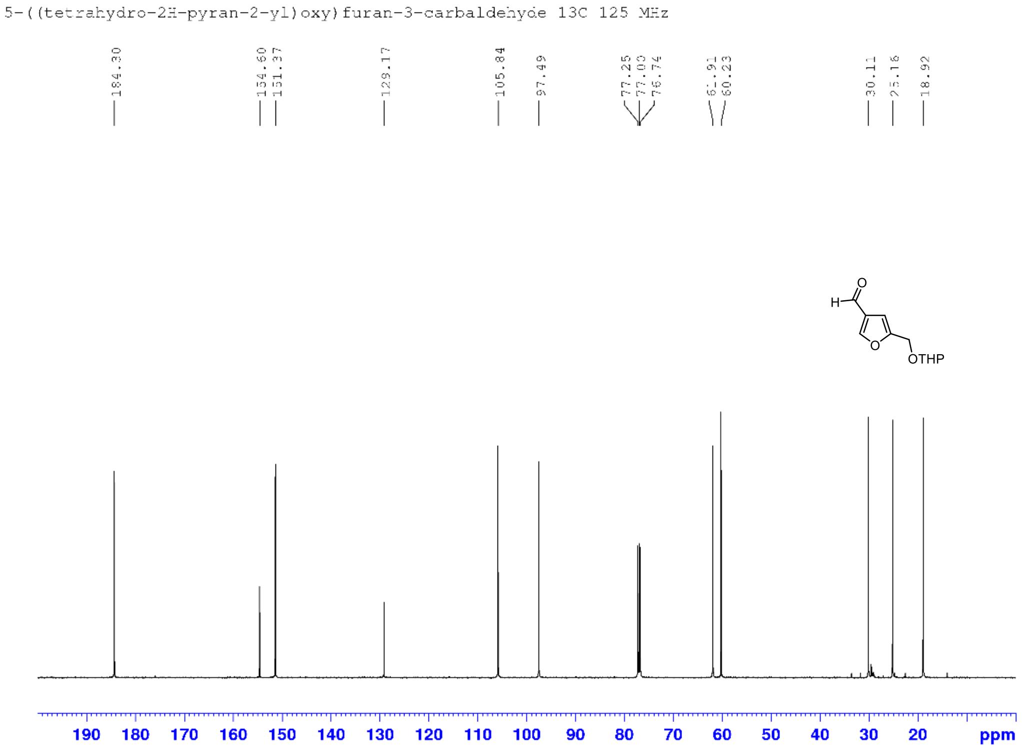

$^{13}C\{^{1}H\}$ NMR spectrum of **5k** ($CDCl_3$, 125 MHz)

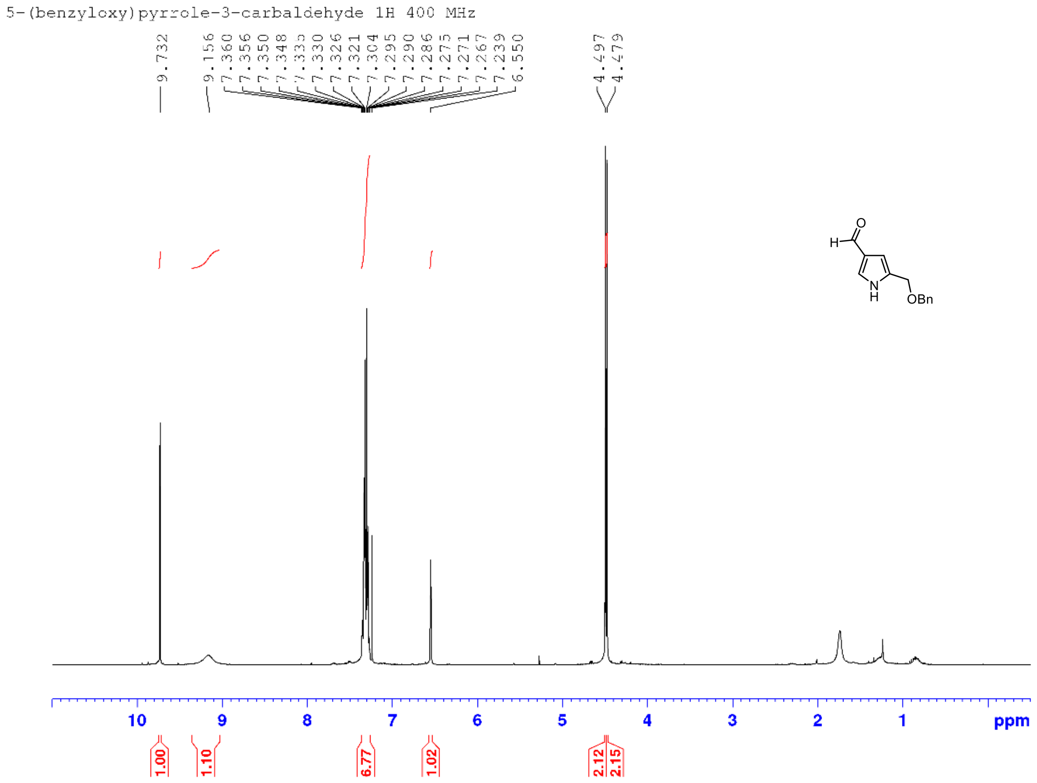

^{1}H NMR spectrum of **4l** ($CDCl_3$, 400 MHz)

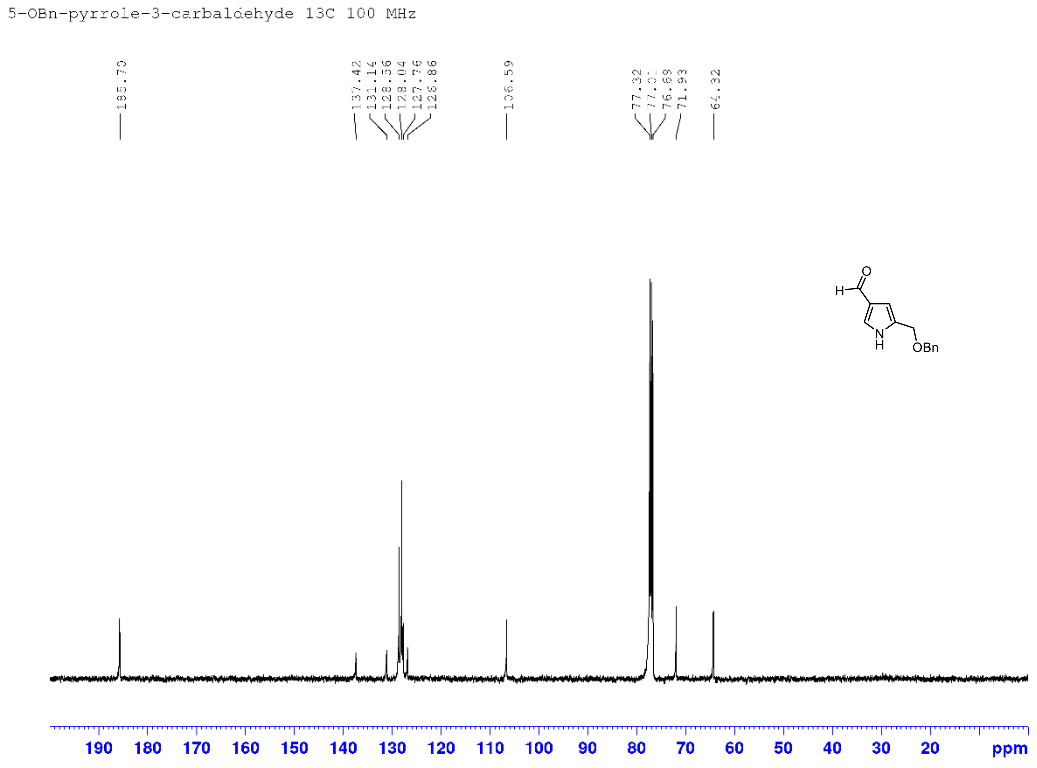

^{13}C{^{1}H} NMR spectrum of **4l** ($CDCl_3$, 100 MHz)

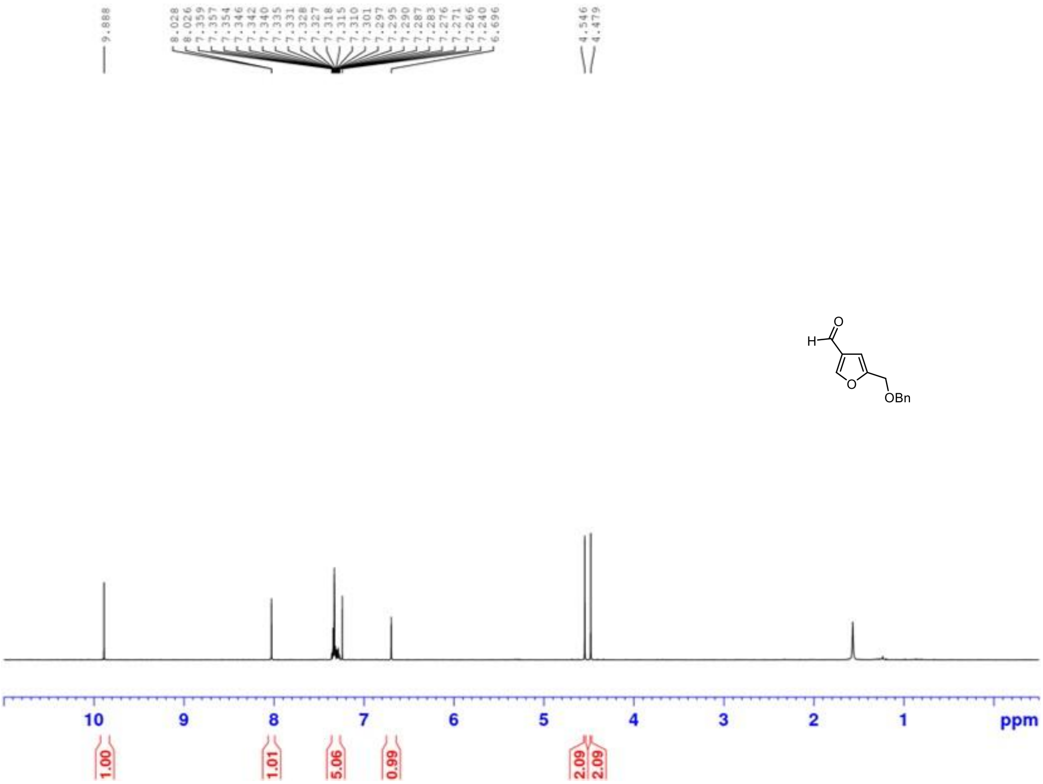

^{1}H NMR spectrum of **5l** ($CDCl_3$, 500 MHz)

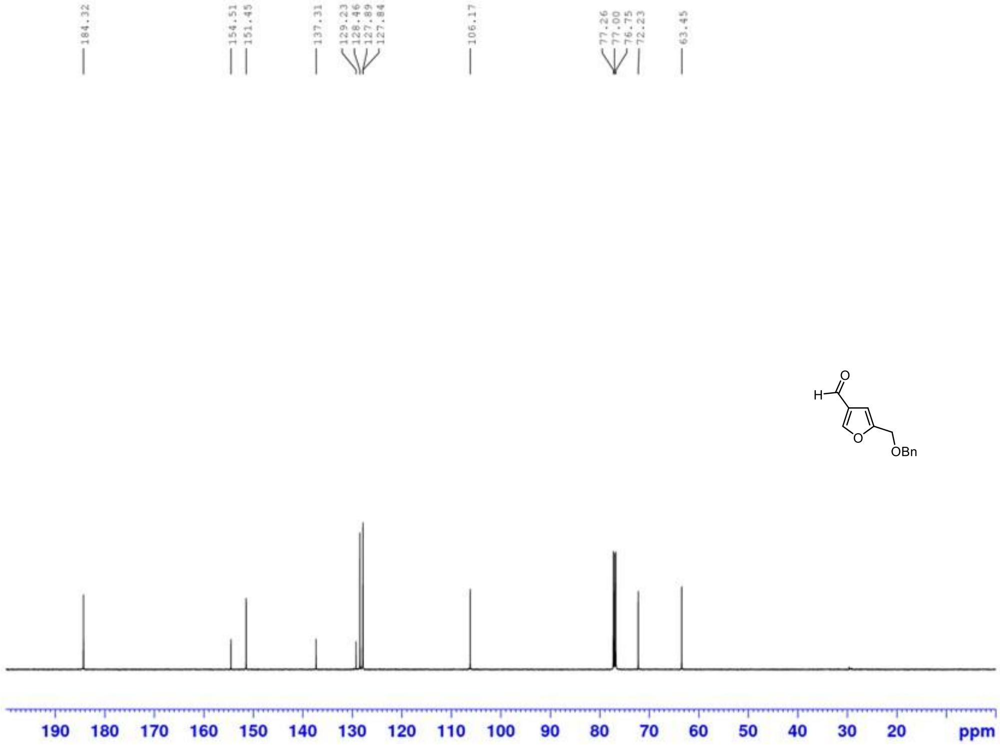

^{13}C{^{1}H} NMR spectrum of **5l** ($CDCl_3$, 125 MHz)

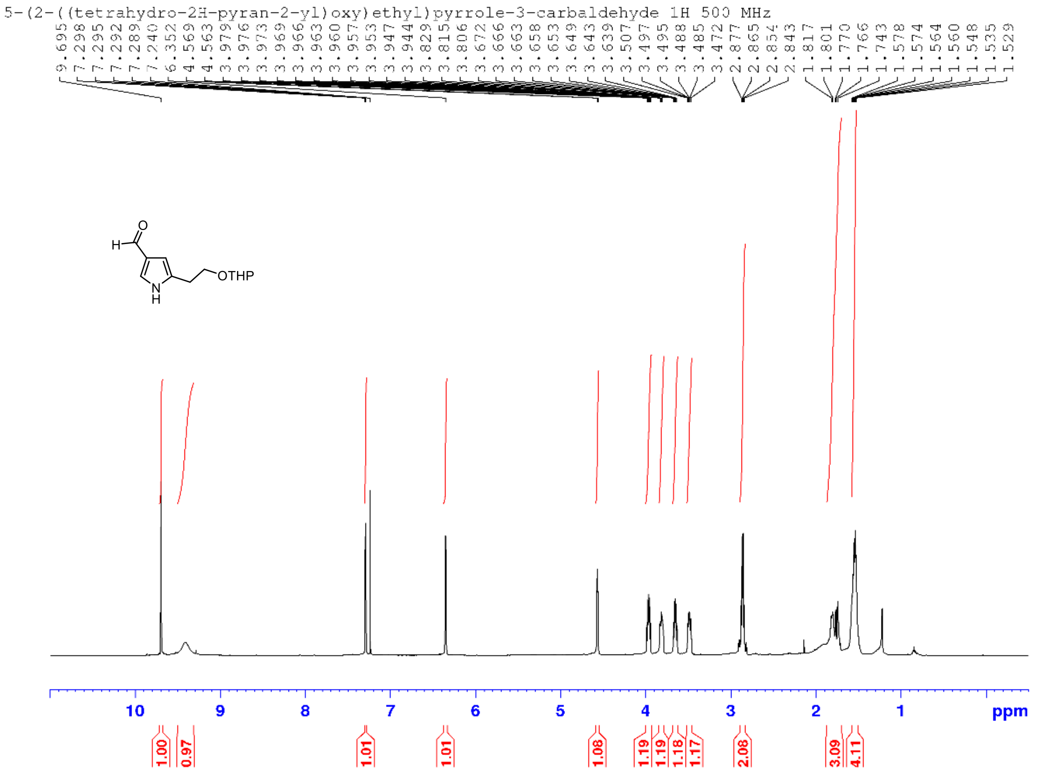

^{1}H NMR spectrum of **4m** (CDCl$_3$, 500 MHz)

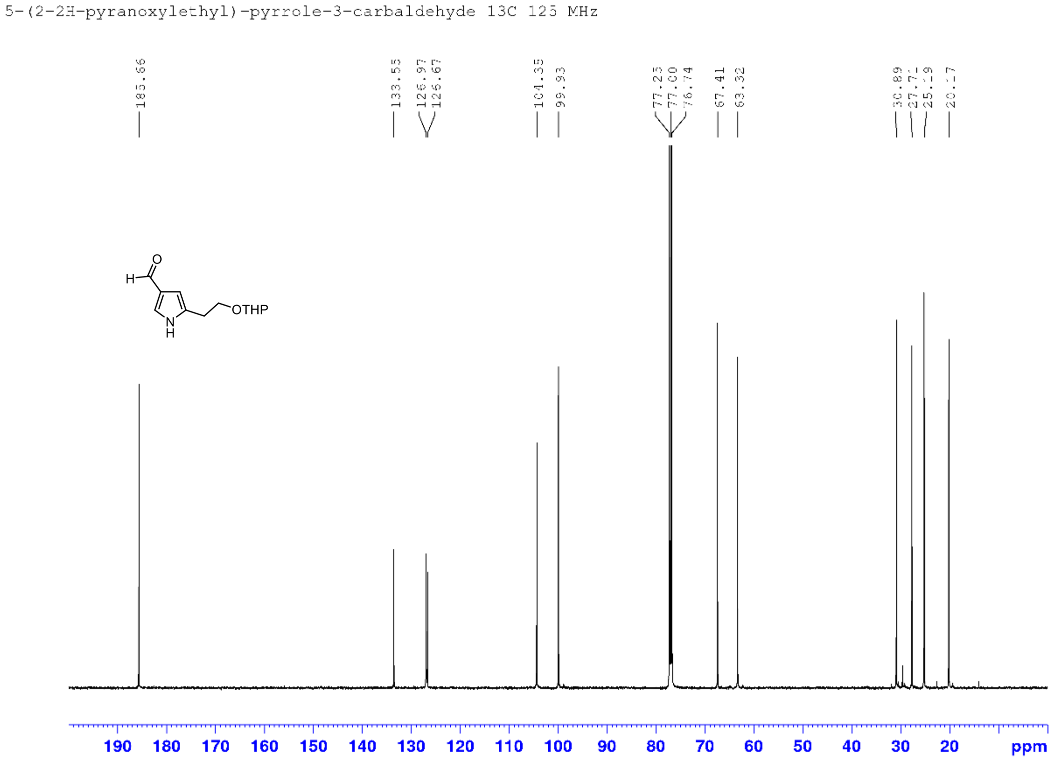

^{13}C{^{1}H} NMR spectrum of **4m** (CDCl$_3$, 125 MHz)

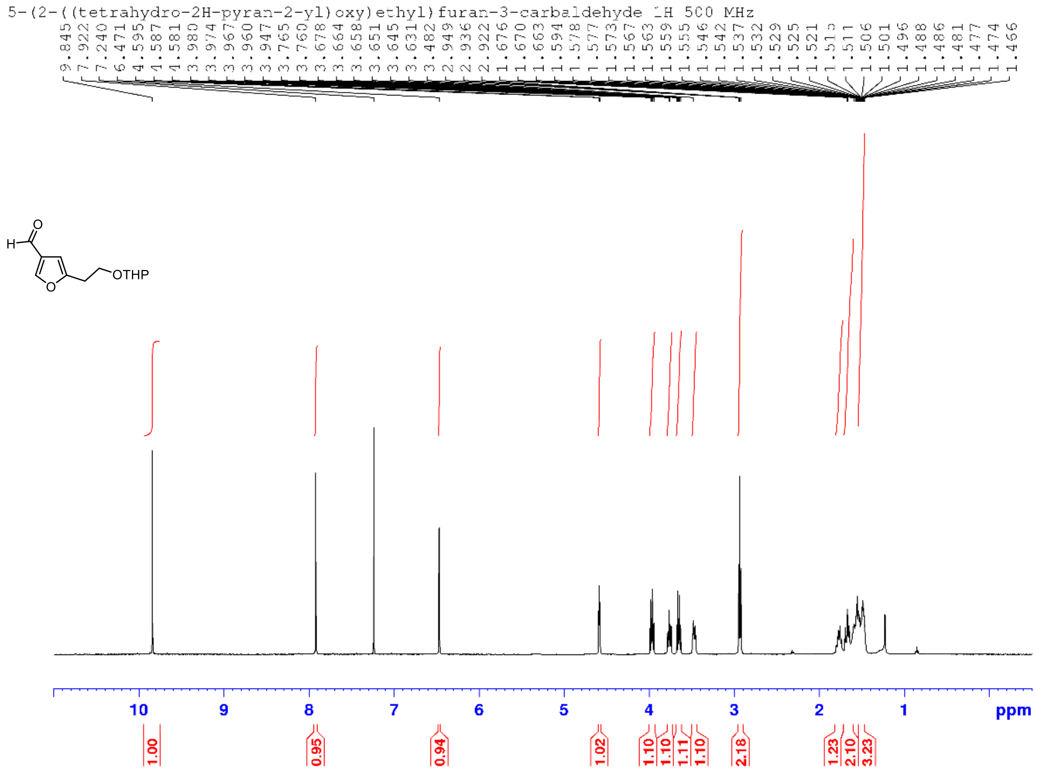

^{1}H NMR spectrum of **5m** ($CDCl_3$, 500 MHz)

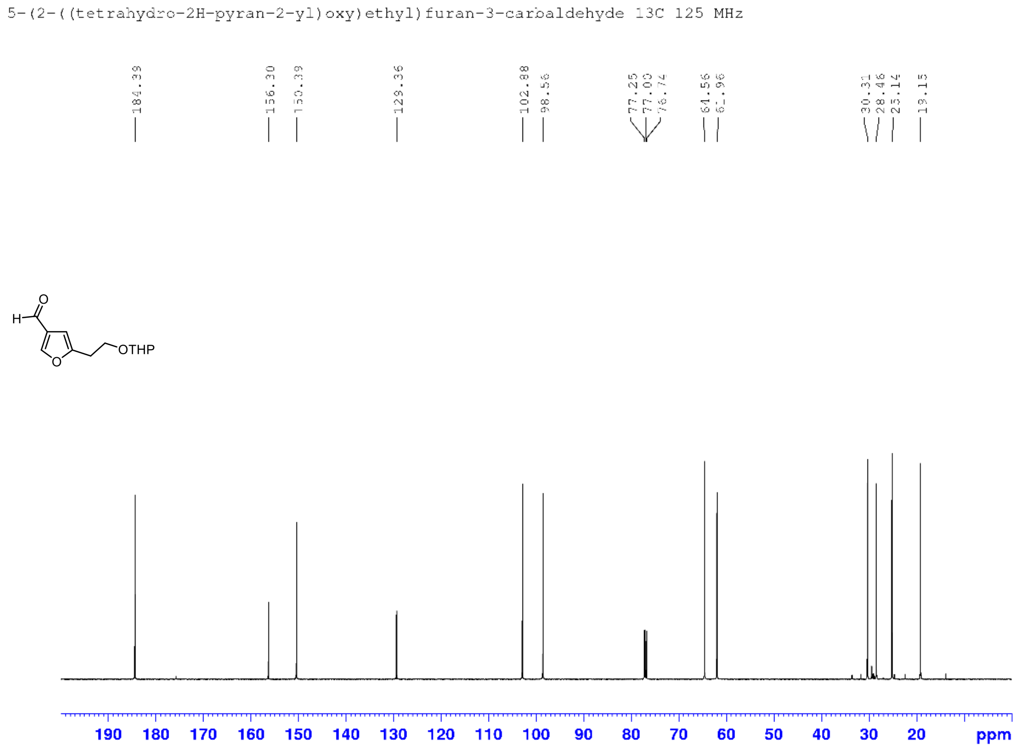

^{13}C{^{1}H} NMR spectrum of **5m** ($CDCl_3$, 125 MHz)

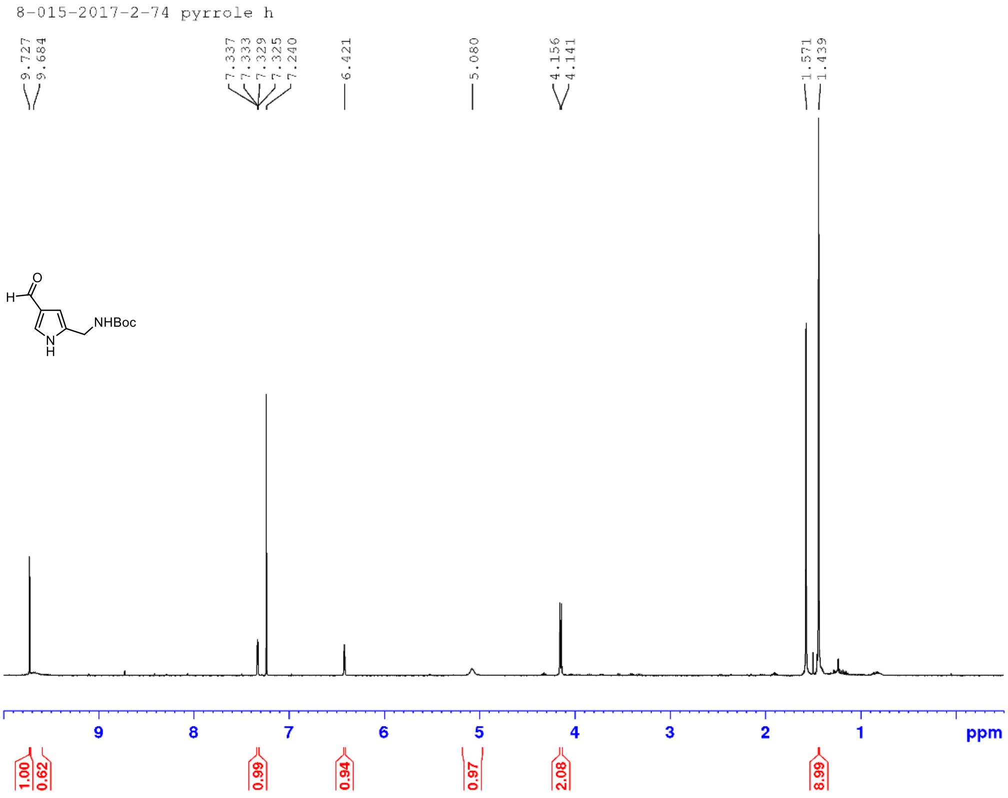

^{1}H NMR spectrum of **4n** ($CDCl_3$, 400 MHz)

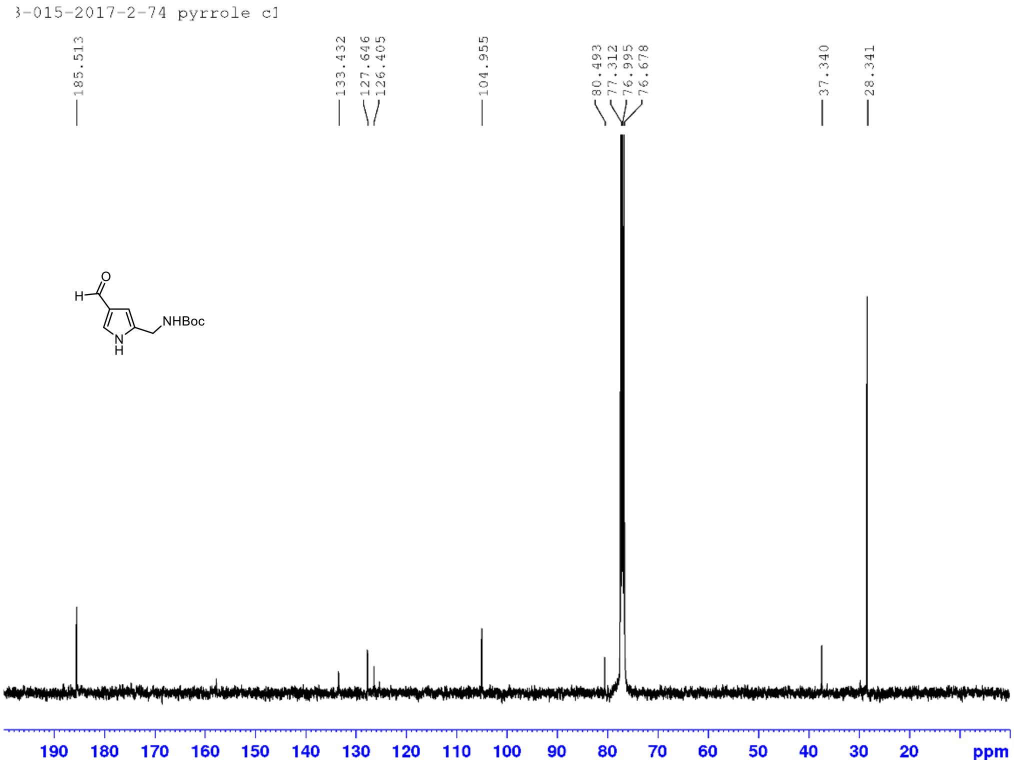

^{13}C{^{1}H} NMR spectrum of **4n** ($CDCl_3$, 100 MHz)

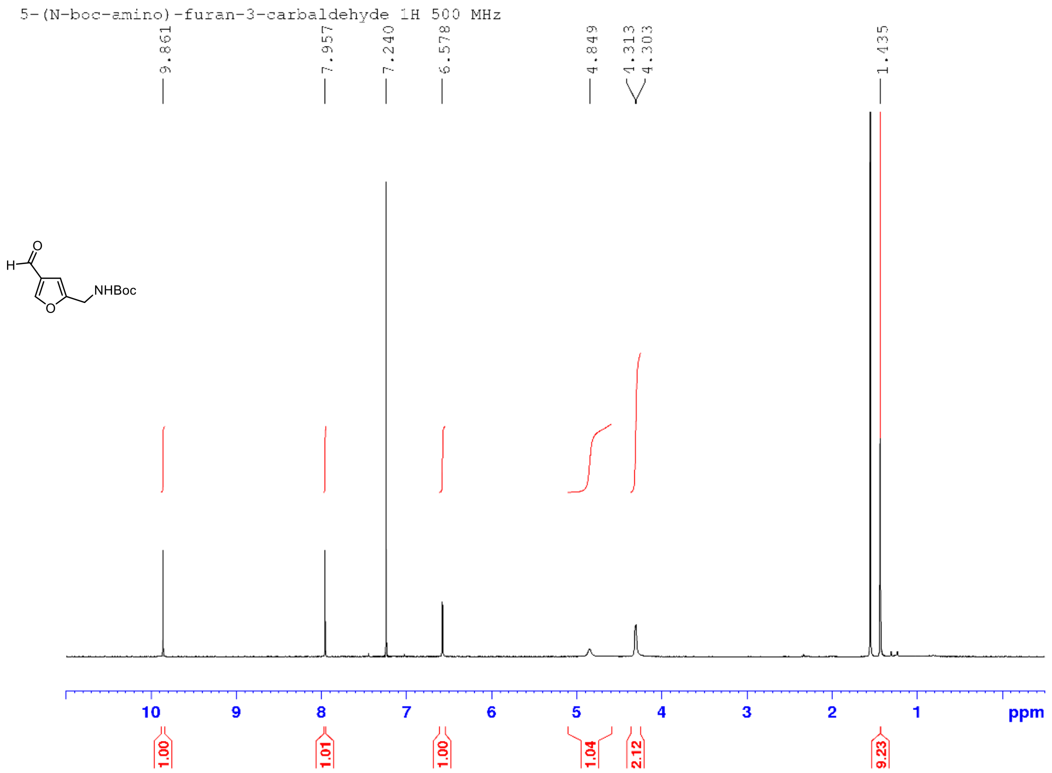

^{1}H NMR spectrum of **5n** ($CDCl_3$, 500 MHz)

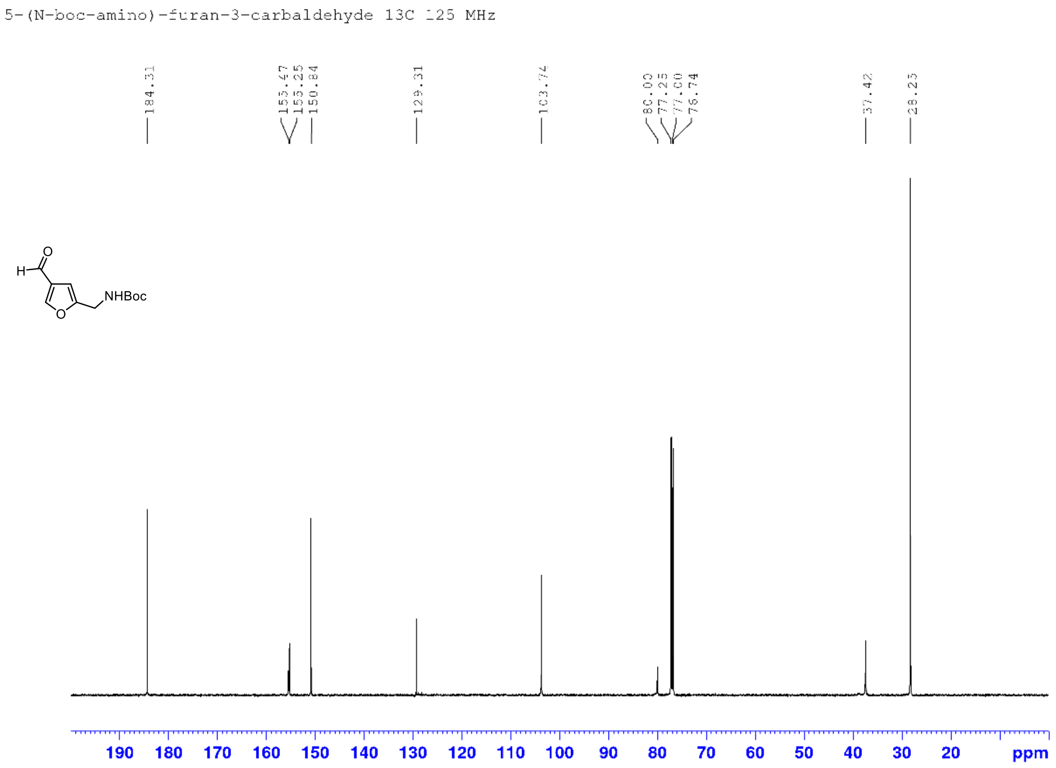

$^{13}C\{^{1}H\}$ NMR spectrum of **5n** ($CDCl_3$, 125 MHz)

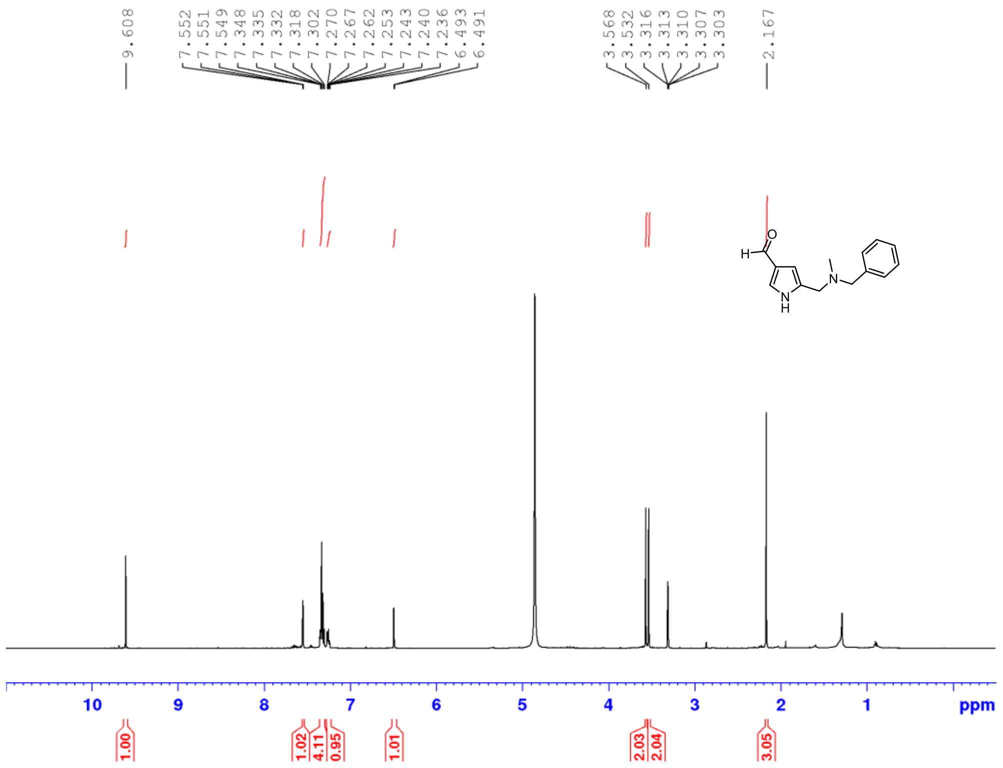

^{1}H NMR spectrum of **4o** (CD_3OD, 500 MHz)

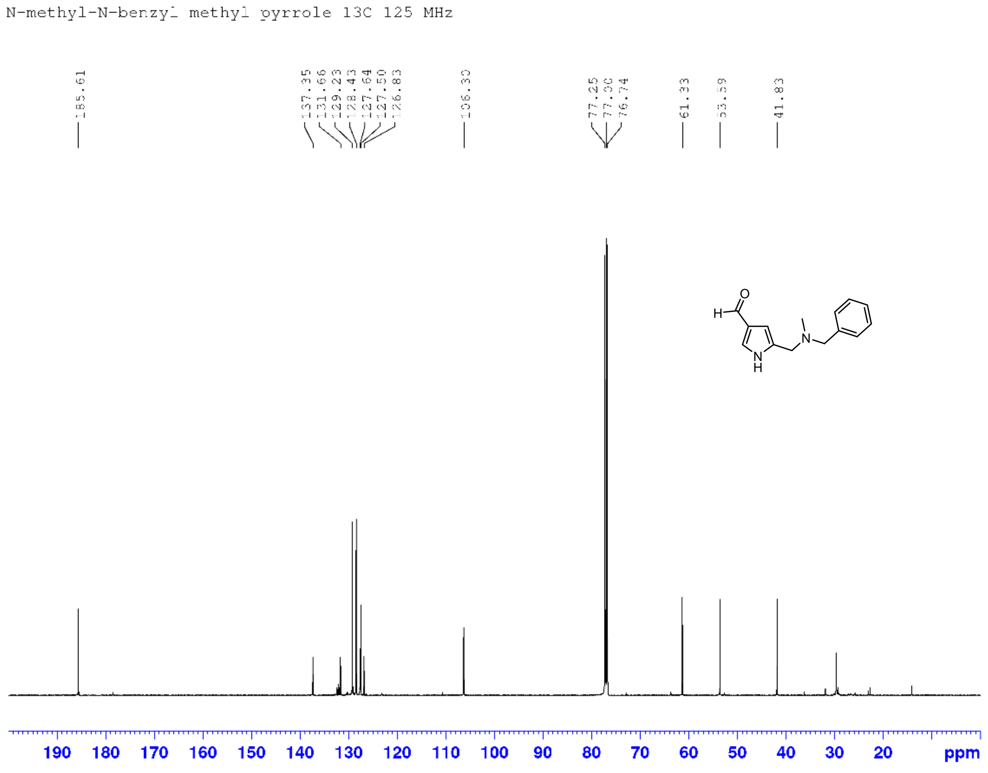

^{13}C{^{1}H} NMR spectrum of **4o** ($CDCl_3$, 125 MHz)

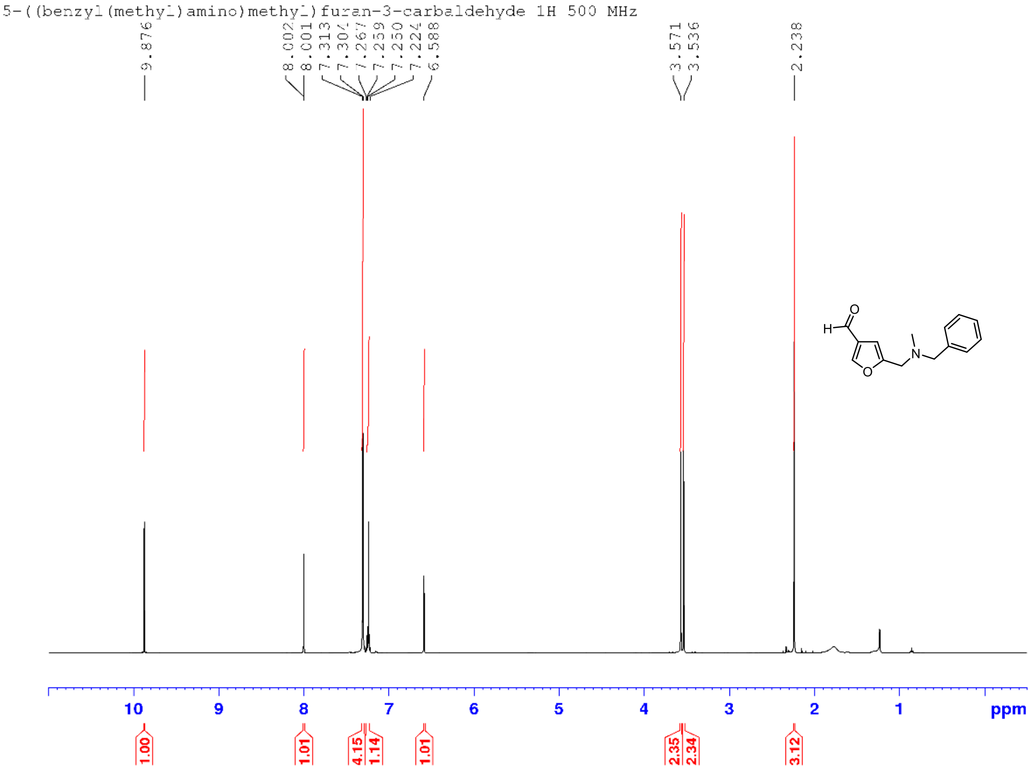

^{1}H NMR spectrum of **5o** ($CDCl_3$, 500 MHz)

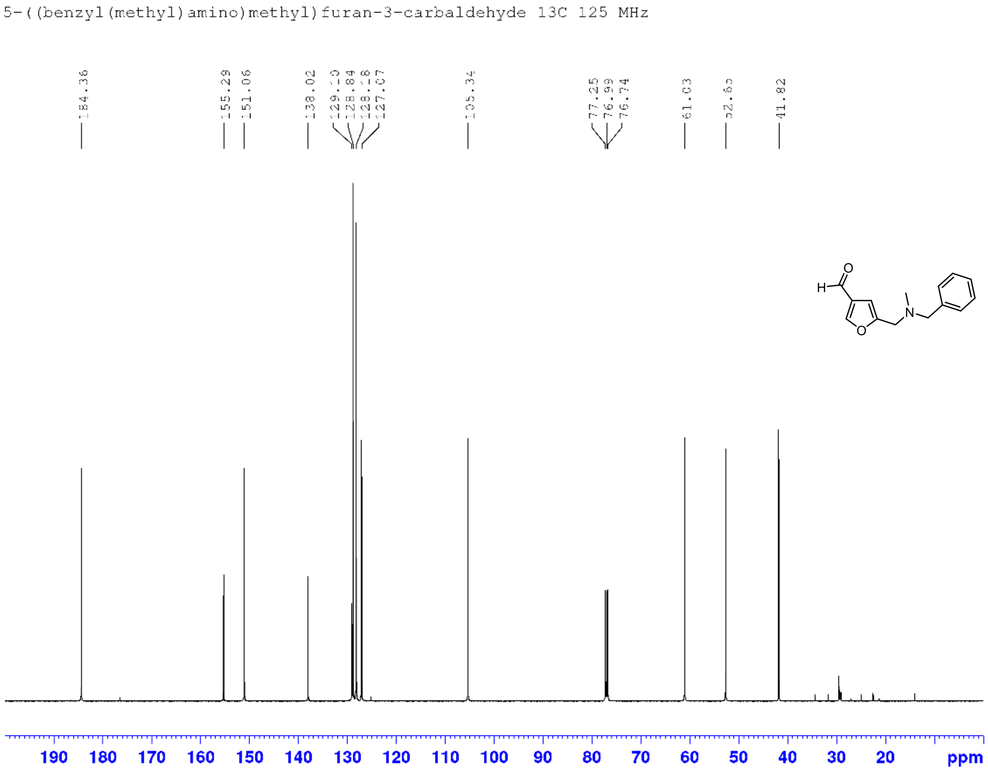

$^{13}C\{^{1}H\}$ NMR spectrum of **5o** ($CDCl_3$, 125 MHz)

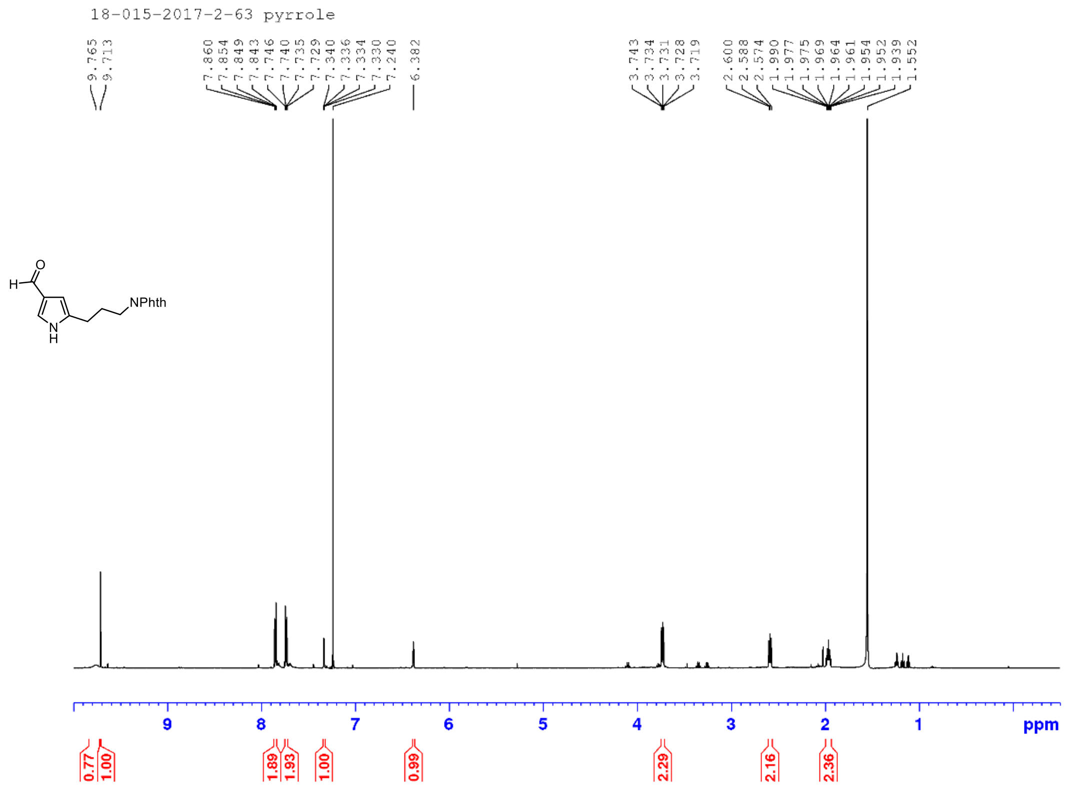

^{1}H NMR spectrum of **4p** ($CDCl_3$, 500 MHz)

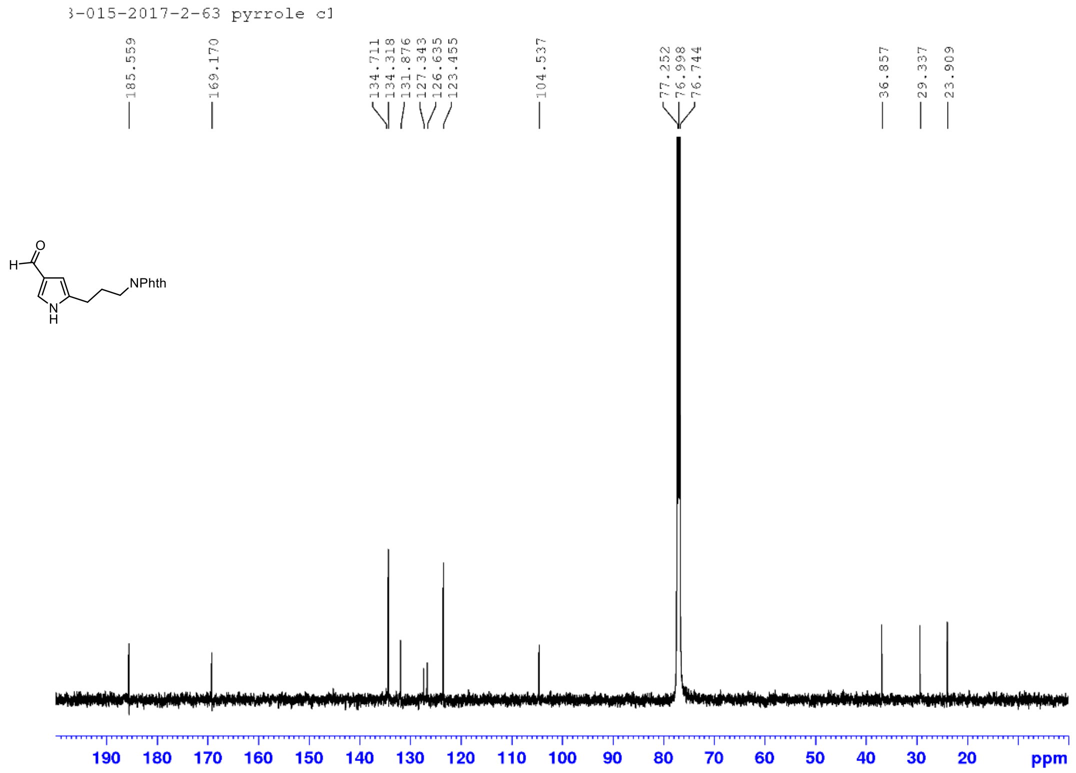

^{13}C{^{1}H} NMR spectrum of **4p** ($CDCl_3$, 125 MHz)

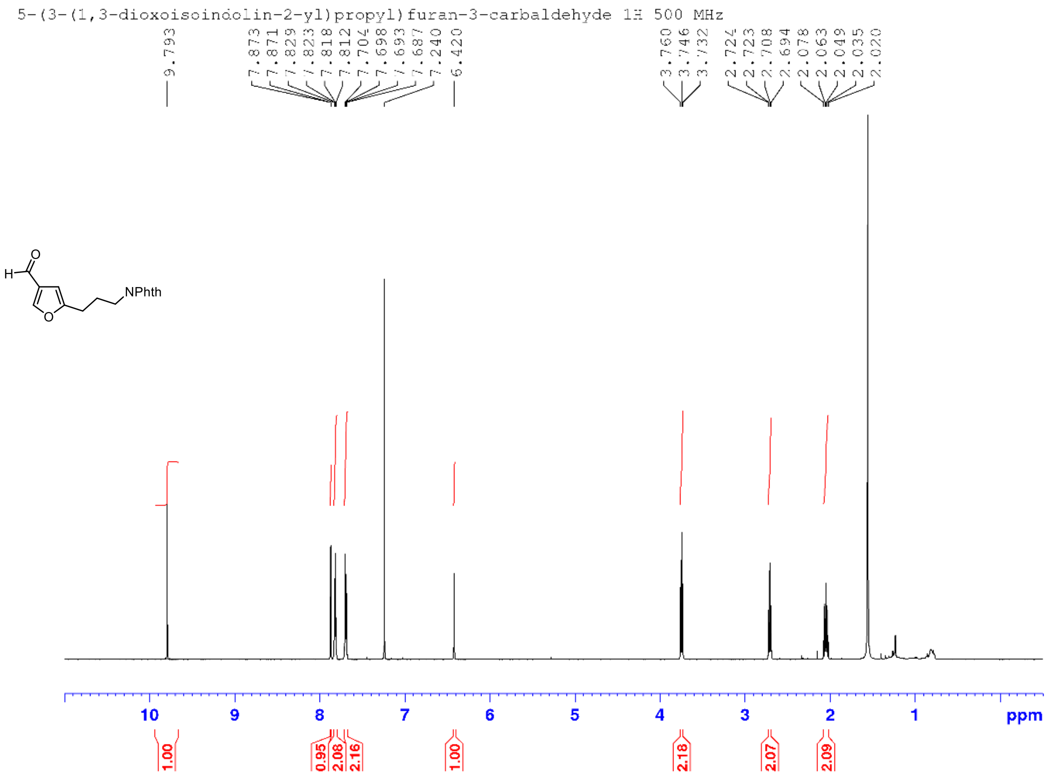

^{1}H NMR spectrum of **5p** ($CDCl_3$, 500 MHz)

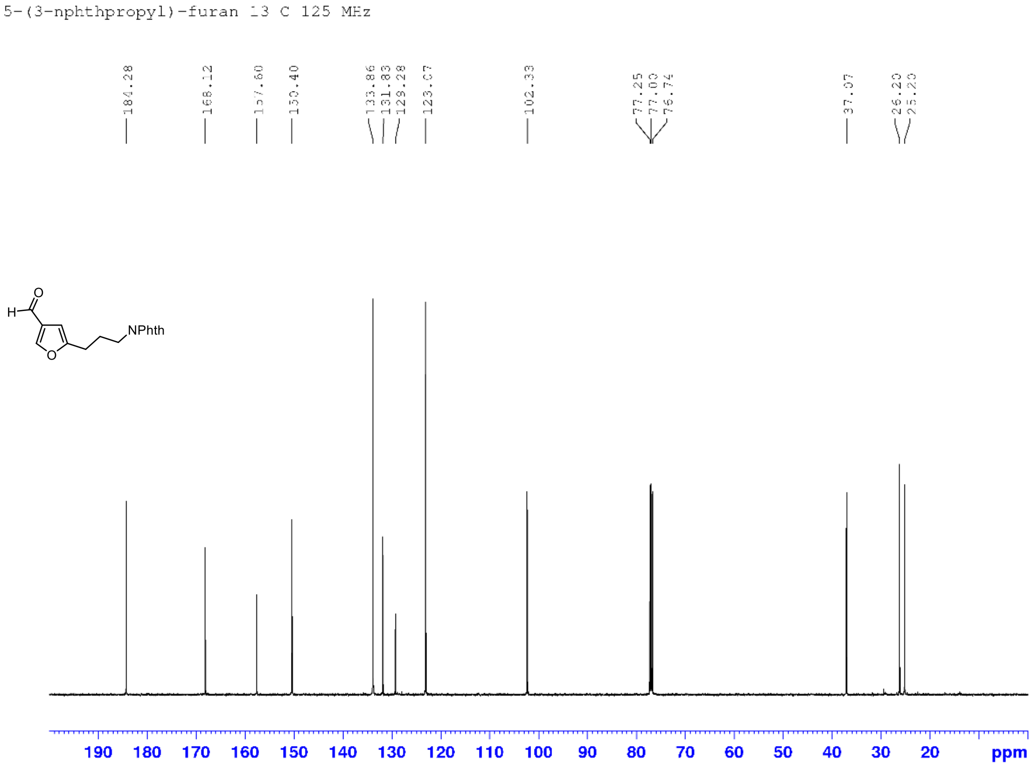

^{13}C{^{1}H} NMR spectrum of **5p** ($CDCl_3$, 125 MHz)

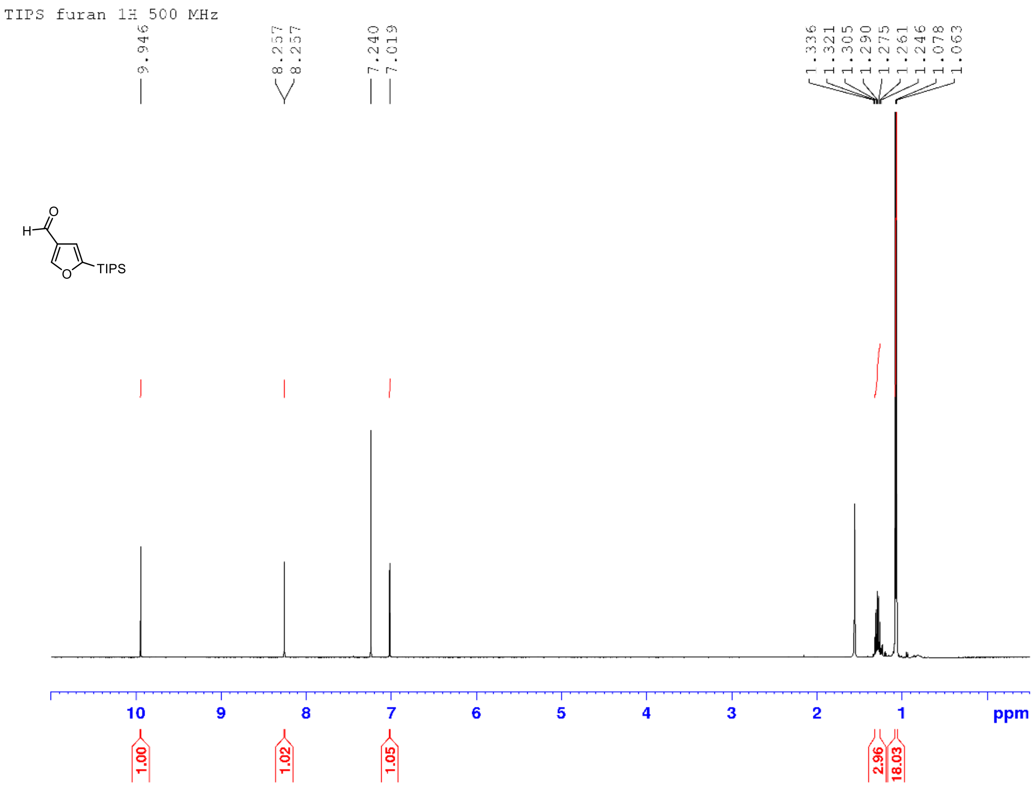

^{1}H NMR spectrum of **5q** ($CDCl_3$, 500 MHz)

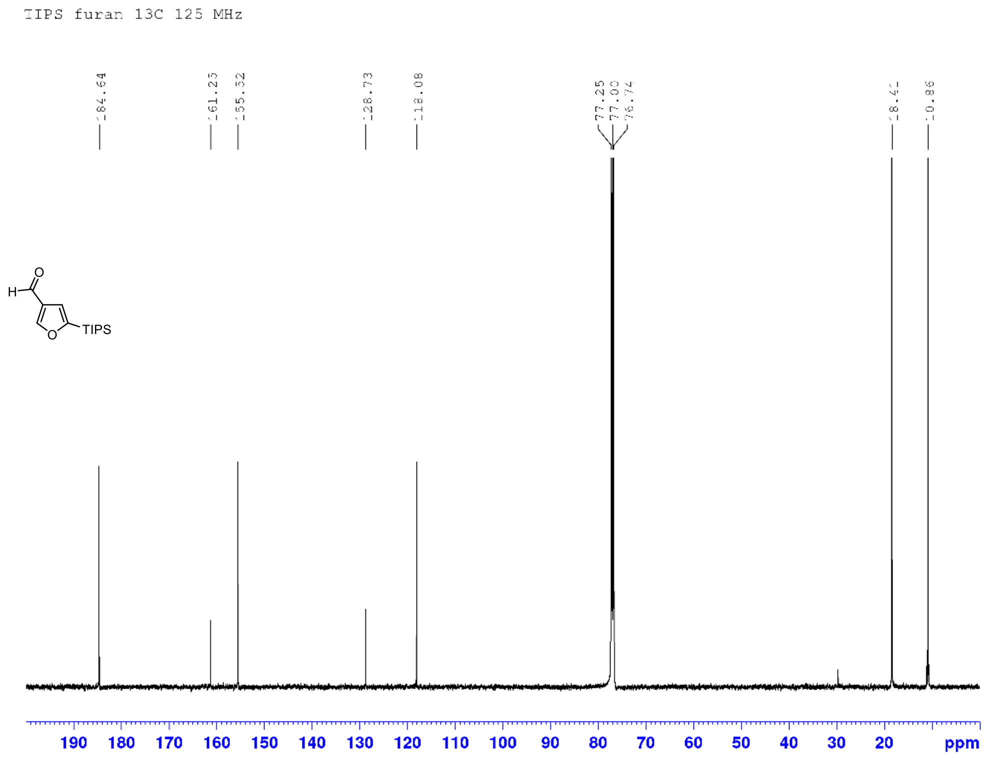

$^{13}C\{^{1}H\}$ NMR spectrum of **5q** ($CDCl_3$, 125 MHz)

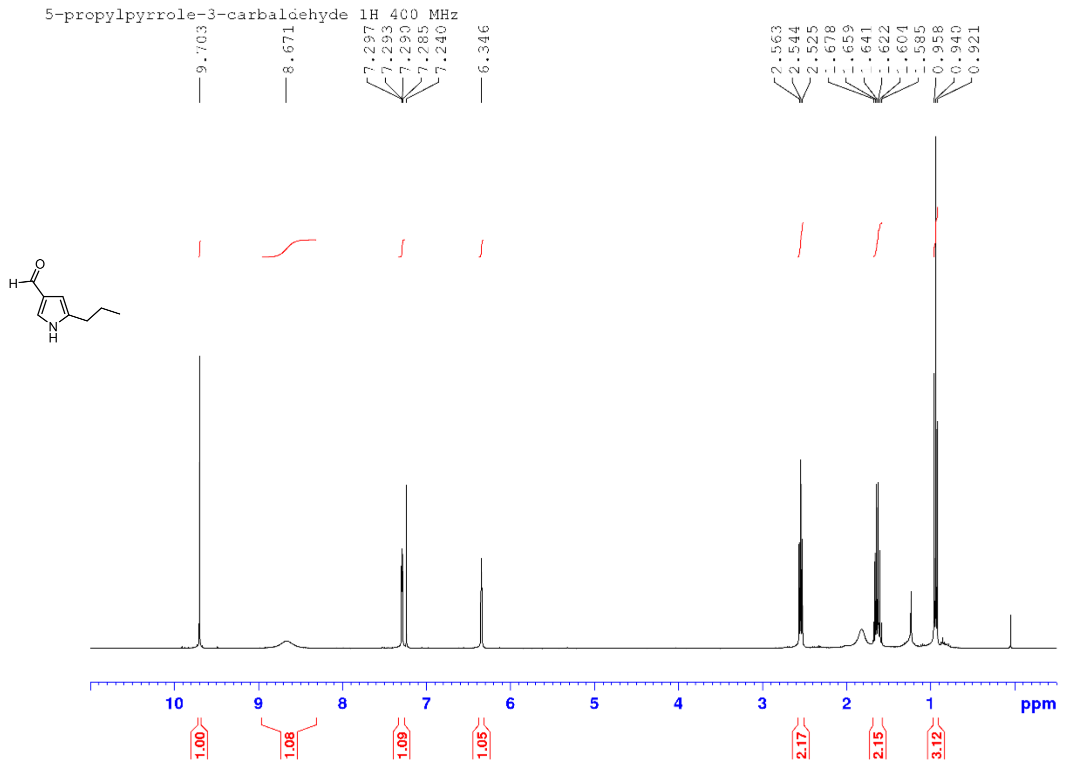

^1H NMR spectrum of **4r** ($CDCl_3$, 400 MHz)

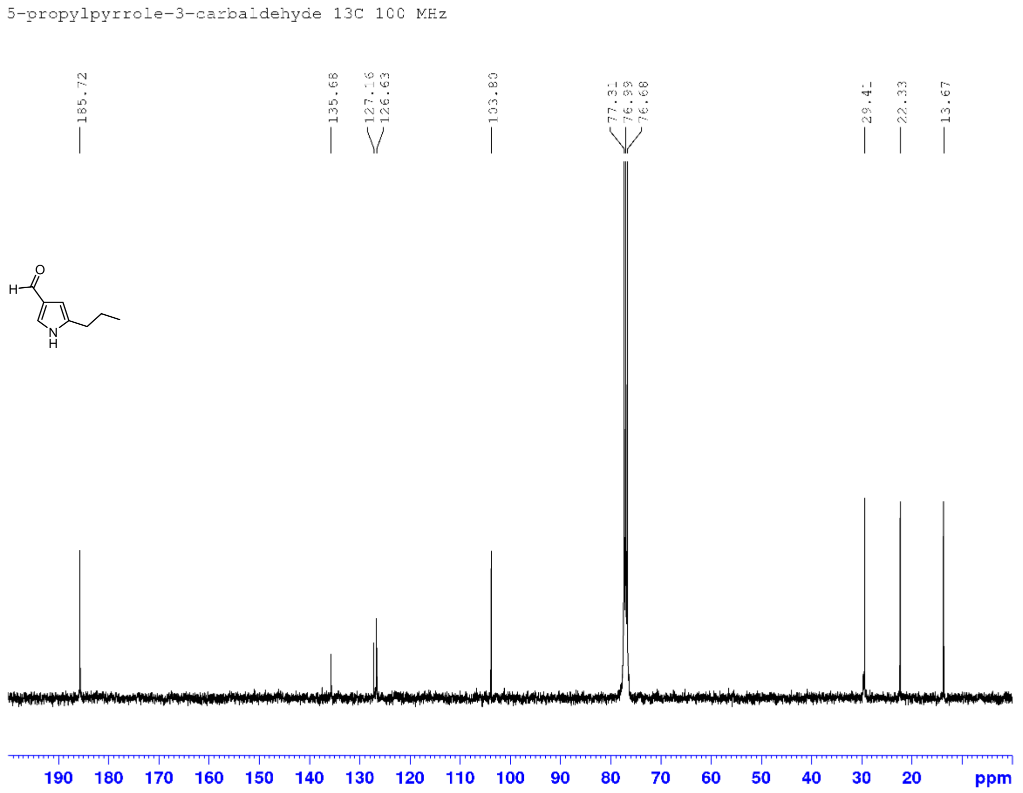

$^{13}C\{^1H\}$ NMR spectrum of **4r** ($CDCl_3$, 100 MHz)

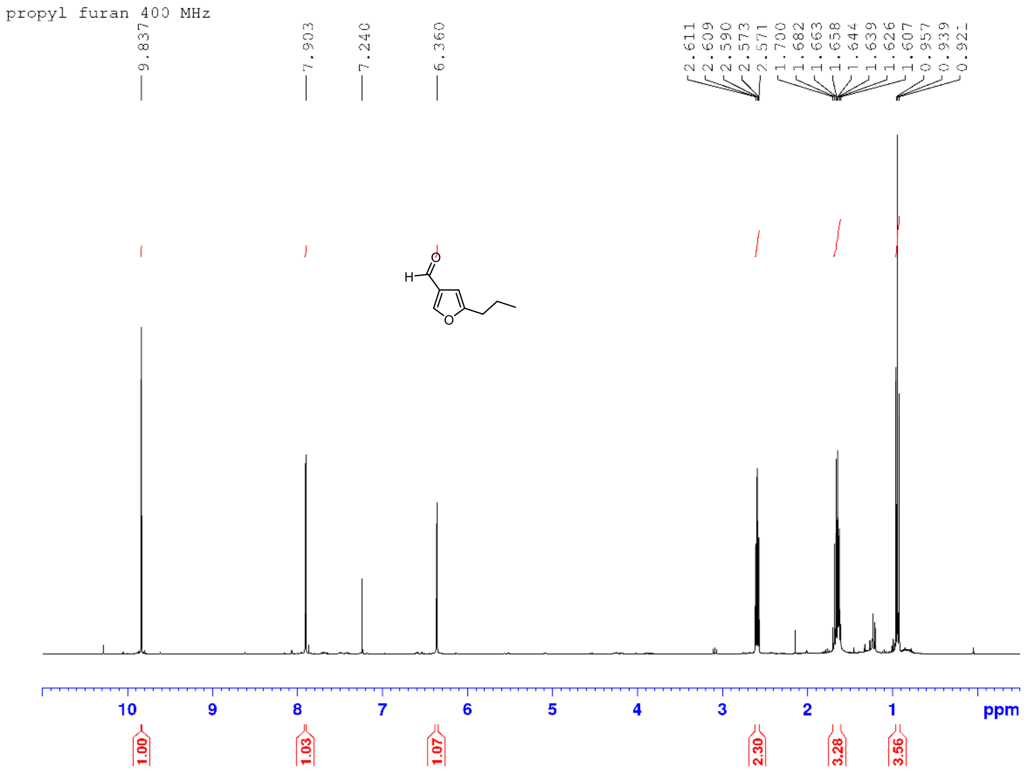

^{1}H NMR spectrum of **5r** ($CDCl_3$, 400 MHz)

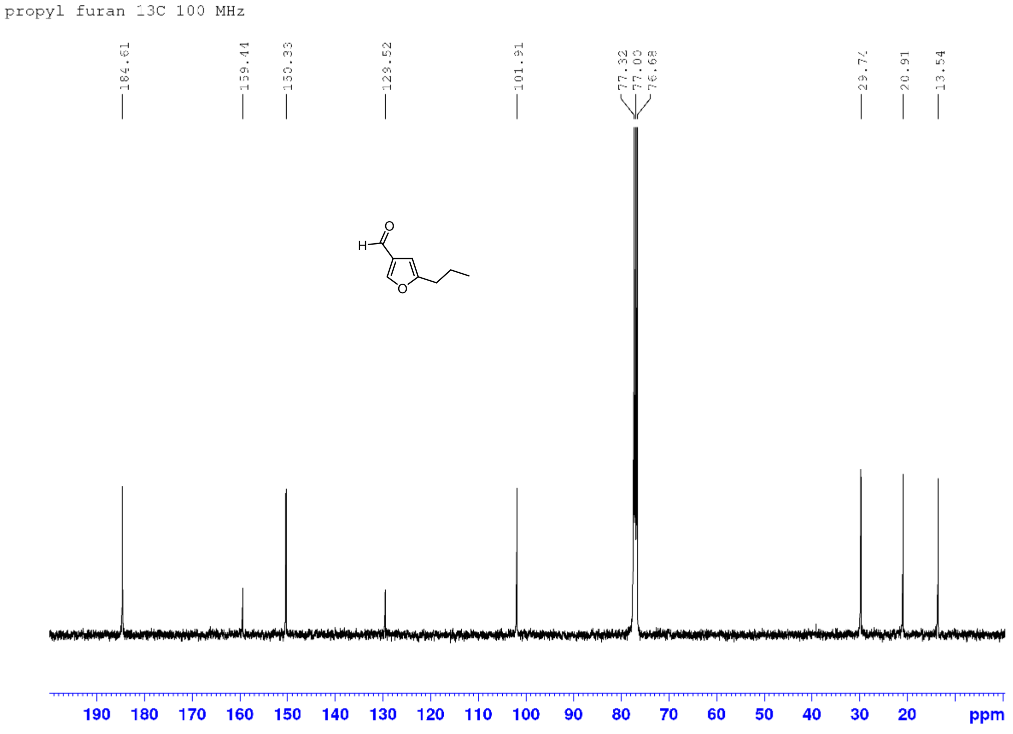

$^{13}C\{^{1}H\}$ NMR spectrum of **5r** ($CDCl_3$, 100 MHz)

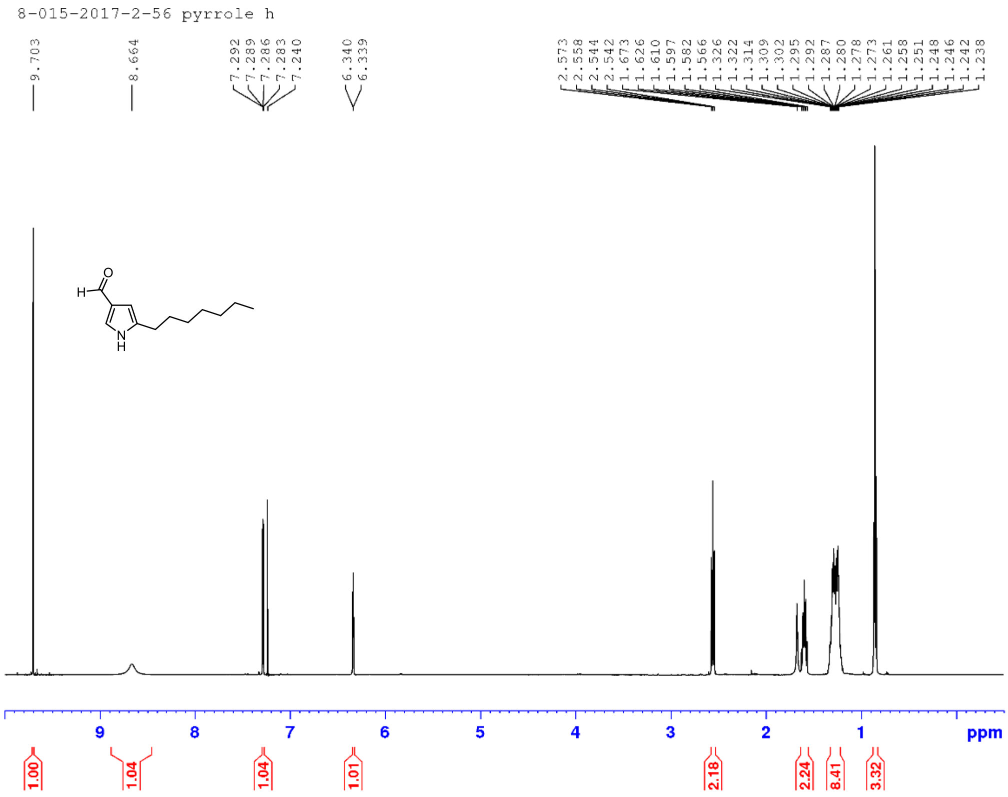

^{1}H NMR spectrum of **4s** ($CDCl_3$, 500 MHz)

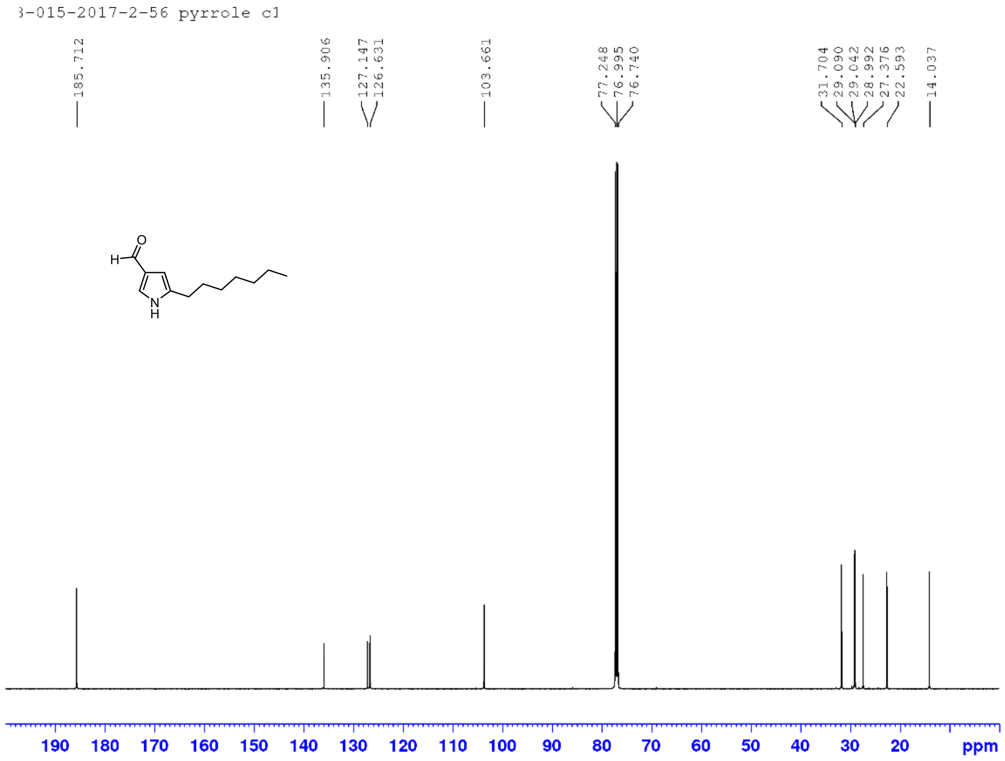

$^{13}C\{^1H\}$ NMR spectrum of **4s** ($CDCl_3$, 125 MHz)

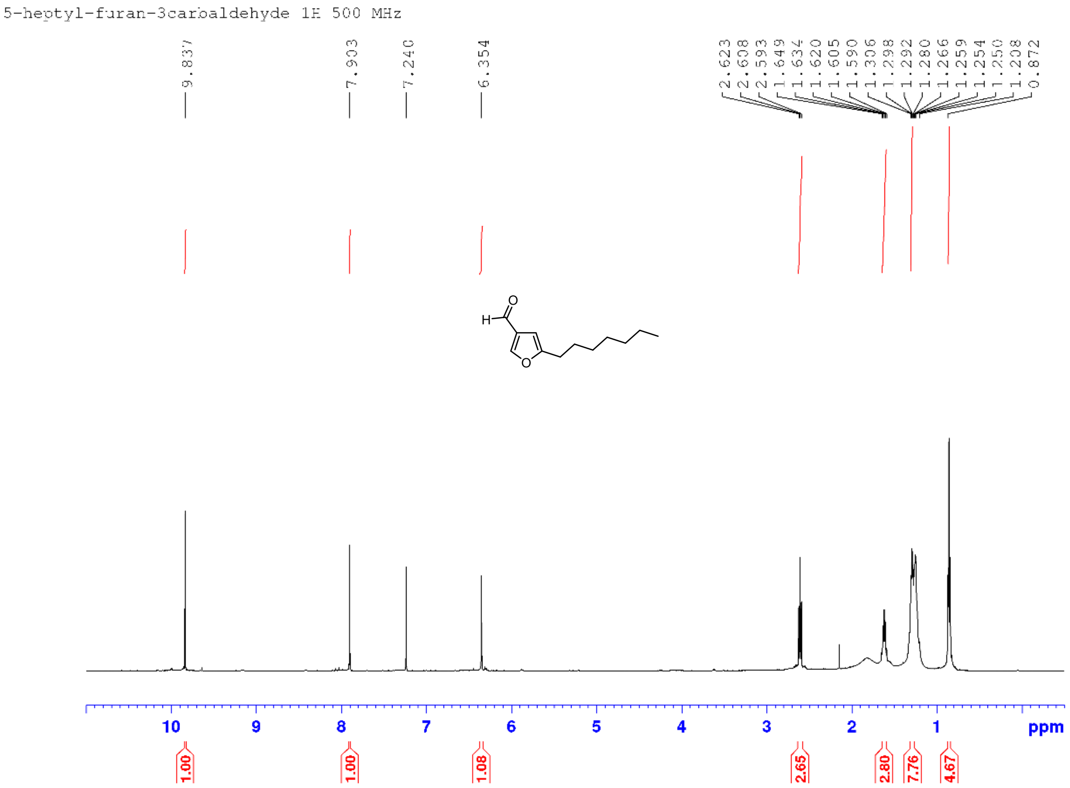

^{1}H NMR spectrum of **5s** ($CDCl_3$, 500 MHz)

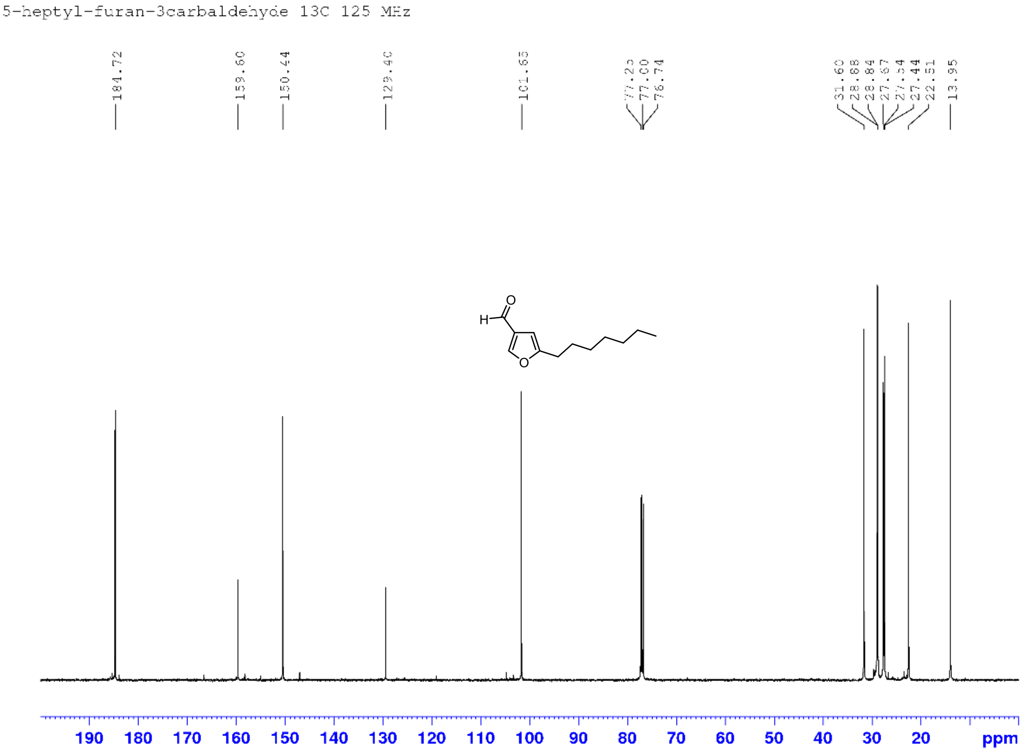

$^{13}C\{^{1}H\}$ NMR spectrum of **5s** ($CDCl_3$, 125 MHz)

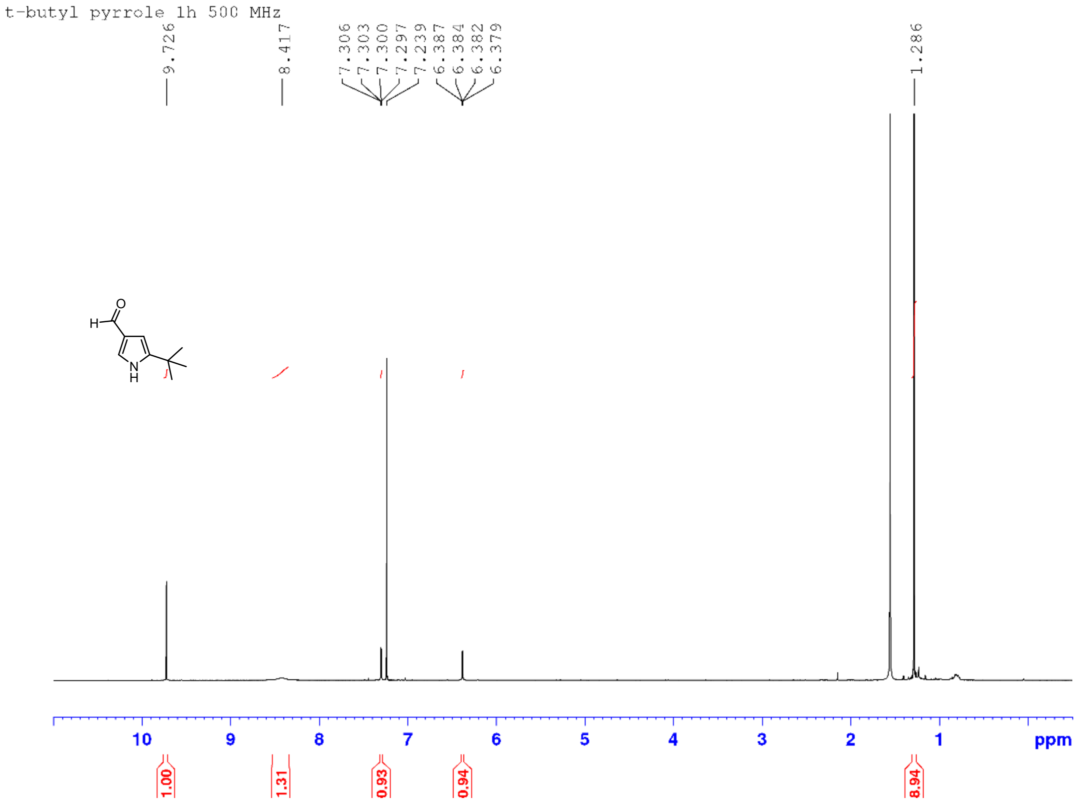

^{1}H NMR spectrum of **4t** ($CDCl_3$, 500 MHz)

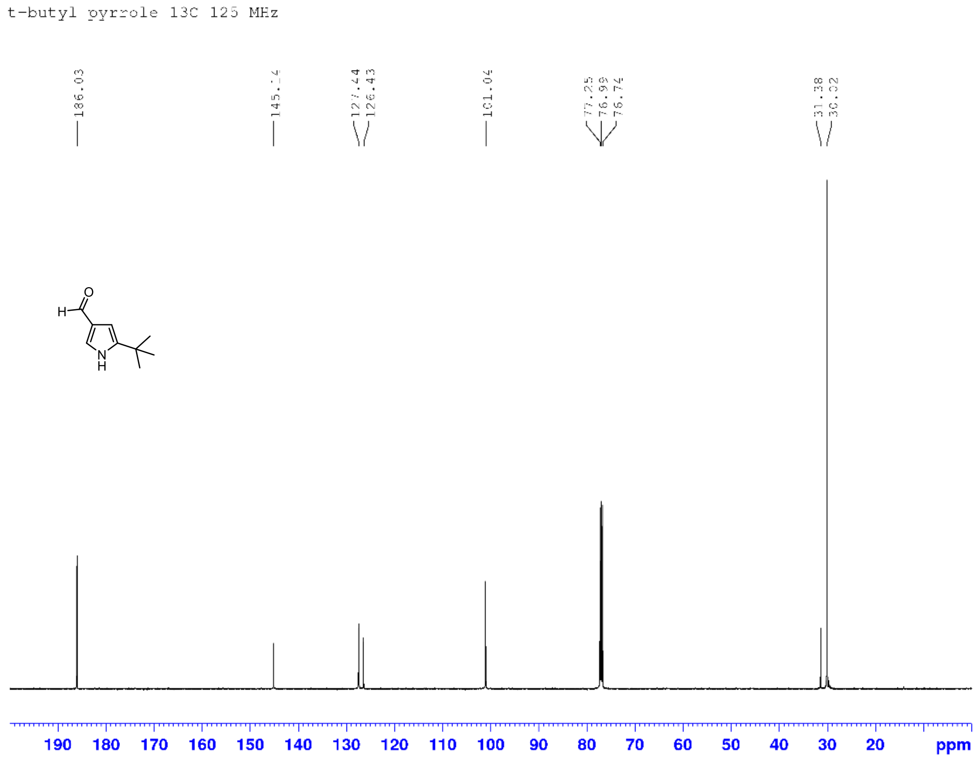

$^{13}C\{^{1}H\}$ NMR spectrum of **4t** ($CDCl_3$, 125 MHz)

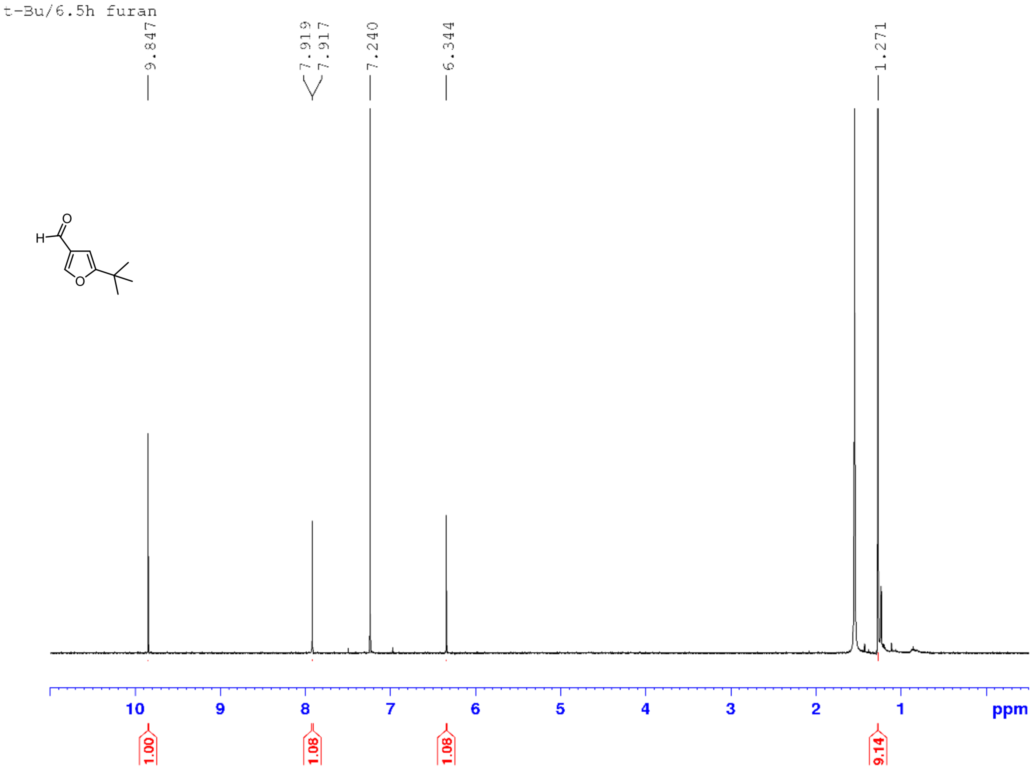

^{1}H NMR spectrum of **5t** ($CDCl_3$, 400 MHz)

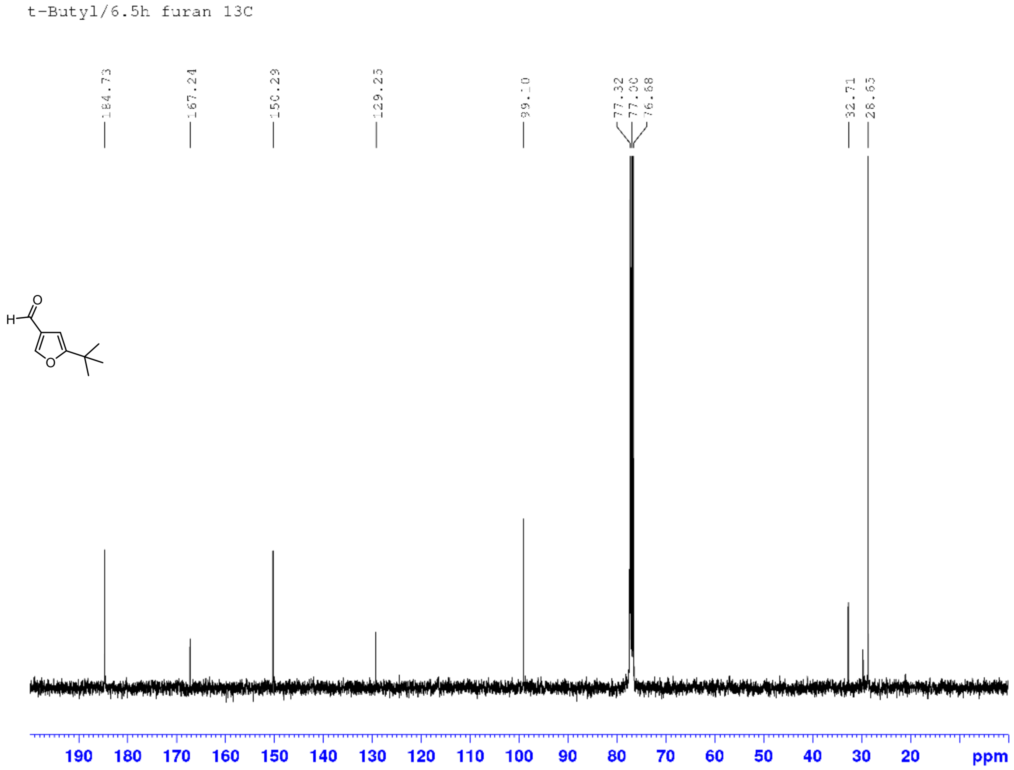

$^{13}C\{^{1}H\}$ NMR spectrum of **5t** ($CDCl_3$, 100 MHz)

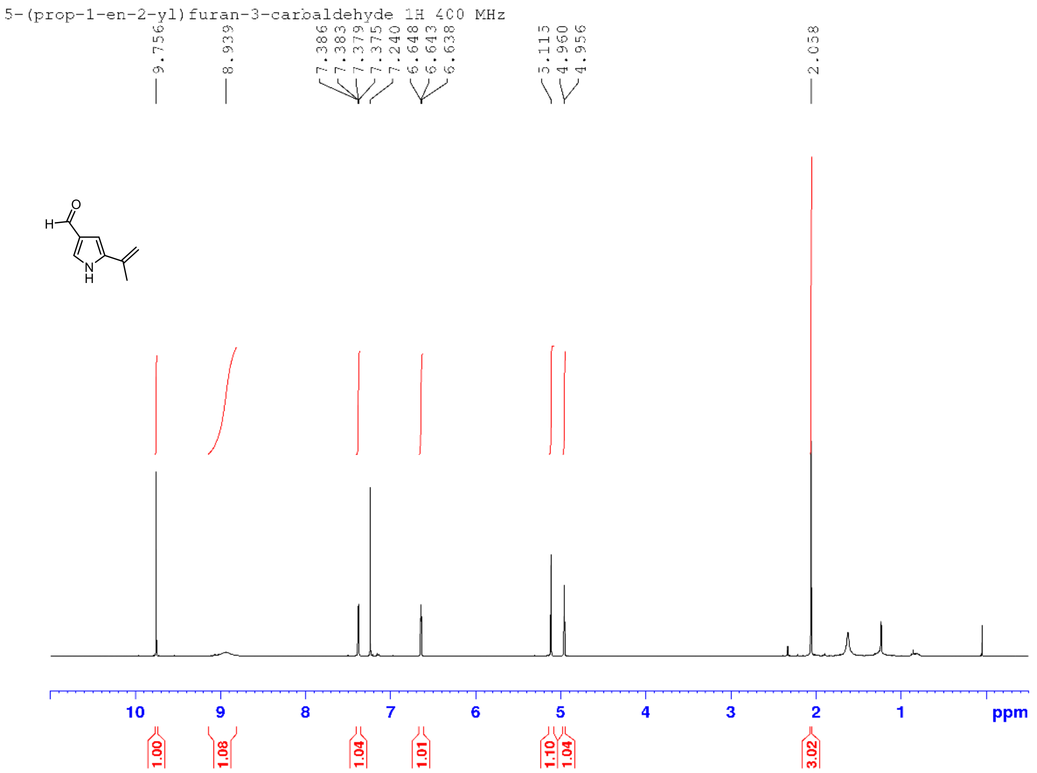

^{1}H NMR spectrum of **4u** ($CDCl_3$, 400 MHz)

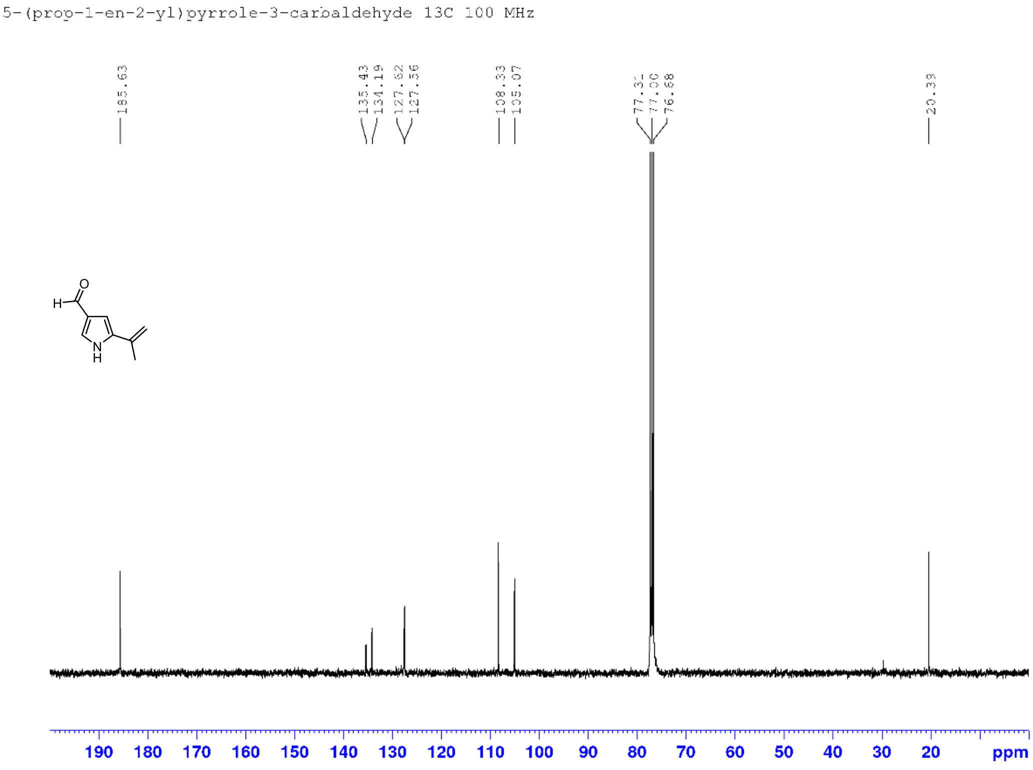

^{13}C{^{1}H} NMR spectrum of **4u** ($CDCl_3$, 100 MHz)

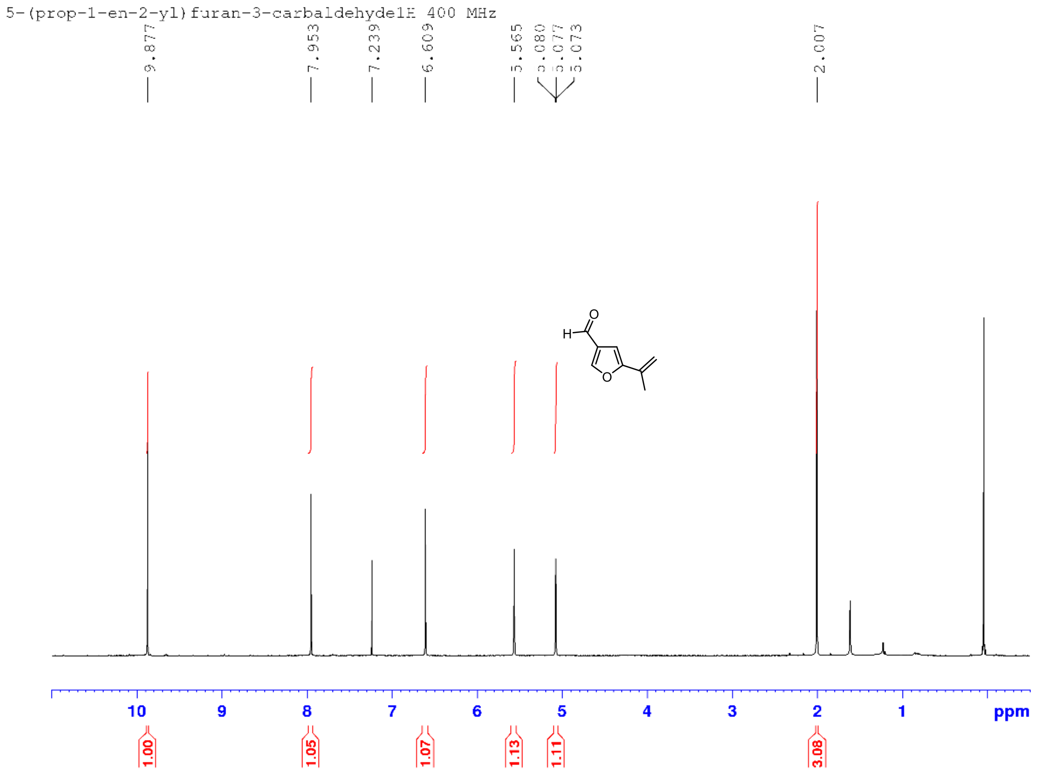

^{1}H NMR spectrum of **5u** ($CDCl_3$, 400 MHz)

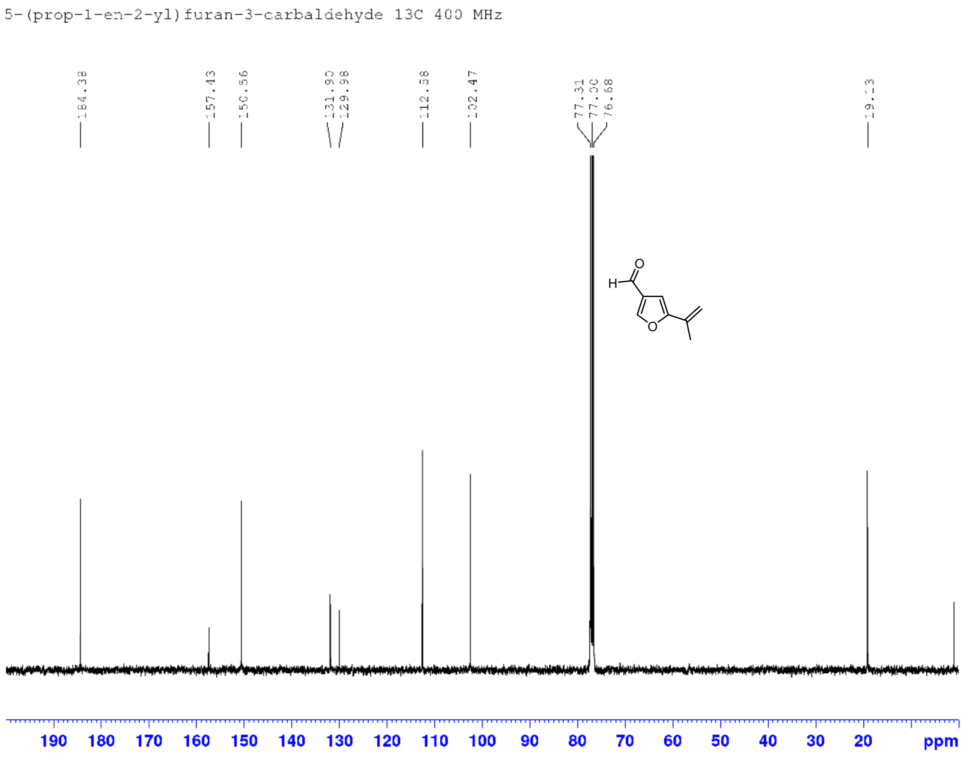

$^{13}C\{^{1}H\}$ NMR spectrum of **5u** ($CDCl_3$, 100 MHz)

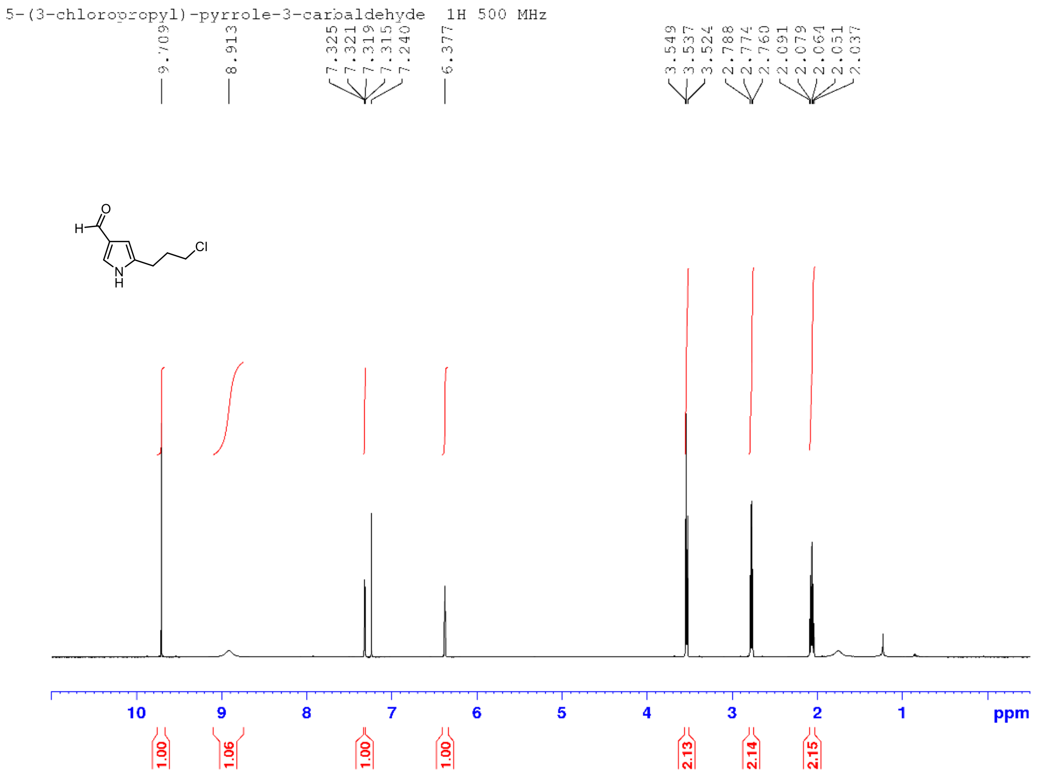

^{1}H NMR spectrum of **4v** ($CDCl_3$, 500 MHz)

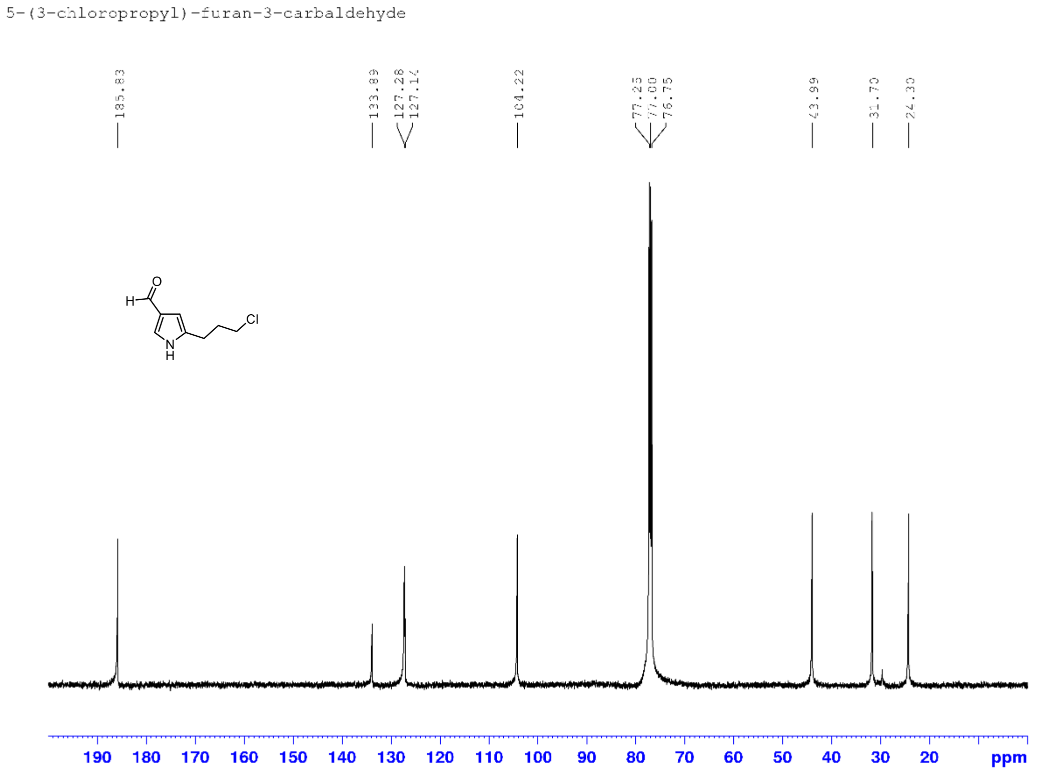

$^{13}C\{^1H\}$ NMR spectrum of **4v** ($CDCl_3$, 125 MHz)

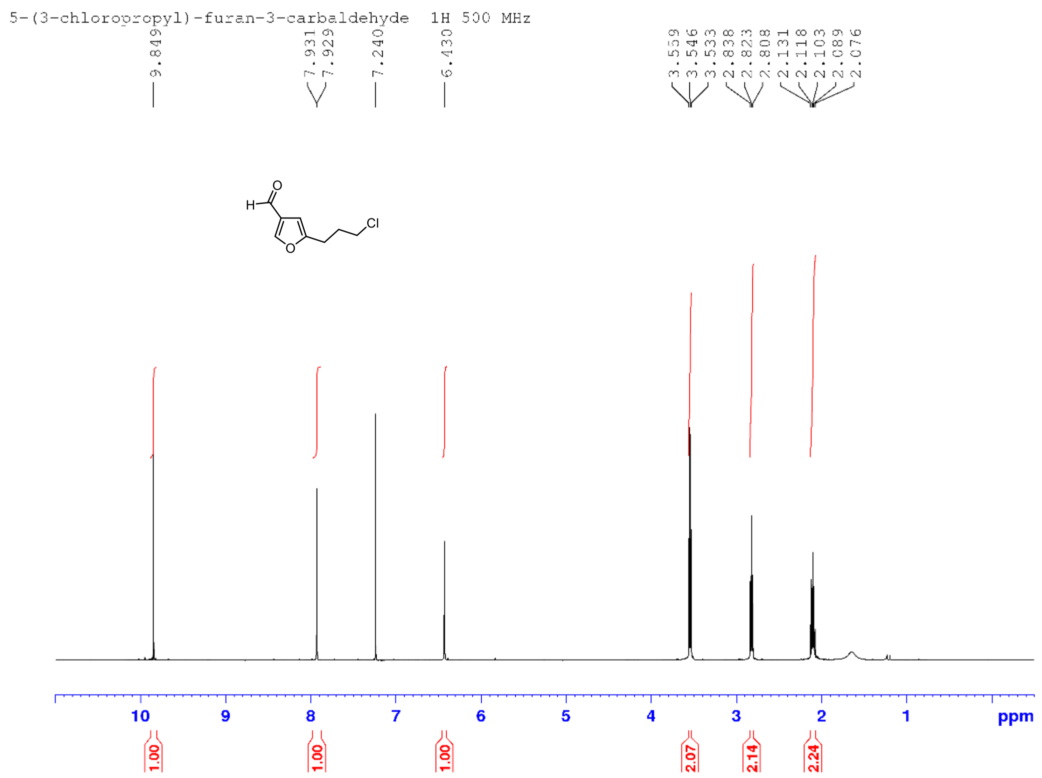

^{1}H NMR spectrum of **5v** ($CDCl_3$, 500 MHz)

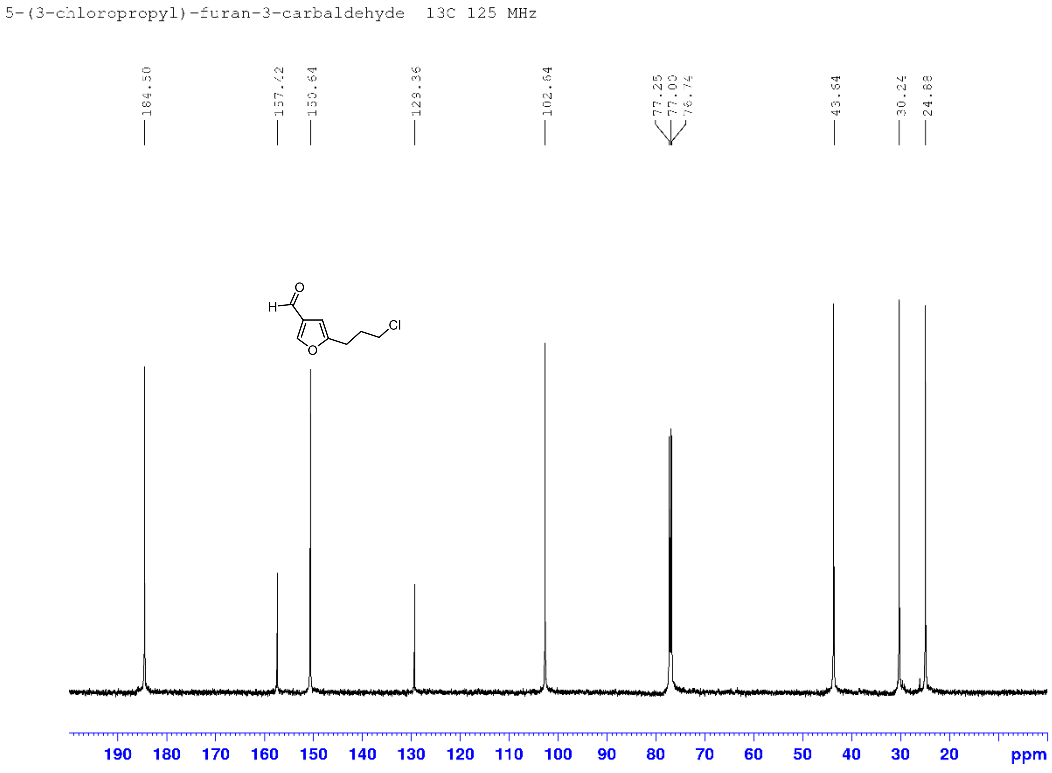

^{13}C{^{1}H} NMR spectrum of **5v** ($CDCl_3$, 125 MHz)

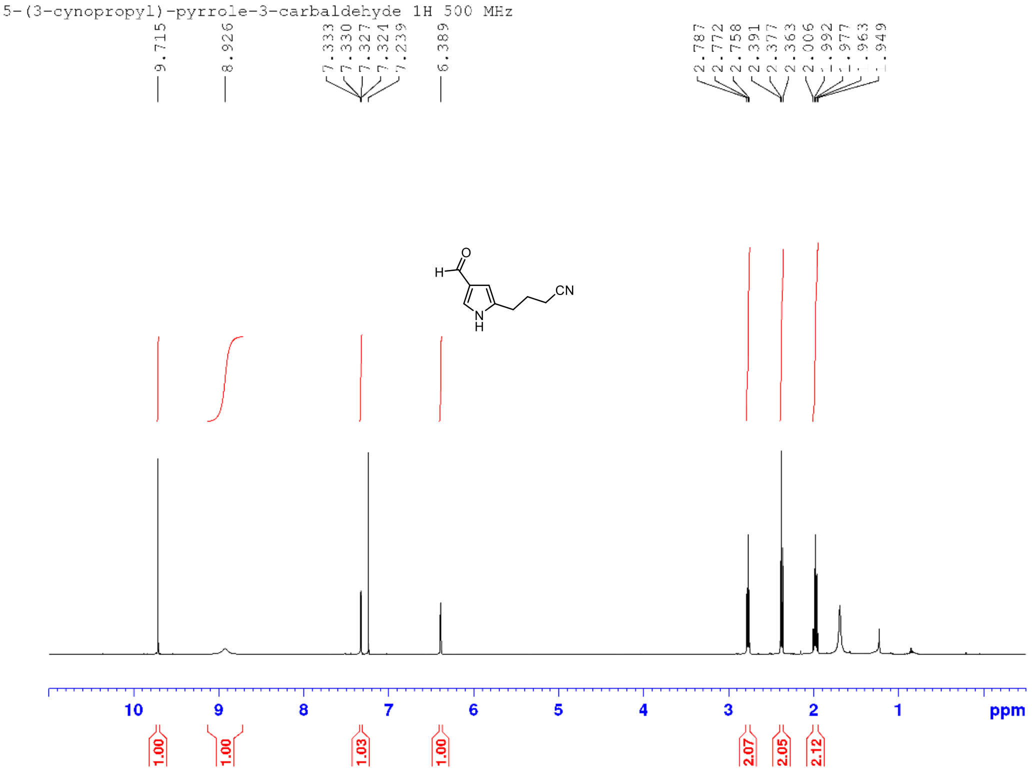

^{1}H NMR spectrum of **4w** ($CDCl_3$, 500 MHz)

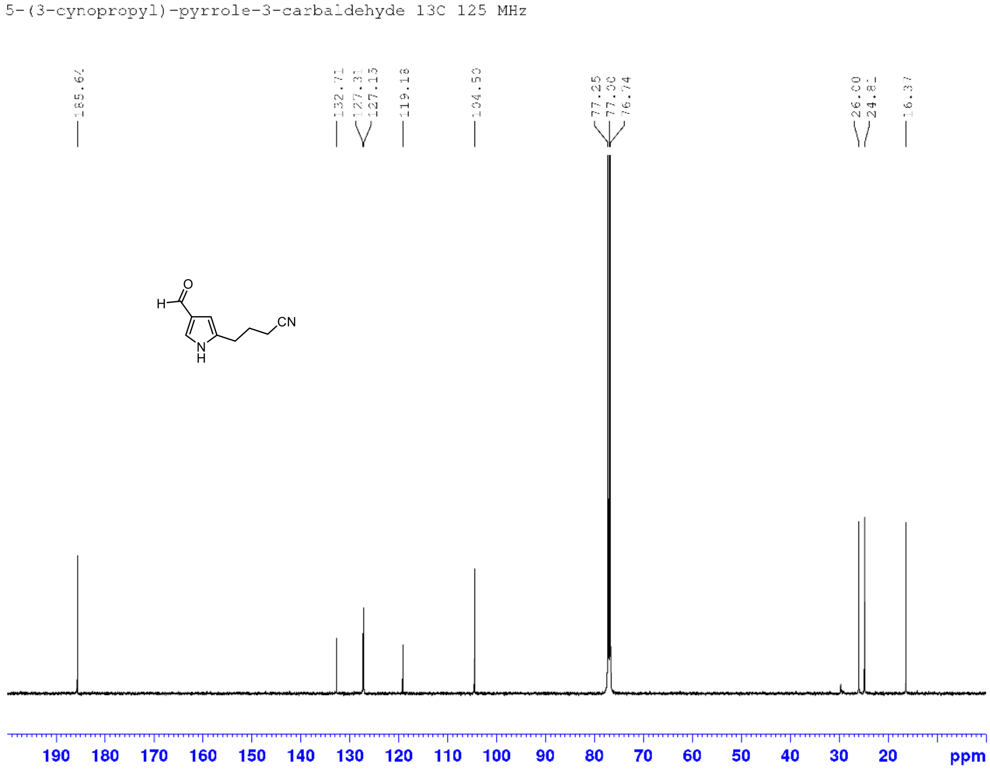

^{13}C{^{1}H} NMR spectrum of **4w** ($CDCl_3$, 125 MHz)

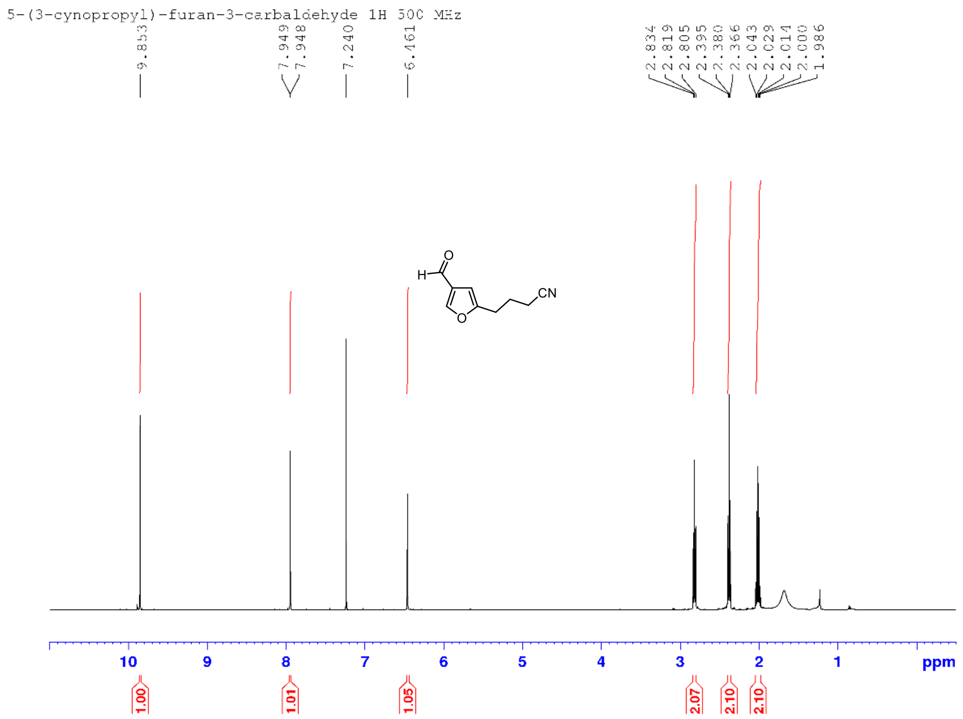

^{1}H NMR spectrum of **5w** ($CDCl_3$, 500 MHz)

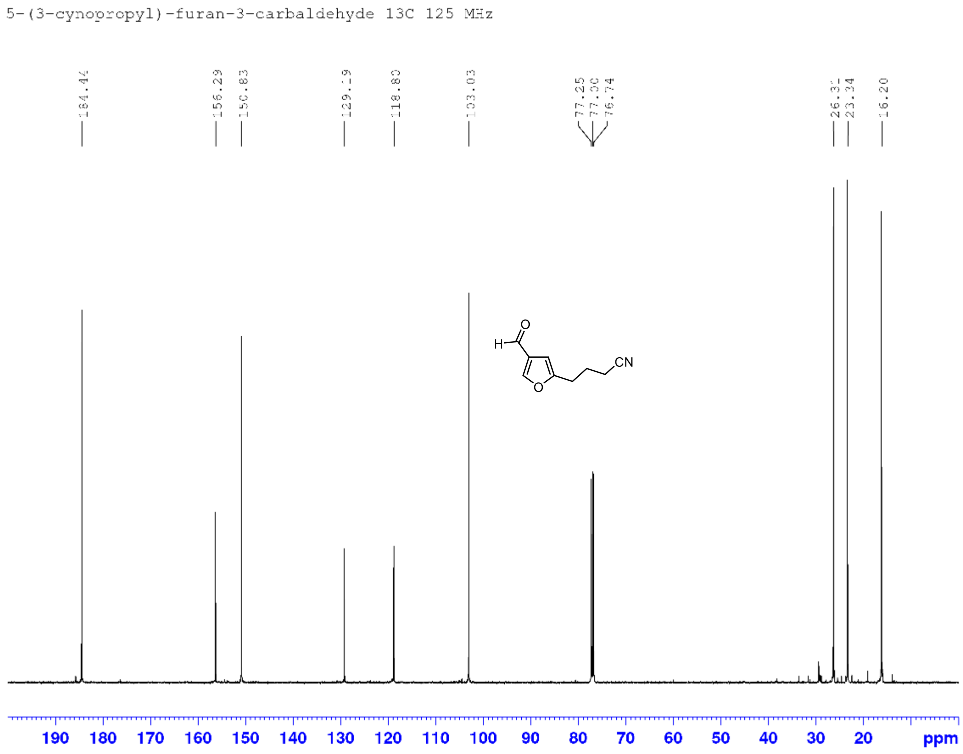

$^{13}C\{^{1}H\}$ NMR spectrum of **5w** ($CDCl_3$, 125 MHz)

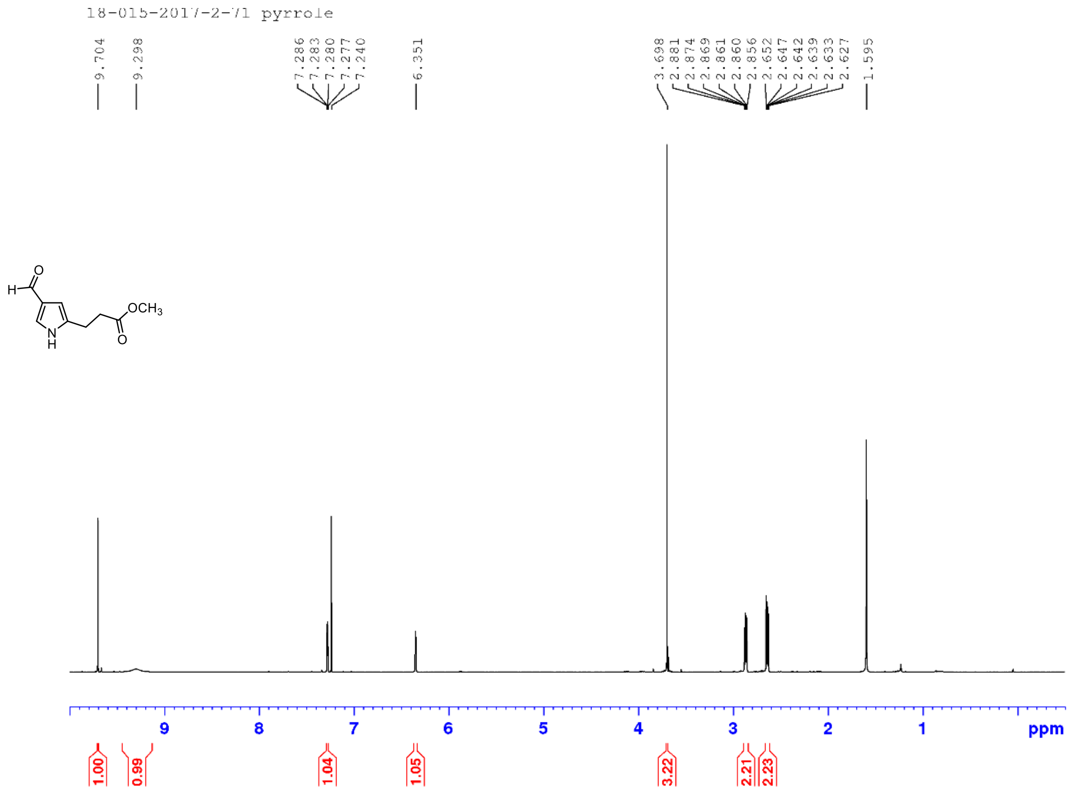

^{1}H NMR spectrum of **4x** ($CDCl_3$, 500 MHz)

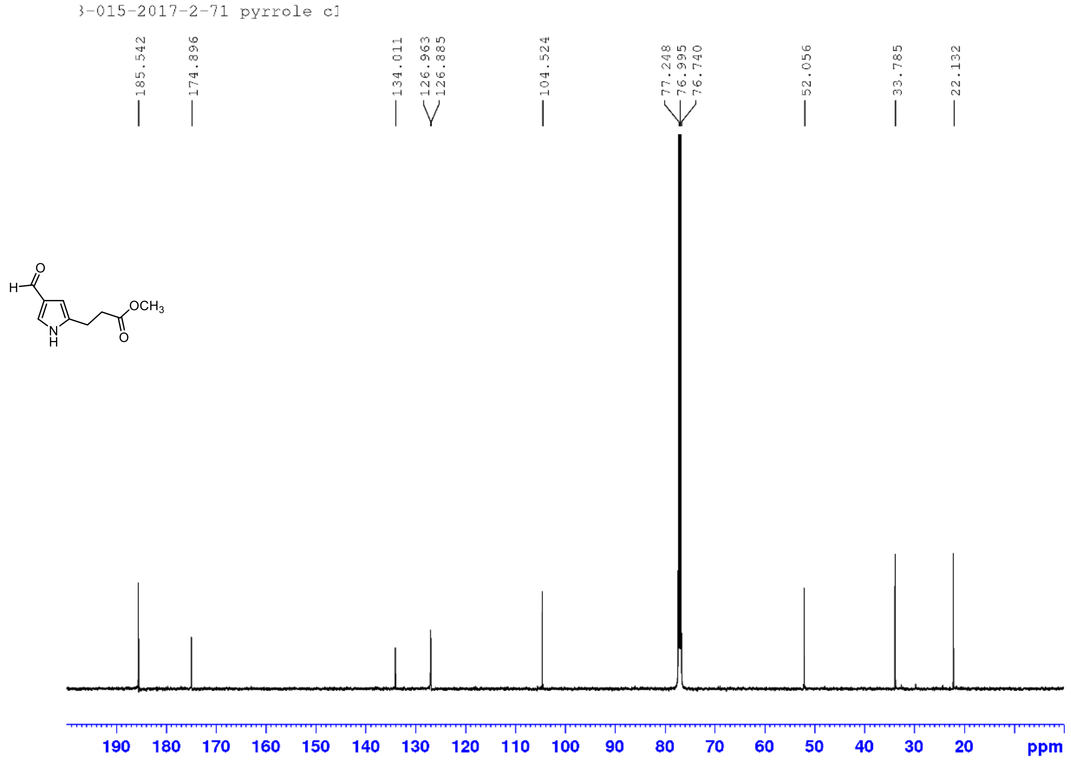

^{13}C{^{1}H} NMR spectrum of **4x** ($CDCl_3$, 125 MHz)

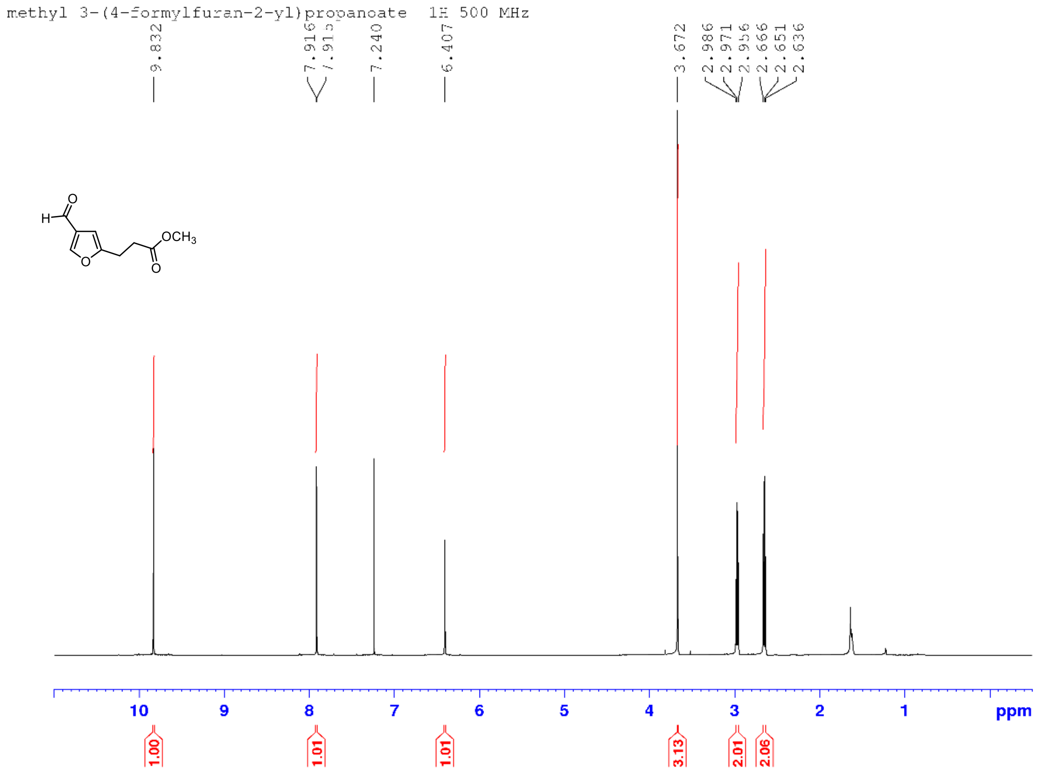

^{1}H NMR spectrum of **5x** (CDCl$_3$, 500 MHz)

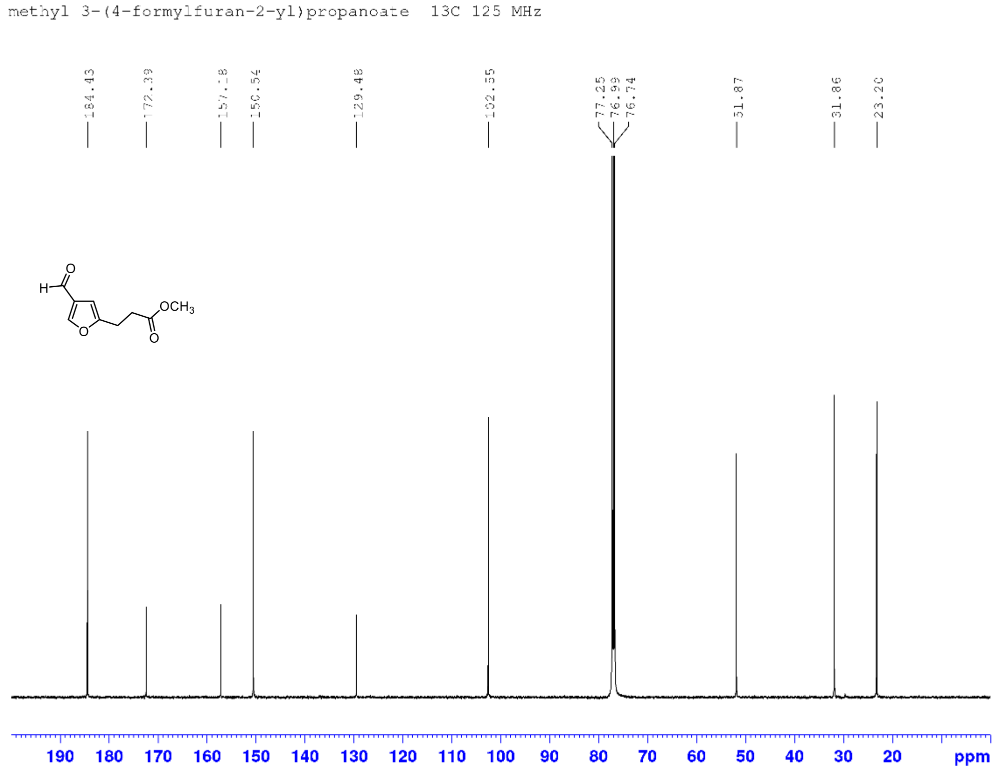

^{13}C{^{1}H} NMR spectrum of **5x** (CDCl$_3$, 125 MHz)

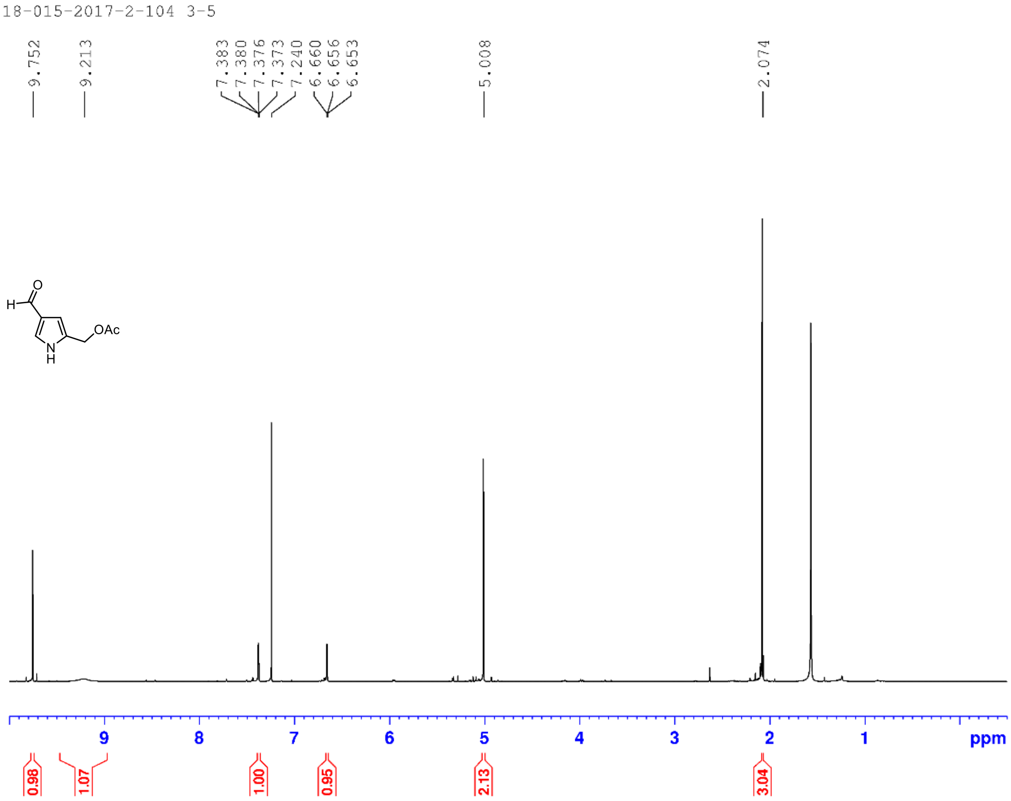

^{1}H NMR spectrum of **4y** ($CDCl_3$, 500 MHz)

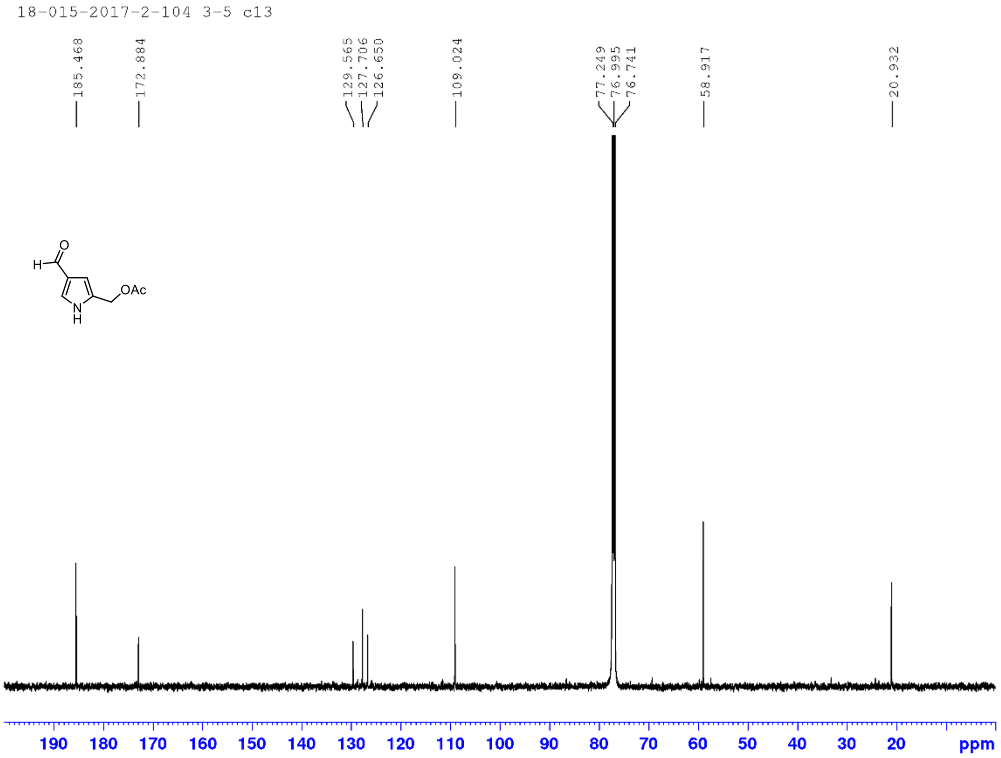

^{13}C{^{1}H} NMR spectrum of **4y** ($CDCl_3$, 125 MHz)

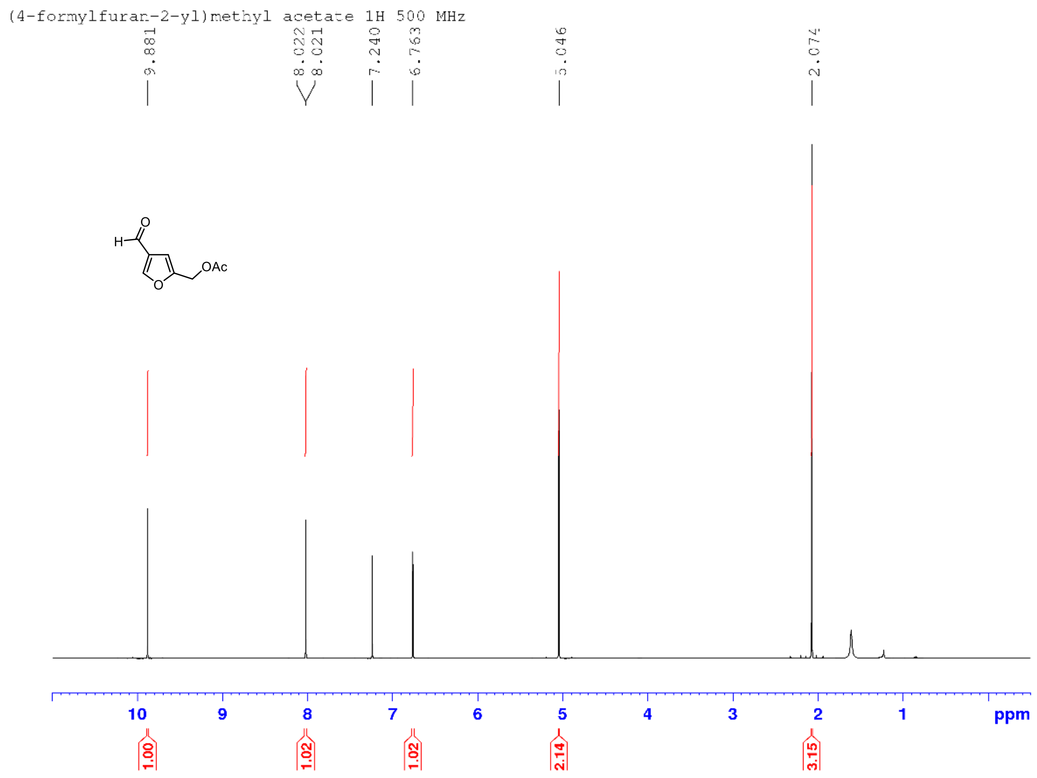

^{1}H NMR spectrum of **5y** ($CDCl_3$, 500 MHz)

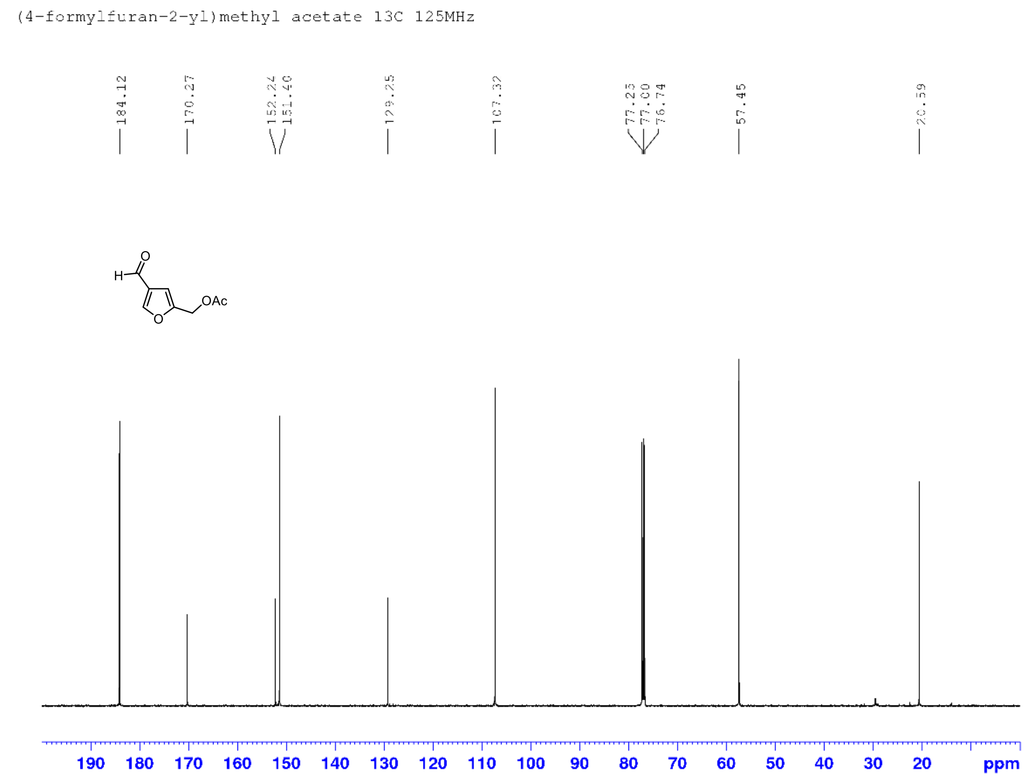

^{13}C{^{1}H} NMR spectrum of **5y** ($CDCl_3$, 125 MHz)

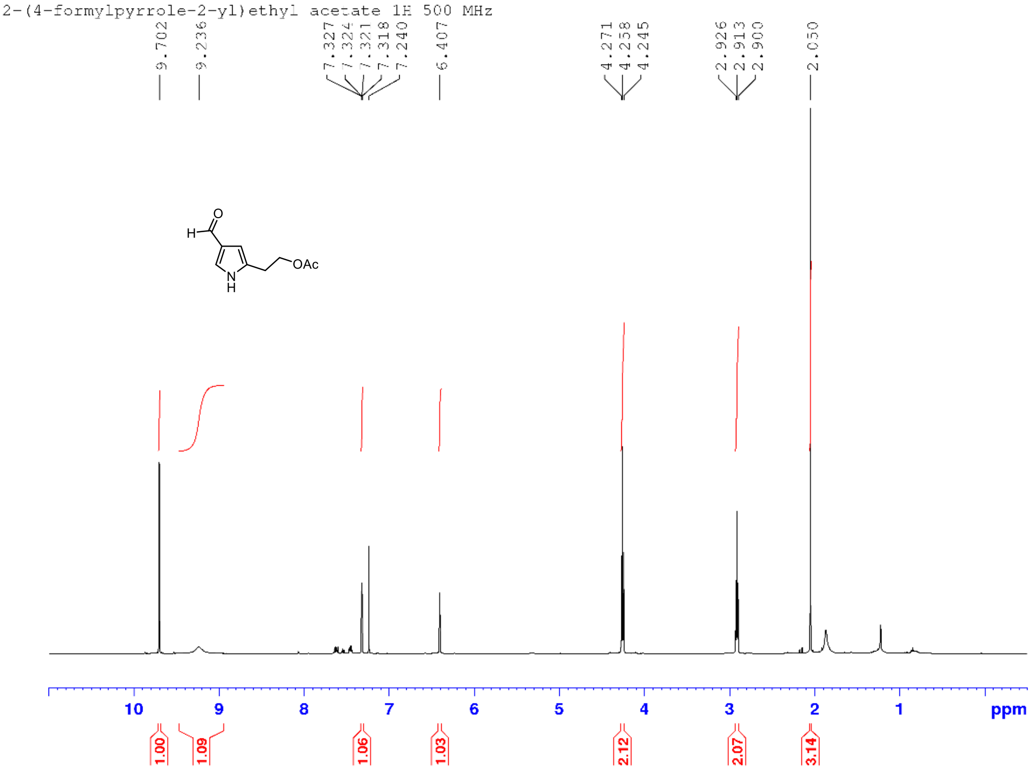

^{1}H NMR spectrum of **4z** ($CDCl_3$, 500 MHz)

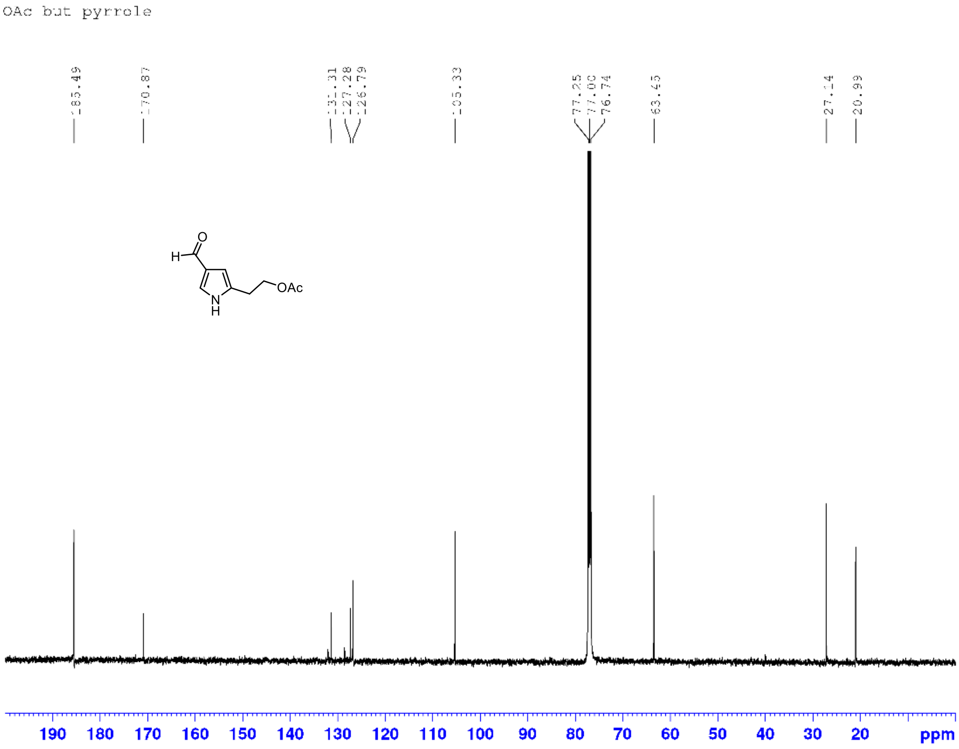

$^{13}C\{^1H\}$ NMR spectrum of **4z** ($CDCl_3$, 125 MHz)

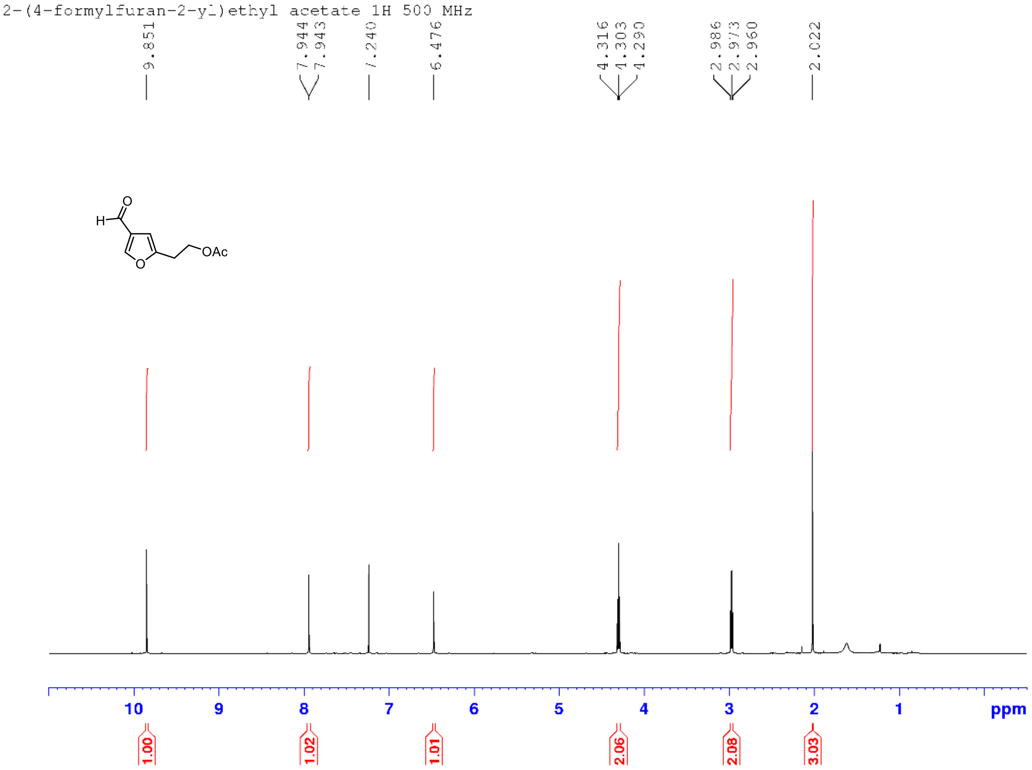

^{1}H NMR spectrum of **5z** ($CDCl_3$, 500 MHz)

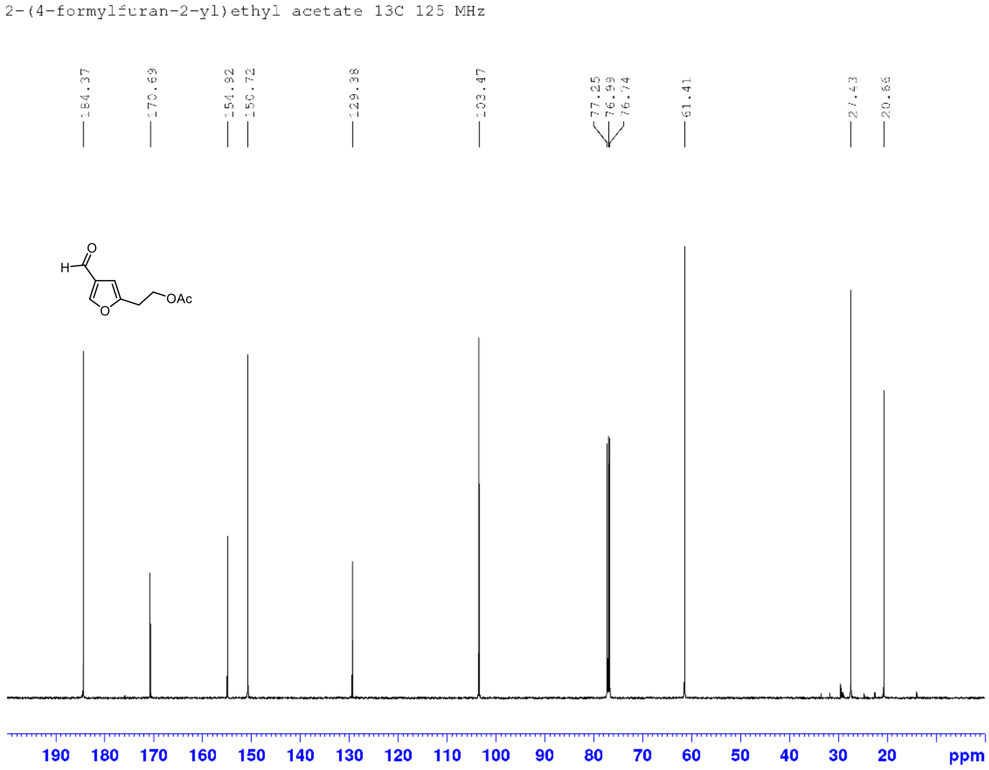

^{13}C{^{1}H} NMR spectrum of **5z** ($CDCl_3$, 125 MHz)

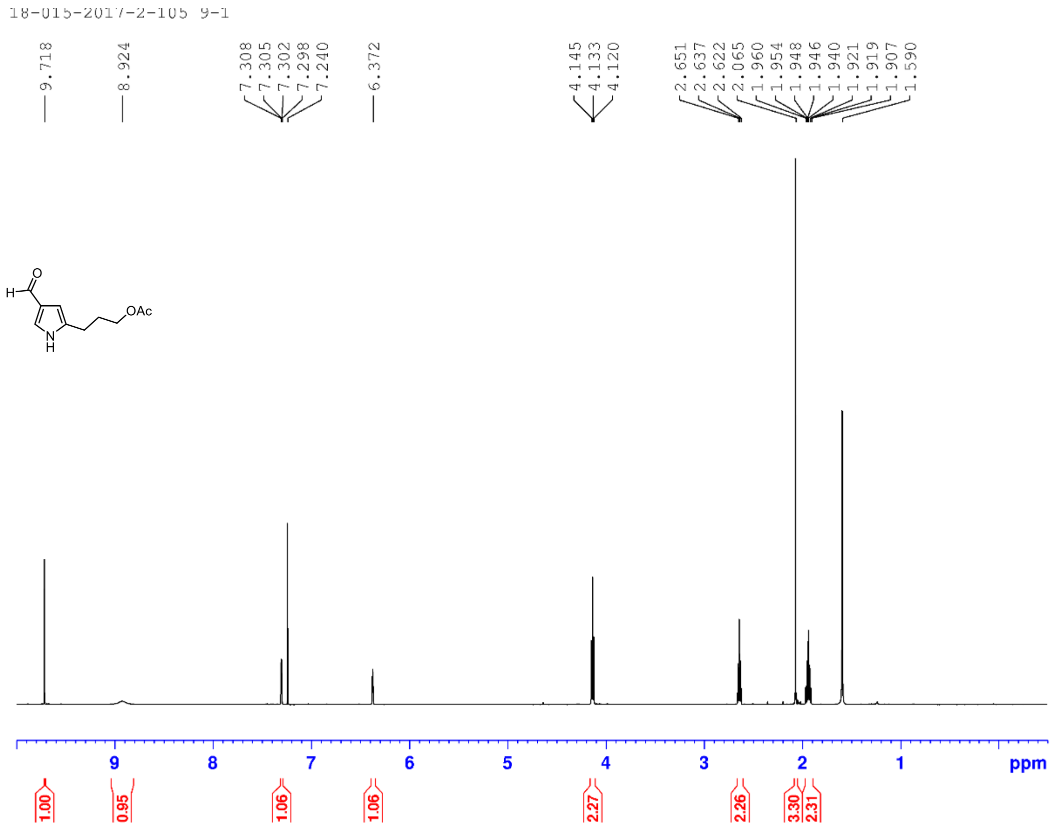

^{1}H NMR spectrum of **4a'** ($CDCl_3$, 500 MHz)

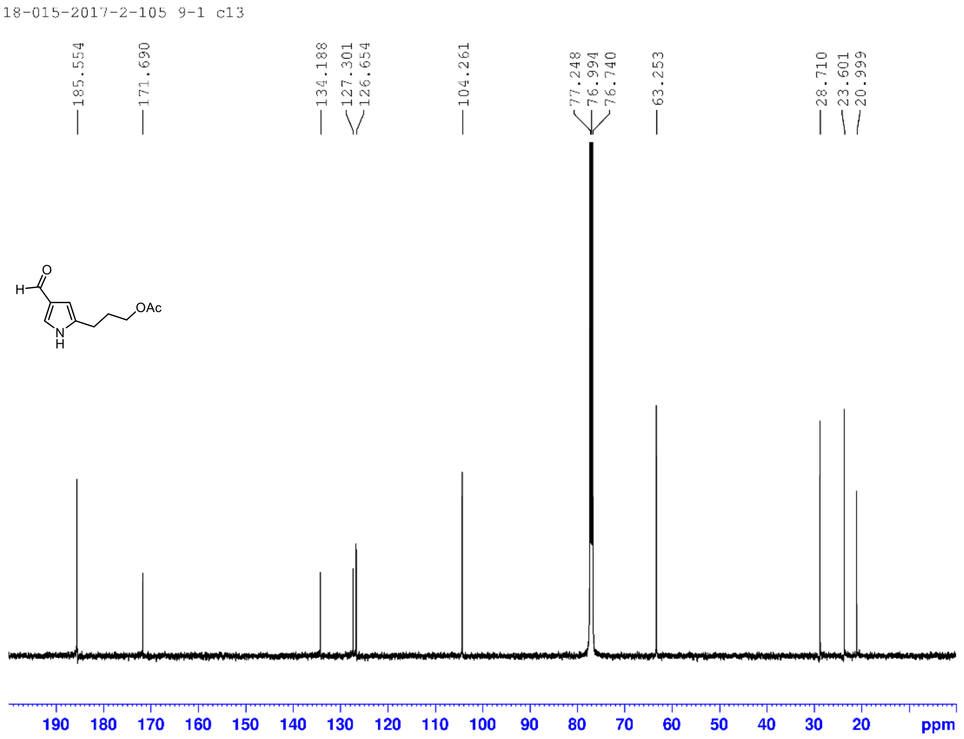

^{13}C{^{1}H} NMR spectrum of **4a'** ($CDCl_3$, 125 MHz)

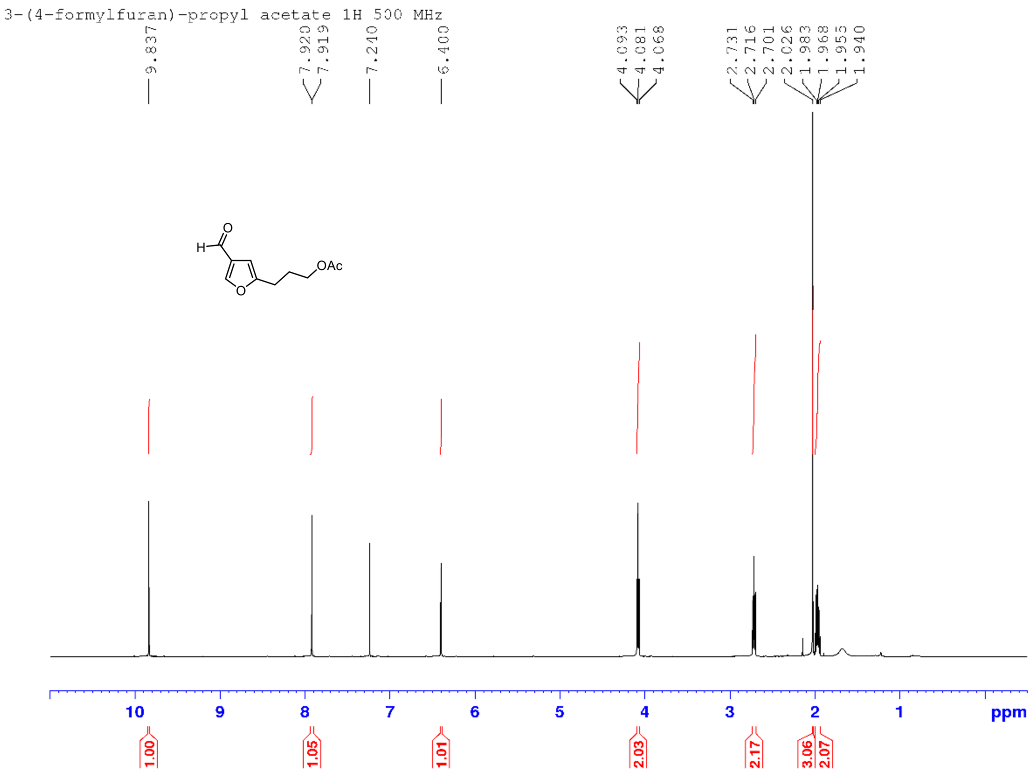

^{1}H NMR spectrum of **5a'** ($CDCl_3$, 500 MHz)

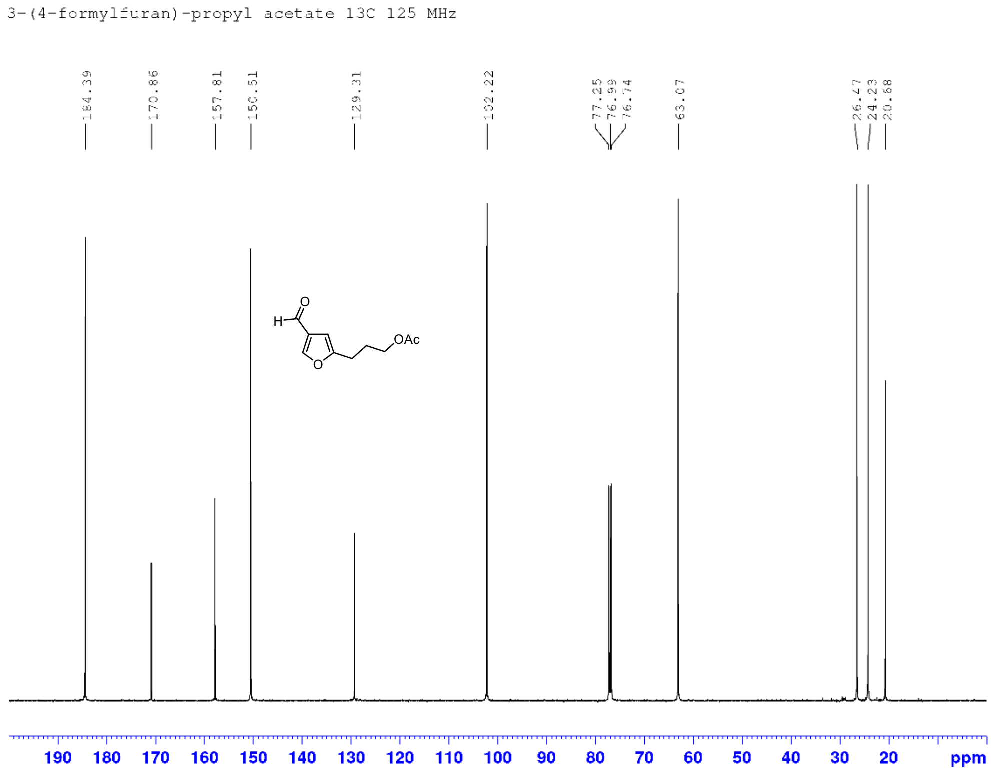

^{13}C{^{1}H} NMR spectrum of **5a'** ($CDCl_3$, 125 MHz)
